# Does the growing of Bt maize change abundance or ecological function of non-target animals compared to the growing of non-GM maize? A systematic review

**DOI:** 10.1186/s13750-022-00272-0

**Published:** 2022-06-06

**Authors:** Michael Meissle, Steven E. Naranjo, Jörg Romeis

**Affiliations:** 1 Agroscope, Research Division Agroecology and Environment, Reckenholzstrasse 191, 8046 Zurich, Switzerland; 2 USDA-ARS, Arid-Land Agricultural Research Center, 21881 North Cardon Lane, Maricopa, AZ 85138 USA

**Keywords:** *Bacillus thuringiensis*, Corn, Critical appraisal, Cry protein, Environmental risk assessment, Genetic engineering, Meta-analysis, Non-target organisms, Sensitivity analysis, Systematic literature search

## Abstract

**Background:**

Hundreds of studies on environmental effects of genetically modified (GM) crops became available over the past 25 years. For maize producing insecticidal proteins from *Bacillus thuringiensis* (Bt), potential adverse effects on non-target organisms are a major area of concern and addressed in risk assessments. Reviews and meta-analyses have helped various stakeholders to address uncertainties regarding environmental impacts of the technology. Many field studies from Europe and other parts of the world have been published in the last decade, and those data are often not covered by previous meta-analyses. Therefore, we conducted a systematic review to answer the question: “Does the growing of Bt maize change abundance or ecological function of non-target animals compared to the growing of non-GM maize?”

**Methods:**

Literature published until August 2019 was searched systematically in 12 bibliographic databases, 17 specialized webpages, and reference sections of 78 review articles. Defined eligibility criteria were applied to screen titles, abstracts, and full texts of the retrieved references. A custom-made database was developed with quantitative data on invertebrate abundance, activity density, or predation/parasitism rates. Eligible data that did not fit the quantitative database were captured in detailed tables and summarized narratively. For the first time, a critical appraisal scheme for field studies on non-targets in GM crops was developed to estimate the risk of bias (internal validity) and the suitability to answer the review question (external validity) of all primary data. Meta-analyses on different taxonomic levels, functional groups, and types of Bt maize were conducted. Untreated Bt maize was either compared with untreated non-Bt maize, or with insecticide-treated non-Bt maize. The influence of contributions by private sector product developers on reported effects was investigated.

**Review findings:**

The database on non-target effects of Bt maize field trials contains more than 7200 records from 233 experiments and 120 articles. Meta-analyses on different taxonomic levels revealed only few and often non-robust significant effect sizes when both Bt maize and non-Bt maize were untreated. Bt maize harboured fewer parasitoids (Braconidae, Tachinidae) of the European corn borer, the main target pest of Lepidoptera-active Bt maize, compared with non-Bt maize. Similarly, sap beetles (Nitidulidae), that are associated with Lepidoptera damage, were recorded less in Bt maize. In some analyses, a negative effect of Bt maize was observed for rove beetles (Staphylinidae) and hoverflies (Syrphidae) and a positive effect for ladybeetles (Coccinellidae), flower bugs (Anthocoridae), and lacewings (Neuroptera). However, those effects were not consistent for different analyses and often related to individual articles. When untreated Bt maize was compared with pyrethroid-treated non-Bt maize, more effect sizes were significant. In particular, populations of predators were reduced after pyrethroid treatment, while few data were available for other insecticides. Funnel plots showed no evidence for publication bias and the analyses of private sector contribution revealed no evidence for influence of vested interests. Conclusions about potential effects of Bt maize on vertebrates or on animals inhabiting off-crop habitats were not possible, because only few such studies fitting the format of direct Bt/non-Bt comparisons on plot or field level were identified.

**Conclusions:**

The current work largely confirmed previously published results. The effects of Bt maize on the community of non-target invertebrates inhabiting maize fields were small and mostly neutral, especially when compared with the effects of broad-spectrum pyrethroid insecticide treatments.

**Supplementary Information:**

The online version contains supplementary material available at 10.1186/s13750-022-00272-0.

## Background

Genetically modified (GM) crops have been cultivated commercially since 1996 [1]. First generation GM crops provided the plant with resistance against Lepidoptera or Coleoptera pests by producing single Cry proteins derived from *Bacillus thuringiensis* (Bt) and/or tolerance to certain herbicides. In recent years, those products are being replaced by a variety of stacked and pyramided gene constructs combining multiple Bt Cry and vegetative insecticidal proteins (VIP) for insect resistance against Lepidoptera and Coleoptera as well as herbicide tolerance traits. The greatest variety of GM crops is grown in the USA with more than 175 transformation events approved for cultivation [2]. In contrast, commercial production in Europe is restricted to one single-gene product, event MON810, which is corn borer resistant maize expressing the *cry1Ab* gene from Bt [2, 3].

Before new GM crops can be grown commercially, they have to undergo environmental risk assessment [4]. When assessing insect-resistant GM crops, such as Bt crops, potential effects on non-target organisms are particularly important [5]. Beneficial organisms that contribute to important ecosystem services are acknowledged as an important protection goal [6, 7]. Laboratory and field studies conducted for regulatory dossiers have been supplemented by studies performed and published by the scientific community and it is difficult to keep track of the hundreds of studies dealing with environmental effects of GM crops that have been published over the past 25 years. This has been recognized by the European Commission. The call FP7-KBBE-2012–6 states that: “Environmental, health and socio-economic effects of the aforementioned GMOs have been the subject of scientific analysis, however a comprehensive review of national, EU and international research activities in this regard and in view of any potential benefits of GMOs is missing.” A list of review questions of high relevance for Europe was developed within the EU project GRACE (GMO Risk Assessment and Communication of Evidence) [8].

The current review focuses on Bt maize because (1) this crop is the most widely grown insect-resistant GM crop worldwide; (2) Bt maize was among the first GM crops grown commercially and therefore was subject to many scientific studies; and (3) it is currently the only GM crop of commercial relevance for Europe. The review covers field data of non-target animals, because a reduction of populations of valued organisms may lead to environmental harm, no matter if the underlying effects are direct or indirect, anticipated or unanticipated [9]. In addition to toxicity of the introduced Bt proteins, effects also may include food web effects as a result of the missing target species in Bt crops and effects resulting from changes in plant physiology or changes in crop management practices [9].

Although non-target effects of Bt crops in the field have been reviewed (and meta-analyzed) previously [10–17], the current review has several benefits. Many data from European field studies have been published in the last decade, and those data are not covered by earlier works. In addition, we followed the guidelines for systematic reviews of the Collaboration for Environmental Evidence (CEE) [18] and their adaptation for GMO risk assessment developed by GRACE [19]. Because systematic reviews ensure a high standard for rigour, objectivity, and transparency, they are of particular value for decision-makers [20, 21]. Transparency and availability of data and protocols was not always provided in previous reviews and meta-analyses. In addition, for the first time a critical appraisal scheme for non-target animals in field studies with GM crops was developed and applied to all data to estimate the risk of bias (internal validity) and the suitability to answer the review question (external validity).

The core of this systematic review is a set of meta-analyses that compared non-target invertebrates inhabiting Bt and non-Bt maize fields. Meta-analysis optimises statistical power for detecting treatment effects by combining data from multiple similar studies [22, 23]. This systematic review follows the protocol previously published by Meissle et al. [9]. Stakeholders from academic institutions, competent authorities, NGOs, and the private sector were involved in the development of the protocol and the discussion of preliminary results [24].

## Objective of the review

The question we address in this systematic review is: “Does the growing of Bt maize change abundance or ecological function of non-target animals compared to the growing of non-GM maize?” The review question contains the following PICO elements: the population (P) is represented by non-target animals inhabiting field-grown maize or field margins; the intervention (I) is represented by the growing of Bt maize; a plot-to-plot or field-to-field comparison (C) with non-Bt maize is available; and the outcome (O) is represented by changes in abundance or ecological function. See “eligibility criteria” section below for further details. The following objectives were addressed:

- Identification of relevant literature by searching in different bibliographic databases, on webpages of relevant organizations, and reference sections of reviews.
- Development of a critical appraisal scheme to document the risk of bias (internal validity) and the relevance for the review question (external validity) of each dataset.
- Establishment of a database on quantitative, population related measures (abundance, activity density, predation and parasitism rates) for invertebrates in Bt maize compared with non-Bt maize plots or fields.
- Using the database for meta-analyses on different taxa and functional groups for different types of Bt maize (Lepidoptera-active, Coleoptera-active, or both) and different Cry proteins, including:
  o Exploration of potential effect modifiers, such as critical appraisal levels, plot size, years of Bt maize cultivation, and private sector contribution.
  o Specific analyses of taxonomic subgroups (including species), individual sampling methods, and juvenile life stages.
  o Analyses of invertebrates in Bt maize compared with non-Bt maize when non-Bt maize received insecticides that were not applied to Bt maize.
- Narrative summary of studies on vertebrates, studies on animals collected in field margins, studies with different pesticides applied to Bt and non-Bt maize, studies with response parameters not related to population sizes, such as biodiversity indices, and studies where data were not available in a format suitable for the quantitative database.

Box 1: definitions Reference: entry in a bibliographic database (Endnote or online databases) or in the references section of an article (= citation).

Article: journal article (paper), report, or other document, including reports submitted with regulatory dossiers (= publication).

Experiment: field study in one location (field, group of fields) in one year (= study).

Record: one line in our custom made database. Represents one taxon collected with one method in one year. Each record includes the mean, SD, and sample size from one particular set of Bt and corresponding non-Bt plots or fields.

## Methods

### Deviations from the protocol

This review follows the protocol previously published [9] with the following deviations:

*Data storage:* The database established for this systematic review and the critical appraisal scheme have been published in a companion article [25] and all other materials are provided as Additional files to this article. In the review protocol we stated that the review would be documented in CADIMA [9], but the system was not ready for use when we conducted the review.

*Searching:* Search strings were adapted to the specific requirements of the bibliographic databases to be searched. For full text databases, we searched for “nontarget” OR “non-target” and omitted the terms “natural enemy” OR predator OR parasitoid OR decomposer OR pollinator. The main reason was to simplify the search string for those databases. We did not conduct bibliographic database searches in languages other than English, but we included documents in other languages, such as Spanish, French, German, and Chinese when we identified them on webpages or in reference lists of articles. We searched in two additional bibliographic databases: SciELO Citation-Index and Scopus.

*Screening process:* In the protocol, we stated that a maximum of 200 references would be screened at the beginning of the screening process by a second reviewer as a quality assurance measure. During the course of the project, it became evident that kappa statistics have little value to ensure high quality of the screening process [18]. We thus screened as many references as possible at title/abstract level by a second reviewer with the given resources. For the initial literature search in 2014, approximately 1000 references were screened twice, while for the updates in 2019, all references (approximately 3000) were screened twice. We concentrated on the later references for double-screening as earlier articles have a higher chance of being included in reviews and previously published databases. We also double-checked decisions on full text level.

*Critical appraisal: *Plot size was not covered in the critical appraisal because no established guidelines exist for specific threshold values. We did, however, analyze the influence of plot size in the meta-analyses part of this review. We also did not include type and application of sampling methods in the critical appraisal because it was not possible to judge objectively which method is suitable or unsuitable for recording a specific taxon. Instead, we set a threshold for minimum amounts of recorded invertebrates per season to ensure that taxa that were only collected occasionally with a certain method were not included in the statistical analyses. Reporting bias was addressed with statistical tools rather than in the critical appraisal (funnel plot and meta-analyses on studies with private sector contribution compared with studies conducted by the public sector only).

*Data extraction:* In addition to data on finest reported taxonomic resolution and life stage, we also extracted, calculated, or estimated values for higher taxonomic levels (e.g. family or order) and all life stages combined to ensure a consistent analytical resolution for all experiments.

*Synthesis:* In addition to abundance, we also included measures of activity density and parasitism/predation function in the statistical analyses. Heterogeneity was generally low in the performed analyses, so further exploration of potential causes of heterogeneity was not necessary. We did not perform statistical analyses on the peer review status of extracted data. It became evident that many datasets could not be extracted directly from peer reviewed articles, but were extracted from associated non-peer reviewed work (e.g. reports or theses), or were directly supplied by the authors (raw data or aggregated data). In some cases, datasets supplied by authors also included additional taxa that were not mentioned explicitely in the respective peer reviewed articles. It would thus be difficult to decide if a specific data point should be considered peer reviewed or not.

### Search for articles

To find original data relevant to our review question, we searched multiple bibliographic databases. We also screened reviews and websites for references (Box 1) that may point to original data.

#### Search terms and strings

The search string was developed to find articles dealing with the growing of Bt maize in the field. In the development of the review protocol [9], the string was tested in 4 different bibliographic databases and the results were compared with the references known from a previous meta-analysis by Naranjo [14]. This scoping exercise demonstrated the suitability of the string [9]. The final search string for abstracting databases (that do not search in full texts) included terms related to three parts:

1. Maize (maize, corn, *Zea mays*);
2. Field (field, plot, location, trial, farm-scale, scouting, trap, sampling, monitoring);
3. Bt-genetic modification (transgenic, Bt, *Bacillus thuringiensis*, GM, genetically modified, genetically engineered, Cry, VIP);

The three parts were connected with AND (mandatory), while the terms within each part were connected with OR (Additional file 1: Table S1.1). For searches in full-text databases (Google Scholar, JSTOR), we added another part containing search terms related to non-target animals:

4. Non target, nontarget.

#### Search limitations

All database searches were conducted with English terms only, because many articles in other languages have at least an English title and abstract. Non-English articles (Spanish, German, French, Chinese) discovered in database and specialist searches and reference screening of reviews (described below) were included, because we did not want to ignore any known relevant data. Abstracting databases were searched initially in December 2014 (search period: all years). Updates were performed in January 2019 (search period: 2014–2019) and August 2019 (search period: 2019).

#### Search sources: bibliographic databases

The following bibliographic databases were searched: Web of Science Core Collection, BIOSIS, Zoological Record, CAB Abstracts, Agricola, AGRIS, ProQuest Dissertations and Thesis A and I, BASE, Google Scholar, and JSTOR. For details on those databases, see Ref. [9]. In addition, the following databases were included: SciELO Citation Index (Clarivate Analytics, Philadelphia, USA; provided by Web of Science) and Scopus (Elsevier B.V.). Retrieved references were exported to Endnote X8 (Clarivate Analytics). The search strings used for each database and the number of references (hits) that were exported to Endnote are documented in Additional file 1: Table S1.1.

#### Search sources: specialist searches

Specialist searches were conducted on the following webpages related to environmental effects of GMOs [9]: regulatory agencies (European Food Safety Authority; Federal Office for the Environment FOEN, Switzerland; Bundesamt für Naturschutz, Germany; Bundesministerium für Gesundheit, Austria), project databases (Cordis; GMO-Safety; AMIGA), GM-crop databases (Bibliosafety by ICGEB; Center for Environmental Risk Assessment; PlantGeneRisk by Testbiotech; ISAAA), industry organisations (Europabio), and civil society organisations (GM watch; Third World Network; Friends of the Earth; Greenpeace Research Laboratories; Greenpeace International).

When searching these websites, all relevant references not identified through previous searches were downloaded and added to the Endnote library. In the EFSA Register of Questions, the search focused on applications for cultivation of Bt maize in Europe. The obtained list was compared with Devos et al. [26], who reviewed EFSAs work in the past 10 years. The details on the searched websites and the identified references are presented in Additional file 1: Table S1.2.

#### Other sources of information

Previous reviews and other articles without original data (e.g., regulatory documents, summeries, reports) identified during the literature screening process were checked for potentially useful additional references (see section below). Furthermore, articles received or identified as a result of personal communication also were evaluated for original data or additional references.

#### Search results: assembly of reference library

The references identified in the bibliographic database searches (Additional file 1: Table S1.1) were combined in one Endnote library. Duplicates were searched and eliminated first based on similarity of authors, title, and year, and second based on title, year, and page numbers. The second step was necessary because authors are often spelled out differently in the different databases (e.g., initials only *vs.* full names) and sometimes misspelled.

### Article screening and study eligibility criteria

#### Eligibility criteria

Inclusion criteria for this systematic review were refined based on Meissle et al. [9]:

Eligible populations: Natural populations of non-target animals. Non-target animals were defined as all animals except *Diabrotica* spp. (Coleoptera: Chrysomelidae) for Coleoptera-active Bt proteins and the Crambidae *Chilo partellus*, *Diatraea grandiosella*, *Diatraea saccharalis*, *Ostrinia nubilalis*, and *Ostrinia furnacalis*, as well as the Noctuidae *Busseola fusca*, *Helicoverpa armigera*, *Helicoverpa zea*, *Heliothis* spp., *Sesamia nonagrioides*, *Spodoptera exigua*, *Spodoptera frugiperda*, and *Striacosta albicosta* for Lepidoptera-active Bt proteins.

Eligible intervention: Bt maize (producing Cry and/or VIP proteins from *B. thuringiensis*) grown under open field conditions.

Eligible comparator: Non-Bt maize control with which Bt maize is compared.

Eligible outcome: Effects of Bt maize on non-target animal abundance or activity density, ecological function, species richness, biodiversity, community structure, or other measure. Regarding ecological function, experiments measuring predation and parasitization, which can be linked directly to the presence of animals (invertebrate natural enemies), were considered. Not relevant was the function of pollination, because maize is wind pollinated. Experiments measuring decomposition (e.g., biomass reduction over time) were not considered eligible because they cannot be linked to animals alone (microbes substantially contribute). Herbivory (e.g., damage assessment) related to target pests also was not considered, but herbivory related to non-targets was considered.

Eligible types of study design: Direct comparisons between Bt and non-Bt maize lines in replicated field-to-field comparisons, split field or plot experimental designs. Experimental designs with one field of Bt maize and one field of non-Bt maize divided into several plots were considered not replicated and thus excluded. This ensured that the unit of intervention (Bt-maize cultivation and/or insecticide treatment) is the same as the unit of analysis (plots or fields), minimizing clustering and non-random issues in the experimental design.

Additional criteria: Only articles with original data were considered. Duplicated references and references referring to the same data were only included once.

#### Screening process

Titles and abstracts were screened and articles (Box 1) with potential original data matching the eligibility criteria (described above) as well as potentially relevant reviews were labelled for full text evaluation. Duplicate references that were not detected automatically (e.g., because of differences in the spelling of titles or other errors) were excluded.

Full text versions of the identified articles were screened once more by applying the eligibility criteria described above. All references for which full texts were screened are listed in Additional file 2, where excluded references are presented with the reason for exclusion and included references are presented with a unique identifier number.

#### Consistency of eligibility decisions

All references and full texts were screened by one experienced main reviewer. In addition, a subset of randomly selected 10% of the references obtained in the first literature search in 2014 were screened by a second reviewer at the title/abstract level (1006 references). The references obtained in the updates in January and August 2019 were all screened by both reviewers at the title/abstract level (2933 references). Titles and abstracts that were excluded by the main reviewer but included by the second reviewer were checked again by both reviewers at full text level and a common decision was made after discussion between both reviewers. In addition, articles excluded on full text level were all double checked and descrepancies were solved within the team.

Decisions regarding inclusion or validity assessment of studies co-authored by a review team member were made by other team members that were not authors of the study.

### Data extraction

Of the articles containing original data relevant for this review, most reported abundance (e.g., by field counts or catches) or activity density (e.g., by traps) of non-target invertebrates inhabiting Bt and non-Bt maize fields (or plots). Therefore, we decided to quantitatively extract data that are related to population sizes of non-target invertebrates, such as abundance, activity density, parasitism rate, and predation rate. A purpose-built Microsoft Access 2016 database was used for data-entry and selection of records for statistical meta-analysis. The content of this database is published separately in Meissle et al. [25].

**Fig. 1:**
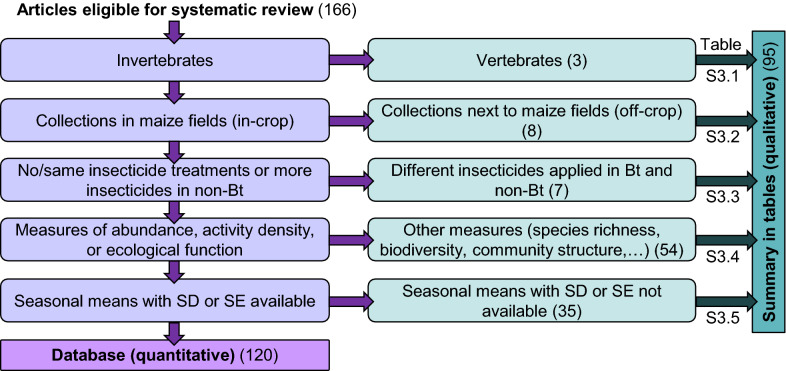
Criteria for quantitative data extraction (database for meta-analyses) and qualitative data extraction (summary in tables). Numbers indicate references in each of the categories of the narrative-only summary (on the right side) and in the database (at the bottom). Note: several references contained data for more than one category, e.g., abundance data for the database as well as biodiversity data for the narrative-only summary, and were thus counted multiple times

All experiments (Box 1) reported in articles eligible for this systematic review were evaluated for suitability for the database based on the decision tree in Fig. 1. To be entered into the database, experiments needed to fulfil the following criteria:

1. The studied population consisted of invertebrates. Experiments on vertebrates are summarized in Additional file 3: Table S3.1.
2. Assessments were conducted in maize fields (in-crop). Data on non-target invertebrates inhabiting field margins or other habitats near maize fields are presented in Additional file 3: Table S3.2.
3. The experiment included one or more of the following comparisons: Bt maize without insecticide *versus* control maize without insecticides (untreated/untreated), Bt maize without insecticides *versus* control maize with insecticides (untreated/treated), Bt maize with insecticides *versus* control maize with identical insecticides (treated/treated), or Bt maize with insecticides *versus* control maize with identical insecticides plus additional insecticides (treated 1/treated 1 + treated 2). One example of the latter case might be a seed treatment in Bt and non-Bt maize and a foliar spray only in the non-Bt maize for the target pest. Studies where Bt and non-Bt maize were treated with different insecticides are summarized in Additional file 3: Table S3.3.
4. The measured outcome was abundance, activity density, predation rate or parasitism rate. Outcome variables aggregating over invertebrate communities (e.g., species richness, biodiversity indices, community structure) or other measures (e.g., size, behaviour) are presented in Additional file 3: Table S3.4.
5. Means with SD or SE based on true replicates (N fields or plots) covering one cropping cycle (seasonal means) were provided for defined Bt/non-Bt comparisons or could be derived from the data presented in the article or the data supplied by the authors upon request. If such data were not available, the experiment was included in Additional file 3: Table S3.5.

#### Variables of the database

Data from experiments meeting the requirements for quantitative extraction (Fig. 1) were entered into the database as specified in [9] with a few amendments to the data structure (see [25], data file 2 for a complete list of variables). Each record of the database (Box 1) contains detailed information on the bibliography of the article, maize lines and Bt proteins, location of the experiment, experimental design, field management, insecticide treatment, comparison type, sampling method and procedure, recorded taxon, response variable, and critical appraisal.

For transparency and consistency, we established a set of rules as to which data were entered into the database. Those rules concern the taxonomic level, calculation of seasonal means and SDs, and the multiple use of data. Further details on data extraction rules are provided in Additional file 4.

#### Contacting authors

If crucial information was not available from the original article, or if datasets were not presented in a way directly useable for the database, the authors of the study were contacted and asked to provide the required information or the (raw) data. For 95 articles we tried to contact the authors and for 51 articles, we received the requested information or at least parts of the full request. For 27 articles the authors replied but could not provide the requested data and for 17 articles, we did not get a reply to our request, not even after a reminder was sent.

#### Consistency of data extraction

Datasets from articles eligible for quantitative data extraction that were used previously in the meta-analysis of Naranjo [14] (43 articles) were checked and transferred to the current database by one team member. In general, uncertainties occurring during the data extraction process were discussed continuously with at least one other person in the review team and data were only entered after an agreement was reached. From the 77 articles unique to the present database, a subset of 20 articles was double checked by a second member of the review team and uncertainties were discussed until an agreement was reached. This consistency check also comprised the critical appraisal of each record. As no relevant disagreements occurred, we decided to do no further consistency checks.

#### Qualitative data extraction

Relevant experiments not qualifying for quantitative data extraction (Fig. 1) were summarized in the form of detailed tables and discussed narratively (Additional file 3: Tables S3.1–S3.5). The following information was extracted: country and location of the study, transformation event and produced Bt protein(s), plot size and study years, comparison type including insecticide treatments, sampling method and number of samples per season, recorded taxa, response variables, number of replicates (N), observed effects, critical appraisal of the study, reference, and experimentID. Significant effects were reported based on the statistical analysis applied by the authors. For these tables, information was extracted on article level, i.e., each article represents one line in the respective tables.

### Study validity assessment

Critical appraisal of study validity is a key element in systematic reviews. All records in the database (Fig. 1 and Ref. [25]) were assessed for both external validity (the degree to which the study records are appropriate or applicable for answering the review question) and internal validity (risk of selection, performance, detection, and attrition bias). Labelling each record of the database with validity levels allows the exclusion of data with low external validity or data bearing high risk of bias, or the exploration of the influence of such studies on statistical analyses. To our knowledge, such a critical appraisal has not been done for previous meta-analyses or reviews of non-target field data of GM crops and so no predefined scheme of how to evaluate validity was available. Meissle et al. [9] translated the general guidance for critical appraisal provided by CEE to more specific criteria applicable to the present review question. For the final review, those criteria were further refined to 16 specific questions that were applied to each record in the database (data file 3 of Ref. [25], 10.5281/zenodo.6517033). Four answer categories were defined:

*Green:* Information provided by the authors for the criterion in question suggests a low risk of bias (influence of factors other than the Bt trait is unlikely) or high external validity (study design is appropriate to answer the review question).

*Yellow:* Information provided by the authors indicates that the criterion in question is suboptimal, but the risk of bias is limited and the external validity is moderate.

*Red:* Information provided by the authors indicates a major drawback in the criterion in question, which is likely to influence the measured outcomes by introducing factors other than the studied difference between Bt and non-Bt maize (risk of bias). This category also includes studies where the design is not appropriate to answer the review question (low external validity).

*Unavailable information:* If no information for the criterion in question was provided by the authors, the record was flagged “unreported”. For each criterion, we decided if unreported information is treated as either green, yellow, or red for the selection of records for meta-analyses, depending on the likelihood that the lack of information reduces validity. See data file 3 in Ref. [25] for details.

For each appraisal question, possible answers for each of the four validity categories were formulated as precisely as possible. Defining clear cut-off values was considered important to ensure transparency, consistency, and reproducibility of the judgement. The questions and cut-off values were developed within the review team and discussed with external experts during a workshop at the 13th ISBGMO meeting in Cape Town (South Africa) in November 2014 [27] and bilaterally with one expert on critical appraisal in systematic reviews and one expert on maize arthropods. The final list of questions and cut-off values are presented in Meissle et al. [25], data file 3).

The same critical appraisal criteria were also applied to the articles included in the summary tables (Additional file 3: Tables S3.1–S3.5). Critical issues (red and yellow flags) based on the defined criteria are provided for each article in the last column of the tables.

### Data analyses

The records in the database were analysed quantitatively by meta-analyses. We conducted meta-analyses on different levels whenever sufficient numbers of records were available.

#### Potential effect modifiers/reasons for heterogeneity

**Fig. 2:**
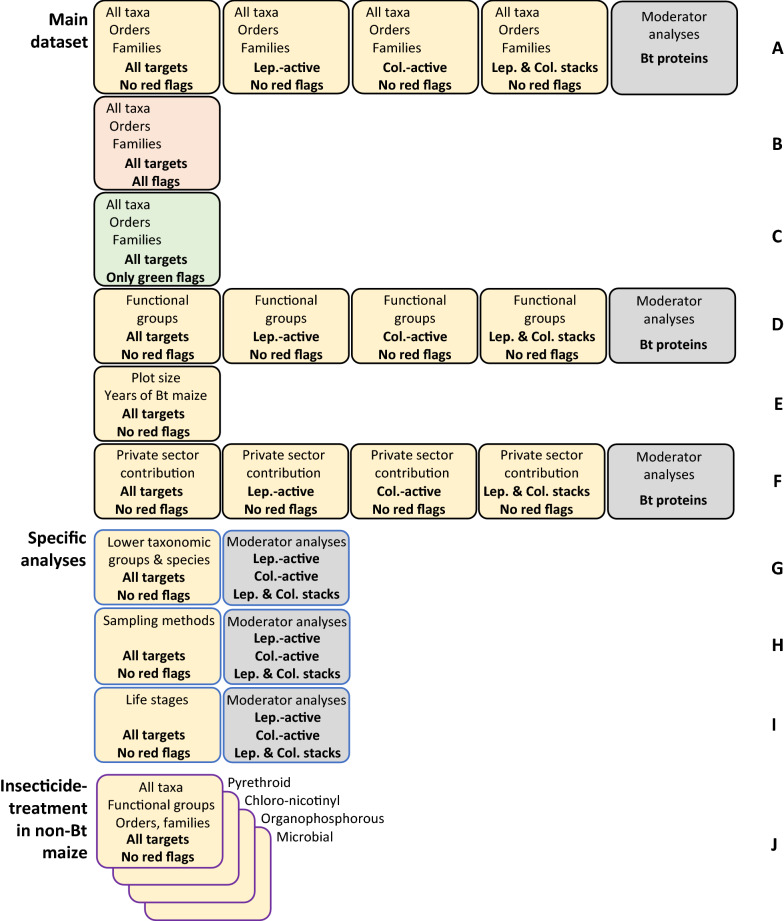
Overview of conducted meta-analyses. Lines **A**–**J** represent the sequence of analyses as presented in the paper. Lep. = Lepidoptera, Col. = Coleoptera. ”No red flags” indicates that records with any critical appraisal criterion flagged “high risk” were excluded. “Only green flags” indicates that only records with all critical appraisal criteria flagged “low risk” were included. “All flags” indicates that no records were excluded based on critical appraisal criteria

We considered the following variables for our main meta-analyses: different taxonomic levels, target orders of the Bt proteins, critical appraisal levels, functional groups, influence of private sector contribution, plot size, and years of Bt maize cultivation. In addition, specific analyses were conducted on taxonomic subgroups and species, individual sampling methods and juvenile life stages. Finally, analyses of insecticide treated non-Bt plots compared with untreated Bt plots were conducted (Fig. 2).

#### Data selection for meta-analysis

In many cases the database contains multiple records for the same invertebrate population from individual experiments. Because we wanted each meta-analysis to include each population only once, the most appropriate records were selected for each analysis. This ensured balanced analyses where each experiment was represented in a comparable way, independent from the way authors presented their data. For example, data from one experiment were available for lower taxonomic levels (e.g., species) and higher levels (species data aggregated to families, orders, or higher taxonomic units) or for individual life stages and all life stages combined. Depending on the conducted analyses, the aggregated records or the lower level records were selected. When the same invertebrate group had been recorded with different sampling methods within one experiment, we retained the record with the lowest coefficient of variation (CV), which indicates the highest precision. The mean coefficient of variation across both Bt and non-Bt treatments was estimated as CV = (SD_Bt_/Mean_Bt_ + SD_Control_/Mean_Control_)/2. In addition, we selected records without “red” fields in the critical appraisal unless only records with red fields were available (note: red-flagged records were excluded for most meta-analyses later on). If the CVs were similar, we retained records that used a more common method and/or the method that recorded more individuals. As an exception, for spiders (Araneae) we retained records for methods that collected ground-dwelling species as well as records for methods that collected plant-dwelling species, because ground and plant dwelling spiders are often represented by different families and species.

In some cases, experiments included multiple lines of Bt maize (multiple Bt treatments) that were compared to the same control maize line (one non-Bt treatment). In this case, all records (all comparisons) were selected for analysis. Similar to previous meta-analyses [10, 13, 14], we accepted this level of data-duplication.

Experiments where Bt and non-Bt maize received no or the same insecticide treatment (e.g., seed treatments) were analysed together and are refered to as “untreated”. We also analyzed data from studies comparing untreated Bt maize with insecticide-treated non-Bt maize. This group included studies where Bt maize was treated with insecticide 1 (e.g., a seed treatment) and non-Bt maize treated with insecticide 1 (seed treatment) + insecticide 2 (e.g., a pyrethroid spray). We refer to this group as “insecticide treated”.

The records that were selected for the different meta-analyses including the relevant variables for filtering and for moderator analyses are documented in Additional file 5. Note that the full database with all variables is available in data file 1 of Ref. [25] (https://doi.org/10.5061/dryad.3j9kd51jq).

#### Hedges’ d and meta-analysis models

Statistical analyses were conducted in R, version 4.0.5 [28], package metafor [29]. For each record, Hedges’ d and its variation was calculated (escalc function). The effect sizes with their 95% confidence intervals as well as heterogeneity (variation in outcomes between records) were estimated using random-effects models with restricted maximum-likelihood estimators for heterogeneity (rma function, method = “REML”). Effect sizes were considered significant if their confidence intervals did not include 0. Effect size estimates were structured such that negative values are associated with lower abundance, activity density, predation or parasitism in Bt maize fields or plots compared with non-Bt maize.

The R-code used to analyze the data is available in Additional file 6. The code is structured in the same way as the results are presented in the following. The supplied code has been applied to the spread sheet provided in Additional file 5, which is a simplified version of the full database (Ref. [25], data file 1) that contains the fields that are necessary for the statistical analyses. The field “Analyse_taxon” is a selection marker for the different taxonomic groups that were analysed individually. The term “Minor taxa” was applied to all records belonging to taxa that were not analysed individually. The field “Analyse1” was used to select records for the main analyses (described below), “Analyse4” for records with insecticide-treated non-Bt plots, “Analyse5” for taxonomic subgroups or species, “Analyse6” for specific analyses of different sampling methods and “Analyse7” for specific analyses of juvenile life stages.

As a minimum requirement, an individual meta-analysis was only conducted when at least 5 records were available for a given taxon and comparison type [30] and when those records derived from 3 different articles to ensure a certain level of data independence. Records of taxa not fulfilling these requirements, however, were included in higher level analyses. For example, for the family Curculionidae (Coleoptera), there were 6 records available from 2 articles. We thus did not conduct an individual meta-analysis for Curculionidae, but the 6 records were included in the higher level analysis for Coleoptera.

#### Main meta-analyses

*Different taxa and target orders:* From what is known about the mode of action, Bt proteins of certain classes act on certain taxonomic groups (target orders), i.e., Cry1, Cry2, and VIP class proteins on Lepidoptera and Cry3 class proteins on chrysomelid Coleoptera. We thus identified the taxonomy of the non-target species and target order of the Bt proteins as major variables for our meta-analyses.

For the main set of meta-analyses, records were selected on the taxonomic levels specified in Additional file 4: Nematoda, Myriapoda, Acarina, Oligochaeta, Collembola, Araneae, Opiliones, Dermaptera, Mecoptera, Neuroptera, Orthoptera, Psocoptera, Thysanoptera, Anthicidae, Cantharidae, Carabidae, Chrysomelidae, Cicindelidae, Coccinellidae, Elateridae, Lathrididae, Nitidulidae, Scarabaeidae, Staphylinidae, Chironomidae, Chloropidae, Dolichopodidae, Otitidae, Tachinidae, Syrphidae, Aphididae, Anthocoridae, Cicadellidae, Delphacidae, Geocoridae, Miridae, Nabidae, Pentatomidae, Braconidae, Formicidae, Ichneumonidae, Mymaridae, and Vespidae. Meta-analyses were conducted for each of those taxonomic groups whenever sufficient data were available (5 records from 3 articles).

Additional analyses were done for the taxonomic orders of Coleoptera, Diptera, Hemiptera, and Hymenoptera and for all taxa together. For those higher level analyses, no new database records were created, but the datasets for the taxonomic levels described above were used. For example, if one order contained 5 records for family 1 and 15 records for family 2, the order level analysis was conducted with all 20 records. Therefore, experiments that recorded data from several families had a higher weight in those analyses.

To account for the different mode of action of Bt proteins, we conducted separate statistical analysis with (1) all Bt proteins independent of target order; (2) only Lepidoptera-active proteins (Cry1, Cry2, and VIP); (3) only Coleoptera-active proteins (Cry3); and (4) only stacks containing Lepidoptera-active and Coleoptera-active proteins (Fig. 2, line A).

Records with any “red” field in the critical appraisal were excluded because they have the potential to introduce bias or they have low relevance for the review question. We also conducted moderator analyses (analyses with a grouping factor) for “Bt-protein”, and reported the results whenever at least 5 records from 3 articles for a given Bt protein were available.

*Different taxa and critical appraisal levels:* With the same dataset, analyses on the different taxonomic levels specified above were repeated for 1) all records, no records excluded based on critical appraisal (Fig. 2, line B), and 2) only records with green flags for all critical appraisal questions, i.e., studies with low risk of bias and high relevance for the review question (Fig. 2, line C). For those analyses, records with all Bt proteins were used.

*Functional groups:* Potential effects of Bt maize on functional groups (decomposers, herbivores, omnivores, parasitoids, predators) were analysed for (1) all Bt proteins, (2) Lepidoptera-active only, (3) Coleoptera-active only, and (4) Lepidoptera- and Coleoptera-active stacks. Records with “red” fields were excluded (Fig. 2, line D). Moderator analyses with the factor “Bt protein” also were conducted. Furthermore, additional analyses were performed for the response variables “predation rate” and “parasitism rate” for the functional groups of predators and parasitoids, respectively. Those rates measure the biological control function of natural enemies and are therefore of particular interest.

*Influence of plot size and years of Bt maize:* Studies conducted in larger plots might show stronger effects because of reduced plot-to-plot movements of invertebrates and because of reduced edge effects. Therefore, meta-regressions were conducted using plot size against the absolute value of effect size. We further hypothesized that multiple years of Bt-maize cultivation on the same plots or fields may result in higher effect sizes than first year Bt maize cultivation. Therefore, meta-regressions were conducted with years of Bt maize against the absolute value of effect size. Analyses on plot size and years of Bt maize were done for each functional group with records from all Bt proteins. Records with “red” fields were excluded (Fig. 2, line E).

*Influence of private sector contribution:* We analysed if records from articles with authors from private sector product developers (biotechnology companies) or where funding was provided by such members of the private sector, resulted in different outomes than studies authored exclusively by researchers from public (including governmental) institutions and without declared private sector funding. Analyses on private sector contribution were done for all taxa combined for (1) all Bt proteins, (2) Lepidoptera-active only, (3) Coleoptera-active only, and (4) Lepidoptera- and Coleoptera-active stacks. Records with “red” fields were excluded (Fig. 2, line F). Moderator analyses with the factor “Bt protein” were conducted.

*Funnel plot:* A funnel plot was produced by plotting the effect size (Hedges’ d) on the y-axis and the variation of effect size on the x-axis. Egger’s test was used to determine if the distribution of data is unsymmetrical, indicating potential publication bias [29]. This plot included all records except the ones with “red” fields.

#### Specific analyses for taxonomic subgroups, sampling methods, and juvenile life stages

Specific analyses on lower taxonomic levels were conducted for 31 species and the following taxonomic groups: Oribatidae and Mesostigmata (Acarina, order-level), Chilopoda (Myriapoda, class-level), *Diabrotica* spp. (Coleoptera: Chrysomelidae), *Scymnus* spp. (Coleoptera: Coccinellidae) (genus-level), Chrysopidae (Neuroptera), Hemerobiidae (Neuroptera) (family-level), herbivorous and predatory Thysanoptera, and parasitoids in Lepidoptera-active maize excluding those of target (*O. nubilalis*) larvae, such as Braconidae and Tachinidae (Fig. 2, line G).

Furthermore, specific analyses were performed for different sampling methods, e.g., pitfall traps, visual counts, sweep nets, etc. (Fig. 2, line H), and for juvenile life stages, e.g., eggs, larvae & pupae (Fig. 2, line I). Sampling methods and life stages were analysed for the same taxonomic groups used for the main analyses described above.

The specific analyses were performed with all Bt proteins combined and results of moderator analyses with the factor Bt maize target order (Lepidoptera-active only, Coleoptera-active only, and Lepidoptera- and Coleoptera-active stacks) were added whenever at least 5 records were available from 3 articles for a given target order. Records with “red” fields in the critical appraisal were excluded.

#### Insecticide treatments in non-Bt maize

Insecticide treated non-Bt plots were compared to untreated Bt plots (Fig. 2, line J) for the different taxonomic levels and for functional groups. These analyses were done for all Bt proteins combined, while records with “red” fields were excluded. Separate analyses were conducted for the different insecticide classes: pyrethroid, chloro-nicotinyl, organophosphorous, and microbial. However, almost no data were available for the latter two groups.

#### Robustness of significant effect sizes

For significant effect sizes (95% confidence intervals do not include 0) in all previously described analyses (except moderator analyses), the robustness of the effect was further evaluated by calculating the fail safe number according to Rosenberg [31], i.e., the number of studies with effect size 0 that need to be added to the analysis to turn the outcome non-significant. According to Rosenberg [31], for a robust analysis, the fail safe number should be larger than 5n + 10. In addition, we repeated each significant analysis multiple times while leaving one record out at the time to identify individual records with high influence on the analysis result [29]. For example, if a significant meta-analysis included 20 records, the analysis was repeated 20 times leaving a different record out each time. This procedure was repeated at the experiment and article levels to see which individual experiments and articles might have had a greater influence on the result. Fail safe numbers, the values for 5n + 10 and the results of the “leave one out” analyses for records, experiments, and articles are provided in Additional file 7: Table S7.14.

## Review findings

### Review descriptive statistics

The literature searching in 12 bibliographic databases resulted in a total of 12,967 references after the automatic deduplication steps. From the searches on specialist webpages, 110 additional references were identified. These include various application dossiers and monitoring reports of MON810 retrieved from the EFSA register of questions. According to Regulation (EC) No. 1049/2001, we requested read-access for those EFSA documents [32]. For 5 regulatory reports, we approached the study owners for approval to use the data, which was granted in 3 cases. Finally, 19 more references were obtained through other sources (e.g., identified in reviews or received from colleagues).

**Fig. 3:**
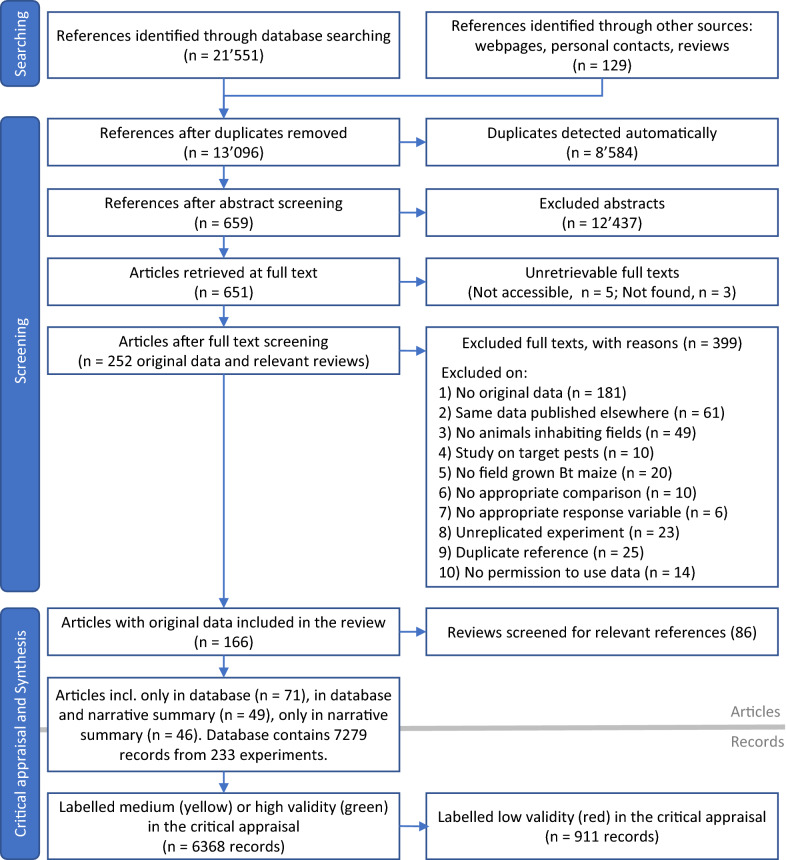
ROSES flow diagram for systematic reviews after Haddaway et al. [33]. Overview of the number of references retrieved from literature search, articles retained after applying eligibility criteria, and records in the database classified according to critical appraisal criteria

At the end of the literature retrieval, screening, and sorting process, the Endnote library consisted of 659 references (Fig. 3, Additional file 2). For 8 references no full texts could be obtained and 399 references were excluded based on the criteria listed above (Additional file 2, Fig. 3). The screening of 86 reviews on environmental or non-target effects of GM crops, Bt crops, or Bt maize revealed 7 additional references. Finally, 166 articles were used for this systematic review, either for quantitative data extraction only (71), for the narrative summary tables only (46), or for both (49).

The consistency checks at title/abstract level for the first literature search revealed 5 references that were excluded by the main reviewer, but not by the second reviewer. When both reviewers checked those references again at full-text, none of them turned out to be relevant for this systematic review. For the references obtained in the updates in January and August 2019, thirteen were excluded by the main reviewer, but included by the second reviewer. After closer examination of the full texts by both reviewers, two references were identified as useful reviews to be checked for additional references and one reference contained data for the narrative summary tables. Only one relevant study with original data was authored by a member of the review team and this study was not evaluated by this team member. Consistency checks at full text level and after data extraction and critical appraisal did not reveal major differences among members of the review team.

### Narrative synthesis: summary of data not contained in the database

Detailed tables with all extracted information and critical appraisal for studies relevant to the review question, but not included in the quantitative database can be found in Additional file 3 (Tables S3.1–S3.5). This also includes a narrative summary of the data based on the findings reported by the authors.

### Narrative synthesis: characterization of the database

**Fig. 4:**
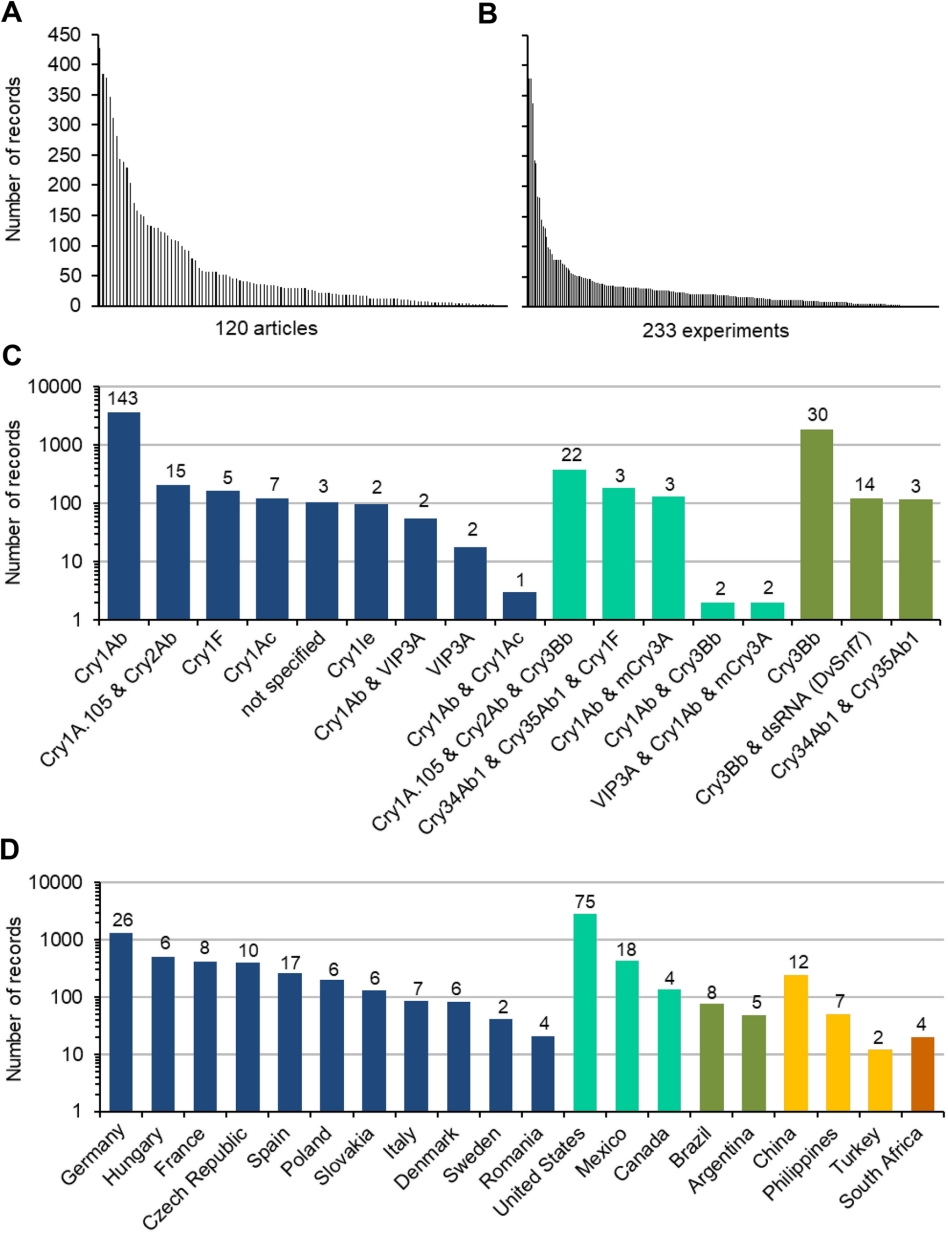
Distribution of non-target invertebrate records in the database (articles, experiments, Bt proteins, countries). **A** records per article (each bar represents one article), **B** records per experiment, **C** Bt protein produced by the studied Bt-maize line grouped by target order (Lepidoptera-active in blue, Coleoptera-active in green, Lepidoptera- and Coleoptera-active in turquoise), and **D** countries where the experiments were conducted (different colours indicate continents). Within panels (**C**) and (**D**), the number of experiments associated with each bar is given. Note the log-scale

Altogether, the final database contains 7279 invertebrate records (lines in the database), extracted from 120 articles. The data in the database were derived from 233 experiments (defined as field or plot setup in one location and one year). The number of records extracted from each article varied from 1 to 428 with a median of 30 records (Fig. 4A), and from each experiment from 1 to 379 with a median of 18 records (Fig. 4B).

The most studied Bt proteins were Lepidoptera-active (4505 records, 178 experiments) led by Cry1Ab (Fig. 4C). Additional Lepidoptera-active proteins were Cry1A.105, Cry1Ac, Cry1F, Cry1Ie, Cry2Ab, and VIP3A. In 263 records (18 experiments), multiple Lepidoptera-active Bt proteins were pyramided. Coleoptera-active Bt proteins (2078 records, 47 experiments) were mainly represented by Cry3Bb. Coleoptera-active proteins also included mCry3A and Cry34Ab1/Cry35Ab1. In 696 records (30 experiments), Lepidoptera-active Bt proteins were stacked with Coleoptera-active ones.

Almost half of the records (2839) represented 75 experiments from the USA (Fig. 4D). The majority of the remaining records (3434) represented European experiments (98) with most data generated in Germany (1291 records, 26 experiments). Less than 450 records from 38 experiments were available from South America, Asia, and Africa combined.

**Fig. 5:**
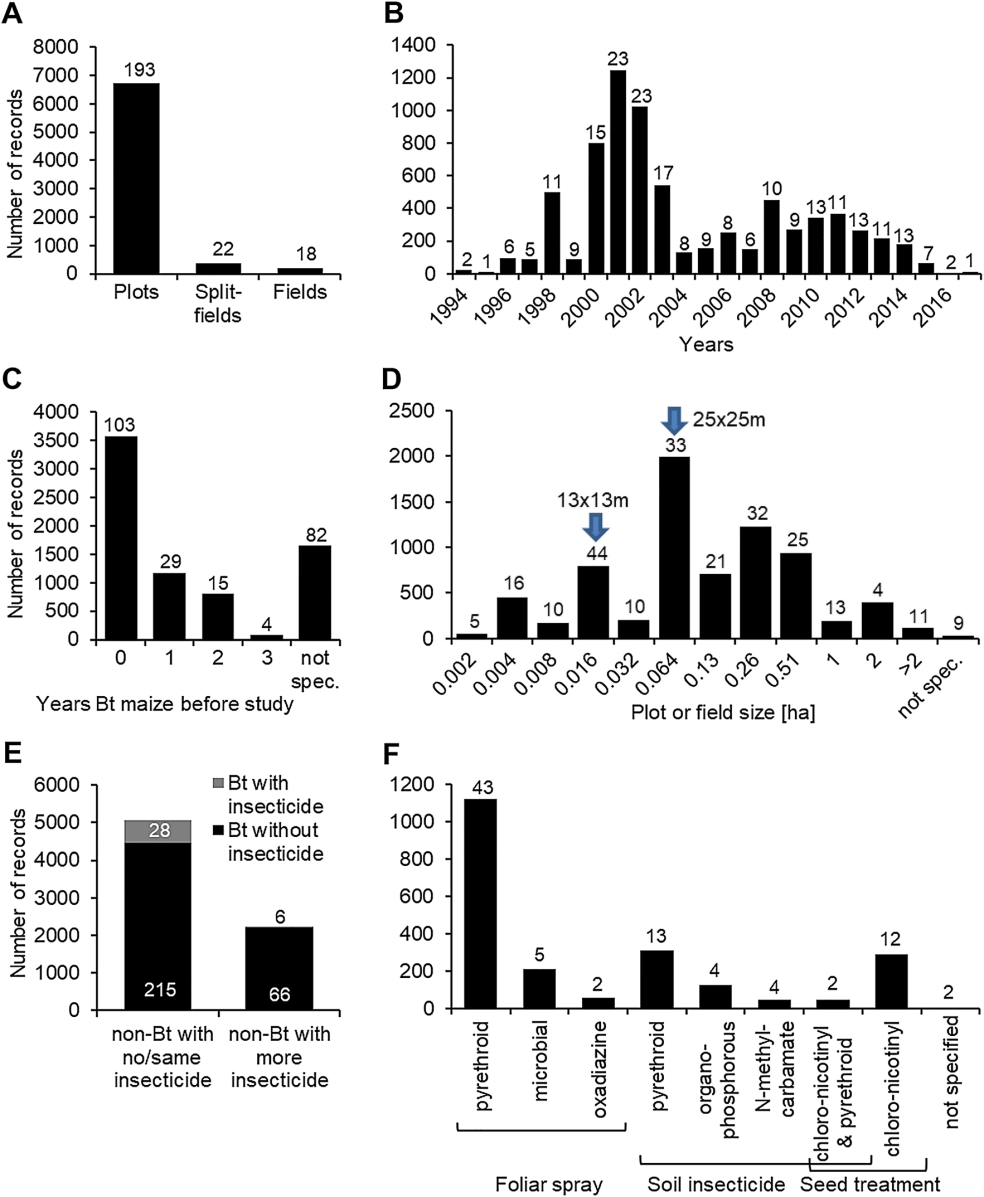
Distribution of non-target invertebrate records in the database (experimental design, planting year, years Bt maize, plot/field size, comparison type, insecticides), **A** experimental design (use of plot designs within a field, split-field designs, or field-to-field comparisons), **B** year when the field experiment was conducted, **C** number of years in which Bt maize was cultivated on the same plots as Bt maize in the studied year (not spec.: authors did not state previous crop), **D** plot size of the replicated units (numbers represent maximal plot sizes in the respective category, e.g., 0.004 means that plots were larger than 0.002 and maximal 0.004 ha), **E** type of comparison between Bt maize and non-Bt maize (e.g., untreated Bt maize versus insecticide treated non-Bt maize), **F** insecticide classes and application mechanism in those cases where non-Bt maize was treated with insecticides differently to Bt maize. Within the panels, the number of experiments associated with each bar is given

The experimental design for 92% of the records (193 experiments) consisted of replicated plots in one or two fields (Fig. 5A). Split-field designs and designs where different fields served as replicates represented the remaining 5% and 3% of the records (22 and 18 experiments), respectively. Data were available from field studies conducted from 1994 to 2017 (Fig. 5B). Most data, however, were generated between 2000 and 2003. Most invertebrates were collected in plots that were not planted to Bt maize in the year(s) before the experiment (49% of the records, 103 experiments) (Fig. 5C). In 16% of the records, Bt maize was grown for 1 year before the sampling season (29 experiments), 11% for 2 years (15), and 1% for 3 years (4). In 23% of the records (82 experiments), however, the authors did not specify if Bt maize was grown in the plots before the start of the experiment. Plot sizes ranged from 0.001 ha to 27 ha (Fig. 5D). Almost 80% of the records derived from plots larger than 0.016 ha (e.g., 13 × 13 m plots) and almost 50% from plots greater than 0.06 ha (e.g., 25 × 25 m plots).

In 61% of the records in the database (215 experiments), invertebrates recorded in Bt maize without any insecticide treatment were compared with those recorded in non-Bt maize without any treatment (Fig. 5E, black bar left). In 8% of the records (28 experiments), Bt and non-Bt maize received the same insecticide treatment (e.g., seed treatments or treatments against pests other than the targets of the Bt maize) (grey top bar left). For simplicity, those records were included in the “untreated” group for meta-analyses. In one third of the records (66 experiments), invertebrates recorded in untreated Bt maize were compared to those from insecticide-treated non-Bt maize (black bar right). Ten records (0.1%, 6 experiments) were available from experiments where both Bt and non-Bt plots received the same insecticides (seed treatments or foliar sprays), but non-Bt maize received additional insecticide treatments (grey top bar right). Those records were subsequently included in the “Bt untreated / non-Bt treated” group. Insecticides were either applied to the plants, the soil, or the seeds (Fig. 5F). Foliar sprays were mainly conducted using pyrethroids or microbial Bt or spinosad formulations. Some data also are available for oxadiazine. Soil insecticides are represented by pyrethroids, organophosphates, and carbamates. Finally, seeds were mostly coated with chloro-nicotinyl insecticides. In 6 records (2 experiments), insecticide treatment was mentioned, but not specified further.

**Fig. 6:**
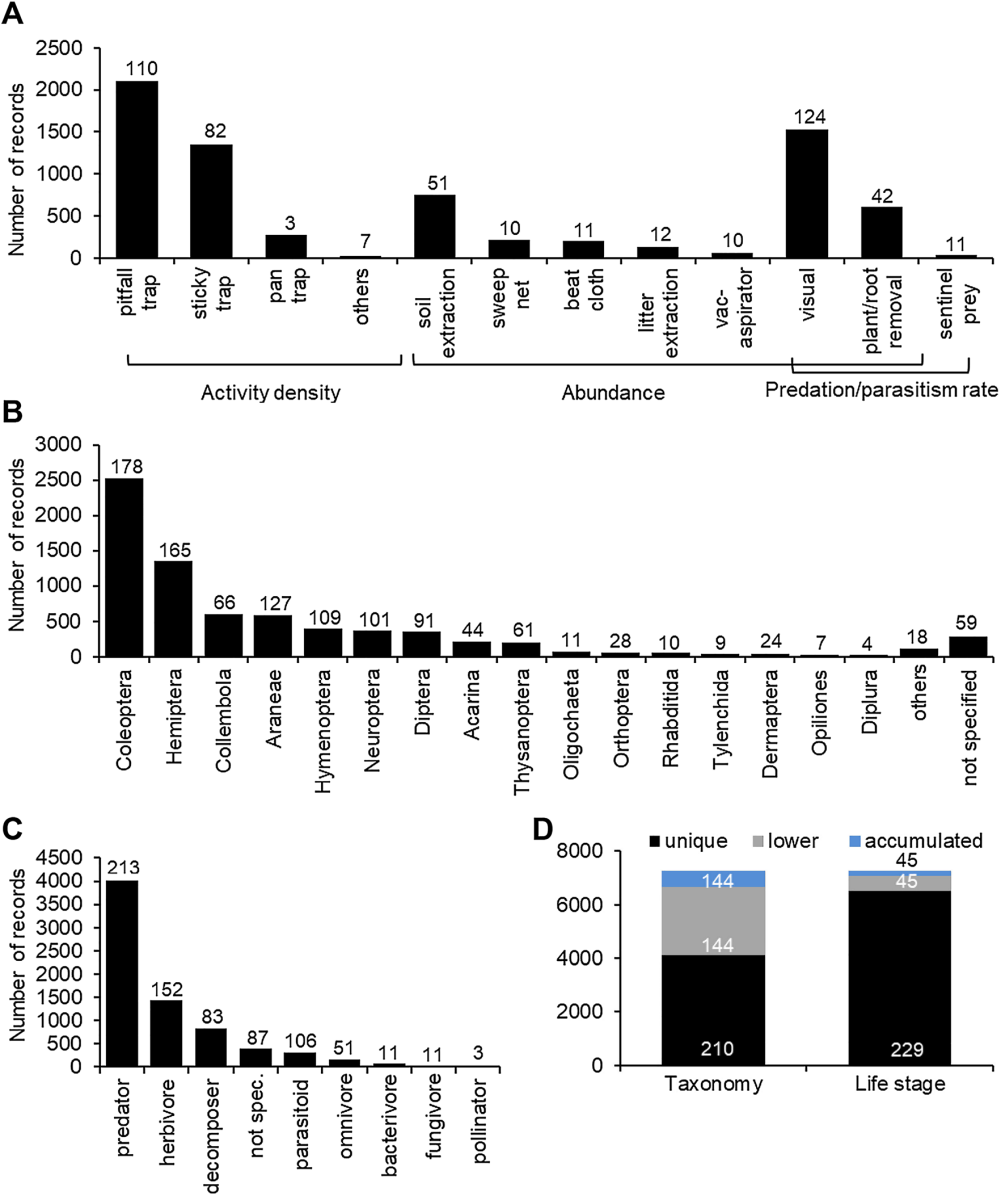
Distribution of non-target invertebrate records in the database (sampling method, taxonomic order, functional group, record type). **A** Sampling method used to record invertebrates and the corresponding response variables, **B** taxonomic order of the recorded invertebrates, **C** functional group (not spec: no functional group could be assigned to the taxon), and **D** number of records that represent aggregated data (accumulated) for taxonomy or life stage, number of records of lower taxonomic units or individual life stages (lower) that have been used to generate the aggregated records, and number of records for taxa and life stages that were not aggregated (unique). Within the panels, the number of experiments associated with each bar is given

Invertebrates were collected with a range of different sampling methods (Fig. 6A). Activity density was mainly recorded with pitfall traps, sticky traps, and pan traps. In addition, Malaise traps, baited traps, and stem eclectors were used (“others” in Fig. 6A). Abundance was recorded with visual observations (either counting in the field or removing specimens), plant or root removals followed by sorting in the laboratory, soil extraction, sweep nets, beat cloths, litter extractions, and aspirators. Parasitism and predation rates were determined with visual observations, plant removal, or sentinel prey (egg cards or artificial prey). The response variables used in most studies were activity density (53% of records, 151 experiments) and abundance (46%, 176 experiments). Parasitism and predation rates were only reported in a total of 54 records (0.8%, 19 experiments).

Various invertebrate orders were collected in Bt and non-Bt maize (Fig. 6B). The most studied order was Coleoptera (35% of all records, 178 experiments). The families of Coccinellidae and Carabidae together represented 64% of all Coleoptera records, followed by Staphylinidae, Chrysomelidae, Nitidulidae Elateridae, and 16 other families. The second most studied order was Hemiptera (19% of all records, 165 experiments). Hemiptera were represented by Anthocoridae, Aphididae, Cicadellidae, Nabidae, Miridae, and 8 other families.

Data on species level are available for 280 species in 2466 records and 157 experiments. The most studied single species is the predatory flower bug *Orius insidiosus* (Hem: Anthocoridae) with 258 records, followed by the ladybird beetles *Coleomegilla maculata* (Col: Coccinellidae) (93 records) and *Harmonia axyridis* (Col.: Coccinellidae) (76 records), the lacewing *Chrysoperla carnea* (Neu: Chrysopidae) (63 records), the ground beetle *Pterostichus melanarius* (Col: Carabidae) (56 records), the aphid *Rhopalosiphum padi* (Hem: Aphididae) (52 records) and the parasitoid *Macrocentrus cingulum* (Hym: Braconidae) (47 records).

When analysing functional groups, predators dominated with 55% of all records (213 experiments) (Fig. 6C). The functional groups of herbivores, decomposers, parasitoids, and omnivores represented 20%, 12%, 4.4%, and 2.3% of the records (152, 83, 106, 51 experiments), respectively. Some nematode records were assigned to the functional group of bacterivores or fungivores (together 1.4%, 11 experiments). Only 7 records for pollinators were available (0.1%, 3 experiments). Records of higher taxonomic units that comprise species with different modes of feeding, were assigned “not specified”. (5.6%, 87 experiments).

Of all records in the database, 35% (144 experiments) represent data of lower taxonomic groups (e.g., species), which also have been used to generate records for aggregated, higher taxonomic units (e.g., families or orders) (Fig. 6D). Those aggregated higher taxa represent 8% of the total records, while 57% of the records are unique (data of the taxon not used for aggregated records, 210 experiments). Similarly, 8% of the records (45 experiments) represent individual life stages, which also have been used for additional records with aggregated life stages (2%), while 90% of the records are unique (life stage only once in the database, 229 experiments).

### Narrative synthesis: study validity assessment

**Fig. 7:**
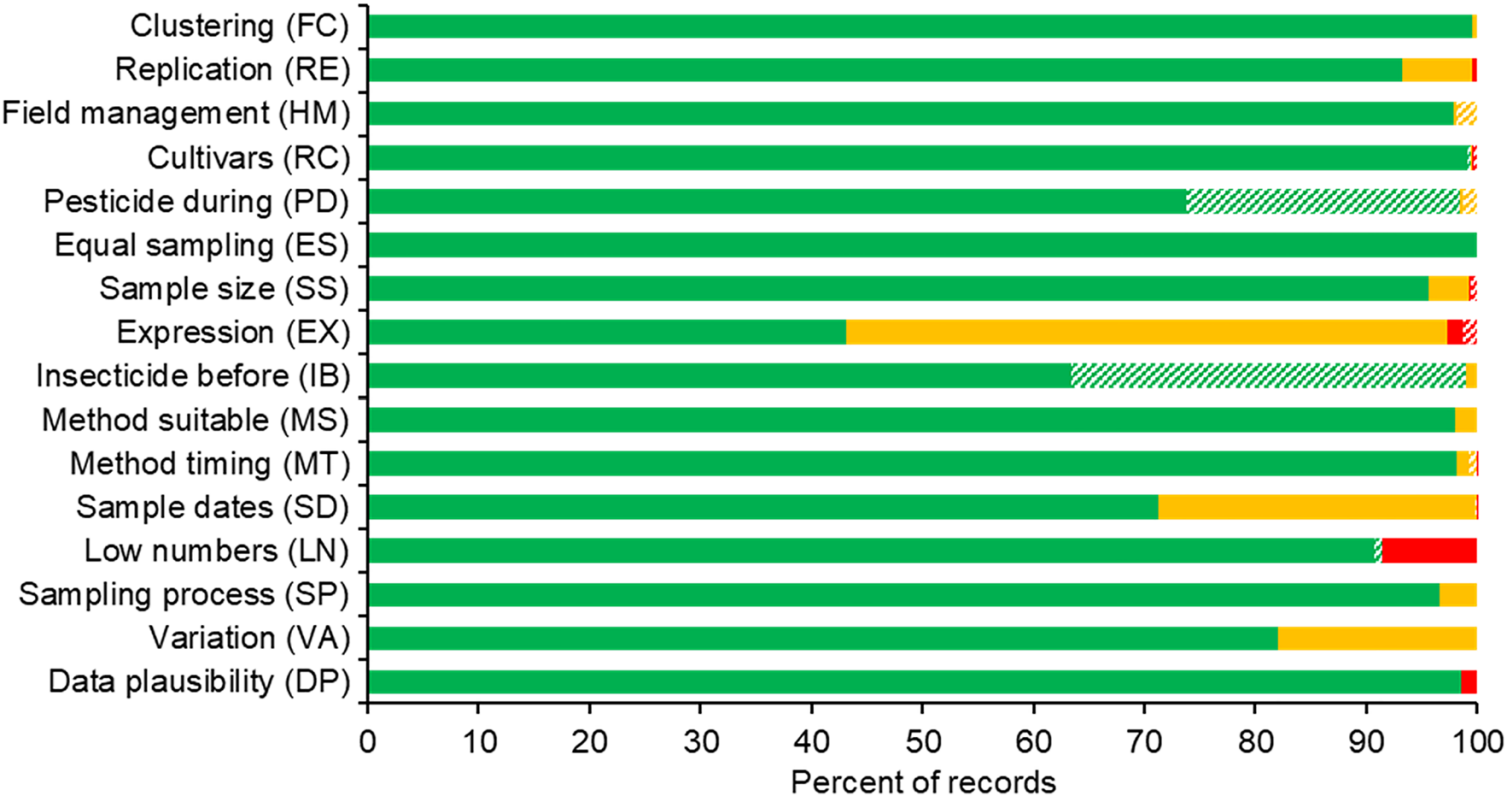
Critical appraisal for the 7279 records in the database. Red bars represent records with low, yellow bars with medium, and green bars with high validity. Hatched bars indicate that no information was available. Missing information for each criterion was treated as either red, yellow, or green for meta-analyses. See Ref. [25] for details

The design of the studies consisted in most cases of plots, which were randomized or systematically distributed across the field without evidence of clustering (FC, 2-letter codes used in Fig. 7 and in Ref. [25]). Usually, three or more plots per treatment or four or more separated fields were deployed (RE). There was no indication that Bt and non-Bt fields had a different history of management before the study (HM). For plot and split-field designs, the history of management was assumed to be equal as plots were installed within the same field. Authors generally reported that they used near-isolines or nearest comparators for Bt and non-Bt maize (RC). There also was no indication that Bt and non-Bt plots received different pesticides (e.g., fungicides, herbicides) during the study, except for insecticide treatments that were part of the experimental design (PD). For 25% of records, however, no information on pesticide treatments during the study was provided. For meta-analyses, we assumed that no pesticides were applied when authors did not mention such treatments or that Bt and non-Bt plots received similar treatments. Bt and non-Bt plots were sampled with the same methodology (ES), and the sample size in Bt and non-Bt plots or fields was in most cases similar (SS).

Regarding external validity of a record, which means that the data are relevant for the review question, expression of the Bt protein was often not confirmed by the authors in the field experiments where invertebrates were collected (EX). If commercial seeds were used, however, sufficient expression was assumed (green label). If a commercialized transformation event was used (e.g., MON810), but no confirmation of expression was provided, we treated the record as “yellow”. This was the case for 54% of the records. Red labels were applied to non-commercialized transformation events when the authors did not provide evidence for Bt protein expression. In most cases no insecticides were applied before the start of the study (IB). If no information was provided (36% of the records, hatched area in Fig. 7), we assumed that no insecticides were applied and treated the record for this criterion as “green” for meta-analyses.

Generally, invertebrates were collected with common and appropriate methods (MS) and also the time of collections was suitable to cover the population over the season (MT). In more than 70% of the records, ≥ four samples were performed over the season (SD). The remaining 30% of the records, labelled “yellow”, were based on ≤ three samplings. The criterion LN tried to capture low sampling success for the respective taxa. If a low number of specimens was collected in all plots combined over the whole season, it is likely that the population of this taxon is not described adequately. In 9% of the records less than 20 individuals were collected over the whole season in all plots (LN).

The last 3 criteria of the critical appraisal cover uncertainty of the data. The sampling procedure was sufficiently described in most cases (SP). One problem that occurred when extracting data was that authors often did not specify how they calculated SDs or SEs. SEs were sometimes based on the number of traps or sampled plants rather than the number of true replicates (plots or fields) (VA). Reported values were assigned an increased level of uncertainty (yellow) when (1) it was known that the authors based their calculations not on the true number of replicates, but also (2) when the seasonal SD had to be estimated from individual sampling dates, or (3) when error bars and/or the calculation method were not properly explained. Approximately 18% of the records showed such uncertainty in the calculation of variation. Finally, a few records showed other issues that indicated that the data might not be plausible (e.g., very low SEs for all taxa) (DP).

In summary, the total number of records in the database labelled with “red” in at least one critical appraisal criterion, thus indicating low internal or external validity, was 911 of 7279 records (12.5%). Most red labels were assigned because of low numbers of collected individuals. In the yellow category (moderate issues in validity), the main problems were the lack of reported Bt protein expression, a low number of sampling dates over the season, and uncertainty in the calculation of variation.

### Data synthesis

#### Different taxa and target orders

**Fig. 8:**
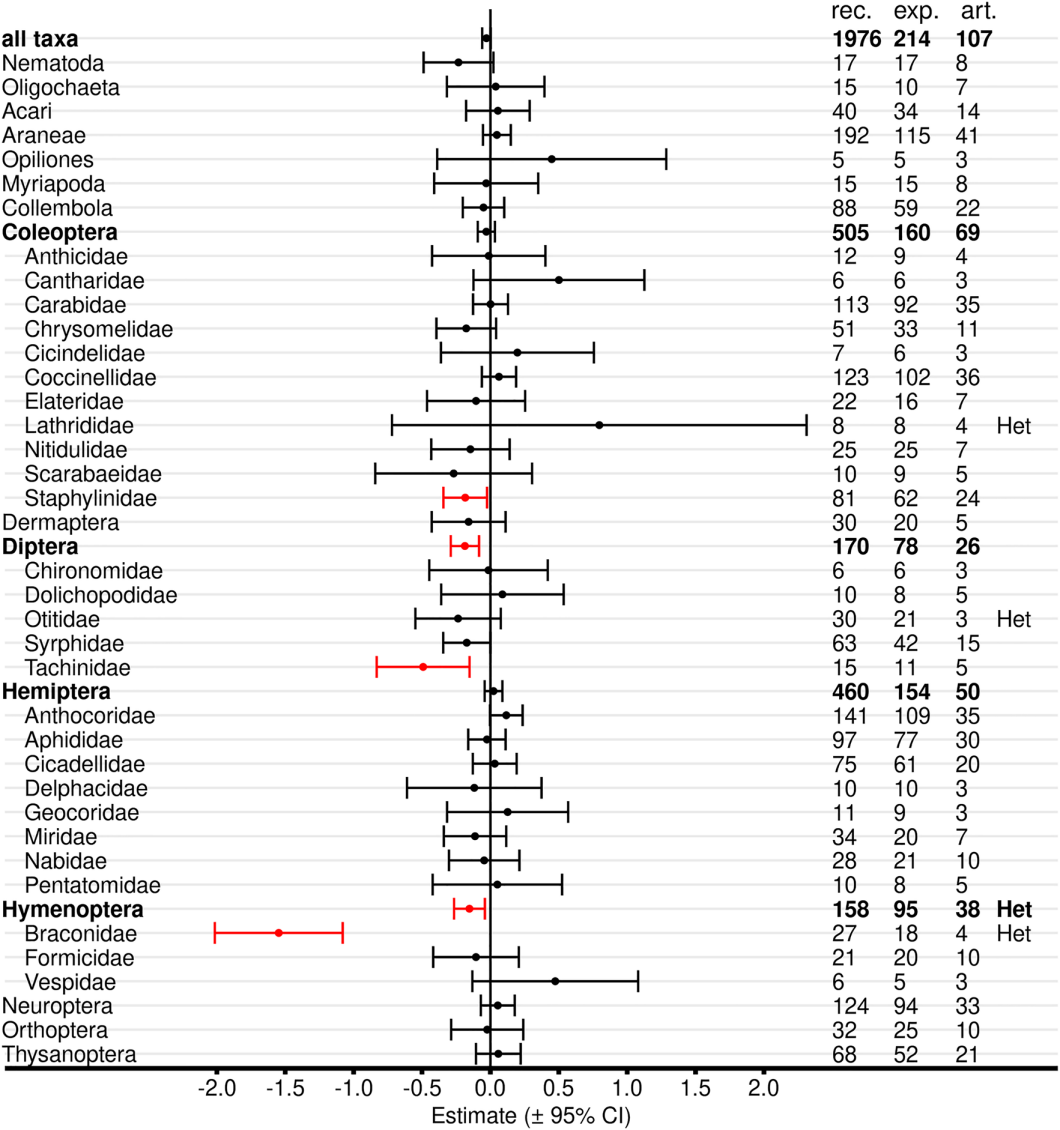
Meta-analyses on different taxonomic levels for untreated Bt and non-Bt maize (all Bt proteins, red-flagged records excluded). For each taxon, the effect size estimate and the 95% confidence interval is given (negative effect sizes indicate lower populations in Bt compared with non-Bt maize and vice versa). Significant intervals (red) do not include 0. On the right side is the number of records (rec.), experiments (exp.), and articles (art.) included in each analysis. Het indicates significant (*P* < 0.05) heterogeneity. See Additional file 7: Table S7.1 for details

Meta-analyses with all Bt proteins, but without red flagged records in the critical appraisal revealed reduced abundance, activity density, or predation/parasitisation rates in untreated Bt maize compared with untreated non-Bt maize for Staphylinidae (Coleoptera), Tachinidae (Diptera), and Braconidae (Hymenoptera) (Fig. 8, Additional file 7: Table S7.1). Syrphidae showed a negative effect size with an upper boundary of exact zero. Braconidae also revealed significant data heterogeneity. Effect sizes for order-level analyses in Diptera and Hymenoptera also were significant and Hymenoptera showed significant heterogeneity. No differences between Bt and non-Bt maize were evident for the other 35 analyzed taxa as well as for the higher level analyses of Coleoptera, Hemiptera, and all taxa. Significant heterogeneity was observed for Lathrididae (Coleoptera) and Otitidae (Diptera), but both groups were represented by records from only 4 and 3 articles (8 and 21 experiments), respectively.

The main analyses with 1976 selected records labelled as comparing “untreated” Bt and non-Bt maize included 82 records (4%) from studies where Bt and non-Bt plots were treated with the same insecticide: 60 records with seed treatment (26 chloro-nicotinyl, 7 chloro-nicotinyl or organophosphorous, 1 organophosphorous, 26 not specified), 19 with foliar spray (carbamate), and 3 with soil insecticides (2 carbamate, 1 organophosphorous). Because of the low number of records and because we did not see a clear hypothesis for interaction of those insecticides with Bt proteins, we included them in the “untreated” category for simplicity.

Altogether, 384 of the 1976 records from 5 articles (19.6%) represent experiments where the data from the control plots were used twice (332 records) or four times (52 records) because two or four Bt maize cultivars had the same control cultivar. This situation is analogous to the use of Dunnett’s test, where each treatment is compared with the same control, but without correction for multiple tests. Thus, our results here might be slightly less conservative.

**Fig. 9:**
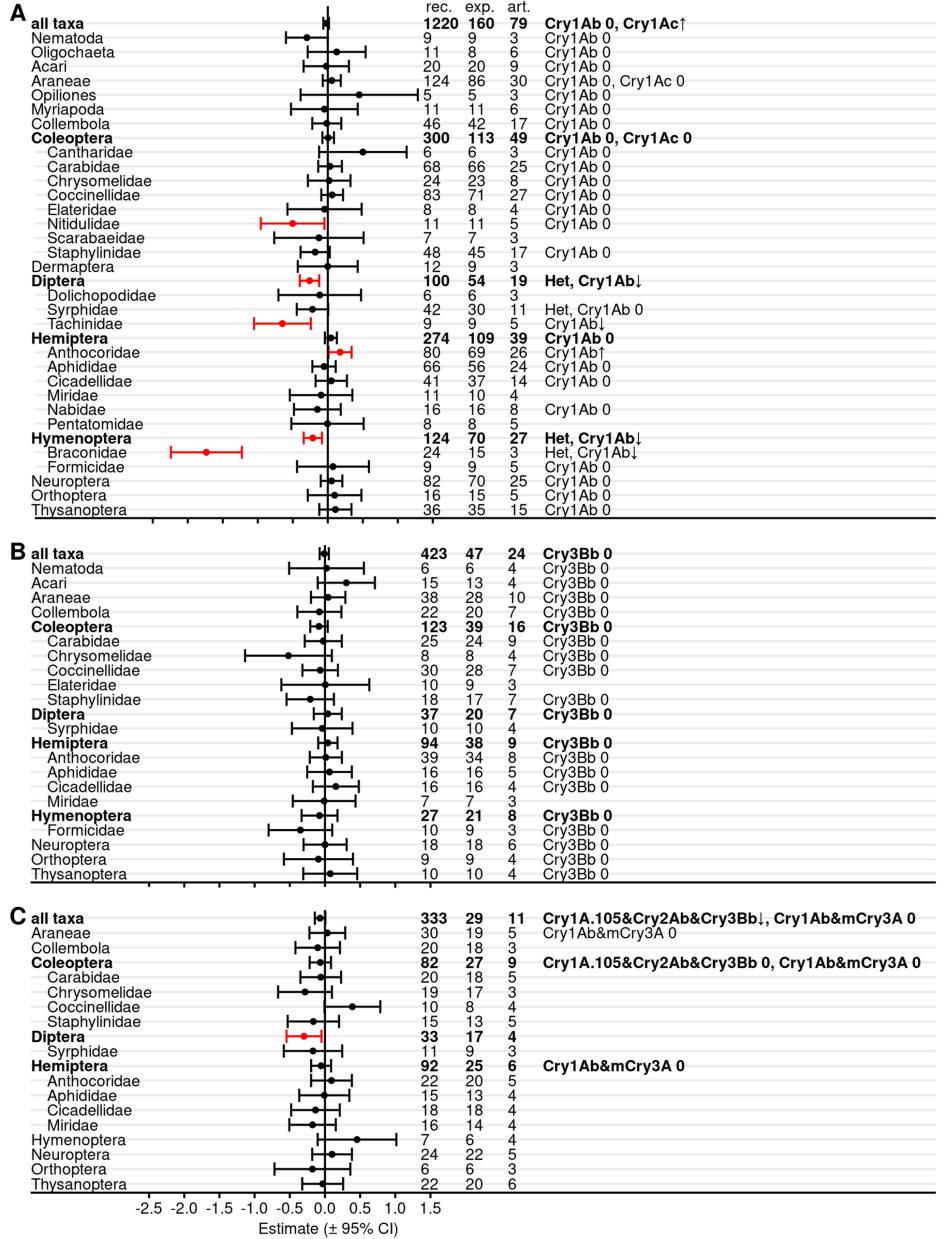
Meta-analyses on different taxonomic levels for untreated Bt and non-Bt maize (by target order). Only Lepidoptera-active (**A**), Coleoptera-active (**B**), or stacked Lepidoptera- and Coleoptera-active (**C**) Bt proteins were included. Records with any red flag in the critical appraisal were excluded. For each taxon, the effect size estimate and the 95% confidence interval is given (negative effect sizes indicate lower populations in Bt compared with non-Bt maize and vice versa). Significant intervals (red) do not include 0. On the right side is the number of records (rec.), experiments (exp.), and articles (art.) included in each analysis. Het indicates significant (*P* < 0.05) heterogeneity. See Additional file 7: Table S7.2 for details. Results of moderator analyses for individual Bt proteins (Additional file 7: Table S7.3) are indicated with arrows. ↑: higher values in Bt compared with non-Bt treatment (positive effect size), ↓: lower values (negative effect size), 0: no effect

When the analyses were repeated for only Lepidoptera-active Bt proteins, lower populations in Bt plots or fields (significant negative effect size) were observed for Nitidulidae, Tachinidae, and Braconidae, while higher populations in Bt plots were observed for Anthocoridae (positive effect size) (Fig. 9A, Additional file 7: Table S7.2). On higher taxonomic level, Diptera and Hymenoptera showed negative effect sizes. Data heterogeneity was present for Diptera, Syrphidae, Hymenoptera, and Braconidae. Moderator analyses, which examined the effects of different Bt proteins within a target group, revealed that the negative effects on Tachinidae (and Diptera) and on Braconidae (and Hymenoptera) were mainly associated with effects of Cry1Ab-producing maize. Furthermore, the moderator analyses showed a positive effect of Cry1Ab maize on Anthocoridae and of Cry1Ac maize on all taxa combined (Fig. 9A, Additional file 7: Table S7.3). Analyses with maize producing Coleoptera-active Bt proteins revealed no effects on any taxon (Fig. 9B, Additional file 7: Tables S7.2, S7.3). Stacked Lepidoptera and Coleoptera-active maize revealed a negative effect size for Diptera (Fig. 9C, Additional file 7: Table S7.2) and a negative effect size for maize producing Cry1A.105 & Cry2Ab & Cry3Bb when all taxa were combined (Additional file 7: Table S7.3).

#### Different taxa and critical appraisal levels

**Fig. 10:**
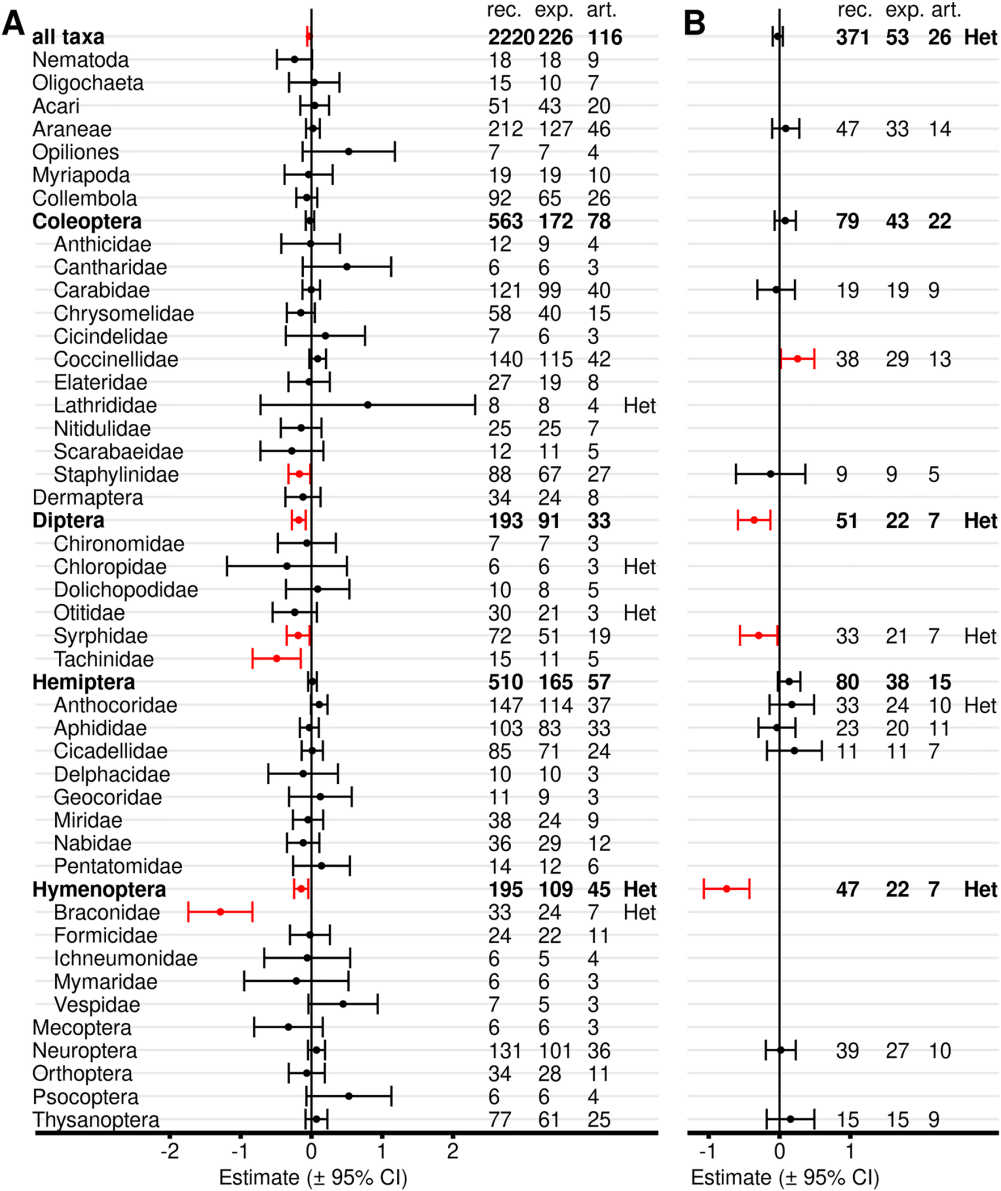
Meta-analyses on different taxonomic levels for untreated Bt and non-Bt maize (by critical appraisal level). Either all records (**A**), or only records with all critical appraisal criteria flagged “green” were included (**B**). All Bt proteins were included. For each taxon, the effect size estimate and the 95% confidence interval is given (negative effect sizes indicate lower populations in Bt compared with non-Bt maize and vice versa). Significant intervals (red) do not include 0. On the right side is the number of records (rec.), experiments (exp.), and articles (art.) included in each analysis. Het indicates significant (*P* < 0.05) heterogeneity. For details see Additional file 7: Table S7.4

Critical appraisal is an important element in systematic reviews. By default, we excluded records with any “red” flagged critical appraisal criteria for meta-analyses to avoid potential bias. However, we also repeated the main analyses (all Bt proteins included) for all records without any exclusions based on critical appraisal (Fig. 10A, Additional file 7: Table S7.4). Inclusion of the red-flagged records resulted in a similar outcome of the analyses with a negative effect size (lower populations in Bt maize) for Staphylinidae, Syrphidae, Tachinidae, and Braconidae, and consequently for the higher taxonomic orders Diptera and Hymenoptera.

Towards the opposite end, we also conducted meta-analyses with only records that are flagged “green” in all critical appraisal criteria (Fig. 10B, Additional file 7: Table S7.4). Relatively few records were available for these analyses (371) compared to 2220 in total and 1976 when only red flagged records were excluded.

The negative effect sizes for Diptera, Syrphidae, and Hymenoptera were confirmed. In addition, a positive effect on Coccinellidae became apparent. Overall, analyses with only green flagged records showed broader confidence limits because of the low number of available records compared to the other two analyses, but results were qualitatively similar with the exception of Coccinellidae.

#### Functional groups

**Fig. 11:**
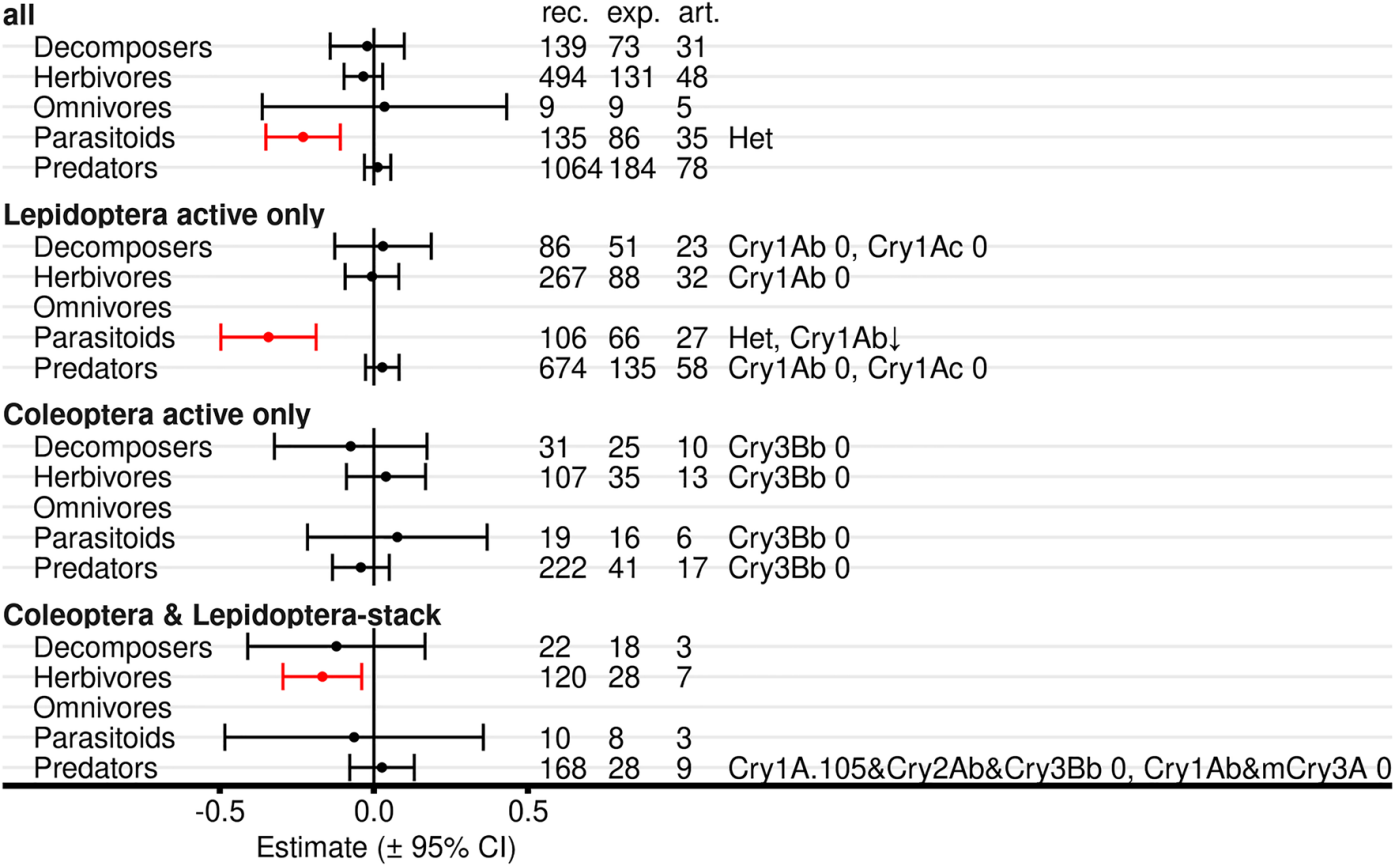
Meta-analyses on functional group level for untreated Bt and non-Bt maize. Either records of all Bt proteins were included, or only Lepidoptera-active, Coleoptera-active, or stacked Coleoptera- and Lepidoptera-active Bt proteins. Records with any red flag in the critical appraisal were excluded. For each functional group, the effect size estimate and the 95% confidence interval is given (negative effect sizes indicate lower populations in Bt compared with non-Bt maize and vice versa). Significant intervals (red) do not include 0. On the right side is the number of records (rec.), experiments (exp.), and articles (art.) included in each analysis. Het indicates significant heterogeneity. For details see Additional file 7: Table S7.5. Results of moderator analyses for individual Bt proteins (Additional file 7: Table S7.6) are indicated with arrows. ↓: lower values in Bt compared with non-Bt treatment (negative effect size), 0: no effect

Analyses on functional group level revealed no effect of Bt maize on decomposers, omnivores, and predators (Fig. 11, Additional file 7: Table S7.5, S7.6). Parasitoids showed lower populations in Bt maize (negative effect size) in the analysis with all Bt proteins (and significant heterogeneity), and in the analysis with Lepidoptera-active Bt maize (significant heterogeneity; moderator analysis significant for Cry1Ab). In addition, analysis for herbivores indicated lower populations in stacked Coleoptera- and Lepidoptera-active maize.

While most data in the database represent measures of abundance or activity density of invertebrates in maize fields, some experiments included direct measures of the parasitism or predation function. In addition to the analyses with all response variables (Fig. 11), we also analysed parasitism and predation functions separately. Parasitism, evaluated by field collected aphids, lacewing eggs, corn borer egg masses or corn borer larvae was comparable between Bt maize (all Cry1Ab) and non-Bt maize (effect size − 0.14 ± 0.15 [− 0.45; 0.16], N = 19 records, 8 experiments, 6 articles). Predation rates, determined with sentinel corn borer egg masses or artificial prey, also were similar between Bt and non-Bt maize (effect size − 0.035 ± 0.14 [− 0.31; 0.24], N = 14, 12 experiments, 5 articles).

#### Influence of plot size and years of Bt maize

On the functional group level, the influence of plot size was analysed (Table 1). The meta-regressions showed no significant effect of plot size for any functional group (confidence interval of slope included 0 in all cases). Similarly, the number of years of continuous Bt maize cultivation was analysed on functional group level by meta-regression (Table 1). No effects were evident for any functional group. This indicates that plot size and years of Bt maize cultivation had no influence on absolute effect sizes between Bt and non-Bt maize.

**Table 1:** Meta-regression effects of plot size and number of years with Bt maize cultivation on differences between Bt and non-Bt maize on functional group level

Functional group	Records	Experiments	Articles	Intercept	Slope
Plot size					
Decomposers	139	73	31	0.46 ± 0.062 (0.34; 0.58)	0.010 ± 0.022 (− 0.032; 0.053)
Herbivores	490	128	46	0.52 ± 0.033 (0.45; 0.58)	− 0.011 ± 0.019 (− 0.047; 0.025)
Parasitoids	131	82	33	0.57 ± 0.065 (0.44; 0.70)	− 0.013 ± 0.029 (− 0.069; 0.043)
Predators	1056	181	77	0.49 ± 0.022 (0.44; 0.53)	0.005 ± 0.014 (− 0.022; 0.032)
Years Bt maize					
Decomposers	94	45	27	0.47 ± 0.094 (0.28; 0.65)	− 0.020 ± 0.081 (− 0.179; 0.139)
Herbivores	329	91	37	0.46 ± 0.047 (0.37; 0.55)	0.027 ± 0.043 (− 0.058; 0.111)
Parasitoids	84	58	28	0.49 ± 0.092 (0.31; 0.67)	− 0.055 ± 0.087 (− 0.225; 0.115)
Predators	663	119	65	0.47 ± 0.033 (0.41; 0.53)	0.006 ± 0.034 (− 0.060; 0.072)

Records with any red flag in the critical appraisal were excluded. For effect sizes, the absolute values were used. Omnivores were not analysed because only 9 records were available for this group

#### Influence of private sector contribution

**Fig. 12:**
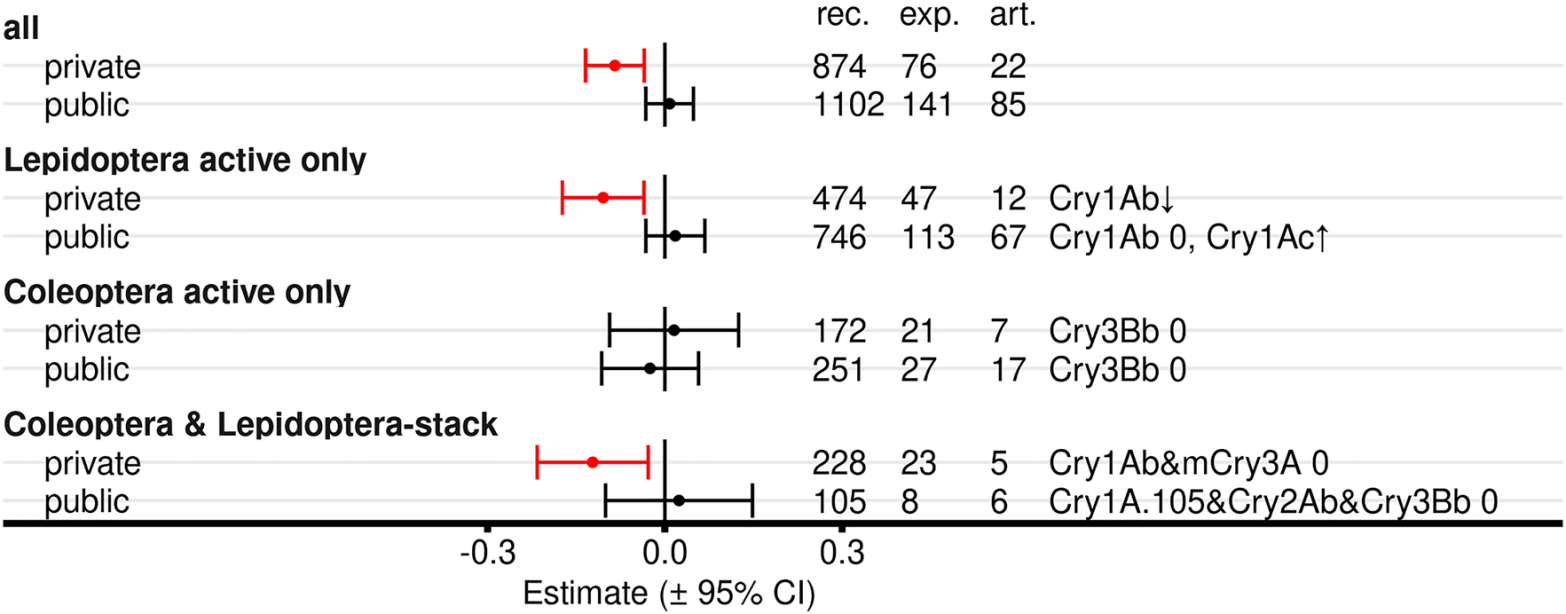
Meta-analyses on the influence of private sector contribution (private or public). The analyses examined all Bt proteins or only Lepidoptera-active, Coleoptera-active, or stacked Coleoptera- and Lepidoptera-active Bt proteins. Records with any red flag in the critical appraisal were excluded. For each analysis, the effect size estimate and the 95% confidence interval is given (negative effect sizes indicate lower populations in Bt compared with non-Bt maize and vice versa). Significant intervals (red) do not include 0. On the right side is the number of records (rec.), experiments (exp.), and articles (art.) included in each analysis. For details see Additional file 7: Table S7.7. Results of moderator analyses for individual Bt proteins (Additional file 7: Table S7.8) are indicated. ↓: lower values in Bt compared with non-Bt treatment (negative effect size), ↑: higher values (positive effect size), 0: no effect

We tested for differences in reported Bt effects when the study included authors from private sector product developers or if the study was sponsored by such members of the private sector (Fig. 12, Additional file 7: Table S7.7). For these analyses, records from all taxa were included, but records with red flags in the critical appraisal were excluded. When the private sector contributed to the data and all Bt proteins were analysed together, lower invertebrate populations in Bt maize (negative effect size) were observed. This effect derived mainly from Lepidoptera-active maize and stacked Coleoptera- and Lepidoptera-active maize (Fig. 12, Additional file 7: Table S7.7), and the Bt protein Cry1Ab (Additional file 7: Table S7.8). The articles [34] and [35] contributed the highest number of records to the overall analysis (124 and 353 of 874 records, respectively).

When only authors from public institutions and articles without declared private sector funding were selected, no difference between Bt maize and non-Bt maize was evident (Fig. 12, Additional file 7: Table S7.7). However, moderator analyses indicated significantly positive effect sizes in Cry1Ac-producing maize compared with non-Bt maize (Additional file 7: Table S7.8).

#### Funnel plot

A common criticism of meta-analyses is that authors tend not to publish non-significant results—the so called “file drawer problem”. The funnel plot with records from all taxa and all Bt proteins (with red flagged records excluded) shows a symmetrical distribution of data points, thus suggesting a lack of publication bias (Egger’s test, z = − 0.8608, p = 0.39) (Additional file 7: Figure S7.1).

#### Specific analyses for taxonomic subgroups and species

**Fig. 13:**
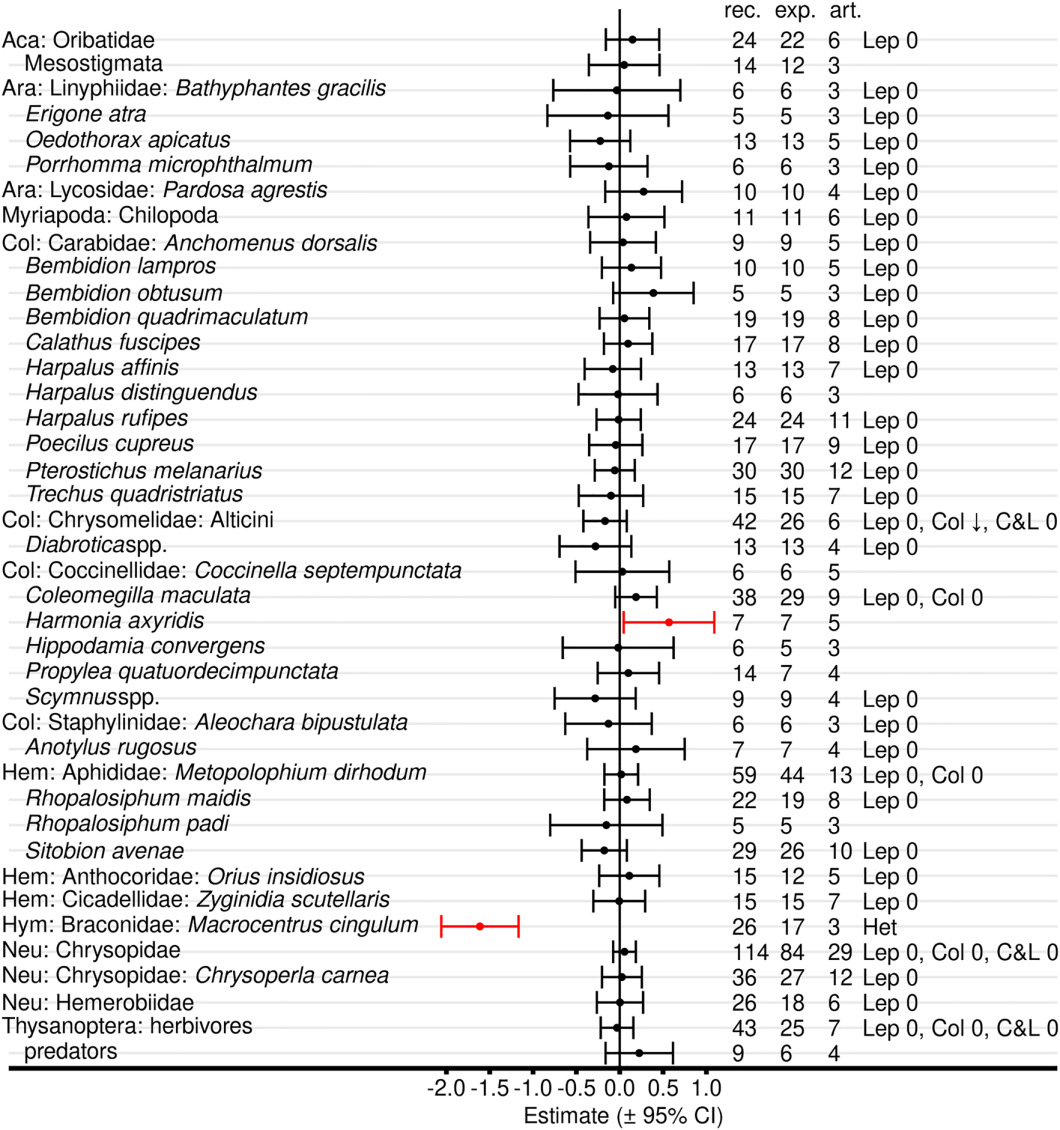
Meta-analyses on taxonomic subgroups and species for untreated Bt and non-Bt maize. Records with any red flag in the critical appraisal were excluded. All Bt proteins were included. For each taxon, the effect size estimate and the 95% confidence interval is given (negative effect sizes indicate lower populations in Bt compared with non-Bt maize and vice versa). Significant intervals (red) do not include 0. On the right side is the number of records (rec.), experiments (exp.), and articles (art.) included in each analysis. Results of moderator analyses with target order of Bt proteins (Additional file 7: Table S7.10) are indicated: ↓ lower values in Bt compared with non-Bt treatment (negative effect size), 0: no effect (Lep = Lepidoptera-active, Col = Coleoptera-active, C&L = stacked Lepidoptera- and Coleoptera-active)

Sufficient records were available to analyse a total of 31 individual species and 8 other taxonomic subgroups. Of those, only 2 showed significant effect sizes: higher numbers of *H. axyridis* and lower numbers of *M. cingulum* were recorded in Bt maize compared with non-Bt maize (Fig. 13, Additional file 7: Table S7.9). Moderator analyses were conducted for data of different target orders of Bt proteins (Additional file 7: Table S7.10). Sufficient data were available mainly for Lepidoptera-active Bt proteins. None of these analyses revealed significant effects, except for Alticini (Col: Chrysomelidae), which were less abundant in Coleoptera-active Bt maize.

An additional analysis was conducted with Lepidoptera-active maize and parasitoids, excluding parasitoids of target (*O. nubilalis*) larvae, such as Braconidae and Tachinidae. Based on 71 records from 43 experiments and 22 articles, the effect size was 0.0032 [− 0.1704; 0.1640] without heterogeneity (Q = 44.74; p = 0.99).

#### Specific analyses for individual sampling methods and juvenile life stages

Data for the different sampling methods were analyzed in a second set of specific analyses. For the different taxa, 61 analyses were possible with at least 5 records from 3 articles (Additional file 7: Fig. S7.2, Table S7.11). Lower populations in Bt maize (negative effect sizes) were detected with sticky traps for Staphylinidae, Syrphidae, and Braconidae, and with beat cloths for Aphididae. Higher populations in Bt maize (positive effect sizes) were observed with sticky traps for Coccinellidae, and with visual counts for Anthocoridae and Neuroptera. Moderator analyses revealed negative effect sizes in Lepidoptera-active Bt maize for Syrphidae collected with sticky traps and in Coleoptera-active Bt maize for Chrysomelidae collected with sticky traps (Additional file 7: Table S7.12). Positive effect sizes were present in Lepidoptera-active Bt maize for Coccinellidae collected with sticky traps and Anthocoridae recorded with visual counts and in stacked Bt maize for Neuroptera recorded with visual counts.

Furthermore, we analyzed juvenile life stages for Carabidae and Anthocoridae, larvae and pupae of Coccinellidae, Syrphidae, and Tachinidae, and eggs of Coccinellidae and Neuroptera. In Bt maize (Lepidoptera-active), fewer larvae and pupae of Tachinidae and more eggs of Neuroptera were recorded compared with non-Bt maize (Additional file 7: Figure S7.2, Table S7.11, S7.12).

#### Insecticide treatments in non-Bt maize

**Fig. 14:**
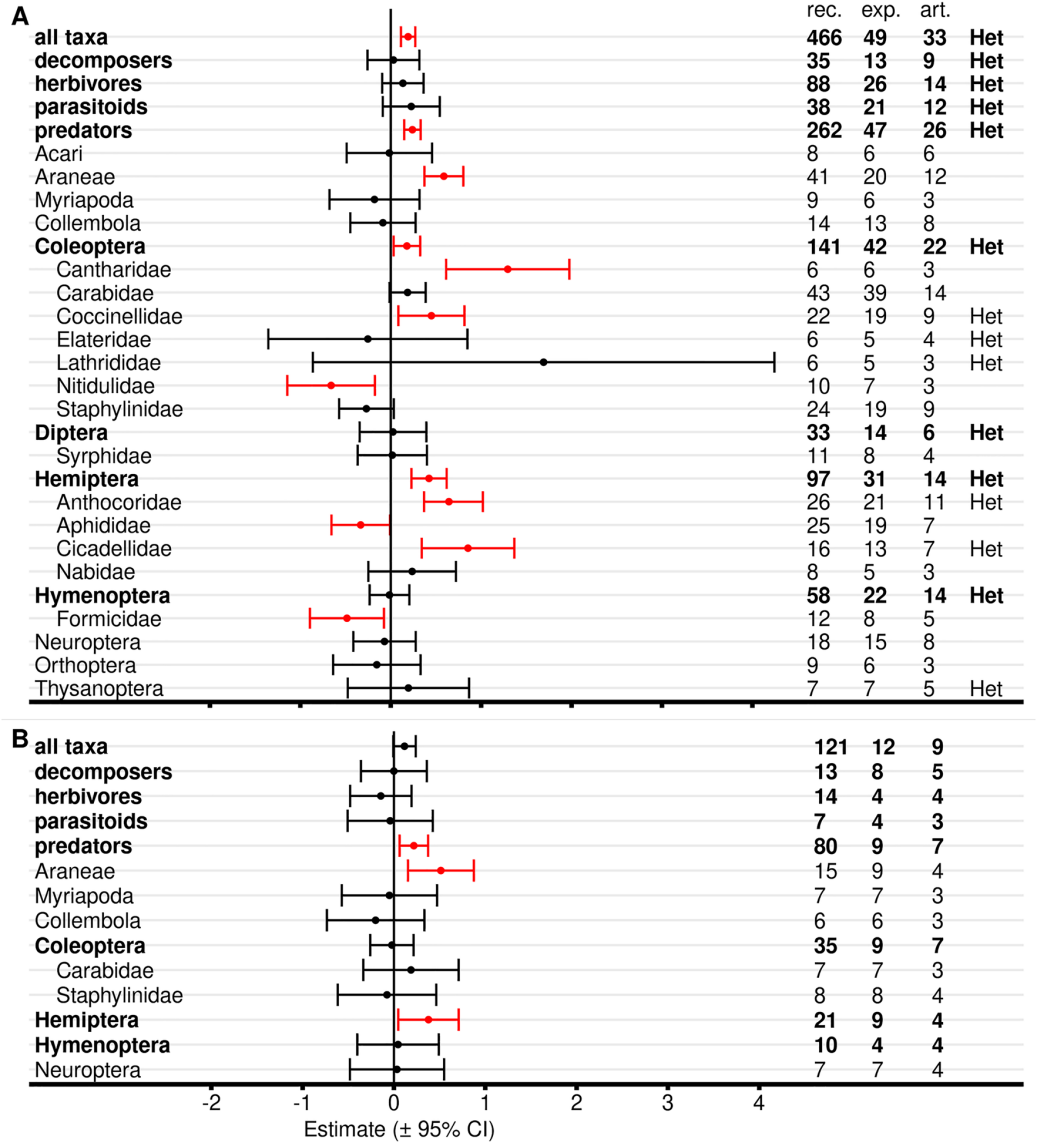
Meta-analyses on different taxonomic levels and functional groups for insecticide-treated non-Bt maize. **A** Pyrethroid- and **B** chloro-nicotinyl-treated non-Bt maize were compared with untreated Bt maize. Records with any red flag in the critical appraisal were excluded. All Bt proteins were included. For each taxon, the effect size estimate and the 95% confidence interval is given (negative effect sizes indicate lower populations in Bt compared with non-Bt maize and vice versa). Significant intervals (red) do not include 0. On the right side is the number of records (rec.), experiments (exp.), and articles (art.) included in each analysis. Het indicates significant heterogeneity

When untreated Bt maize was compared with pyrethroid treated non-Bt maize (mainly foliar and soil application), positive effect sizes (higher populations in Bt maize) were observed for Araneae, Cantharidae, Coccinellidae, Anthocoridae, and Cicadellidae and on higher taxonomic levels for Coleoptera, Hemiptera, and all taxa (Fig. 14A, Additional file 7: Table S7.13). Furthermore, predatory species were more abundant in untreated Bt maize compared with pyrethroid-treated non-Bt maize. In contrast, negative effect sizes (lower populations in Bt maize) were observed for Nitidulidae, Aphididae, and Formicidae. Heterogeneity was significant for half of the analysed taxa and all functional groups.

Fewer records were available for seed treatments with chloro-nicotinyl insecticides (Fig. 14B, Additional file 7: Table S7.13). Positive effect sizes were observed for Araneae, Hemiptera and the functional group of predators; no heterogeneity was detected for any analysed taxon or functional group.

Organophosphorous insecticides and microbial insecticides could only be analysed for Coleoptera, the functional group of predators, and all taxa combined (Additional file 7: Table S7.13). No significant effect sizes were observed for Coleoptera and all taxa (organophosphorous or microbial). In contrast, more predators were collected in untreated Bt maize compared to non-Bt maize treated with organophosphorous or microbial insecticides.

#### Robustness of significant effect sizes

Fail safe numbers for comparisons with untreated Bt and non-Bt maize indicate that the observed significances of effect sizes are not robust (fail safe number < 5n + 10 [31]) except for the comparisons involving Braconidae (Additional file 7: Table S7.14). The “leave one out” analyses revealed that the majority of significant effects were robust when one record or one experiment was removed at the time. For 9 of 39 significant effects, however, at least one record was identified that changed the respective analysis to non-signicficant when removed and 15 significant effects turned to non-significant when at least one experiment was removed. When at least one article was removed, 34 significant effect sizes turned to non-significant, while only 5 effect sizes remained significantly negative (including Diptera, Tachinidae, and *M. cingulum*). Three articles had a high influence on the significance of effect sizes [34–36]. Those are also the articles that contributed most records to a range of different analyses. For example, for the analysis on all taxa and Bt proteins with no records excluded, Ref. [35] contributed 358, Ref. [36] 158, and Ref. [34] 126 of 2220 total records (Additional file 7: Table S7.4).

When non-Bt maize was treated with pyrethroids, fail safe numbers indicate robustness of positive effect sizes (higher populations in untreated Bt maize compared to insecticide-treated non-Bt maize) for all taxa, Araneae, Hemiptera, and predators. The “leave one out” analyses with pyrethroid-treated non-Bt maize revealed that 2 out of 12 significant analyses turned to non-significant when at least one record was removed and 3 when one experiment was removed. When one article was removed, 4 analyses turned to non-significant, while 7 effect sizes remained significantly positive (all taxa, Araneae, Cantharidae, Hemiptera, Anthocoridae, Cicadellidae, and predators) and one effect size significantly negative (Nitidulidae). When non-Bt maize was treated with chloro-nicotinyl, microbial insecticides, or organophosphorous, fail safe numbers indicate that significant effects were not robust. The “leave one out” analyses showed non-significant effects in 2 of 5 cases when one record was removed, and 4 cases when one experiment or one article was removed. The positive effect size for Araneae in Bt maize compared with chloro-nicotinyl-treated non-Bt maize remained significant in all “leave one out” analyses.

### Interpretation of review findings

Our main meta-analyses revealed no significant effect sizes for 36 of 39 analyzed taxa (Fig. 8, Additional file 7: Table S7.1) when both Bt maize and non-Bt maize were not treated with insecticides. More significant effect sizes on different taxa (8 of 20) were evident when untreated Bt maize was compared with alternative methods (pyrethroids and other insecticides) employed for pest control in non-Bt maize (Fig. 14, Additional file 7: Table S7.13). Authors’ conclusions for experiments summarized narratively (Additional file 3) were similar to the results of our statistical meta-analyses. Studies conducted in off-crop habitats and on vertebrates, however, were rare. In the following, the significant effects observed in the different statistical analyses are interpreted and a reference to previous meta-analyses is provided.

#### Effects of Bt maize on parasitoids of target pests

The overall negative effect of Bt maize on Braconidae (Hymenoptera) (Fig. 8) can be tracked down to Lepidoptera-active maize producing Cry1Ab (Fig. 9), and to the species *M. cingulum* (Fig. 13). Braconidae are parasitoids of insect larvae and *M. cingulum* is a parasitoid specialized to corn borer larvae of the genus *Ostrinia*, the target pests of Lepidoptera-active Bt maize. The parasitoid was introduced to the USA almost 100 years ago and established as an effective biocontrol agent. The effects reported from meta-analyses derived from data of two articles with *M. cingulum*: Bruck et al. [37] (2 records) and Pilcher [34] (21 records). In both studies, *M. cingulum* adults were collected with sticky traps. In addition, Siegfried et al. [38] observed reduced parasitism rates of diapausing *O. nubilalis* larvae collected in commercial Bt maize (event 176) compared with non-Bt maize fields by *M. cingulum* or *Eriborus terebrans* (Hym.: Ichneumonidae). No overall effect on the parasitism rate of *O. nubilalis* in event 176 maize was found by Orr & Landis [39]; most of the recovered parasitoids, however, were *E. terebrans*, while only few specimens of *M. cingulum* were collected in non-Bt maize (0–25% of parasitized larvae, depending on plot) and none in Bt maize. The observed effect size on Braconidae in several analyses can be considered robust according to fail safe numbers, but the analyses turned to non-significant when article [34] was removed (Additional file 7: Table S7.14). From experiments presented in the narrative summary tables, Venditti and Steffey [40] reported that *M. cingulum* parasitism of first generation *O. nubilalis* larvae did not differ among maize types (non-Bt and Bt), but a significant effect was present for the second generation, mainly because in one of two years, no parasitized larva was collected in Bt maize, while 8.6% of the larvae were parasitized in non-Bt maize (in the other year, 7.8% and 12.6% parasitism was reported in Bt and non-Bt maize, respectively) (Additional file 3: Table S3.5). Similarly, the number of *M. cingulum* adults captured in non-Bt plots in another study was double the number captured in Bt plots [41] (Additional file 3: Table S3.5). Effects of Lepidoptera-active Bt maize on *M. cingulum* also have been reported in previous meta-analyses [13, 14, 16].

The second group of parasitoids of *O. nubilalis* is Tachinidae, which also were less abundant in Lepidoptera-active Bt maize. The effect, however, was not robust according to fail safe numbers. Bourguet et al. [42] collected *O. nubilalis* larvae and recorded reduced parasitism by *Lydella thompsoni* and *Pseudoperichaeta nigrolineata* (both Diptera: Tachinidae) in Cry1Ab-producing Bt maize (event 176) in France. Effects on abundance and parasitism by Tachinidae in Italy were confirmed [43–45]. Records from Madrid et al. [35], who collected Tachinidae in Cry1A.105 & Cry2Ab producing maize in Brazil, also may have contributed to the overall negative effect on this family.

Whenever measures are taken to reduce target pests, e.g., by biological control, pesticides, or Bt crops, an impact on the natural enemy community of those pests is expected [46]. Reduced numbers of target pests, reduced nutritional quality of the remaining ones, and lack of host-derived or induced attractant cues may particularly affect parasitoids specialized to target pests, such as *M. cingulum* or Tachinidae [34, 47]. In contrast, no effects on *M. cingulum* were reported from Coleoptera-active maize [48] and no overall effect of Coleoptera-active maize on parasitoids was observed in our meta-analyses.

The effects on parasitoids of *O. nubilalis* in our analyses also contributed to effects at higher taxonomic levels, i.e., Hymenoptera, Diptera and the functional group of parasitoids and to heterogeneity (Hymenoptera, parasitoids). When we repeated the meta-analyses on these groups for Lepidoptera-active Bt maize without records of Braconidae (*M. cingulum*) and Tachinidae, no effect was observed and heterogeneity disappeared, indicating that parasitoids were only affected when specialized on *O. nubilalis* larvae. This has been discussed previously [13].

#### Effects of Bt maize on beetles

Many Nitidulidae (sap beetles) are fungivores or herbivores that are attracted to maize plants with corn borer tunnels, other damage, or exposed kernels, where they can feed on fungi growing on the plant wound exudates or directly on the kernels [37, 49]. In Lepidoptera-active Bt maize, pitfall traps and plant samples recorded less Nitidulidae than in non-Bt maize [37, 49], and Bt sweet corn showed less kernels damaged by Nitidulidae [50, 51], resulting in a negative effect size in our meta-analyses. The effect, however, was not robust according to fail safe numbers and “leave one out” analyses. Effects on Nitidulidae might be linked to the reduced Lepidoptera damage and thus to the reduction of attractive cues for the beetles [37, 49]. However, no clear relationship between Bt status and the presence of sap beetles in pheromone baited traps was reported by Dowd [52]. Our meta-analyses also showed a reduced number of Nitidulidae in Bt maize when compared with pyrethroid-treated non-Bt maize (Fig. 14).

When records for non-targets associated with Bt maize targeting Lepidoptera, Coleoptera, or stacks were examined together, a negative effect size on Staphylinidae was present, i.e., fewer rove beetles were recorded in Bt maize compared with non-Bt maize (Figs. 8, 10). The effect was not robust according to fail safe numbers and “leave one out” analyses. No significance was present when each Bt maize type was analyzed separately (Fig. 9), or when records with only green flags were analyzed (Fig. 10B). There also were no effects on the staphylinid species *Aleochara bipustulata* and *Anotylus rugosus* when analyzed individually (Fig. 13). In contrast to the results for parasitoids of target Lepidoptera pests and for Nitidulidae, the overall effect on Staphylinidae thus cannot be linked to particular types of Bt maize, Bt proteins, or species. In addition, no heterogeneity was found in the dataset and no individual studies with many or high weighted observations were obvious that could be associated with the observed effect. When sampling methods were analyzed separately, a significant, but non-robust negative effect size was shown for sticky traps (17 records), but not for pitfall traps (61 records), or litter extraction (8 records). The study by Szenasi et al. [36] was mainly responsible for the negative effect size seen with sticky traps. Laboratory studies do not support the hypothesis of direct effects of Bt maize on Staphylinidae. Studies with *Dalotia coriaria* (Col.: Staphylinidae) indicated no effects on different life table parameters when beetles were fed spider mites reared on Cry1Ab-[53] or Cry3Bb-producing maize [54] or when purified Cry3Aa and Cry1Ab was provided in artificial diet [55]. It is thus likely that the weak negative effect size observed for staphylinids in our meta-analyses may have derived from indirect, food web related effects, other ecological factors, or from chance.

In contrast to Staphylinidae, Coccinellidae (lady beetles) showed higher populations in Bt maize (non-robust positive effect size) when records with all green flags were analyzed (Fig. 10B) and in specific analyses with sticky trap data. Confidence intervals included zero, however, in main analyses with all records (Fig. 10A), with red flagged records excluded (Fig. 8), or in analyses of Bt maize targeting different orders (Fig. 9). One study [34] dominated the analysis of all-green flagged records as it contributed 20 of 38 records. On the species level, a weak positive effect was observed for *H. axyridis* (Fig. 13). The mechanisms behind these patterns remain uncertain, but a positive effect does not support a hypothesis of toxicity of Bt proteins to lady beetles.

#### Effects of Bt maize on hoverflies

When red flagged records were excluded, the upper boundary of the confidence interval for Syrphidae was 0.0000 (Fig. 8). A borderline, non-robust negative effect of Bt maize on Syrphidae was present when all records (including red-flagged ones) were analysed, when only green-flagged records were analysed, and when sticky trap data were analysed, while red-flagged records were excluded (Fig. 10, Additional file 7: Table S7.11). However, no significance was obtained when Bt maize targeting different orders or Bt proteins were analysed separately (Fig. 9). One study [34] with Cry1Ab-producing maize (21 of 63 records, red flags excluded, all target orders) had a high influence on those analyses with mostly negative effect sizes. Similar to the group of Staphylinidae, indirect, food web or other ecological effects, or chance may have caused the weak negative effect sizes.

#### Effects of stacked Bt maize on herbivores

A weak (non-robust) negative effect of stacked Bt maize on the herbivore functional group was observed, but no such effect was evident for Lepidoptera-active or Coleoptera-active maize (Fig. 11). The analysis with stacked maize was dominated by two articles, [35] (68 of 120 records) and [36] (28 records). The negative effect might derive from data on Otitidae (Diptera), but this needs to be interpreted with caution, because the data come from a single study [35]. *Euxista* spp. (Otitidae) are sweet maize pests in the Americas. The flies prefer damaged tissue or the silk at the tip of the ears for oviposition, so a connection between damage caused by Lepidoptera target pests of Bt maize and Otitidae is possible, similar to the case of Nitidulidae [56].

#### Other effects of Bt maize

Taxonomic subgroup analyses revealed reduced numbers of Alticini (flea beetles) in Coleoptera-active Bt maize, which might reflect toxicity of Cry3Bb-producing Bt maize, because Alticini belong to the target group of Chrysomelidae. In contrast, higher populations of Anthocoridae (flower bugs) were recorded in Lepidoptera-active Bt maize (Cry1Ab, visual counts) and higher populations of Neuroptera (lacewings) were recorded in Bt maize with visual counts (of all stages) and when only eggs were analyzed. The reasons for those positive effect sizes remain unclear, but effects were not robust according to fail safe numbers and “leave one out” analyses.

#### Effects of insecticide treatments in non-Bt maize

Most of the records for insecticide-treated non-Bt maize were for pyrethroids that were mainly applied as foliar sprays, sometimes also as soil insecticides, and for seed treatments with chloro-nicotinyl. Insecticides from other classes, such as organophosphorous, microbial, carbamate, or oxadiazine insecticides were only used in few studies and the required minimum numbers of records (5) and articles (3) for meta-analyses were not available in most cases. Untreated Bt maize harboured generally more arthropods than pyrethroid treated non-Bt maize (all taxa combined, Fig. 14). Positive effect sizes (higher populations in untreated Bt maize compared with treated non-Bt maize) that were robust according to fail safe numbers and “leave one out” analyses were observed for all taxa combined, spiders (Araneae), Hemiptera, and the functional group of predators. Positive, non-robust effect sizes were present for beetles (Coleoptera), in particular Cantharidae and Coccinellidae, Anthocoridae and Cicadellidae. It can be hypothesized that those taxa were negatively affected by the insecticide applied to non-Bt maize and therefore, higher populations were recorded in untreated Bt maize. In contrast, negative and non-robust effect sizes (fewer individuals recorded in Bt maize) were obtained for Nitidulidae, Aphididae, and Formicidae. The relationship of Nitidulidae with damaged maize was discussed previously, and perhaps this relationship was present despite the pyrethroid sprays. For aphid populations, it has been reported frequently that they recover quickly after insecticide sprays, while their natural enemies need more time to recover or to recolonize the fields [57]. It remains unclear why ants (Formicidae) were more abundant in pyrethroid-treated fields. Maybe they showed increased activity (movement) after pyrethroid application (most common collection method was pitfall traps), or they responded to changed food availability (e.g., more aphids). Approximately half of the analysed taxa and all functional groups (Fig. 14) showed significant heterogeneity in the pyrethroid treatments, which demonstrates that within those taxa, some studies showed more effects than others and/or the direction of effect sizes was variable.

Seed treatments with chloro-nicotinyl in non-Bt maize also resulted in higher populations in the untreated Bt maize (positive, non-robust effect sizes) for spiders (Araneae), Hemiptera, and for the functional group of predators (Fig. 14). Organophosphorous and microbial insecticides similarly affected predators. In the case of microbial insecticides, the effect can be linked to one study that used spinosad [58], while studies using Bt products showed no significant effect size.

Overall, comparisons of insecticide-treated non-Bt maize with untreated Bt maize resulted in more significant effect sizes, higher robustness, but also higher heterogeneity than analyses of untreated non-Bt and Bt maize. In particular predatory species were harmed by insecticide treatments. Previous meta-analyses came to the same conclusion [13, 14].

#### Influence of publication bias and private sector contribution

Non-robust negative effect sizes were seen in studies with private sector contribution (authors and/or funding from private sector product developers), while no Bt effects were evident in studies without such private sector contribution (Fig. 12). Two articles were mainly responsible for the negative effect size as they contributed more than half of the records in the group of private sector contribution: [34] and [35]. This does not support the hypotheses that the private sector might have vested interests in hiding (adverse) effects data while public sector scientists might be more interested in publishing effects. Such conflicts of interest have been suggested, for example, for studies on Bt crop efficacy and durability [59] and for studies on health risks and nutritional value of GM crops [60].

It has to be noted that ecological studies are not pre-registered (as common in medical science), so it is impossible to know which proportion of conducted studies is being published. The funnel plot for records of all taxa combined (Additional file 7: Figure S7.1), however, revealed a balanced distribution of effect sizes, providing no evidence that non-significant results are not being published [29].

#### Consistency with previous meta-analyses on non-target animals in Bt crops

The first meta-analyses on field abundance of non-target invertebrates in Bt maize (Cry1Ab and Cry3Bb) and Bt cotton (Cry1Ac) by Marvier et al. consided articles up to 2006 [10]. Systematic literature searches were conducted, defined inclusion criteria were used, authors were contacted for missing information, and rules were defined for the selection of datasets for meta-analyses. In Cry1Ab producing maize, significantly fewer invertebrates were recorded compared with untreated non-Bt maize and this effect was largely attributed to a reduced number of parasitic wasps (Hymenoptera). More non-target invertebrates, however, were recorded in untreated Bt maize compared to pyrethroid-treated non-Bt maize. Overall effects of Cry3Bb-producing maize were insignificant, either without or with pyrethroid application in non-Bt maize [10]. Follow-up analyses of the same dataset by functional guilds confirmed that Bt maize (data for Cry1Ab- and Cry3Bb-producing maize combined) compared to untreated non-Bt maize reduced parasitoid abundance, while effects on predators, herbivores, omnivores, detritivores and mixed feeders were insignificant [13]. When compared to pyrethroid-treated non-Bt maize, untreated Bt maize revealed higher numbers of predators, herbivores, and mixed feeders and lower numbers of omnivores and detritivores [13]. Naranjo [14] updated the database established by Marvier et al. [10] with articles up to 2008 and repeated the analyses on different functional groups in Bt maize, cotton, and potato with essentially the same result. Data from 13 (partly unpublished) Bt maize field trials in Spain with different Cry proteins (single or stacked), but conducted in comparable layout and with similar arthropod sampling methodology were subject to meta-analyses by Comas et al. [15]. None of the analysed taxa for each sampling method (7 taxa for visual sampling, 7 for pitfalls, 12 for sticky traps) showed effect sizes significantly different from zero. Pellegrino et al. [16] conducted meta-analyses on field data of agronomic, environmental, and toxicological traits of GM maize and non-target organisms were part of this work. Only peer-reviewed literature from the Web of Science Core Collection until 2016 was considered. Authors were not contacted, but the database by Wolfenbarger et al. [13] was consulted when data could not be extracted directly from the articles. Analyses (Lepidoptera- and Coleoptera-active Bt maize combined) revealed no significant effect sizes on Anthocoridae, Aphididae, Araneae, Carabidae, Chrysopidae (larvae and adults), Coccinellidae (larvae and adults), Nabidae, Nitidulidae, a strong negative effect of Bt maize on Braconidae, and a weak positive effect on Cicadellidae. A systematic review including meta-analyses on soil invertebrates inhabiting Bt crops was conducted by Krogh et al. [17]. Over all crops and Bt proteins, no effects on soil invertebrates were detected. The conclusions of our work are largely consistent with the conclusions drawn previously. Our work, however, provides more in depth meta-analyses (e.g., higher taxonomic resolution and separate analyses for different target orders of Bt maize) on a much larger dataset and with consideration of validity issues.

#### Evidence from laboratory studies

With the exception of Staphylinidae [53–55], no controlled exposure studies to examine direct toxic effects of Bt proteins are available for the taxa where our meta-analyses showed lower populations in Bt maize. However, numerous non-target laboratory studies with a wide range of taxa using purified Bt proteins, plant material, or prey that had consumed Bt proteins have demonstrated the specificity of Cry1- and Cry2-class proteins to Lepidoptera and Cry3-class proteins to Chrysomelidae (Coleoptera) [47, 61–63]. This also has been concluded from several meta-analyses of laboratory data [11, 12, 14]. Putative adverse effects of Bt proteins or Bt plant material on non-target species have been reported in a review of laboratory studies [64], but such effects appeared to be linked to natural enemies being provided with sublethally affected hosts or prey [65]. Our field data, showing no effects for 36 analyzed taxonomic groups, thus largely confirm the laboratory data. Negative effects of Bt maize observed in some meta-analyses on Braconidae, Tachinidae, and Nitidulidae are likely linked to reduced numbers of corn borer larvae in Lepidoptera-active maize, while negative effect sizes for Staphylinidae and Syrphidae and positive effect sizes for Anthocoridae, Coccinellidae, and Neuroptera remain unexplained. Those effects, however, were not robust and significant only in some analyses. Direct toxicity of the plant-produced Bt proteins is thus unlikely.

## Review limitations

### Critical appraisal

Criteria for critical appraisal were developed and applied to all datasets in the database. Most records had high or medium internal and external validity (green or yellow flag). Nevertheless, only few studies had a green flag for all appraisal criteria (Fig. 10B). In particular, verification of Bt protein expression in the plant was often not considered by the study-authors. We also observed that information on field management was often incomplete and we decided to assume that no mentioning of interventions, such as pesticide applications, indicates the lack of such interventions. The critical appraisal was used to filter out potentially problematic studies, but we also conducted analyses with all records as well as with only-green-flagged records (Fig. 10). The individual critical appraisal questions and specific cut-off values were designed starting from generic schemes often used for systematic reviews and further refined and discussed with other scientists. Nevertheless, there is no commonly agreed critical appraisal scheme for animals collected in GE field studies and one might argue that some of our cut-off values are arbitrary. For transparency reasons, however, it was important for us to have clearly defined cut-off values. Our critical appraisal may provide guidance on the kind of information that is important to judge the reliability of a study. In general, we call for more detailed descriptions of the materials and methods used in future publications of field studies. In particular, expression of Bt protein should be addressed by the authors, even if using commercial events, and clearly specified variance terms should be reported along with means and sample sizes. Blinding or masking treatment information for the staff recording data in the field, as it is common in medical science, was not reported by study authors and therefore not assessed. Such procedures, however, are recommended for future entomological or ecological studies to reduce the risk for bias due to intentional or unintentional personal opinions.

### Data independence

Previous meta-analyses [10, 12–14] selected data on the finest available taxonomic level. For example, one study reported effects of Bt maize on ground beetles on species level, which would have resulted in 50 records in our database. Another study just reported an aggregated value for all carabid species, resulting in one record in the database. Consequently, the meta-analysis would have been conducted with 51 records. Such an imbalance on taxonomic levels may lead to bias towards individual studies because the different species collected in the same plots and years should not be considered independent. In the current work, we thus aggregated all lower taxa (mainly species) to a total of 39 taxa on family, order, or higher level for our main meta-analyses (Fig. 8) so that each record in the meta-analyses represented data from a unique plot, location, and year. Despite this effort to achieve data independence, data from control plots were used multiple times if different Bt maize lines had the same control, so a certain level of data-dependence remained. When experiments included several sampling methods that recorded the same taxa, we selected one method with the lowest CV to avoid data-duplication in a given meta-analysis. Because we acknowledge that different methods may not be identical in the proportion of recorded species and life stages, we also conducted specific analyses for the different sampling methods (Additional file 7: Table S7.11, S7.12) Lack of independence also may occur when multiple records are derived from the same article. In fact, some articles (e.g., [34–36]) contributed high numbers of records as they reported on experiments with multiple transformation events from several locations and years. Within one article, potentially the same crew of people used the same batches of seeds and the same data recording and processing methodology, which may introduce bias due to records not being independent. Furthermore, overrepresentation of particular batches of seeds, experimental fields or regions may lead to local effects that may not be representative for Bt maize cultivation in general. Such potential limited validity, however, is not captured in our meta-analyses that use individual records as the unit of analysis. To address the issue of high influence of individual articles, we provide information on which article contributed how many records to each analysis and we performed “leave one out” analyses (Additional file 7).

### Geographic coverage

Most data included in this review derived from field studies conducted in North America and Europe, while only few datasets were available from South America, Africa, and Asia. South America is particularly underrepresented as it represents approximately one third of the global Bt maize production [66].

### Uncertainty

Another limitation is the fact that some SD values had to be estimated by averaging rather than by exact calculations. Those estimated SDs were generally higher than the calculated ones and thus more conservative, reflecting the uncertainty of this estimation.

### Data availability

Data availability in general was a major drawback for the construction of the database. These limitations have been emphasized in prior meta-analyses [13], but the problem persists. For a large number of studies, authors had to be contacted because detailed data or some crucial information was lacking in the articles and in several cases our attempt to get this information was fruitless. Consequently, a number of relevant studies could only be summarized narratively (Additional file 3: Table S3.5), but not used for quantitative analyses. Therefore, we urge authors to provide detailed datasets on replicate resolution along with each article. Such data availability is already demanded by many journals, which will help greatly for future meta-analyses.

### Private sector contribution

We considered the influence of private sector contribution on available results of field studies. Private sector contribution was indicated if authors were affiliated with private sector product developers and/or if financial support from such members of the private sector was acknowledged. The actual influence of the private sector and other institutions or funding bodies on study results and the completeness of the authors’ self-declarations, however, remain unclear.

### Relevance of historical data

In the 25 years of Bt maize cultivation, some transformation events used in early products (e.g., event 176) have been replaced. In addition, more and more stacked events have been introduced. Our analyses include experiments with all transformation events and planting years, because the principal mechanisms and spectrum of activity of the produced Bt proteins (Cry1, Cry2, or Cry3 type proteins) remained the same. Of all records in the present database, 39% are from studies published later than 2008, so our meta-analyses are based on a substantially larger dataset compared with others [14].

### Plot size

Although our meta-regressions suggested a general lack of effect of plot size on outcomes at the level of functional guild, we strongly encourage researchers to employ plots as large as feasible, especially when assessing mobile organisms [67]. The fact that there was no correlation of plot size and effect size in our review may be explained by the general lack of substantial effects of Bt proteins on non-target invertebrates. However, we cannot exclude the possibility of plot size effects on particular taxa and life stages. Most non-target studies were conducted in replicated plots within a field. Depending on the mobility of the studied taxa, extrapolating conclusions from such small-scale plot-experiments to actual farmer fields might be limited [68, 69].

### Animals other than in-crop invertebrates

This review is limited to direct comparisons of animals recorded in Bt and non-Bt maize fields and their adjacent habitats. Some groups of non-target species, such as bees and other pollinators, butterflies and moths, aquatic organisms, or vertebrates are usually not studied in such experimental setups. Experiments for those groups may comprise specimens or colonies placed in fields, landscape studies, experiments in cages in the glasshouse or in the laboratory, modelling studies, or feeding studies with harvested plant material [11, 70–72]. The conclusions of our review are thus largely limited to invertebrates inhabiting maize fields. Furthermore, we focused on population measures, such as abundance and activity density. Community measures, such as biodiversity indices were captured in tables and summarized narratively (Additional file 3), but were quite inconsistent among studies in terms of taxonomic inclusion and reporting of outcomes. Additional effort is needed in this area to have sufficient data for robust meta-analyses.

### Multiple statistical tests

It has to be noted that we conducted many individual statistical tests with different datasets without correcting confidence limits for multiple testing. While this ensures a high probability for detecting potential effects, some significant effect sizes (negative or positive) might be a product of chance, especially when the observed effects are not robust and one boundary of the confidence limit is close to zero, because for 95% confidence intervals, there is a chance of 5% that the true effect size is outside the estimated interval.

## Review conclusions

### Implications for policy

Our review provides evidence that Bt maize represents a highly selective pest control technology with relatively few negative consequences on a wide array of taxa associated with maize production, especially when compared with the alternative use of broad-spectrum insecticides for managing Bt-targeted pests. The Bt maize transformation events that have been cultivated commercially worldwide have all gone through regulatory environmental risk assessments that concluded that no unacceptable risks for non-target organisms and biodiversity exist [2, 73, 74]. Our systematic review generally supports these conclusions.

### Implications for research

Our results largely agreed with prior meta-analyses focused on or including Bt maize. The robust approach ameliorates many of the shortcomings of prior analyses, such as level of analysis, data-dependence issues, and consideration of internal and external validity. One shortcoming of this current review and all prior ones, however, is the limited availability of appropriate data (in particular raw data) with the consequence that some data had to be estimated (rather than calculated) for the quantitative analyses, while other datasets could not be analyzed at all. Future articles should make full datasets publicly available to foster future meta-analyses and focus on taxonomic groups and geographies that are under-represented in the current database. More research is recommended on experimental plot sizes necessary for a better representation of farm scale conditions for animals with mobile life stages and multiple generations per season. Furthermore, research on the biological relevance of differences in field populations is needed. For example, some evidence suggests small changes in abundance do not impact biological control function in Bt cotton [75]. That is, even if statistical differences in experimental studies and meta-analyses are observed, how are such differences linked to ecosystem functions and can they lead to harm?

## Supplementary Information

**Additional file 1:** Literature search (2 Tables).

**Additional file 2:** References included in the full text screening.

**Additional file 3:** Narrative summary and tables—studies relevant for the systematic review but not fitting the database for quantitative analyses (5 Tables).

**Additional file 4:** Rules for extraction of data for the database.

**Additional file 5:** Data selected for meta-analyses.

**Additional file 6:** R-code for meta-analyses.

**Additional file 7:** Detailed results of statistical meta-analyses (14 Tables, 2 Figures).

**Additional file 8:** ROSES form.

## Data Availability

The full database including variable definitions and critical appraisal criteria that was used for the quantitative analyses in this review is publicly available (Ref. [25], direct link to data files: https://doi.org/10.5061/dryad.3j9kd51jq). Any other data are supplied online in the Additional files of this publication.
