# Supplementary material for: Does the growing of Bt maize change abundance or ecological function of non-target animals compared to the growing of non-GM maize? A systematic review
Source: Environ Evid. 2022 Jun 6;11:21. doi: 10.1186/s13750-022-00272-0 (PMC11378853; doi:10.1186/s13750-022-00272-0)
Supplement: Supplementary file 1 — Additional file 1: Literature search (2 Tables). [file 13750_2022_272_MOESM1_ESM.pdf]

Does the growing of Bt maize change abundance or ecological function of non-target animals compared to the growing of non-GM maize? A systematic review

Michael Meissle[1*], Steven E. Naranjo[2], and Jörg Romeis[1]
[1]Agroscope, Research Division Agroecology and Environment, Reckenholzstrasse 191, 8046 Zurich, Switzerland, michael.meissle@agroscope.admin.ch; joerg.romeis@agroscope.admin.ch
[2]USDA-ARS, Arid-Land Agricultural Research Center, 21881 North Cardon Lane, Maricopa 85138, Arizona, USA, steve.naranjo@usda.gov

[*] Corresponding author

Published in: Environmental Evidence (2022), https://doi.org/10.1186/s13750-022-00272-0

Additional file 1: Literature search (2 Tables)

Table S1.1: Literature searches in abstracting and full text databases. Presented is the database portal and hyperlink, the search string used in the respective portal, the dates of the search, the specific database searched, the number of references (hits) exported to the bibliographic software, and the specific settings used. The advanced search function was used where available.

Portal	Search string	Search Dates	Database	Hits[a]	Search settings
Web of Science (Clarivate Analytics) www.webofknowledge.com	TS = ((maize OR corn OR "Zea mays") AND (field OR plot OR location OR trial OR farm-scale OR scouting OR trap OR trapping OR sampling OR monitoring) AND (transgenic OR Bt OR "Bacillus thuringiensis" OR GM OR "genetically modified" OR "genetically engineered" OR Cry* OR Vip*))	9.12.2014 28.1.2019 23.8.2019	Web of Science Core Collection (WOS)	5508	Indexes: SCI-EXPANDED, SSCI, A&HCI, CPCI-S, CPCI-SSH, BKCI-S, BKCI-SSH, CCR-EXPANDED, IC.
			BIOSIS Citation Index (BIO)	3511	Indexes: BCI
			Zoological Record (ZOO)	618	Indexes: Zoological Record
			SciELO Citation Index (SCI)	90	Indexes: SCIELO
Ovid SP (Wolters Kluwer) http://ovidsp.tx.ovid.com	((maize OR corn OR Zea mays) AND (field? OR plot? OR location? OR trial? OR farm-scale OR scouting OR trap? OR trapping OR sampling OR monitoring) AND (transgenic OR Bt OR Bacillus thuringiensis OR GM OR genetically modified OR genetically engineered OR Cry* OR Vip*)).ti,ab,hw.	9.12.2014 28.1.2019 23.8.2019	Agricola (AOA)	1715	
			AGRIS (AGS)[b]	477	
			CAB Abstracts (CAB)	3515	
Bielefeld Academic Search Engine (BASE) www.base-search.net	(maize corn "Zea mays") AND (field* plot* location* trial* farm-scale scouting trap* trapping sampling monitoring) AND (transgenic Bt "Bacillus thuringiensis" GM "genetically modified" "genetically engineered" Cry* Vip*)	10.12.2014 28.1.2019 23.8.2019	ca. 3200 content sources (BAS)	1765	Search in entire document, first search was split in 2 searches: 1980-2007 and 2008-2014.
ProQuest LLC http://search.proquest.com	(maize OR corn OR "Zea mays") AND (field*1 OR plot*1 OR location*1 OR trial*1 OR farm-scale OR scouting OR trap*1 OR trapping OR sampling OR monitoring) AND (transgenic OR Bt OR "Bacillus thuringiensis" OR GM OR "genetically modified" OR "genetically engineered" OR Cry* OR Vip*)	11.12.2014 4.2.2019 23.8.2019	ProQuest Dissertations & Theses A&I (PRO)	300	3 parts of the search string in separate fields, connected with AND: search anywhere except full text - ALL
Scopus www.scopus.com	TITLE-ABS-KEY((maize OR corn OR "Zea mays") AND (field OR plot OR location OR trial OR farm-scale OR scouting OR trap OR trapping OR sampling OR monitoring) AND (transgenic OR Bt OR "Bacillus thuringiensis" OR GM OR "genetically modified" OR "genetically engineered" OR Cry* OR Vip*))	15.12.2014 28.1.2019 23.8.2019	(SCO)	3023	
JSTOR www.jstor.org	(nontarget OR "non target") AND (maize OR corn) AND (field& OR plot& OR location& OR trial& OR farm& OR trap& OR sampling OR	11.12.2014 23.8.2019	(JST)	554	Including external content, no limitations on content

	monitoring) AND (transgenic OR Bt OR "Bacillus thuringiensis" OR "genetically modified" OR "genetically engineered")				
Google Scholar www.scholar-google.com	(maize OR corn) AND (field OR plot OR location OR trial OR farm OR trap OR sampling OR monitoring) AND (transgenic OR Bt OR "Bacillus thuringiensis" OR "genetically modified" OR "genetically engineered") AND (nontarget OR "non target")	10.12.2014 5.8.2019	(GOO)	479	Patents and citations excluded, time range 1980-2014, first 300 exported in initial search and update

[a] numbers represent references combined from the initial search and updates after duplicates were eliminated using the find duplicates function in Endnote (author, title, year / title, year, pages).
[b] AGRIS was no longer available through Ovid SP when performing updates, so searches were conducted through USDA, National Agricultural Library Database, Navigator Database Platform (Digitop, USDA's Digital Library) using the search string (maize OR corn OR "Zea mays") AND (field* OR plot* OR location* OR trial* OR farm-scale OR scouting OR trap* OR sampl* OR monitor*) AND (transgenic OR Bt OR "Bacillus thuringiensis" OR GM OR "genetically modified" OR “genetically engineered" OR Cry* OR Vip*)

Table S1.2: Specialist searches on websites related to GMO risk assessment. Presented are the organization and hyperlink, the dates of the searches, specific settings or subpages used for the search, and the number of references (hits) exported to the bibliographic software. Documents found on several webpages are only listed once.

Organisation & hyperlink	Date	Search settings	Hits
European Food Safety Authority (EFSA) Register of Questions http://registerofquestions.efsa.europa.eu/roqFrontend/?wicket:interface=:3::::	10.12.2014 6.8.2019	Unit Filter = GMO, result: 449 entries.	47
Bundesamt für Umwelt (BAFU), Switzerland www.bafu.admin.ch/biotechnologie/01786/index.html?lang=de	6.2.2015 5.9.2019	Publications biotechnology	1
Bundesamt für Naturschutz (BfN), Germany https://www.bfn.de/themen/agro-gentechnik/veroeffentlichungen.html	6.2.2015 5.9.2019	BfN-Publikationen und weiterführende Literatur zum Thema Agro-Gentechnik	2
Bundesministerium für Gesundheit (BMG), Austria www.bmg.gv.at	6.2.2015 5.9.2019	Gesundheit – Gentechnik – grüne Gentechnik	10
CORDIS www.cordis.europa.eu	20.8.2015 6.8.2019	Project Publications, Report Summaries, Bt maize (11 entries), Bt corn (6), GM maize (31), GMP biodiversity (9)	2
GMO-Safety www.gmo-safety.eu	20.8.2015 6.8.2019	Database, research area maize, topic non-target organisms. 26 projects retrieved. 2019: database no longer existing	6
AMIGA Project www.amigaproject.eu	10.9.2015 6.8.2019	Documents: Publications & Literature 2019: Website under construction (not available)	1
Bibliosafety by ICGEB http://bibliosafety.icgeb.org	21.8.2015 6.8.2019	Risk category: Environment, search for Bt maize in abstract, result: 374 entries. Screened online. 2019: website not responding	3
Center for Environmental Risk Assessment (CERA) http://cera-gmc.org	15.12.2014 6.8.2019	CERA Publications, protein monographs, search for Bt maize, Bt corn, GM maize, GM corn	6
Testbiotech www.testbiotech.org/en	16/18.2.2015 6.8.2019	PlantGeneRisk Database, filter set: Insecticidal (trait), maize (plant); Reports and publications	11
ISAAA www.isaaa.org	8.8.2019	General search, Bt maize non-target, first 100 documents screened	2
Europabio www.europabio.org	2.2.2015 6.8.2019	Sector: agricultural biotech/publications	7
GM watch www.gmwatch.org	6.2.2015 6.8.2019	General search, keywords: biodiversity, Bt maize; section articles, subsection GM reports, subsection GM myths	1
Third World Network www.thirdworldnetwork.net; www.biosafety-info.net	2.2.2015 6.8.2019	Biotechnology/Biosafety, keyword search: Bt maize, Bt corn, non-target effect, biodiversity	7
Friends of the Earth www.foe.org	6.2.2015 8.8.2019	General search, keywords: Bt maize, Bt corn; filtered for food and agricultural issues	0
Greenpeace Research Laboratories www.greenpeace.to	6.2.2015 8.8.2019	General search (search publications), keywords: Bt maize, Bt corn	2
Greenpeace International www.greenpeace.org	6.2.2015 8.8.2019	General search, keywords: Bt maize, GM maize	2
