# Supplementary material for: Does the growing of Bt maize change abundance or ecological function of non-target animals compared to the growing of non-GM maize? A systematic review
Source: Environ Evid. 2022 Jun 6;11:21. doi: 10.1186/s13750-022-00272-0 (PMC11378853; doi:10.1186/s13750-022-00272-0)
Supplement: Supplementary file 2 — Additional file 2: References included in the full text screening. [file 13750_2022_272_MOESM2_ESM.pdf]

Does the growing of Bt maize change abundance or ecological function of non-target animals compared to the growing of non-GM maize? A systematic review

Michael Meissle[1*], Steven E. Naranjo[2], and Jörg Romeis[1]
[1]Agroscope, Research Division Agroecology and Environment, Reckenholzstrasse 191, 8046 Zurich, Switzerland, michael.meissle@agroscope.admin.ch; joerg.romeis@agroscope.admin.ch
[2]USDA-ARS, Arid-Land Agricultural Research Center, 21881 North Cardon Lane, Maricopa 85138, Arizona, USA, steve.naranjo@usda.gov

[*] Corresponding author

Published in: Environmental Evidence (2022), https://doi.org/10.1186/s13750-022-00272-0

Additional file 2: References included in the full text screening

References included in the quantitative database and/or the narrative summary tables. References were assigned a unique identifier (publication number). References used for the database are labelled with "**DB**". References used for the narrative summary tables are labelled with **N1-N5** according to the table number in Additional file 3.

The sources (bibliographic databases, full text databases, specialist search, personal contacts, reviews) for each reference are included. Databases are abbreviated as follows: WOS: Web of Science Core Collection; BIO: BIOSIS; ZOO: Zoological Record; SCI: SciELO; AOA: Agricola; AGS: AGRIS; CAB: CAB Abstracts; BAS: BASE; PRO: ProQuest; GOO: Google Scholar; JST: JSTOR; SCO: Scopus. See Additional file 1, Table S1.1 for details.

The following list of references is sorted by author(s) and year.

Ahmad A, Negri I, Oliveira W, Brown C, Asiimwe P, Sammons B, Horak M, Jiang CJ, Carson D: Transportable data from non-target arthropod field studies for the environmental risk assessment of genetically modified maize expressing an insecticidal double-stranded RNA. *Transgenic Research* 2016, 25(1):1-17.°**(659 DB) Source: WOS BIO ZOO AOA AGS CAB SCO**

Ahmad A, Wilde GE, Whitworth RJ, Zolnerowich G: Effect of corn hybrids expressing the coleopteran-specific Cry3Bb1 protein for corn rootworm control on aboveground insect predators. *Journal of Economic Entomology* 2006, 99(4):1085-1095.°**(246 DB) Source: WOS BIO ZOO AOA CAB GOO SCO**

Ahmad A, Wilde GE, Zhu KY: Detectability of coleopteran-specific Cry3Bb1 protein in soil and its effect on nontarget surface and below-ground arthropods. *Environmental Entomology* 2005, 34(2):385-394.°**(175 DB) Source: ZOO AOA CAB GOO SCO WOS BIO**

Albajes R, Farinos GP, Perez-Hedo M, de la Poza M, Lumbierres B, Ortego F, Pons X, Castanera P: Post-market environmental monitoring of Bt maize in Spain: Non-target effects of varieties derived from the event MON810 on predatory fauna. *Spanish Journal of Agricultural Research* 2012, 10(4):977-985.°**(646 DB) Source: WOS BIO ZOO CAB SCO BAS**

Alcantara EP: Postcommercialization Monitoring of the Long-Term Impact of Bt Corn on Non-Target Arthropod Communities in Commercial Farms and Adjacent Riparian Areas in the Philippines. *Environmental Entomology* 2012, 41(5):1268-1276.°**(601 DB N2 N4) Source: WOS BIO ZOO CAB SCO**

Alcantara EP, Caoili BL, Javier PA, Mostoles MDJ: A multilayer, large scale comparison of arthropod communities in commercially managed Bt and non-Bt corn fields. *Philippine Entomologist* 2010, 24(2):150-164.°**(632 DB N4) Source: BIO ZOO CAB**

Al-Deeb MA, Wilde GE: Effect of Bt corn expressing the Cry3Bb1 toxin for corn rootworm control on aboveground nontarget arthropods. *Environmental Entomology* 2003, 32(5):1164-1170.°**(88 DB) Source: WOS BIO ZOO AOA CAB GOO SCO**

Al-Deeb MA, Wilde GE, Blair JM, Todd TC: Effect of Bt corn for corn rootworm control on nontarget soil microarthropods and nematodes. *Environmental Entomology* 2003, 32(4):859-865.°**(90 DB N5) Source: WOS BIO ZOO CAB GOO SCO**

Al-Deeb MA, Wilde GE, Higgins RA: No effect of Bacillus thuringiensis corn and Bacillus thuringiensis on the predator Orius insidiosus (Hemiptera : Anthocoridae). *Environmental Entomology* 2001, 30(3):625-629.°**(83 DB) Source: WOS BIO AOA GOO SCO**

Arias-Martin M, Garcia M, Castanera P, Ortego F, Farinos GP: Farm-scale evaluation of the impact of Cry1Ab Bt maize on canopy nontarget arthropods: a 3-year study. *Insect Science* 2018, 25(1):87-98.°**(658 DB) Source: WOS BIO ZOO AGR SCO**

Arias-Martin M, Garcia M, Lucianez MJ, Ortego F, Castanera P, Farinos GP: Effects of three-year cultivation of Cry1Ab-expressing Bt maize on soil microarthropod communities. *Agriculture Ecosystems & Environment* 2016, 220:125-134.°**(657 DB N4) Source: WOS BIO ZOO CAB AGR SCO**

Arpas K, Toth F, Kiss J: Foliage-dwelling arthropods in Bt-transgenic and isogenic maize: A comparison through spider web analysis. *Acta Phytopathologica et Entomologica Hungarica* 2005, 40(3-4):347-353. **(208 DB) Source: BIO ZOO CAB SCO**

Balog A, Kiss J, Szekeres D, Szenasi A, Marko V: Rove beetle (Coleoptera: Staphylinidae) communities in transgenic Bt (MON810) and near isogenic maize. *Crop Protection* 2010, 29(6):567-571.°**(521 DB N4) Source: WOS BIO ZOO AOA CAB GOO SCO**

Balog A, Szenasi A, Szekeres D, Palinkas Z: Analysis of soil dwelling rove beetles (Coleoptera: Staphylinidae) in cultivated maize fields containing the Bt toxins, Cry34/35Ab1 and Cry1F x Cry34/35Ab1. *Biocontrol Science and Technology* 2011, 21(3):293-297.°**(524 DB) Source: WOS BIO ZOO SCO AOA CAB AGS**

Benker U, Priesnitz KU: Effects of Bt maize containing three Bt proteins on ground beetles and spiders [http://www.gmo-safety.eu/database/1037.effects-maize-containing-three-proteins-ground-beetles-spiders.html]°**(649 DB) Source: Specialist Search (GMO-safety.eu)**

Bhatti MA, Duan J, Head G, Jiang CJ, McKee MJ, Nickson TE, Pilcher CL, Pilcher CD: Field evaluation of the impact of corn rootworm (Coleoptera : Chrysomelidae)-protected Bt corn on ground-dwelling invertebrates. *Environmental Entomology* 2005, 34(5):1325-1335.°**(166 DB) Source: WOS BIO ZOO AOA CAB GOO SCO**

Bhatti MA, Duan J, Head GP, Jiang CJ, McKee MJ, Nickson TE, Pilcher CL, Pilcher CD: Field evaluation of the impact of corn rootworm (Coleoptera : Chrysomelidae)-protected Bt corn on foliage-dwelling arthropods. *Environmental Entomology* 2005, 34(5):1336-1345.°**(167 DB) Source: WOS BIO ZOO AOA CAB GOO SCO**

Bhatti MA, Duan J, Pilcher CL, Pilcher CD, McKee MJ, Nickson TE, Head GP, Jiang C: Ecological assessment for non-target organisms in the plots of corn rootworm insect-protected corn hybrid containing MON 863 event: 2000-2001 field trials. Monsanto Company; 2002.°**(144 DB) Source: Personal contact**

Bitzer RJ, Rice ME, Pilcher CD, Pilcher CL, Lam WKF: Biodiversity and community structure of epedaphic and euedaphic springtails (Collembola) in transgenic rootworm Bt corn. *Environmental Entomology* 2005, 34(5):1346-1376.°**(171 DB N4) Source: WOS BIO ZOO CAB GOO SCO AOA**

Bohnenblust E, Breining J, Roth G, Tooker J: Corn pith weevil, Geraeus penicillus (Coleoptera: Curculionidae), in transgenic, insect-resistant and conventional field corn hybrids. *Journal of Applied Entomology* 2013, 137(9):668-672.°**(602 DB) Source: WOS BIO ZOO CAB SCO**

Bourguet D, Chaufaux J, Micoud A, Delos M, Naibo B, Bombarde F, Marque G, Eychenne N, Pagliari C: Ostrinia nubilalis parasitism and the field abundance of non-target insects in transgenic Bacillus thuringiensis corn (Zea mays). *Environmental Biosafety Research* 2002, 1:49-60.°**(38 DB) Source: BIO ZOO CAB GOO SCO**

Bruck DJ, Lopez MD, Lewis LC, Prasifka JR, Gunnarson RD: Effects of transgenic Bacillus thuringiensis corn and permethrin on nontarget arthropods. *Journal of Agricultural and Urban Entomology* 2006, 23(3):111-124.°**(516 DB) Source: WOS BIO ZOO CAB SCO**

Büchs W: Research into the impact of Bt maize (Cry 3Bb1) on non-target organisms living in the soil [http://www.gmo-safety.eu/database/1028.research-impact-maize-cry-3bb1-non-target-organisms-living-soil.html]°**(901 N5) Source: Specialist Search (GMO-safety.eu)**

Campos RC, Hernandez MIM: The Importance of Maize Management on Dung Beetle Communities in Atlantic Forest Fragments. *Plos One* 2015, 10(12):15.°**(958 N1 N2) Source: WOS BIO ZOO CAB SCO**

Campos RC, Hernández MIM: Changes in the dynamics of functional groups in communities of dung beetles in Atlantic forest fragments adjacent to transgenic maize crops. *Ecological Indicators* 2014, 49:216-227.°**(946 N2) Source: SCO**

Candolfi MP, Brown K, Grimm C, Reber B, Schmidli H: A faunistic approach to assess potential side-effects of genetically modified Bt-corn on non-target arthropods under field conditions. *Biocontrol Science and Technology* 2004, 14(2):129-170.°**(603 DB N4) Source: WOS AOA CAB GOO SCO**

Carter ME, Villani MG, Allee LL, Losey JE: Absence of non-target effects of two Bacillus thuringiensis coleopteran active delta-endotoxins on the bulb mite, Rhizoglypus robini (Claparede) (Acari, Acaridae). *Journal of Applied Entomology* 2004, 128(1):56-63.°**(609 DB) Source: WOS BIO ZOO CAB SCO**

Cerevkova A, Cagan L: Effect of transgenic insect-resistant maize to the community structure of soil nematodes in two field trials. *Helminthologia* 2015, 52(1):41-49. **(653 DB N4) Source: Specialist Search (AMIGA project website)**

Cerevkova A, Miklisova D, Szoboszlay M, Tebbe CC, Cagan L: The responses of soil nematode communities to Bt maize cultivation at four field sites across Europe. *Soil Biology & Biochemistry* 2018, 119:194-202.°**(660 DB N4) Source: WOS BIO ZOO CAB AGR SCO**

Chambers CP, Whiles MR, Rosi-Marshall EJ, Tank JL, Royer TV, Griffiths NA, Evans-White MA, Stojak AR: Responses of

stream macroinvertebrates to Bt maize leaf detritus. *Ecological Applications* 2010, 20(7):1949-1960.°**(947 N2) Source: WOS BIO ZOO CAB JST SCO**

Coman D: Study regarding useful fauna on corn plants. *Scientific Papers Series A, Agronomy* 2012, 55:133-136.°**(933 N5) Source: CAB**

Coman D, Rosca I: Researches regarding evolution of speciae Chrysoperla carnea - Neuroptera fauna in corn agro ecosystem. *Scientific Papers Series A, Agronomy* 2013, 56:480-483.°**(934 N5) Source: CAB**

Coman D, Rosca I: Structure, dynamics and abundance of carabid species collected in corn fields. *Scientific Papers Series A, Agronomy* 2013, 56:477-479.°**(935 N4 N5) Source: CAB**

Comas C, Lumbierres B, Pons X, Albajes R: No effects of Bacillus thuringiensis maize on nontarget organisms in the field in southern Europe: a meta-analysis of 26 arthropod taxa. *Transgenic Research* 2014, 23(1):135-143.°**(902 N5) Source: WOS BIO AOA CAB SCO AGS**

Cortet J, Griffiths BS, Bohanec M, Demsar D, Andersen MN, Caul S, Birch ANE, Pernin C, Tabone E, de Vaufleury A *et al*: Evaluation of effects of transgenic Bt maize on microarthropods in a European multi-site experiment. *Pedobiologia* 2007, 51(3):207-218.°**(504 DB) Source: WOS BIO ZOO SCO CAB GOO**

Crosariol Netto J: Infestação e danos de Dichelops melacanthus (Dallas, 1851) (Heteroptera: Pentatomidae) em híbridos transgênicos e convencionais de milho, submetidos ao controle químico. Universidade Estadual Paulista (UNESP), São Paulo, Brasil; 2013.°**(932 N5) Source: BAS**

Daly T, Buntin GD: Effect of Bacillus thuringiensis transgenic corn for lepidopteran control on nontarget arthropods. *Environmental Entomology* 2005, 34(5):1292-1301.°**(169 DB) Source: WOS BIO ZOO AOA CAB GOO SCO**

de Araujo LF, da Silva AG, Cruz I, do Carmo EL, Horvath Neto A, Goulart MMP, Rattes JF: Population dynamics of Spodoptera frugiperda (J. E. Smith), Diatraea saccharalis (Fabricius) and Doru luteipes (Scudder) in conventional and Bt transgenic maize. *Revista Brasileira de Milho e Sorgo* 2011, 10(3):205-214.°**(911 N5) Source: CAB BAS**

de la Poza Gómez M: Maíz Bt: Seguimiento de la resistencia de Sesamia nonagrioides (Lepidoptera: Noctuidae) y Ostrinia nubilalis (Lepidoptera: Crambidae) y efectos en artrópodos depredadores. Madrid: Universidad Politécnica de Madrid; 2004.°**(672 DB) Source: Reference list of review "Anonymous 2010: Planes de seguimiento ambiental del cultivo de maíz modificado genéticamente en España."**

de la Poza M: Potential effects of Bt11 x MIR604 x GA21 maize on non-target arthropods - Spain. Syngenta; 2010: 79 pages.°**(676 DB) Source: Specialist Search (EFSA)**

de la Poza M, Pons X, Farinos GP, Lopez C, Ortego F, Eizaguirre M, Castanera P, Albajes R: Impact of farm-scale Bt maize on abundance of predatory arthropods in Spain. *Crop Protection* 2005, 24(7):677-684.°**(182 DB) Source: WOS BIO ZOO AOA CAB GOO SCO**

Debeljak M, Cortet J, Demsar D, Krogh PH, Dzeroski S: Hierarchical classification of environmental factors and agricultural practices affecting soil fauna under cropping systems using Bt maize. *Pedobiologia* 2007, 51(3):229-238.°**(508 DB) Source: WOS BIO ZOO SCO CAB**

Delrio G, Verdinelli M, Serra G: Monitoring of pest and beneficial insect populations in summer sown Bt maize. *IOBC-WPRS Bulletin* 2004, 27(3):43-48.°**(230 DB) Source: ZOO CAB**

Di Grumo D, Lovei GL: Body size inequality in ground beetle (Coleoptera: Carabidae) assemblages as a potential method to monitor environmental impacts of transgenic crops. *Periodicum Biologorum* 2016, 118(3):223-230.°**(669 DB N4) Source: WOS BIO ZOO CAB SCO**

DiTommaso A, Ryan MR, Mohler CL, Brainard DC, Shuler RE, Allee LL, Losey JE: Effect of Cry3Bb Bt Corn and Tefluthrin on Postdispersal Weed Seed Predation. *Weed Science* 2014, 62(4):619-624.°**(912 N5) Source: WOS BIO CAB AGR SCO AGS JST**

Dively G: Field Scale Evaluation of Effects of Agrisure RW Rootworm-Protected (Event MIR604) Corn on Non-Target Coleoptera and Other Beneficial Arthropods. Syngenta; 2010: 26 pages.°**(678 DB) Source: Specialist Search (EFSA)**

Dively GP: Impact of Transgenic VIP3A x Cry1Ab Lepidopteran-resistant Field Corn on the Nontarget Arthropod Community. *Environmental Entomology* 2005, 34(5):1267-1291.°**(170 DB N4) Source: WOS BIO ZOO AOA CAB SCO GOO**

Dowd PF: Dusky sap beetles (Coleoptera : Nitidulidae) and other kernel damaging insects in Bt and non-Bt sweet corn in Illinois. *Journal of Economic Entomology* 2000, 93(6):1714-1720.°**(86 DB) Source: WOS BIO ZOO AOA CAB SCO BAS**

Dowd PF: Indirect reduction of ear molds and associated mycotoxins in Bacillus thuringiensis corn under controlled and open field conditions: Utility and limitations. *Journal of Economic Entomology* 2000, 93(6):1669-1679.°**(900 N5) Source: WOS BIO AOA CAB GOO SCO**

Dowd PF: Suitability of commercially available insect traps and pheromones for monitoring dusky sap beetles (Coleoptera : Nitidulidae) and related insects in Bt sweet corn. *Journal of Economic Entomology* 2005, 98(3):856-861.°**(919 N5) Source: WOS BIO ZOO AOA CAB SCO BAS**

Eckert J: Effekte des Anbaus von Bt-Mais auf Nichtzielarthropoden der Krautschichtfauna : Monitoringorganismen und praktikable Erfassungsmethoden ; Effects of the Cultivation of Bt-maize on nontarget Arthropods of the Herblayer Fauna : Monitoringorganisms and suitable Methods. *PhD*, RWTH Aachen University; 2006.°**(600 DB) Source: BAS**

Eckert J, Schuphan I, Hothorn LA, Gathmann A: Arthropods on maize ears for detecting impacts of Bt maize on nontarget organisms. *Environmental Entomology* 2006, 35(2):554-560.°**(213 DB) Source: WOS BIO ZOO AOA CAB GOO SCO**

El-Wakeil N, Volkmar C: Monitoring of key non-target arthropods in insect-resistant transgenic maize lines during 2010 and 2011 in field studies in central Germany. *IOBC/WPRS Bulletin* 2013, 97:31-48.°**(611 DB) Source: CAB**

Fan CM, Wu FC, Dong JY, Wang BF, Yin JQ, Song XY: No impact of transgenic cry1Ie maize on the diversity, abundance and composition of soil fauna in a 2-year field trial. *Scientific Reports* 2019, 9:10333.°**(966 N4 N5) Source: WOS BIO SCO**

Farinos GP, de la Poza M, Hernandez-Crespo P, Ortego F, Castanera P: Diversity and seasonal phenology of aboveground arthropods in conventional and transgenic maize crops in Central Spain. *Biological Control* 2008, 44(3):362-371.°**(514 DB N4) Source: WOS BIO ZOO AOA CAB GOO SCO BAS**

Fernandes OA, Faria M, Martinelli S, Schmidt F, Carvalho VF, Moro G: Short-term assessment of Bt maize on non-target arthropods in Brazil. *Scientia Agricola* 2007, 64(3):249-255.°**(507 DB) Source: WOS SCI CAB GOO SCO BAS**

Ferrante M, Lövei GL, Magagnoli S, Minarcikova L, Tomescu EL, Burgio G, Cagan L, Ichim MC: Predation pressure in maize across Europe and in Argentina: An intercontinental comparison. *Insect Science* 2017,26(3):545-554.°**(668 DB) Source: SCO AOA SCO WOS ZOO**

Floate KD, Carcamo HA, Blackshaw RE, Postman B, Bourassa S: Response of ground beetle (Coleoptera : Carabidae) field Populations to four years of Lepidoptera-Specific Bt corn production. *Environmental Entomology* 2007, 36(5):1269-1274.°**(615 DB) Source: WOS BIO ZOO AOA CAB GOO SCO**

Frouz J, Elhottova D, Helingerova M, Kocourek F: The Effect of Bt-corn on Soil Invertebrates, Soil Microbial Community and Decomposition Rates of Corn Post-Harvest Residues Under Field and Laboratory Conditions. *Journal of Sustainable Agriculture* 2008, 32(4):645-655.°**(624 DB) Source: WOS AOA CAB SCO**

Gathmann A, Wirooks L, Hothorn LA, Bartsch D, Schuphan I: Impact of Bt maize pollen (MON810) on lepidopteran larvae living on accompanying weeds. *Molecular Ecology* 2006, 15(9):2677-2685.°**(945 N2) Source: WOS BIO ZOO AOA CAB GOO SCO**

Griffiths BS, Caul S, Thompson J, Birch ANE, Cortet J, Andersen MN, Krogh PH: Microbial and microfaunal community structure in cropping systems with genetically modified plants. *Pedobiologia* 2007, 51(3):195-206. **(914 N5) Source: WOS BIO ZOO SCO CAB**

Griffiths BS, Caul S, Thompson J, Birch ANE, Scrimgeour C, Andersen MN, Cortet J, Messean A, Sausse C, Lacroix B *et al*: A comparison of soil microbial community structure, protozoa and nematodes in field plots of conventional and genetically modified maize expressing the Bacillus thuringiens is CryIAb toxin. *Plant and Soil* 2005, 275(1-2):135-146.°**(913 N5) Source: WOS BIO AOA CAB GOO SCO JST**

Grozea I, Stef R, Carabet A, Virteiu AM, Badea M, Molnar L, Cotuna O: Preliminary studies regarding the useful and pest animal species from different variants of maize in Timis, Calarasi and Braila counties. *Research Journal of Agricultural Science* 2009, 41(1):228-234.°**(625 DB) Source: CAB**

Gullu M, Tatli F, Kanat AD, Islamoglu M: Population development of some predatory insects on Bt and non-Bt maize hybrids in Turkey. *IOBC-WPRS Bulletin* 2004, 27(3):85-91.°**(621 DB) Source: ZOO CAB**

Guo J, Zhang C, Yuan Z, He K, Wang Z: Impacts of transgenic corn with cry1Ie gene on arthropod biodiversity in the fields. *Acta Phytophylacica Sinica* 2014, 41(4):482-489.°**(953 N4) Source: CAB**

Guo JF, He KL, Bai SX, Zhang TT, Liu YJ, Wang FX, Wang ZY: Effects of transgenic cry1Ie maize on non-lepidopteran pest abundance, diversity and community composition. *Transgenic Research* 2016, 25(6):761-772.°**(670 DB N4) Source: WOS BIO CAB AGR SCO**

Guo JF, He KL, Hellmich RL, Bai SX, Zhang TT, Liu YJ, Ahmed T, Wang ZY: Field trials to evaluate the effects of transgenic cry1Ie maize on the community characteristics of arthropod natural enemies. *Scientific Reports* 2016, 6:12.°**(671 DB N4) Source: WOS BIO ZOO CAB SCO**

Guo Y, Feng Y, Ge Y, Tetreau G, Chen X, Dong X, Shi W: The cultivation of Bt corn producing Cry1Ac toxins does not adversely affect non-target arthropods. *Plos One* 2014, 9(12).°**(626 DB N4) Source: SCO**

Habustova O, Dolezal P, Spitzer L, Svobodova Z, Hussein H, Sehnal F: Impact of Cry1Ab toxin expression on the non- target insects dwelling on maize plants. *Journal of Applied Entomology* 2014, 138(3):164-172.°**(604 DB N4) Source: WOS BIO ZOO CAB SCO AOA AGS**

Hassell RL, Shepard BM: Insect populations on Bacillus thuringiensis transgenic sweet corn. *Journal of Entomological Science* 2002, 37(4):285-292.°**(910 N5) Source: BIO**

Hernandez-Juarez A, Aguirre LA, Cerna E, Flores M, Frias GA, Landeros J, Ochoa YM: Abundance of non-target predators in genetically modified corn. *Florida Entomologist* 2019, 100(1):96-100.°**(963 N5) Source: SCO WOS BIO ZOO**

Hernandez-Juarez A, Aguirre LA, Cerna E, Landeros J, Fries GA, Flores M, Ochoa YM: Effect of Transgenic Maize on Abundance of the Corn Flea Beetle, Chaetocnema pulicaria Melsheimer, as a Non-Target Pest. *Southwestern Entomologist* 2018, 43(4):841-846.°**(961 N5) Source: WOS BIO SCO**

Higgins LS: Field monitoring of Bt maize for non-target organism effects. *Ph.D.* Ann Arbor: The University of Nebraska - Lincoln, USA; 2011.°**(952 N4 N5) Source: PRO BAS**

Higgins LS, Babcock J, Neese P, Layton RJ, Moellenbeck DJ, Storer N: Three-year field monitoring of Cry1F, event DAS-01507-1, maize hybrids for nontarget arthropod effects. *Environmental Entomology* 2009, 38(1):281-292.°**(527 DB N4) Source: ZOO AOA WOS BIO CAB GOO SCO**

Hoheisel GA, Fleischer SJ: Coccinellids, aphids, and pollen in diversified vegetable fields with transgenic and isoline cultivars. *Journal of Insect Science* 2007, 7.°**(505 N3) Source: WOS CAB BAS SCO BIO AOA**

Höss S, Nguyen HT, Menzel R, Pagel-Wieder S, Miethling-Graf R, Tebbe CC, Jehle JA, Traunspurger W: Assessing the risk posed to free-living soil nematodes by a genetically modified maize expressing the insecticidal Cry3Bb1 protein. *Science of the Total Environment* 2011, 409(13):2674-2684.°**(645 DB N4) Source: WOS BIO CAB SCO AOA**

Höss S, Reiff N, Ottermanns R, Pagel-Wieder S, Dohrmann AB, Tebbe CC, Traunspurger W: Risk assessment of the cultivation of a stacked Bt-maize variety (MON89034 x MON88017) for nematode communities. *Soil Biology & Biochemistry* 2015, 91:109-118.°**(656 DB N4) Source: WOS BIO ZOO CAB**

Hurej M, Twardowski JP, Beres P, Klukowski Z: Population development of bird cherry-oat aphid Rhopalosiphum padi (L.) (Hemiptera, Aphididae) on conventional and Bt-maize expressing the insecticidal protein Cry1Ab. *Bulgarian Journal of Agricultural Science* 2014, 20(3):658-665.°**(667 DB) Source: CAB SCO**

Jasinski JR, Eisley JB, Young CE, Kovach J, Willson H: Select nontarget arthropod abundance in transgenic and nontransgenic field crops in Ohio. *Environmental Entomology* 2003, 32(2):407-413.°**(28 DB) Source: WOS BIO ZOO AOA CAB GOO SCO**

Jesse LCH, Obrycki JJ: Occurrence of Danaus plexippus L. (Lepidoptera : Danaidae) on milkweeds (Asclepias syriaca) in transgenic Bt corn agroecosystems. *Agriculture Ecosystems & Environment* 2003, 97(1-3):225-233.°**(942 N2) Source: WOS BIO ZOO CAB SCO**

Kamota A, Muchaonyerwa P, Mnkeni PNS: Effects of transgenic maize expressing the Cry1Ab protein (event MON810) on locally adapted earthworms in a sandy loam soil in the Central Eastern Cape, South Africa. *African Journal of Biotechnology* 2012, 11(85):15168-15170.°**(905 N5) Source: Personal contact**

Kennard KS: The Effects of Prey Abundance and Bt (Bacillus thuringiensis) Crops on Bat Activity in South-Central Texas Agroecosystems. *M.Sc.*The University of Tennessee, Knoxville, USA; 2008.°**(950 N1) Source: BAS**

Kocourek F, Saska P, Rezac M: Diversity of Carabid Beetles (Coleoptera: Carabidae) under Three Different Control Strategies against European Corn Borer in Maize. *Plant Protection Science* 2013, 49(3):146-153.°**(643 DB) Source: WOS BIO ZOO CAB SCO AGS**

Krogh PH, Griffiths B, Demsar D, Bohanec M, Debeljak M, Andersen MN, Sausse C, Birch ANE, Caul S, Holmstrup M *et al*: Responses by earthworms to reduced tillage in herbicide tolerant maize and Bt maize cropping systems. *Pedobiologia* 2007, 51(3):219-227.°**(636 DB) Source: WOS BIO ZOO SCO CAB**

Kryger AM: Behavioral Response of White-Tailed Deer to Bacillus Thuringiensis Maize. *Ph.D.* Western Michigan University, USA; 2009.°**(951 N1) Source: BAS**

Lang A: Monitoring the impact of Bt maize on butterflies in the field: estimation of required sample sizes. *Environmental Biosafety Research* 2004, 3(1):55-66.°**(941 N2) Source: BIO ZOO CAB GOO SCO**

Lang A: Verbundprojekt: Sicherheitsforschung und Monitoringmethoden zum Anbau von Bt-Mais, Teilprojekt Effekte von Bt-Mais auf flugfähige Blütenbesucher und Prädatoren höherer Straten. Schlussbericht zum BMBF-Forschungsvorhaben; 2004: 89 pages.°**(650 DB) Source: Specialist Search (GMO-safety.eu)**

Lang A, Arndt M, Beck R, Bauchhenss J, Pommer G: Monitoring der Umweltwirkungen des Bt-Gens. In: *Schriftenreihe, Bayerische Landesanstalt für Landwirtschaft* vol. 7; 2005: 113 pages.°**(225 DB N5) Source: Previous database by Marvier et al. (2007)**

Lauer KF, Graepel H: Potential effects of Bt11 x MIR604 x GA21 maize on non-target arthropods - Romania. Syngenta; 2010: 79 pages.°**(677 DB N5) Source: Specialist Search (EFSA)**

Leslie TW, Biddinger DJ, Mullin CA, Fleischer SJ: Carabidae Population Dynamics and Temporal Partitioning: Response to Coupled Neonicotinoid-transgenic Technologies in Maize. *Environmental Entomology* 2009, 38(3):935-943.°**(642 DB N3) Source: WOS BIO ZOO AOA CAB SCO**

Leslie TW, Biddinger DJ, Rohr JR, Fleischer SJ: Conventional and Seed-Based Insect Management Strategies Similarly Influence Nontarget Coleopteran Communities in Maize. *Environmental Entomology* 2010, 39(6):2045-2055.°**(948 N3 N4) Source: WOS BIO ZOO CAB SCO**

Leslie TW, Hoheisel GA, Biddinger DJ, Rohr JR, Fleischer SJ: Transgenes sustain epigeal insect biodiversity in diversified vegetable farm systems. *Environmental Entomology* 2007, 36(1):234-244.°**(949 N3) Source: WOS BIO ZOO AOA CAB GOO SCO**

Lit IL, Jr., Caasi-Lit MT, Abenis KO, Manalo NAQ, Larona AR, Eusebio OL, Benigno EA: Non-target organisms on Bt corn hybrids MON89034 and MON89034/NK603: part 3. Functional guilds of arthropods in regulated field trial sites during the wet season in Luzon and Mindanao, Philippines. *Philippine Entomologist* 2016, 30(1):29-51.°**(955 N6) Source: BIO ZOO CAB**

Lit IL, Jr., Caasi-Lit MT, Benigno EA, Ramal AFB, Yap SA, Sapin GD, Eusebio OL, Larona AR: Non-target organisms on Bt corn hybrids MON89034 and MON89034/NK603: Part 2. Functional guilds of arthropods in regulated field trial sites during dry season in Luzon and Mindanao, Philippines. *Philippine Entomologist* 2012, 26(1):28-53.°**(904 N4 N5) Source: BIO ZOO CAB**

Lit IL, Jr., Caasi-Lit MT, Ramal AFB, Yap SA, Sapin GD, Eusebio OL, Larona AR: Non-target organisms on Bt corn MON89034 and MON89034/NK603: Part 1. Abundance and diversity of canopy- and ground surface-dwelling arthropods in regulated field trial sites during dry season in Luzon and Mindanao, Philippines. *Philippine Entomologist* 2011, 25(2):131-155.°**(968 N4 N5) Source: BIO ZOO CAB**

Liu X, Wang B, Wang J, Feng S, Song X: Effects of leaf residue decomposition of cry1Ac-transgenic insect-resistant maize on community structure of soil animals. *Acta Phytophylacica Sinica* 2016, 43(3):384-390.°**(665 DB N4) Source: CAB**

Lopez MD, Prasifka JR, Bruck DJ, Lewis LC: Utility of Ground Beetle Species in Field Tests of Potential Nontarget Effects of Bt Crops. *Environmental Entomology* 2005, 34(5):1317-1324.°**(168 DB) Source: WOS BIO ZOO AOA CAB GOO SCO BAS**

Lozzia GC: Biodiversity and structure of ground beetle assemblages (Coleoptera Carabidae) in Bt corn and its effects on non

target insects. *Bollettino di Zoologia Agraria e di Bachicoltura* 1999, 31(1):37-50.°**(52 DB N5) Source: ZOO CAB GOO BIO**
Lozzia GC, Rigamonti IE: Preliminary study on the effects of transgenic maize on non target species. *Bulletin OILB/SROP* 1998, 21(8):171-180.°**(620 DB) Source: CAB**
Ludy C, Lang A: A 3-year field-scale monitoring of foliage-dwelling spiders (Araneae) in transgenic Bt maize fields and adjacent field margins. *Biological Control* 2006, 38(3):314-324.°**(244 DB N2 N4) Source: WOS BIO ZOO AOA CAB SCO GOO**
Lumbierres B, Stary P, Pons X: Effect of Bt maize on the plant-aphid-parasitoid tritrophic relationships. *Biocontrol* 2011, 56(2):133-143.°**(630 DB) Source: WOS BIO ZOO AOA CAB SCO**
Ma Y, He H, Shen W, Liu B, Xue K: Effects of transgenic maize on arthropod diversity. *Biodiversity Science* 2019, 27(4):419-432.°**(673 DB N4) Source: CAB**
Madrid JLC, Carrillo JLM, Martinez MBO, Pompa HAD, Escobedo JA, Quinones FJ, Tiznado JA, Espinoza LC, Garcia FZ, Banda AE *et al*: Transportability of non-target arthropod field data for the use in environmental risk assessment of genetically modified maize in Northern Mexico. *Journal of Applied Entomology* 2018, 142(5):525-538.°**(675 DB N3) Source: WOS BIO ZOO CAB AGR SCO**
Manachini B: Ground beetle assemblages (Coleoptera, Carabidae) and plant dwelling non-target arthropods in isogenic and transgenic corn crops. *Bollettino di Zoologia Agraria e di Bachicoltura* 2000, 32(3):181-198.°**(616 DB) Source: BIO CAB GOO**
Manachini B: Effects of transgenic corn on Lydella thompsoni Herting (Diptera Tachinidae) parasitoid of Ostrinia nubilalis Hb. (Lepidoptera Crambidae). *Bollettino di Zoologia Agraria e di Bachicoltura* 2003, 35(2):111-125.°**(617 DB) Source: BIO ZOO CAB**
Manachini B, Lozzia GC: First investigations into the effects of Bt corn crop on Nematofauna. *Bollettino di Zoologia Agraria e di Bachicoltura* 2002, 34(1):85-96.°**(619 DB N4) Source: BIO ZOO CAB**
Manachini B, Lozzia GC: Studies on the effects of Bt corn expressing Cry1Ab on two parasitoids of Ostrinia nubilalis Hb. (Lepidoptera: Crambidae). *IOBC-WPRS Bulletin* 2004, 27(3):109-116.°**(618 DB) Source: ZOO CAB**
McManus BL, Fuller BW, Boetel MA, French BW, Ellsbury MM, Head GP: Abundance of Coleomegilla maculata (Coleoptera: Coccinellidae) in corn rootworm-resistant Cry3Bb1 maize. *Journal of Economic Entomology* 2005, 98(6):1992-1998.°**(906 N5) Source: WOS BIO ZOO AOA CAB SCO**
Meissle M, Lang A: Comparing methods to evaluate the effects of Bt maize and insecticide on spider assemblages. *Agriculture Ecosystems & Environment* 2005, 107(4):359-370.°**(215 DB) Source: WOS BIO ZOO CAB SCO AOA GOO**
Minarcikova L, Cagan L, Kovac L: Influence of Bt maize to epigeic collembolan communities. *IOBC/WPRS Bulletin* 2013, 97:89-96.°**(651 DB) Source: CAB**
Mücher T: Untersuchung möglicher Effekte von transgenem, insektenresistentem Mais (Bt-Mais) auf Nichtzielorganismen im Boden, sowie Analyse und Bewertung von Beifußpopulationen (Artemisia vulgaris L.) des Maiszünslers (Ostrinia nubilalis Hbn.) als Mittel zur Resistenzverzögerung. *Ph.D.* RWTH Aachen University; 2004.°**(622 DB) Source: BAS**
Musser FR, Shelton AM: Bt sweet corn and selective insecticides: Impacts on pests and predators. *Journal of Economic Entomology* 2003, 96(1):71-80.°**(31 DB) Source: WOS BIO ZOO AOA CAB GOO SCO**
Naïbo B, Marque G: Contrairement à un insecticide conventionnel, le maïs Bt préserve la faune utile. *Perspectives Agricoles* 2003, 293:14-17.°**(903 N5) Source: Reference in Review (Ortego et al. 2009)**
Neher DA, Muthumbi AWN, Dively GP: Impact of coleopteran-active Bt corn on non-target nematode communities in soil and decomposing corn roots. *Soil Biology & Biochemistry* 2014, 76:127-135.°**(635 DB N4) Source: WOS BIO ZOO**
Novillo C, Fernandez-Anero FJ, Costa J: Performance of insect-protected corn varieties derived from Bt line MON 810, in Spain. *Boletin de Sanidad Vegetal, Plagas* 2003, 29(3):427-439.°**(633 DB) Source: CAB**
Orr DB, Landis DA: Oviposition of European corn borer (Lepidoptera:Pyralidae) and impact of natural enemy populations in transgenic versus isogenic corn. *Journal of Economic Entomology* 1997, 90(4):905-909.°**(4 DB) Source: WOS BIO ZOO AOA AGS CAB SCO**
Palinkas Z, Kiss J, Zalai M, Szenasi A, Dorner Z, North S, Woodward G, Balog A: Effects of genetically modified maize events expressing Cry34Ab1, Cry35Ab1, Cry1F, and CP4 EPSPS proteins on arthropod complex food webs. *Ecology and Evolution* 2017, 7(7):2286-2293.°**(960 N4) Source: WOS BIO ZOO CAB BAS SCO**
Palizada SA, Tiroesele B, Kondidie DB, Ullah MI, Mustafa F, Hunt TE, Clark PL, Molina-Ochoa J, Skoda SR, Foster JE: Minute pirate bug (Orius insidiosus Say) populations on transgenic and non-transgenic maize using different sampling techniques. *Pakistan Entomologist* 2014, 36(1):1-6.°**(908 N5) Source: CAB**
Pilcher CD, Obrycki JJ, Rice ME, Lewis LC: Preimaginal development, survival, and field abundance of insect predators on transgenic Bacillus thuringiensis corn. *Environmental Entomology* 1997, 26(2):446-454.°**(10 DB) Source: WOS ZOO AOA AGS CAB GOO SCO BIO**
Pilcher CD: Phenological, physiological, and ecological influences of transgenic Bt corn on European corn borer management. *Ph.D.* Iowa State University, USA; 1999.°**(173 DB) Source: Reference in review (Obrycki et al. 2004)**
Pons X, Lumbierres B, Lopez C, Albajes R: Abundance of non-target pests in transgenic Bt-maize: A farm scale study. *European Journal of Entomology* 2005, 102(1):73-79.°**(214 DB) Source: WOS BIO ZOO CAB SCO GOO AGS**
Priesnitz KU: Potential impact of Diabrotica resistant Bt-maize expressing Cry3Bb1 on ground beetles (Coleoptera: Carabidae). *Ph.D.* RWTH Aachen University, Germany; 2010.°**(610 DB N4) Source: BAS**
Priestley AL, Brownbridge M: Field trials to evaluate effects of Bt-transgenic silage corn expressing the Cry1Ab insecticidal

toxin on non-target soil arthropods in northern New England, USA. *Transgenic Research* 2009, 18(3):425-443.°**(916 N4 N5) Source: WOS BIO AOA CAB GOO SCO**

Puza V, Habustova O, Hussein HM, Svobodova Z: The abundance, distribution and natural host range of entomopathogenic nematodes (Nematoda: Steinernematidae) in the experimental GM maize MON 88017 field. *IOBC/WPRS Bulletin* 2011, 66:367-370.°**(608 DB) Source: CAB**

Rauschen S: Biosafety research into Diabrotica-resistant Bt-maize : indicator organisms and monitoring methods. *Ph.D.* RWTH Aachen University, Germany; 2008.°**(647 DB) Source: BAS**

Rauschen S, Eckert J, Schaarschmidt F, Schuphan I, Gathmann A: An evaluation of methods for assessing the impacts of Bt-maize MON810 cultivation and pyrethroid insecticide use on Auchenorrhyncha (planthoppers and leafhoppers). *Agricultural and Forest Entomology* 2008, 10(4):331-339.°**(229 DB) Source: WOS ZOO AOA CAB GOO SCO**

Reyes SG: Wet season population abundance of Micraspis discolor (Fabr.) (Coleoptera: Coccinellidae) and Trichomma cnaphalocrosis Uchida (Hymenoptera: Ichnuemonidae) on three transgenic corn hybrids in two sites in the Philippines. *Asia Life Sciences* 2005, 14(2):217-224.°**(627 DB) Source: Reference in review (EFSA–GMO-CZ-2008-54)**

Reyes SG, Jovillano-Mostoles MDA: Diversity, community structure and wet season population abundance of insects in Bt-corn agroecosystem in two sites on Luzon Island, Philippines. *Asia Life Sciences* 2005, 14(1):55-73.°**(917 N4 N5) Source: ZOO BIO**

Rezac M, Pekar S, Kocourek F: Effect of Bt-maize on epigeic spiders (Araneae) and harvestmen (Opiliones). *Plant Protection Science* 2006, 42(1):1-8.°**(519 DB) Source: BIO ZOO CAB AGS**

Rose R, Dively GP: Effects of insecticide-treated and lepidopteran-active Bt Transgenic sweet corn on the abundance and diversity of arthropods. *Environmental Entomology* 2007, 36(5):1254-1268.°**(224 DB N4) Source: WOS BIO ZOO AOA CAB GOO SCO BAS**

Rose RI: An ecological risk assessment of Bt transgenic sweet corn on non-target arthropod communities. *Ph.D.* Ann Arbor: University of Maryland, College Park, USA; 2005.°**(638 DB N4 N5) Source: PRO BAS**

Sanchez ML, Linares JC, Herrera CF, Garcia KDP: Analysis of benefic entomofauna in transgenic and conventional corn crops, Cordoba-Colombia. *Temas Agrarios* 2018, 23(2):121-130.°**(962 N3) Source: WOS**

Schorling M: Ökologische und phytomedizinische Untersuchungen zum Anbau von Bt-Mais im Maiszünsler-Befallsgebiet Oderbruch; Ecological and phytomedical investigations on Bt maize grown in the European corn borer (Ostrinia nubilalis) infested area in the Oderbruch region (Germany). *Ph.D.* Universität Potsdam, Germany; 2005.°**(612 DB N4) Source: BAS**

Schorling M, Freier B: Six-year monitoring of non-target arthropods in Bt maize (Cry 1Ab) in the European corn borer (Ostrinia nubilalis) infestation area Oderbruch (Germany). *Journal fur Verbraucherschutz und Lebensmittelsicherheit* 2006, 1(Suppl. 1):106-108.°**(613 DB) Source: CAB GOO SCO**

Schultheis E: Faunistic approach for an environmental risk assessment of a Bt-maize with multiple resistance genes and evaluation of Trigonotylus caelestialium (KIRKALDY) and Lumbricus terrestris LINNAEUS as model organisms. *Ph.D.* RWTH Aachen University, Germany; 2011.°**(648 DB N5) Source: BAS**

Sedlacek JD, Friley KL, Hillman SL: Ear Pests and Damage to Organic, Conventional and Bt-Protected Sweet Corn Grown in Central Kentucky. *Journal of Entomological Science* 2009, 44(4):383-390.°**(623 DB) Source: WOS BIO ZOO CAB SCO**

Sehnal F, Habustova O, Spitzer L, Hussein HM, Ruzicka V: A biannual study on the environmental impact of Bt maize. *IOBC-WPRS Bulletin* 2004, 27(3):147-160.°**(228 DB) Source: ZOO CAB**

Siegfried BD, Zoerb AC, Spencer T: Development of European corn borer larvae on Event 176 Bt corn: influence on survival and fitness. *Entomologia Experimentalis Et Applicata* 2001, 100:15-20.°**(641 DB) Source: Reference in review (Romeis et al. 2006)**

Sked SL, Calvin DD: Synchronization of Macrocentrus cingulum (Reinhardt) (Hymenoptera: Braconidae) with its host and the effects of transgenic crop systems on this natural enemy. *California Conference on Biological Control III: 15-16 August, 2002; Berkeley, USA*: Edited by Hoddle MS. Center for Biological Control, College of Natural Resources, University of California 2002: 16-22.°**(907 N5) Source: CAB**

Skokova Habustova O, Svobodova Z, Cagan L, Sehnal F: Use of Carabids for the Post-Market Environmental Monitoring of Genetically Modified Crops. *Toxins* 2017, 9(4):18.°**(661 DB) Source: WOS BIO CAB SCO**

Skoková Habuštová O, Svobodová Z, Spitzer L, Doležal P, Hussein HM, Sehnal F: Communities of ground-dwelling arthropods in conventional and transgenic maize: Background data for the post-market environmental monitoring. *Journal of Applied Entomology* 2015, 139(1-2):31-45.°**(605 DB N4) Source: SCO**

Song X, Chang L, Reddy GVP, Zhang L, Fan C, Wang B: Use of Taxonomic and Trait-Based Approaches to Evaluate the Effects of Transgenic Cry1Ac Corn on the Community Characteristics of Soil Collembola. *Environmental Entomology* 2019, 48(1):263-269.°**(664 N4) Source: CAB SCO WOS BIO ZOO**

Stephens EJ: Direct and indirect non-target effects of Cry3Bb Bt corn. *Ph.D.* Ann Arbor: Cornell University, USA; 2006.°**(915 N5) Source: PRO**

Stephens EJ, Losey JE, Allee LL, DiTommaso A, Bodner C, Breyre A: The impact of Cry3Bb Bt-maize on two guilds of beneficial beetles. *Agriculture Ecosystems & Environment* 2012, 156:72-81.°**(634 DB N4) Source: WOS BIO ZOO AOA CAB SCO**

Sushilkumar, Raghuvanshi MS, Anil D, Singh VP: Glyphosate tolerant and insect resistant transgenic Bt maize efficacy against shoot borer, cob borer and non-target insect pests. *Indian Journal of Weed Science* 2017, 49(3):241-247.°**(959 N3) Source: CAB**

Svobodova Z, Habustova O, Sehnal F, Holec M, Hussein HM: Epigeic spiders are not affected by the genetically modified maize MON 88017. *Journal of Applied Entomology* 2013, 137(1-2):56-67.°**(606 DB N4) Source: WOS BIO ZOO CAB SCO**

Svobodova Z, Habustova OS, Bohac J, Sehnal F: Functional diversity of staphylinid beetles (Coleoptera: Staphylinidae) in maize fields: testing the possible effect of genetically modified, insect resistant maize. *Bulletin of Entomological Research* 2016, 106(4):432-445.°**(655 DB N4) Source: WOS BIO ZOO CAB AGR SCO**

Svobodová Z, Skoková Habuštová O, Hutchison WD, Hussein HM, Sehnal F: Risk Assessment of Genetically Engineered Maize Resistant to Diabrotica spp.: Influence on Above-Ground Arthropods in the Czech Republic. *Plos One* 2015, 10(6):e0130656.°**(637 DB N4) Source: Personal contact**

Svobodová Z, Skoková Habuštová O, Spitzer L, Sehnal F: Importance of functional classification in the use of carabids for the environmental risk assessment of the GE crops and other agricultural practices. *Insect Science* 2020, 27(2):375-388.°**(654 DB N4) Source: SCO**

Szekeres D, Kadar F, Kiss J: Activity density, diversity and seasonal dynamics of ground beetles (Coleoptera : Carabidae) in Bt-(MON810) and in isogenic maize stands. *Entomologica Fennica* 2006, 17(3):269-275.°**(219 DB N4) Source: WOS BIO ZOO CAB SCO**

Szenasi A, Marko V: Flea beetles (Coleoptera: Chrysomelidae, Alticinae) in Bt- (MON810) and near isogenic maize stands: Species composition and activity densities in Hungarian fields. *Crop Protection* 2015, 77:38-44.°**(666 DB N4) Source: WOS BIO ZOO CAB AGR SCO**

Szenasi A, Palinkas Z, Zalai M, Schmitz OJ, Balog A: Short-term effects of different genetically modified maize varieties on arthropod food web properties: an experimental field assessment. *Scientific Reports* 2014, 4:5315.°**(629 DB N4) Source: WOS BAS SCO BIO**

Toschki A, Hothorn LA, Ross-Nickoll M: Effects of cultivation of genetically modified Bt maize on epigeic arthropods (Araneae; Carabidae). *Environmental Entomology* 2007, 36(4):967-981.°**(639 DB N4) Source: WOS BIO ZOO AOA CAB GOO SCO**

Toth F, Arpas K, Szekeres D, Kadar F, Szentkiralyi F, Sztnasi A, Kiss J: Spider web survey or whole plant visual sampling? Impact assessment of Bt corn on non-target predatory insects with two concurrent methods. *Environmental Biosafety Research* 2004, 3(4):225-231.°**(211 DB) Source: BIO ZOO CAB GOO SCO**

Truter J, Van Hamburg H, Van Den Berg J: Comparative Diversity of Arthropods on Bt Maize and Non-Bt Maize in two Different Cropping Systems in South Africa. *Environmental Entomology* 2014, 43(1):197-208.°**(631 DB N4) Source: WOS BIO ZOO CAB SCO BAS**

Twardowski J, Beres P, Hurej M, Klukowski Z: A Quantitative Assessment of the Unintended Effects of Bt-Maize (MON 810) on Rove Beetle (Col., Staphylinidae) Assemblages. *Polish Journal of Environmental Studies* 2014, 23(1):215-220.°**(663 DB) Source: WOS BIO ZOO CAB SCO**

Twardowski JP, Beres P, Hurej M, Klukowski Z, Warzecha R: Effects of maize expressing the insecticidal protein Cry1ab on non-target ground beetle assemblages (Coleoptera, Carabidae). *Romanian Agricultural Research* 2017, 34:352-361.°**(920 N4) Source: Reference in Review (Alvarez 2019 Post market monitoring report)**

Twardowski JP, Beres PK, Hurej M, Klukowski Z, Dabrowski ZT, Sowa S, Warzecha R: The quantitative changes of ground beetles (Col., Carabidae) in Bt and conventional maize crop in Southern Poland. *Journal of Plant Protection Research* 2012, 52(4):404-409.°**(640 DB) Source: BIO CAB SCO**

Venditti ME, Steffey KL: Field effects of Bt corn on the impact of parasitoids and pathogens on European corn borer in Illinois. *1st International Symposium on Biological Control of Arthropods: 14-18 January 2002; Honolulu, Hawaii, USA*: Edited by Van Driesche RG. United States Department of Agriculture, Forest Service 2003: 278-283.°**(918 N5) Source: CAB ZOO**

Vidal S: Untersuchungen zu Kaskadeneffekten einer Bt-Toxin-Expression in Maispflanzen auf Pflanze-Herbivor-Parasitoid-Systeme am Beispiel von Blattläusen und ihren Parasitoidkomplexen. *Verbundprojekt: Sicherheitsforschung und Monitoringmethoden zum Anbau von Bt-Mais, Teilprojekt 125, Final Report.* 2004: 32 pages.°**(652 DB N4) Source: Specialist Search (GMO-safety.eu)**

Wang B, Chang L, Wu D, Song X: Effect of transgenic corn cultivation and sampling location on feeding habits of Collembola. *Journal of Agricultural Science and Technology A* 2014, 4(3):235-242.°**(644 DB N4) Source: CAB**

Wang B, Fan C, Yin J, Wu F, Wang D, Song X: Effects of planting transgenic insect-resistant maize with cry1Ac gene Bt-799 on the composition structure of soil fauna. *Journal of Plant Protection* 2019, 46(1):167-174.°**(674 DB N4) Source: CAB**

Wendt C, Freier B, Volkmar C, Schorling M, Wieacker K: Assessment of Bt maize effects on non-target arthropods in field studies using the evaluation approach of "good ecological state". *IOBC/WPRS Bulletin* 2010, 52:103-109.°**(614 DB) Source: CAB**

Wold SJ, Burkness EC, Hutchison WD, Venette RC: In-field monitoring of beneficial insect populations in transgenic corn expressing a Bacillus thuringiensis toxin. *Journal of Entomological Science* 2001, 36(2):177-187.°**(75 DB) Source: WOS BIO ZOO AOA CAB SCO**

Xing Y, Qin Z, Feng M, Li A, Zhang L, Wang Y, Dong X, Zhang Y, Tan S, Shi W: The impact of Bt maize expressing the Cry1Ac protein on non-target arthropods. *Environmental Science and Pollution Research* 2019, 26(6):5814-5819.°**(662 DB N4) Source: SCO AOA SCO WOS BIO**

Zeilinger AR, Andow DA, Zwahlen C, Stotzky G: Earthworm populations in a northern US Cornbelt soil are not affected by long-term cultivation of Bt maize expressing Cry1Ab and Cry3Bb1 proteins. *Soil Biology & Biochemistry* 2010, 42(8):1284-1292.°**(628 DB) Source: WOS BIO ZOO AOA CAB SCO**

Relevant reviews (“**Rev**”) on environmental/ non-target effects of GM crops/ Bt crops/ Bt maize identified in the reference screening. The reference sections of those reviews were screened for additional references.

The sources (bibliographic databases, full text databases, specialist search, personal contacts, reviews) for each review are included. Databases are abbreviated as follows: WOS: Web of Science Core Collection; BIO: BIOSIS; ZOO: Zoological Record; SCI: SciELO; AOA: Agricola; AGS: AGRIS; CAB: CAB Abstracts; BAS: BASE; PRO: ProQuest; GOO: Google Scholar; JST: JSTOR; SCO: Scopus. See Additional file 1, Table S1.1 for details.

The following list of references is sorted by author(s) and year.

Anonymous: Application for authorization of MON 89034 × MON 88017 production and cultivation in the European Union, according to Regulation (EC) No 1829/2003 on genetically modified food and feed - Part I: Technical dossier. EFSA–GMO-BE-2009-71. Monsanto Company; 2009: 425 pages.°**(Rev) Source: Specialist search (EFSA)**

Anonymous: Application for authorization of MON 89034 x NK603 production and cultivation in the European Union under Regulation (EC) No 1829/2003 on genetically modified food and feed - Part I: Technical dossier. EFSA–GMO-NL-2009-72. Monsanto Company; 2009: 383 pages.°**(Rev) Source: Specialist search (EFSA)**

Anonymous: Annual monitoring report on the cultivation of MON 810 in 2010. Brussels, Belgium: Monsanto Europe S.A.; 2011: 30 pages.°**(Rev) Source: Specialist search (EFSA)**

Anonymous: Planes de seguimiento ambiental del cultivo de maíz modificado genéticamente en España. Ministerio de Medio Ambiente y Medio Rural y Marino; 2010.°**(Rev) Source: Specialist search (ISAAA website)**

Anonymous: Application EFSA-GMO-CZ-2008-54 according to Regulation (EC) No 1829/2003 - Response to EFSA questions. Monsanto Company; 2011: 39 pages.°**(Rev) Source: Specialist search (EFSA)**

Anonymous: Application for authorization of stacked Bt11 x MIR604 x GA21 maize cultivation in the European Union under Regulation (EC) No 1829/2003 - Part 1: technical dossier. EFSA–GMO-NL-2010-84.: Syngenta; 2011: 119 pages.°**(Rev) Source: Specialist search (EFSA)**

Anonymous: Scientific opinion on application (EFSA-GMO-CZ-2008-54) for placing on the market of genetically modified insect resistant and herbicide tolerant maize MON 88017 for cultivation under Regulation (EC) No 1829/2003 from Monsanto. *EFSA Journal* 2011, 9(11):2428.°**(Rev) Source: CAB**

Anonymous: Scientific Opinion updating the evaluation of the environmental risk assessment and risk management recommendations on insect resistant genetically modified maize 1507 for cultivation. *EFSA Journal* 2011, 9(11):2429.°**(Rev) Source: CAB**

Anonymous: A review of the environmental safety of Vip3Aa. Center for Environmental Risk Assessment, ILSI Research Foundation; 2012: 19 pages.°**(Rev) Source: Specialist search (CERA webpage)**

Anonymous: Annual monitoring report on the cultivation of MON 810 in 2011. Brussels, Belgium: Monsanto Europe S.A.; 2012: 31 pages.°**(Rev) Source: Specialist search (EFSA)**

Anonymous: Scientific Opinion updating the risk assessment conclusions and risk management recommendations on the genetically modified insect resistant maize MON 810. *EFSA Journal* 2012, 10(12):3017.°**(Rev) Source: CAB BAS**

Anonymous: A review of the environmental safety of the Cry1F protein. Center for Environmental Risk Assessment, ILSI Research Foundation; 2013: 28 pages.°**(Rev) Source: Specialist search (CERA webpage)**

Anonymous: A review of the environmental safety of the Cry2Ab protein. Center for Environmental Risk Assessment, ILSI Research Foundation; 2013: 26 pages.°**(Rev) Source: Specialist search (CERA webpage)**

Anonymous: A review of the environmental safety of the Cry34Ab1 and Cry35Ab1 proteins. Center for Environmental Risk Assessment, ILSI Research Foundation; 2013: 16 pages.°**(Rev) Source: Specialist search (CERA webpage)**

Anonymous: Annual monitoring report on the cultivation of MON 810 in 2012. Brussels, Belgium: Monsanto Europe S.A.; 2013: 60 pages.°**(Rev) Source: Specialist search (EFSA)**

Anonymous: A review of the environmental safety of the Cry3Bb1 protein. Center for Environmental Risk Assessment, ILSI Research Foundation; 2014: 40 pages.°**(Rev) Source: Specialist search (CERA webpage)**

Anonymous: Revised annual post-market environmental monitoring (PMEM) report on the cultivation of genetically modified maize MON 810 in 2013 from Monsanto Europe S.A. *EFSA Journal* 2015, 13(11).°**(Rev) Source: CAB BAS**

Anonymous: Revised annual monitoring report on the cultivation of MON 810 in 2013. Brussels, Belgium: Monsanto Europe S.A.; 2015: 63 pages.°**(Rev) Source: Specialist search (EFSA)**

Abbas MST: Genetically engineered (modified) crops (Bacillus thuringiensis crops) and the world controversy on their safety. *Egyptian Journal of Biological Pest Control* 2018, 28:12.°**(Rev) Source: WOS CAB**

Álvarez F, Camargo AM, Devos Y, European Food Safety A: Assessment of the 2017 post-market environmental monitoring report on the cultivation of genetically modified maize MON 810. *EFSA Journal* 2019, 17(6).°**(Rev) Source: SCO WOS**

Alvarez F, Devos Y, Georgiadis M, Ean AM, Waigmann E, Efsa: Annual post-market environmental monitoring report on the cultivation of genetically modified maize MON 810 in 2016. *EFSA Journal* 2018, 16(5):34.°**(Rev) Source: WOS CAB**

Ammann K: Effects of biotechnology on biodiversity: herbicide-tolerant and insect-resistant GM crops. *Trends in Biotechnology* 2005, 23(8):388-394.°**(Rev) Source: WOS**
Ammann K: Biodiversity and Genetically Modified Crops. In: *Environmental Impact of Genetically Modified Crops.* Edited by Ferry N, Gatehouse AMR. Wallingford: CAB International; 2009: 240-264.°**(Rev) Source: BIO**
Birch AN, Casacuberta J, De Schrijver A, Gathmann A, Gralak M, Guerche P, Jones H, Manachini B, Messean A, Naegeli H *et al*: Revised annual post-market environmental monitoring (PMEM) report on the cultivation of genetically modified maize MON 810 in 2013 from Monsanto Europe S.A. EFSA Panel on Genetically Modified Organisms (GMO). *EFSA Journal* 2015, 13(11):37.°**(Rev) Source: WOS**
Birch AN, Casacuberta J, De Schrijver A, Gathmann A, Gralak M, Guerche P, Jones H, Manachini B, Messean A, Naegeli H *et al*: Annual post-market environmental monitoring (PMEM) report on the cultivation of genetically modified maize MON 810 in 2014 from Monsanto Europe S.A. *EFSA Journal* 2016, 14(4):26.°**(Rev) Source: WOS CAB BAS**
Carpenter JE: Impacts of GM crops on biodiversity. *GM Crops* 2011, 2(1):1-17.°**(Rev) Source: Specialist search (Europabio webpage)**
Center for Environmental Risk Assessment ILSI Research Foundation: A review of the environmental safety of the Cry1Ab protein. *Environmental Biosafety Research* 2011, 10:51-71.°**(Rev) Source: Specialist search (CERA webpage)**
Dabrowski ZT, Gorecka J: Pollen of genetically modified crops and butterflies. *Kosmos (Warsaw)* 2006, 55(2-3):259-265. **(Rev) Source: ZOO**
Dale PJ, Clarke B, Fontes EMG: Potential for the environmental impact of transgenic crops. *Nature Biotechnology* 2002, 20(6):567-574.°**(Rev) Source: GOO**
Devos Y, Alvarez-Alfageme F, Gennaro A, Mestdagh S: Assessment of unanticipated unintended effects of genetically modified plants on non-target organisms: a controversy worthy of pursuit? *Journal of Applied Entomology* 2016, 140(1-2):1-10.°**(Rev) Source: WOS**
Devos Y, De Schrijver A, De Clercq P, Kiss J, Romeis J: Bt-maize event MON 88017 expressing Cry3Bb1 does not cause harm to non-target organisms. *Transgenic Research* 2012, 21(6):1191-1214.°**(Rev) Source: WOS BIO CAB SCO AGS BAS AOA**
Dolezel M, al. e: Ecological effects of genetically modified maize with insect resistance and/or herbicide tolerance - Ökologische Effekte von gentechnisch verändertem Mais mit Insektenresistenz und/oder Herbizidresistenz. In: *Forschungsberichte der Sektion IV.* vol. 6. Wien: Bundesministerium für Gesundheit und Frauen; 2005: 70 pages.°**(Rev) Source: Specialist search (BMG Austria)**
DongYan W, ZhenYing W, KangLai H, Bin C: The function of Bt transgenic corn in IPM system and its ecological risks. *Acta Phytophylacica Sinica* 2003, 30(1):97-106.°**(Rev) Source: Specialist search (Bibliosafety icgeb.org)**
EFSA Panel on Genetically Modified Organisms: Revised annual post-market environmental monitoring (PMEM) report on the cultivation of genetically modified maize MON 810 in 2013 from Monsanto Europe SA. *EFSA Journal* 2015, 13(11):4295.°**(Rev) Source: GOO**
EFSA Panel on Genetically Modified Organisms: Annual post-market environmental monitoring (PMEM) report on the cultivation of genetically modified maize MON 810 in 2015 from Monsanto Europe SA. *EFSA Journal* 2017, 15(5):e04805.°**(Rev) Source: GOO**
Eizaguirre M, Albajes R, Lopez C, Eras J, Baraibar B, Lumbierres B, Pons X: Transgenic Bt maize: main results of a six-year study on nontarget effects. *Bulletin OILB/SROP* 2006, 29(5):49-55.°**(Rev) Source: CAB**
Eizaguirre M, Albajes R, Lopez C, Eras J, Lumbierres B, Pons X: Six years after the commercial introduction of Bt maize in Spain: field evaluation, impact and future prospects. *Transgenic Research* 2006, 15(1):1-12.°**(Rev) Source: WOS BIO CAB GOO SCO AOA**
Emani C: The effects of transgenic crops on non-target organisms. In: *Biotechnology and Biodiversity.* Edited by Ahuja MR, Ramawat KG. Springer; 2014: 59-66.°**(Rev) Source: GOO**
Federici BA: Effects of Bt on non-target organisms. *Journal of New Seeds* 2003, 5(1):11-30.°**(Rev) Source: Specialist search (Bibliosafety icgeb.org)**
Frisvold GB, Reeves JM: Integrated pest management, Bt crops, and insecticide use: The U.S. experience. In: *Integrated Pest Management.* Edited by Peshin R, Pimentel D. vol. 4: Springer Netherlands; 2014: 15-36.°**(Rev) Source: SCO**
Gatehouse AMR, Ferry N, Edwards MG, Bell HA: Insect-resistant biotech crops and their impacts on beneficial arthropods. *Philosophical Transactions: Biological Sciences* 2011, 366(1569):1438-1452.°**(Rev) Source: JST**
Gould F, Amasino RM, Brossard D, Buell CR, Dixon RA, Falck-Zepeda JB, Gallo MA, Giller K, Glenna L, Griffin TS *et al*: Agronomic and Environmental Effects of Genetically Engineered Crops. Washington: Natl Academies Press; 2016.°**(Rev) Source: WOS**
Hagvar EB, Aasen S: Possible Effects of Genetically Modified Plants on Insects in the Plant Food Web. *Latvijas Entomologs* 2004, 41:111-117.°**(Rev) Source: Specialist search (Third World Network; Biosafety-info.net)**
Head G: Compatibility of biological control with lepidopteran-protected Bt corn. *3rd International Symposium on Biological Control of Arthropods: 8-13 February, 2009; Christchurch, New Zealand.* USDA, Forest Health Technology Enterprise Team 2009: 186-190.°**(Rev) Source: CAB**
Hilbeck A, Schmidt JEU: Another View on Bt Proteins – How Specific are They and What Else Might They Do? *Biopesticides International* 2006, 2(1).°**(Rev) Source: Reference in review (several)**
Icoz I, Stotzky G: Fate and effects of insect-resistant Bt crops in soil ecosystems. *Soil Biology & Biochemistry* 2008, 40(3):559-

586.°**(Rev) Source: WOS BIO ZOO AOA CAB SCO**
Knox O, Hall C, McVittie A, Walker R, Knight B: A systematic review of the environmental impacts of GM crop cultivation as reported from 2006 to 2011. (Special issue on advanced research in genetically modified foods (GMF).). *Food and Nutrition Sciences* 2013, 4(6A):28-44.°**(Rev) Source: CAB BAS**
Kolseth AK, D'Hertefeldt T, Emmerich M, Forabosco F, Marklund S, Cheeke TE, Hallin S, Weih M: Influence of genetically modified organisms on agro-ecosystem processes. *Agriculture Ecosystems & Environment* 2015, 214:96-106.°**(Rev) Source: WOS BIO**
Lang A, Otto M: A synthesis of laboratory and field studies on the effects of transgenic Bacillus thuringiensis (Bt) maize on non-target Lepidoptera. *Entomologia Experimentalis Et Applicata* 2010, 135(2):121-134.°**(Rev) Source: WOS BIO ZOO AOA CAB GOO SCO BAS**
Li B, Meng L, Wan F: The impact of insect resistant transgenic crops on natural enemies. *Chinese Journal of Biological Control* 2002, 18(3):97-105.°**(Rev) Source: ZOO CAB**
Lundgren JG, Gassmann AJ, Bernal J, Duan JJ, Ruberson J: Ecological compatibility of GM crops and biological control. *Crop Protection* 2009, 28(12):1017-1030.°**(Rev) Source: WOS**
Malone LA, Burgess EPJ: Impact of Genetically Modified Crops on Pollinators. In: *Environmental Impact of Genetically Modified Crops.* Edited by Ferry N, Gatehouse AMR. Wallingford: CAB International; 2009: 199-224.°**(Rev) Source: WOS**
Marvier M, McCreedy C, Regetz J, Kareiva P: A meta-analysis of effects of Bt cotton and maize on nontarget invertebrates. *Science* 2007, 316(5830):1475-1477.°**(Rev) Source: WOS BIO ZOO CAB GOO JST SCO AOA**
McKee MJ, Fernandez S, Nickson TE, Head GP: An assessment of the environmental impact of genetically modified crops in the US. *The BCPC International Congress: Crop Science and Technology: 10-12 November 2003; SECC, Glasgow, Scotland, UK.*: British Crop Protection Council 2003: 1075-1084.°**(Rev) Source: CAB WOS**
Mertens M: Assessment of environmental impacts of genetically modified plants - Implementation of the Biosafety Protocol Development of Assessment Bases FKZ 201 67 430/07. *BfN - Skripten.* vol. 217. Bonn: Federal Agency for Nature Conservation; 2008: 240.°**(Rev) Source: Specialist search (BfN Germany)**
Michaud D: Environmental impact of transgenic crops. II. Impact of recombinant traits. *Phytoprotection* 2005, 86(2):107-124.°**(Rev) Source: WOS BIO CAB SCO**
Naegeli H, Birch AN, Casacuberta J, De Schrijver A, Gralak MA, Guerche P, Jones H, Manachini B, Messean A, Nielsen EE *et al*: Annual post-market environmental monitoring (PMEM) report on the cultivation of genetically modified maize MON 810 in 2015 from Monsanto Europe S.A. *EFSA Journal* 2017, 15(5):27.°**(Rev) Source: WOS CAB**
Naranjo SE: Impacts of Bt crops on non-target invertebrates and insecticide use patterns. *CAB reviews: Perspectives in agriculture, veterinary science, nutrition and natural resources* 2009, 4(011):1-11.°**(Rev) Source: CAB GOO SCO AOA BAS**
Naranjo SE, Head G, Dively GP: Field Studies Assessing Arthropod Nontarget Effects in Bt Transgenic Crops: Introduction. *Environmental Entomology* 2005, 34(5):1178-1180.°**(Rev) Source: BIO AOA CAB GOO BAS**
Naranjo SE: Effects of GM crops on non-target organisms. In: *Plant Biotechnology: Experience and Future Prospects.* Editey by Ricroch A et al.; Springer; 2014: 129-142.°**(Rev) Source: GOO**
Obrycki JJ, Ruberson JR, Losey JE: Interactions between natural enemies and transgenic insecticidal crops. In: *Genetics, Evolution and Biological Control.* Edited by Ehler LE, Sforza R, Mateille T. Cambridge: CAB International; 2004: 183-206.°**(Rev) Source: BIO**
O'Callaghan M, Glare TR, Burgess EPJ, Malone LA: Effects of plants genetically modified for insect resistance on nontarget organisms. *Annual Review of Entomology* 2005, 50:271-292.°**(Rev) Source: WOS GOO**
Ortego F, Pons X, Albajes R, Castanera P: European Commercial Genetically Modified Plantings and Field Trials. In: *Environmental Impact of Genetically Modified Crops,* Edited by Ferry N, Gatehouse A. CAB International, 2009:327-343.°**(Rev) Source: WOS BIO SCO CAB**
Pellegrino E, Bedini S, Nuti M, Ercoli L: Impact of genetically engineered maize on agronomic, environmental and toxicological traits: a meta-analysis of 21 years of field data. *Scientific Reports* 2018, 8:12.°**(Rev) Source: WOS BIO SCO**
Peterson JA, Lundgren JG, Harwood JD: Interactions of transgenic Bacillus thuringiensis insecticidal crops with spiders (Araneae). *Journal of Arachnology* 2011, 39(1):1-21.°**(Rev) Source: WOS BIO ZOO CAB JST SCO**
Pilson D, Prendeville HR: Ecological effects of transgenic crops and the escape of transgenes into wild populations. *Annual Review of Ecology Evolution and Systematics* 2004, 35:149-174.°**(Rev) Source: WOS JST**
Ricroch A, Akkoyunlu S, Martin-Laffon J, Kuntz M: Assessing the Environmental Safety of Transgenic Plants: Honey Bees as a Case Study. In: *Transgenic Plants and Beyond.* Edited by Kuntz M, vol. 86. London: Academic Press Ltd-Elsevier Science Ltd; 2018: 111-167.°**(Rev) Source: WOS BIO SCO**
Romeis J, Meissle M, Bigler F: Transgenic crops expressing Bacillus thuringiensis toxins and biological control. *Nature Biotechnology* 2006, 24(1):63-71.°**(Rev) Source: WOS BIO CAB GOO**
Romeis J, Meissle M, Naranjo SE, Li Y, Bigler F: The end of a myth—Bt (Cry1Ab) maize does not harm green lacewings. *Frontiers in Plant Science* 2014, 5:391.°**(Rev) Source: GOO**
Romeis J, Meissle M, Raybould A, Hellmich RL: Impact of Insect-resistant Transgenic Crops on Above-ground Non-target Arthropods. In: *Environmental Impact of Genetically Modified Crops.* Edited by Ferry N, Gatehouse AMR. Wallingford: CAB International; 2009: 165-198.°**(Rev) Source: BIO**
Romeis J, Naranjo SE, Meissle M, Shelton AM: Genetically engineered crops help support conservation biological control.

Biological control 2019, 130(130):136-154.°**(Rev) Source: AOA CAB SCO WOS BIO ZOO**
Sanvido O, Romeis J, Bigler F: Ecological impacts of genetically modified crops: Ten years of field research and commercial cultivation. *Advances in Biochemical Engineering-Biotechnology* 2007, 107:235-278.°**(Rev) Source: WOS SCO**
Sanvido O, Stark M, Romeis J, Bigler F: Ecological impacts of genetically modified crops. Experiences from ten years of experimental field research and commercial cultivation, vol. 1. Zürich: Agroscope Reckenholz-Tänikon Research Station ART; 2006.°**(Rev) Source: AGS CAB**
Schuler TH, Poppy GM, Kerry BR, Denholm I: Potential side effects of insect-resistant transgenic plants on arthropod natural enemies. *Trends in Biotechnology* 1999, 17(5):210-216.°**(Rev) Source: GOO**
Shirai Y: Effects of transgenic insecticidal crops on environments and ecosystems: literature until 2010. *Bulletin of the National Institute of Agro-Environmental Sciences* 2012, 30:1-38.°**(Rev) Source: BIO CAB**
Shirai Y: Nontarget effect of transgenic insecticidal crops: Overview to date and future challenges. *Japanese Journal of Applied Entomology and Zoology* 2007, 51(3):165-186.°**(Rev) Source: WOS BIO ZOO CAB SCO**
Steffey KL, Venditti M, Barrido BR, Felsot AS: Effect of Bacillus thuringiensis corn on natural enemies of the European corn borer. In: *Agricultural Biotechnology: Challenges and Prospects.* Edited by Bhalgat MK, Ridley WP, Felsot AS, Seiber JN, vol. 866. Washington: Amer Chemical Soc.; 2004: 139-150.°**(Rev) Source: WOS**
Theissen B, Russell DJ: The relevance of Collembola in monitoring soil-ecological effects of GMOs. *Gefahrstoffe Reinhaltung Der Luft* 2009, 69(10):391-394.°**(Rev) Source: WOS**
Thies JE, Devare MH: An ecological assessment of transgenic crops. *Journal of Development Studies* 2007, 43(1):97-129.°**(Rev) Source: WOS**
Velkov VV, Medvinsky AB, Sokolov MS, Marchenko AI: Will transgenic plants adversely affect the environment? *Journal of Biosciences* 2005, 30(4):515-548.°**(Rev) Source: GOO**
Venter HJ, Bohn T: Interactions between Bt crops and aquatic ecosystems: a review. *Environmental Toxicology and Chemistry* 2016, 35(12):2891-2902.°**(Rev) Source: WOS**
Wolfenbarger LL: Annotated Bibliography on Environmental and Ecological Impacts from Transgenic Plants II: Unintended Effects. Information Systems for Biotechnology, Virginia Tech; 2003: 37 pages.°**(Rev) Source: Specialist search (ISAAA website)**
Wolfenbarger LL, Naranjo SE, Lundgren JG, Bitzer RJ, Watrud LS: Bt Crop Effects on Functional Guilds of Non-Target Arthropods: A Meta-Analysis. *Plos One* 2008, 3(5).°**(Rev) Source: WOS AOA CAB BAS GOO SCO BIO**
Yang H, Peng Y, Tian J, Wang J, Hu J, Song Q, Wang Z: biosafety assessment of Bt rice and other Bt crops using spiders as example for non-target arthropods in China. *Plant Cell Reports* 2017, 36(4):505-517.°**(Rev) Source: GOO**
Yaqoob A, Shahid AA, Samiullah TR, Rao AQ, Khan MAU, Tahir S, Mirza SA, Husnain T: Risk assessment of Bt crops on the non-target plant-associated insects and soil organisms. *Journal of the Science of Food and Agriculture* 2016, 96(8):2613-2619.°**(Rev) Source: GOO**
Yu HL, Li YH, Wu KM: Risk Assessment and Ecological Effects of Transgenic Bacillus thuringiensis Crops on Non-Target Organisms. *Journal of Integrative Plant Biology* 2011, 53(7):520-538.°**(Rev) Source: WOS BIO SCO CAB AOA GOO AGS**

References excluded at the full text stage are labelled with **EX** together with the reason for exclusion:

- Ex01: No original data (off-topic reviews, summaries, abstracts, opinion papers, etc.)
- Ex02: Same data published in other article with higher quality (e.g., peer-reviewed paper), more detail, or more suitable format
- Ex03: No animal populations naturally inhabiting fields (or their margins) were sampled (e.g., laboratory studies)
- Ex04: Study on target pests (Lepidoptera-targeted maize: *Busseola fusca*, *Chilo partellus, Diatraea grandiosella*, *Diatraea saccharalis*, *Helicoverpa zea*, *Helicoverpa armigera*, *Heliothis* spp., *Ostrinia nubilalis*, *Ostrinia furnacalis, Striacosta albicosta, Sesamia nonagrioides*, *Spodoptera frugiperda*, and *Spodoptera exigua*; Coleoptera-targeted maize: *Diabrotica* spp.)
- Ex05: No Bt maize grown in the field
- Ex06: No appropriate comparison to corresponding non-Bt maize
- Ex07: No data on abundance or activity density, predation or parasitization rate, species richness, biodiversity, or community structure, or other measure for animals in the Bt / non-Bt comparison.
- Ex08: unreplicated experiment (only one Bt and non-Bt field)
- ExDUP: duplicate reference not detected automatically
- ExPERM: Regulatory documents where permission was not requested or not obtained from the study owners

Not obtained full texts are labelled with "**NO**".

The following list of references is sorted by author(s) and year.

Anonymous: Ökologische Risikoabschätzung von Freisetzungen gentechnisch veränderter Organismen für die spezielle Situation in Österreich. In: *Forschungsbericht.* vol. 4; 1997.°**(Ex01)**

Anonymous: Sicherheitsforschung bei transgenen Pflanzen-Endbericht. In: *Forschungsbericht.* vol. 6; 1998.°**(Ex01)**

Anonymous: Report on the potential for exposure of British Lepidoptera to toxic GM maize pollen. Greenpeace International; 1999: 9 pages. **(Ex03)**

Anonymous: Application for authorisation of genetically modified 1507xNK603 maize submitted under Regulation (EC) No 1829/2003 (EFSA-GMO-UK-2005-17) - Response to questions from the Spanish Competent Authority received through the EFSA GMO Unit on 20 July 2009. Pioneer Hi-Bred International / Mycogen Seeds; 2005: 28 pages. **(Ex01)**

Anonymous: Application for the authorisation of genetically modified 1507xNK603 maize and derived food and feed in accordance with Regulation (EC) 1829/2003 including authorisation for cultivation in accordance with Directive 2001/18/EC. EFSA-GMO-UK-2005-17.Pioneer Hi-Bred International / Mycogen Seeds; 2005: 103 pages.°**(Ex01)**

Anonymous: Application for the authorisation of genetically modified 59122 maize and derived food and feed in accordance with regulation (ec) 1829/2003 including authorisation for cultivation in accordance with directive 2001/18/EC - DAS-59122-7 MAIZE. EFSA-GMO-NL-2005-23. Pioneer Hi-Bred International / Mycogen Seeds; 2005: 306 pages.°**(Ex01)**

Anonymous: Response to questions from the Spanish Competent Authority and request for additional information (Application EFSA-GMO-UK-2005-17). Pioneer Hi-Bred International / Mycogen Seeds; 2005: 40 pages.°**(Ex01)**

Anonymous: Scientific review by EFSA of application EFSA-GMO-NL-2005-23 - 59122 maize; Questions to Pioneer Hi-Bred and Mycogen Seeds regarding maize 59122. Pioneer Hi-Bred International / Mycogen Seeds; 2005: 26 pages.°**(Ex01)**

Anonymous: Application EFSA–GMO-NL-2005-26 according to Regulation (EC) No 1829/2003- Responses to Spanish CNB questions. Monsanto Company; 2007: 17 pages.°**(Ex01)**

Anonymous: Application for authorization of NK603 x MON 810 maize for cultivation in the European Union under Regulation (EC) No 1829/2003 on genetically modified food and feed - Part I: Technical dossier. EFSA–GMO-NL-2005-26. Monsanto Company; 2007: 273 pages.°**(Ex01)**

Anonymous: Application EFSA–GMO-NL-2005-26 according to Regulation (EC) No 1829/2003- Responses to French CA questions. Monsanto Company; 2008: 15 pages.°**(Ex01)**

Anonymous: Genepeace Briefing - Blurring the lines between fact and fiction. Europabio. Brussels; 2008: 6 pages.°**(Ex01)**

Anonymous: Application for authorization of Event MIR604 maize cultivation in the European Union under Regulation (EC) No 1829/2003 - Part 1: Technical dossier. EFSA–GMO-UK-2010-83. Syngenta; 2009: 79 pages.°**(Ex01)**
Anonymous: Application for authorization of genetically modified maize 59122 submitted under Regulation (EC) No 1829/2003 – Stop-the-clock (6) - EFSA-GMO-NL-2005-23 Response to questions by the Environment Working Group of the EFSA GMO Panel dated 13 February 2009. Pioneer Hi-Bred International / Mycogen Seeds; 2009: 18 pages.°**(Ex01)**
Anonymous: Biosafety in non-human gene technology. Results of the research programme 2004–2008. Bern: Federal Office for the Environment FOEN; 2009.°**(Ex01)**
Anonymous: EuropaBio Workshop - Environmental risk assessment for the cultivation of genetically modified crops. 15-16 October 2009; Hotel Silken Berlaymont, Brussels, Belgium. EuropaBio: 3 pages.°**(Ex01)**
Anonymous: Annual monitoring report on the cultivation of MON 810 in 2009. Brussels, Belgium: Monsanto Europe S.A.; 2010: 27 pages.°**(Ex01)**
Anonymous: Application for authorization of MON 88017 maize for cultivation in the European Union, according to Regulation (EC) No 1829/2003 on genetically modified food and feed - Part I: Technical dossier. EFSA–GMO-CZ-2008-54. Monsanto Company; 2010: 305 pages.°**(Ex01)**
Anonymous: Application for authorization of MON 89034 maize for cultivation in the European Union, according to Regulation (EC) No 1829/2003 on genetically modified food and feed - Part I: Technical dossier. EFSA–GMO-BE-2011-90. Monsanto Company; 2010: 368 pages.°**(Ex01)**
Anonymous: EuropaBio Workshop - Monitoring cultivation of genetically modified crops. 27 & 28 October 2010; Stanhope Hotel, Brussels, Belgium. EuropaBio: 27 pages.°**(Ex01)**
Anonymous: Submission from Norway on the Risks of GMOs to Biodiversity and Human Health. Third World Network Biosafety Information Centre; 2010: 11 pages.°**(Ex01)**
Anonymous: Scientific Opinion on the annual Post-Market Environmental Monitoring (PMEM) report from Monsanto Europe S.A. on the cultivation of genetically modified maize MON810 in 2009. *EFSA Journal* 2011, 9(10):2376.°**(Ex01)**
Anonymous: Application for authorization of genetically modified maize 59122 submitted under Regulation (EC) No 1829/2003 – Clock remains stopped EFSA (7); Questions to Pioneer on Maize 59122 dated 30 April 2010 (EFSA-GMO-NL-2005-23); Pioneer Response Submitted 13 January 2012. Pioneer Hi-Bred International / Mycogen Seeds; 2012: 58 pages.°**(Ex01)**
Anonymous: Scientific opinion on the annual post-market environmental monitoring (PMEM) report from Monsanto Europe S.A. on the cultivation of genetically modified maize MON 810 in 2010. *EFSA Journal* 2012, 10(4):2610.°**(Ex01)**
Anonymous: Scientific Opinion updating the risk assessment conclusions and risk management recommendations on the genetically modified insect resistant maize 1507. *EFSA Journal* 2012:2933.°**(Ex01)**
Anonymous: Scientific Opinion updating the risk assessment conclusions and risk management recommendations on the genetically modified insect resistant maize Bt11. *EFSA Journal* 2012, 10(12):3018.°**(Ex01)**
Anonymous: Additional information - Application EFSA-GMO-UK-2010-83 for authorization under Regulation (EC) No 1829/2003 of genetically modified MIR604 maize - stop-the-clock (1), January 2013. Syngenta; 2013: 38 pages.°**(Ex01)**
Anonymous: Africa bullied to grow defective Bt Maize: the failure of Monsanto's MON810 maize in South Africa. African Centre for Biosafety; 2013: 38 pages.°**(Ex01)**
Anonymous: Science not fiction: Time to think again about GM. Europabio; 2013: 28 pages.°**(Ex01)**
Anonymous: Scientific Opinion on an application from Pioneer Hi-Bred International and Dow AgroSciences LLC (EFSA-GMO-NL-2005-23) for placing on the market of genetically modified maize 59122 for food and feed uses, import, processing and cultivation under Regulation (EC) No 1829/2003. *EFSA Journal* 2013, 11(3):3135. **(Ex01)**
Anonymous: Scientific Opinion on the annual Post-Market Environmental Monitoring (PMEM) report from Monsanto Europe S.A. on the cultivation of genetically modified maize MON 810 in 2011. *EFSA Journal* 2013, 11(12):3500.°**(Ex01)**
Anonymous: Testbiotech comment on EFSA Scientific Opinion on an application from Pioneer Hi-Bred International and Dow AgroSciences LLC (EFSA-GMO-NL-2005-23) for placing on the market of genetically modified maize 59122 for food and feed uses, import, processing and cultivation under Regulation (EC) No 1829/2003. TestBiotech; 2013: 8 pages.°**(Ex01)**
Anonymous: Annual monitoring report on the cultivation of MON 810 in 2013. Brussels, Belgium: Monsanto Europe S.A.; 2014: 26 pages.°**(Ex01)**
Anonymous: Scientific opinion on the annual post-market environmental monitoring (PMEM) report from Monsanto Europe S.A. on the cultivation of genetically modified maize MON 810 in 2012. *EFSA Journal* 2014, 12(6):3704.°**(Ex01)**
Anonymous: Ecological Biochemistry: Environmental and Interspecies Interactions: Edited by Krauss GI, Nies DH, Wiley-V C H Verlag Gmbh, Weinheim, Germany; 2015. **(Ex01)**
Anonymous: Final Report Summary - GRACE (GMO Risk Assessment and Communication of Evidence). Julius Kühn Institut; 2015: 17 pages.°**(Ex01)**
Anonymous: Periodic Report Summary 3 - AMIGA (Assessing and Monitoring the Impacts of Genetically modified plants on Agro-ecosystems). Agenzia Nazionale Per Le Nuove Tecnologie,L'Energia E Lo Sviluppo Economico Sostenibile; 2016.°**(Ex01)**
Anonymous: XXIX International Horticultural Congress on Horticulture: Sustaining Lives, Livelihoods and Landscapes (IHC2014): III International Genetically Modified Organisms in Horticulture Symposium - Past, Present and Future. In: *XXIX International Horticultural Congress on Horticulture: Sustaining Lives, Livelihoods and Landscapes.* Edited by Panis B, HvoslefEide TAK, Drew R, Lane V, vol. 1124: Int Soc Horticultural Science, Leuven, Belgium; 2016.°**(Ex01)**
Adebayo Oluwakemi O, Omodele I: The current status of cereal (maize, rice and sorghum) crops cultivation in Africa: need for

integration of advances in transgenic for sustainable crop production. *International Journal of Agricultural Policy and Research* 2015, 3(3):133-145.°**(Ex01)**
Afidchao MM, Musters CJM, de Snoo GR: Asian corn borer (ACB) and non-ACB pests in GM corn (Zea mays L.) in the Philippines. *Pest Management Science* 2013, 69(7):792-801.°**(Ex04)**
Agarwal S, Grover A, Paul Khurana SM: Plant molecular biology tools to develop transgenics. In: *Applied Molecular Biotechnology: The Next Generation of Genetic Engineering.* CRC Press; 2016: 33-60.°**(Ex01)**
Ahmad A: Effect of transgenic Bacillus thuringiensis corn expressing the Cry3Bb1 toxin on above and below ground nontarget organisms. *Ph.D.* Ann Arbor: Kansas State University, USA; 2004.°**(Ex02)**
Ahmad A, Wilde GE, Zhu KY: Detectability of coleopteran-specific Cry3Bb1 protein in soil and its effect on nontarget surface and below-ground arthropods. *Environmental Entomology* 2005, 34(2):385-394.°**(ExDUP)**
Ahsan R, Campbell C, Romano MC, Whiles MR, Middleton B: Bt-corn detritus impacts on larval Caddisflies (Lepidostoma liba Ross). *Transactions of the Illinois State Academy of Science* 2002, 95(Supplement):88.°**(Ex01)**
Albajes R, Lumbierres B, Pons X, Comas J: Changes in arthropod fauna from weed management practices in genetically modified herbicide-tolerant maize. *Journal of Agricultural Science* 2016, 6(10) :67-78 **(Ex05)**
Albajes R, Lumbierres B, Madeira F, Comas C, Ardanuy A, Lee MS, Iglesias S, Comas J, Perez-Hedo M, Lopez C *et al*: Field trials for assessing risks of GM maize on non-target arthropods in Europe: the Spanish experience. *IOBC/WPRS Bulletin* 2013, 97:1-8.°**(Ex02)**
Albajes R, Lumbierres B, Madeira F, Pons X: Field trials to assess risks of transgenic crops for non-target arthropods: power analysis and surrogate arthropods in Spain. *IOBC/WPRS Bulletin* 2012, 73:1-7.°**(Ex02)**
Albajes R, Lumbierres B, Pons X, Comas J: Representative taxa in field trials for environmental risk assessment of genetically modified maize. *Bulletin of Entomological Research* 2013, 103(6):724-733.°**(Ex06)**
Alcalde E: Post-market monitoring plans of Bt-176 in Spain: 1998-2005. *Journal fur Verbraucherschutz und Lebensmittelsicherheit* 2006, 1(Suppl. 1):102-105.°**(Ex01)**
Alcantara EP, Caoili BL, Javier PJ, Mostales MDJ: Multi-year, large-scale comparison of arthropod communities in commercially managed Bt and non-Bt corn fields. In: *40th Annual Convention of the Pest Management Council of the Philippines: 5-8 May 2009; Baguio City, Philippines*. Philippine Entomologist 2009: 182-183.°**(Ex01)**
Al-Deeb MA: Transgenic corn for corn rootworm control: effect on corn rootworm biology and above and below ground nontarget organisms. *Ph.D.* Ann Arbor: Kansas State University, USA; 2002.°**(NO)**
Alford AM, Krupke CH: A Meta-analysis and Economic Evaluation of Neonicotinoid Seed Treatments and Other Prophylactic Insecticides in Indiana Maize From 2000-2015 With IPM Recommendations. *Journal of Economic Entomology* 2018, 111(2):689-699.°**(Ex01)**
Allison A. Snow B: Environmental Impact of Genetically Modified Crops. *The Quarterly Review of Biology* 2010, 85(1):97.°**(Ex01)**
Altieri MA, others: The ecological impacts of transgenic crops on agroecosystem health. *Ecosystem Health* 2000, 6(1):13-23.°**(Ex01)**
Alvarez-Alfageme F, Ortego F, Castanera P: Bt maize fed-prey mediated effect on fitness and digestive physiology of the ground predator Poecilus cupreus L. (Coleoptera: Carabidae). *Journal of Insect Physiology* 2009, 55(2):143-149.°**(Ex03)**
Alves VM, Hernandez MIM: Morphometric Modifications in Canthon quinquemaculatus Castelnau 1840 (Coleoptera: Scarabaeinae): Sublethal Effects of Transgenic Maize? *Insects* 2017, 8(4):10.°**(Ex06)**
Andersen MN, Sausse C, Lacroix B, Caul S, Messean A: Agricultural studies of GM maize and the field experimental infrastructure of ECOGEN. *Pedobiologia* 2007, 51(3):175-184.°**(Ex01)**
Anderson PL: Field distribution and effects of Bt-expressing corn anthers on survival, development, and behavior of the monarch butterfly, Danaus plexippus (L.). *Ph.D.* Ann Arbor: Iowa State University, USA; 2004.°**(Ex03)**
Anderson PL, Hellmich RL, Sears MK, Sumerford DV, Lewis LC: Effects of Cry1Ab-expressing corn anthers on monarch butterfly larvae. *Environmental Entomology* 2004, 33(4):1109-1115.°**(Ex03)**
Andow DA, Hilbeck A: Science-based risk assessment for nontarget effects of transgenic crops. *Bioscience* 2004, 54(7):637-649.°**(Ex01)**
Andow DA, Zwahlen C: Ground beetle acquisition of Cry1Ab from plant- and residue-based food webs. *Biological Control* 2016, 103:204-209.°**(Ex07)**
Arndt M: Monitoring the environmental effects of Bacillus thuringiensis maize: possible effects on nematodes. *Gesunde Pflanzen* 2006, 58(1):67-74.°**(Ex02)**
Arpaia S, Birch N, Chesson A, du Jardin P, Gathmann A, Gropp J, Herman L, Hoen-Sorteberg HG, Jones H, Kiss J *et al*: Scientific Opinion on the annual post-market environmental monitoring (PMEM) report from Monsanto Europe S.A. on the cultivation of genetically modified maize MON 810 in 2012 EFSA Panel on Genetically Modified Organisms (GMO). *EFSA Journal* 2014, 12(6):29.°**(Ex01)**
Arpaia S, Birch N, Chesson A, du Jardin P, Gathmann A, Gropp J, Herman L, Hoen-Sorteberg HG, Jones H, Kiss J *et al*: Scientific Opinion on the annual post-market environmental monitoring (PMEM) report from Monsanto Europe S.A. on the cultivation of genetically modified maize MON 810 in 2013. *EFSA Journal* 2015, 13(3):11.°**(Ex01)**
Arpaia S, Birch A, Chesson A, du Jardin P, Gathmann A, Gropp J, Herman L, Hoen-Sorteberg H-G, Jones H, Kiss J *et al*: Scientific Opinion on the annual post-market environmental monitoring (PMEM) report from Monsanto Europe S.A. on the cultivation of genetically modified maize MON 810 in 2012. 2015.°**(ExDUP)**

Arpaia S, Birch ANE, Chesson A, du Jardin P, Gathmann A, Gropp J, Herman L, Hoen-Sorteberg HG, Jones H, Kiss J *et al*: Scientific Opinion on application (EFSA-GMO-DE-2011-95) for the placing on the market of genetically modified maize 5307 for food and feed uses, import and processing under Regulation (EC) No 1829/2003 from Syngenta Crop Protection AG. *EFSA Journal* 2015, 13(5):29.°**(Ex01)**

Arpaia S, Birch ANE, Chesson A, du Jardin P, Gathmann A, Gropp J, Herman L, Hoen-Sorteberg HG, Jones H, Kiss J *et al*: Scientific Opinion on an application (EFSA-GMO-NL-2010-80) for the placing on the market of herbicide-tolerant genetically modified maize NK603 x T25 for food and feed uses, import and processing under Regulation (EC) No 1829/2003 from Monsanto. *EFSA Journal* 2015, 13(7):23.°**(Ex01)**

Arpaia S, Birch ANE, Kiss J, Van Loon JJ, Messéan A, Nuti M, Perry JN, Sweet JB, Tebbe CC: Assessing environmental impacts of genetically modified plants on non-target organisms: the relevance of in planta studies. *Science of the Total Environment* 2017, 583:123-132.°**(Ex01)**

Arpaia S, Messean A, Birch NA, Hokannen H, Haretel S, van Loon J, Lovei G, Park J, Spreafico H, Lovei G *et al*: Assessing and monitoring impacts of genetically modified plants on agro-ecosystems: the approach of AMIGA project. *Entomologia* 2014, 2(1):79-86.°**(Ex01)**

Arpas K, Toth F, Kiss J: Analysis of web content of Theridion impressum L. Koch (Araneae: Theridiidae) in BT (DK 440 BTY, MON 810, Cry1Ab) and isogenic (DK 440) maize. *IOBC-WPRS Bulletin* 2004, 27(3):23-29.°**(Ex02)**

Arpas K, Toth F, Kiss J: Analysis of web content of Theridion impressum L. Koch (Araneae: Theridiidae) in BT and isogenic corn. *Novenyvedelem* 2004, 40(2):61-65.°**(Ex02)**

Balazs E, Dudits D, Sagi L: Plain facts about GMOs - Hungary white paper. Szeged: Barabás Zoltán Federation of Biotechnology / Dénes Dudits; 2011: 136 pages.°**(Ex01)**

Balog A, Szenasi A, Szekeres D, Kiss J: Staphylinids (Coleoptera: Staphylinidae) in genetically modified maize ecosystems: species densities and trophic interactions. *IOBC/WPRS Bulletin* 2010, 52:9-15.°**(Ex02)**

Barry BD, Darrah LL, Huckla DL, Antonio AQ, Smith GS, O'Day MH: Performance of transgenic corn hybrids in Missouri for insect control and yield. *Journal of Economic Entomology* 2000, 93(3):993-999.°**(Ex04)**

Batie SS: The Environmental Impacts of Genetically Modified Plants: Challenges to Decision Making. *American Journal of Agricultural Economics* 2003, 85(5):1107-1111. **(Ex01)**

Bauer-Panskus A, Then C: Testbiotech opinion concerning the application for market approval of genetically modified maize 1507 (DAS-Ø15Ø7-1). TestBiotech; 2010: 25 pages.°**(Ex01)**

Bauer-Panskus A, Then C: Case study: Industry influence in the risk assessment of genetically engineered maize 1507. TestBiotech; 2014: 32 pages.°**(Ex01)**

Bawa AS, Anilakumar KR: Genetically modified foods: safety, risks and public concerns-a review. *Journal of Food Science and Technology* 2013, 50(6):1035-1046. **(Ex01)**

Birch AN, Casacuberta J, De Schrijver A, Gathmann A, Gralak MA, Guerche P, Jones H, Manachini B, Messean A, Naegeli H *et al*: Scientific Opinion on an application by Dow Agrosciences LLC (EFSA-GMO-NL-2009-68) for placing on the market of cotton 281-24-236 x 3006-210-23 x MON 88913 for food and feed uses, import and processing under Regulation (EC) No 1829/2003. *EFSA Journal* 2016, 14(4):21.°**(Ex01)**

Birch AN, Casacuberta J, De Schrijver A, Gralak MA, Guerche P, Jones H, Manachini B, Messean A, Naegeli H, Nielsen EE *et al*: Scientific opinion on application (EFSA-GMO-NL-2011-96) for the placing on the market of genetically modified insect-resistant and herbicide-tolerant cotton GHB119, for food and feed uses, import and processing under Regulation (EC) No 1829/2003 from Bayer CropScience AG. *EFSA Journal* 2016, 14(10):27.°**(Ex01)**

Birch ANE *et al*: Biodiversity and Non-target Impacts: Maize in Kenya. In: *Environmental Risk Assessment of Genetically Modified Organisms.* Edited by Hilbeck A, Andow DA, vol. 1. Wallingford: CAB Interantional; 2004: 117-186.°**(Ex01)**

Birch ANE, Griffiths BS, Caul S, Thompson J, Heckmann LH, Krogh PH, Cortet J: The role of laboratory, glasshouse and field scale experiments in understanding the interactions between genetically modified crops and soil ecosystems: A review of the ECOGEN project. *Pedobiologia* 2007, 51(3):251-260.°**(Ex01)**

Bitzer RJ, Rice ME, Pilcher CD, Pilcher CL, Lam WKF: Bioversity and community structure of epedaphic and euedaphic springtails (Collembola) in transgenic rootworm Bt corn. *Environmental Entomology* 2005, 34(5):1346-1376.°**(ExDUP)**

Blum M, Stolz U, Bagley MJ: Assessing possible ecological risks of genetically modified crops: gene expression assays and genetic monitoring of non-target organisms. *Science Forum 2003: May 5-7, 2003; Washington, D. C., USA*. U. S. Environmental Protection Agency 2003. **(Ex01)**

Botha M, Siebert S, Van den Berg J, Maliba B, Ellis S: Plant and arthropod diversity patterns of maize agro-ecosystems in two grassy biomes of South Africa. *Biodiversity and Conservation* 2015, 24(7):1797-1824.°**(Ex05)**

Brown CR: Natural Enemy Abundance and Biological Control in Bt Maize Using Simulations of Predator-Prey Interactions. *Ph.D.* Ann Arbor: Montana State University; 2018.°**(Ex01)**

Brownbridge M: Preliminary observations on effects of Bt-corn on non-target soil Collembola (abstract). In: *36th Annu Meet Soc Invertebr Pathol, : 2003; Vermont*. 40.°**(Ex01)**

Buechs W, Raubuch M, Prescher S, Behr K, Mueller A, Roose K: Impact of Ostrinia-resistant Bt-maize on microbial and invertebrate decomposer communities in field soils. *Mitteilungen aus der Biologischen Bundesanstalt fuer Land- und Forstwirtschaft Berlin-Dahlem* 2007, 410:26-32.°**(Ex03)**

Buntin GD, Flanders KL, Lynch RE: Assessment of experimental Bt events against fall armyworm and corn earworm in field corn. *Journal of Economic Entomology* 2004, 97(2):259-264.°**(Ex04)**

Carter D: Environmental impacts of GMO's. *Vecteur Environnement* 2004, 37(6):48-59.°**(Ex01)**
Castro BA, Leonard BR, Riley TJ: Efficacy of selected Bt corn hybrids against corn earworm, sugarcane borer and southwestern corn borer, 1998. In: *Arthropod Management Tests.* Edited by Saxena KN, vol. 24. Lanham: Entomological Society of America; 1999: 415 pages.°**(Ex04)**
Catangui MA, Berg RK: Western bean cutworm, Striacosta albicosta (Smith) (Lepidoptera : Noctuidae), as a potential pest of transgenic Cry1Ab Bacillus thuringiensis corn hybrids in South Dakota. *Environmental Entomology* 2006, 35(5):1439-1452.°**(Ex04)**
Cayabyab B, Alcantara E, Cuaterno W, Seigfreid B, Belen J, Malenab C, Dangan J: A field and laboratory study on post commercialization monitoring of Asian corn borer, Ostrinia furnacalis (Guenee), resistance to Bt corn in the Philippines and the impact of pollen dispersal on non-target lepidoptera. *10th International Symposium on the Biosafety of Genetically Modified Organisms Biosafety research of GMOS: past achievements and future challenges: 16-21 November 2008; Wellington, New Zealand*. International Society for Biosafety Research (ISBR) 2008.°**(Ex01)**
Cerevkova A, Cagan L: Effect of transgenic insect-resistant maize to the community structure of soil nematodes in two field trials. *Helminthologia* 2015, 52(1):41-49.°**(ExDUP)**
Chambers CP: Potential impacts of transgenic crop residues on macroinvertebrate communities in agricultural streams. *M.S.* Ann Arbor: Southern Illinois University at Carbondale, USA; 2008.°**(Ex02)**
Chaufaux J, Micoud A, Delos M, Naibo B, Bombarde F, Eychennes N, Pagliari C, Marque G, Bourguet D: Transgenic maize and non target insects: what effects? [French]. *Phytoma* 2002, 555:13-16.°**(Ex02)**
Clark PL, Brown CR, Horak MJ, Zampierin SM, Scarabello MC, Stork LG: Evaluation of phenotypic characteristics and ecological interactions in fields planted with lepidopteran-protected maize MON 89034 and MON 89034 × NK603 in Argentina during 2005/06. Monsanto Company; 2007: 189 pages.°**(ExPERM)**
Cocco J, Dolibaina DR, Casagrande MM, Specht A, Foerster LA: First records of Leucania rawlinsi Adams and L. senescens Moschler (Lepidoptera: Noctuidae) in Brazil: redescription, potential association with Bt maize, larval parasitoids, and spatial and temporal distribution. *Zootaxa* 2019, 4604(3):441-460.°**(Ex06)**
Comas C, Lumbierres B, Pons X, Albajes R: No effects of Bacillus thuringiensis maize on nontarget organisms in the field in southern Europe: a meta-analysis of 26 arthropod taxa. *Transgenic Research* 2014, 23(1):135-143.°**(ExDUP)**
Comas J, Lumbierres B, Comas C, Pons X, Albajes R: Optimising the capacity of field trials to detect the effect of genetically modified maize on non-target organisms through longitudinal sampling. *Annals of Applied Biology* 2015, 166(2):183-195.°**(Ex01)**
Comas J, Lumbierres B, Pons X, Albajes R: Ex-Ante Determination of the Capacity of Field Tests to Detect Effects of Genetically Modified Corn on Nontarget Arthropods. *Journal of Economic Entomology* 2013, 106(4):1659-1668.°**(Ex02)**
Corrales Madrid JL, Martínez Carrillo JL, Osuna Martínez MB, Durán Pompa HA, Alonso Escobedo J, Javier Quinones F, Garzón Tiznado J, Castro Espinoza L, Zavala Garcia F, Espinoza Banda A *et al*: Transportability of non-target arthropod field data for the use in environmental risk assessment of genetically modified maize in Northern Mexico. *Journal of Applied Entomology* 2018, 142(5):525-538.°**(ExDUP)**
Cotter J: GE insect resistant (Bt) maize in Europe: an unnecessary threat to wildlife and GE-free choice. *Technical Note.* vol. 3. Exeter, UK: Greenpeace Research Laboratories 2006: 7 pages.°**(Ex01)**
Cotter J: GM insect-resistant (Bt) maize in Europe: a growing threat to wildlife and agriculture. *Technical Note.* vol. 2. Exeter, UK: Greenpeace Research Laboratories 2009: 10 pages.°**(Ex01)**
Cotter J, Mueller W: A critique of the European Food Safety Authority’s opinion on genetically modified maize MON810. Greenpeace & Friends of the Earth Europe; 2009: 27 pages.°**(Ex01)**
Dai PL, Zhou W, Zhang J, Cui HJ, Wang Q, Jiang WY, Sun JH, Wu YY, Zhou T: Field assessment of Bt cry1Ah corn pollen on the survival, development and behavior of Apis mellifera ligustica. *Ecotoxicology and Environmental Safety* 2012, 79:232-237.°**(Ex03)**
Daly TG: Impact of Bacillus thuringiensis transgenic corn on non-target and phytophagous arthropod populations. *M.Sc.*,The University of Georgia, USA; 2003.°**(Ex02)**
Dang C, Lu ZB, Wang L, Chang XF, Wang F, Yao HW, Peng YF, Stanley D, Ye GY: Does Bt rice pose risks to non-target arthropods? Results of a meta-analysis in China. *Plant Biotechnology Journal* 2017, 15(8):1047-1053. **(Ex01)**
Darvas B, Csoti A, Adel G, Peregovits L, Ronkay L, Lauber E, Polgar AL: Some data to the risk analysis of Bt-corn pollen and protected Lepidoptera species in Hungary. *Novenyvedelem* 2004, 40(9):441-449.°**(Ex01)**
Darvas B, Deli S, Nemeth G, Banati H, Fuleki L, Szekacs A: Field trials with genetically modified plants in Europe and Hungary (1999-2012). *Novenyvedelem* 2013, 49(11):491-500.°**(Ex01)**
Daudu CK, Muchaonyerwa P, Mnkeni PNS: Litterbag decomposition of genetically modified maize residues and their constituent Bacillus thuringiensis protein (Cry1Ab) under field conditions in the central region of the Eastern Cape, South Africa. *Agriculture Ecosystems & Environment* 2009, 134(3-4):153-158.°**(Ex03)**
da Silva LV, de Paula Ribeiro AL, Lucio AD: Aracnidae diversity in soil cultivated with corn (Zea mays). *Semina: Ciencias Agrarias* 2014, 35(4 Suppl):2395-2404.°**(Ex08)**
de Assis VCB, Chagas PG, Marinho CGS, Fadini MAM, Delabie JHC, Mendes SM: Transgenic Bt maize does not affect the soil ant community. *Pesquisa Agropecuaria Brasileira* 2018, 53(2):152-162.°**(Ex08)**
de Billot M: Phenotypic and ecological interactions of Lepidopteran protected and glyphosate-tolerant maize NK603 x MON 810 in Germany and Spain field trials during 2007. Monsanto Company; 2009: 80 pages.°**(ExPERM)**

de Billot M: Phenotypic and ecological interactions of Lepidopteran protected maize MON 89034, Lepidopteran-protected and glyphosate tolerant maize MON 89034 x NK 603 and Coleopteran and Lepidopteran protected and glyphosate-tolerant maize MON 89034 x MON 88017 in Germany and Spain field trials during 2007. Monsanto Company; 2009: 151 pages.°**(ExPERM)**

de Billot M: Amended Report for MSL0022350: Amended Report for MSL0021758: Phenotypic and Ecological Interactions of Lepidopteran-protected maize MON 89034, Lepidopteran- Protected and Glyphosate-Tolerant Maize MON 89034 x NK 603 and Coleopteran and Lepidopteran-Protected and Glyphosate-Tolerant Maize MON 89034 x MON 88017 in Germany and Spain Field Trials During 2007. Monsanto Company; 2010: 149 pages.°**(ExPERM)**

de Sousa MF, Fernandes MG, da Silva Guimaraes AJ: Influence of Bt maize on diversity and composition of non-target arthropod species. *Journal of Agricultural Science* 2019, 11(2):201-209.°**(Ex08)**

Degenhardt J: Verbundprojekt: Sicherheitsforschung und Monitoringmethoden zum Anbau von Bt-Mais, Teilprojekt Auswirkungen von Bt-Endotoxin auf die tritrophische Interaktion zwischen Mais, Nichtziel-Lepidopteren und deren Parasitoiden. München: Max Planck Gesellschaft zur Förderung der Wissenschaften e.V. 2005: 41 pages.°**(Ex03)**

Devos Y, Alan R, Karen EH, Sol O-G: Teosinte and maize-teosinte hybrid plants in Europe-Environmental risk assessment and management implications for genetically modified maize. *Agriculture, ecosystems & environment* 2018, 259(259):19-27.°**(Ex01)**

DiTommaso A, Ryan MR, Mohler CL, Brainard DC, Shuler RE, Allee LL, Losey JE: Effect of Cry3Bb Bt Corn and Tefluthrin on Postdispersal Weed Seed Predation. *Weed Science* 2014, 62(4):619-624.°**(ExDUP)**

DiTommaso A, Ryan MR, Mohler CL, Brainard DC, Shuler RE, Allee LL, Losey JE: Effect of Cry3Bb Bt Corn and Tefluthrin on Postdispersal Weed Seed Predation. *Weed Science* 2014, 62(4):619-624.°**(ExDUP)**

Dively GP, Rose R: Effects of Bt transgenic and conventional insecticides on the natural enemy community in sweet corn. *California Conference on Biological Control III: 15-16 August, 2002; Berkeley, USA*: Edited by Hoddle MS. Center for Biological Control, College of Natural Resources, University of California 2002: 9-15.°**(Ex02)**

Dively GP, Rose R: Effects of Bt transgenic and conventional insecticide control on the non-target natural enemy community in sweet corn. *1st International Symposium on Biological Control of Arthropods: January 14-18, 2002; Honolulu, Hawaii.* USDA Forest Service FHTET-03-05 2003.°**(Ex02)**

Dolezel M, al. e: Review of scientific evidence including latest finding concerning Austrian safeguard measures for GM-Maize lines MON810 and T25. In: *Forschungsberichte der Sektion IV.* vol. 1. Wien: Bundesministerium für Gesundheit, Familie und Jugend; 2007: 69 pages.°**(Ex01)**

Dolezel M, Miklau M, Heissenberger A, Reichenbecher W: Are Limits of Concern a useful concept to improve the environmental risk assessment of GM plants? *Environmental Sciences Europe* 2017, 29:15.°**(Ex01)**

Duan JJ, Jiang C, Head GP, Bhatti MA, Ward DP, Levine SL, Nickson TE, Nemeth MA: Statistical power analysis of a 2-year field study and design of experiments to evaluate non-target effects of genetically modified Bacillius thuringiensis corn. *Ecological Entomology* 2006, 31(5):521-531.°**(Ex02)**

Duan JJ, Marvier M, Huesing J, Dively G, Huang ZY: A Meta-Analysis of Effects of Bt Crops on Honey Bees (Hymenoptera: Apidae). *Plos One* 2008, 3(1).°**(Ex01)**

Eckerstorfer M, al. e: Supplementary Risk Assessment for GM Maize Mon 810 with regard to the conclusions of the WTO-Panel in the case 'Ec Biotech' on Austria safeguard for GM maize. In: *Forschungsberichte der Sektion IV.* vol. 4. Wien: Bundesministerium für Gesundheit, Familie und Jugend; 2007: 35 pages.°**(Ex01)**

Eckert J, Gathmann A, Schuphan I: Impact of growing Bt-maize on non target organisms: thrips and their antagonists. *Mitteilungen der Deutschen Gesellschaft fuer Allgemeine und Angewandte Entomologie* 2004, 14(1-6):439-442.°**(Ex02)**

Ecuru J: A Pathway for Biosafety Regulation of GMOs in Sub-Saharan Africa: Cambridge Univ Press, Cambridge, Uk; 2017.°**(Ex01)**

Eichenseer H, Strohbehn R, Burks J: Frequency and severity of western bean cutworm (Lepidoptera : Noctuidae) ear damage in transgenic corn hybrids expressing different Bacillus thuringiensis cry toxins. *Journal of Economic Entomology* 2008, 101(2):555-563.°**(Ex04)**

Eizaguirre M, Madeira F, Lopez C: Effects of Bt maize on non-target lepidopteran pests. *IOBC/WPRS Bulletin* 2010, 52:49-55.°**(Ex04)**

Eizaguirre M, Pons X, Lumbierres B, Albajes R: Evaluation of Potential Side Effects of Glyphosate-tolerant Maize NK603 × MON 810 to Non Target Organisms under Field Conditions. Monsanto Company; 2009: 72 pages.°**(Ex06)**

El-Wakeil N, Wittmann C, Volkmar C: Evaluation of the abundance of key arthropods in transgenic maize lines in Central Germany. *Julius Kuhn Archiv* 2012, 438:129.°**(Ex01)**

Erasmus A: Response of selected non-target Lepidoptera, Coleoptera and Diptera species to Cry1Ab protein expressed by genetically modified maize. *PhD*, North-West University, South Africa; 2010.°**(Ex03)**

Erasmus A, Van den Berg J: Effect of Bt-maize expressing Cry1Ab toxin on non-target Coleoptera and Lepidoptera pests of maize in South Africa. *African Entomology* 2014, 22(1):167-179.°**(Ex03)**

Estes RE, Heeren JR, Tinsley NA, Steffey KL, Gray ME: Evaluation of transgenic hybrids and insecticidal seed treatments for control of black cutworm larvae (Agrotis ipsilon) in Illinois, 2008. University of Illinois Extension and Department of Crop Sciences; 2008.°**(Ex03)**

Fadini MAM, Araujo OG, Mendes SM, Marinho CGS: Ocorrência do ácaro fitófago Catarhinus tricholaenae Keifer (Acari: Diptilomiopidae) em cultivares de milho Bt Occurrence of the phytophagous mite Catarhinus tricholaenae Keifer (Acari:

Diptilomiopidae) on Bt corn cultivars. *Ciencia Rural, Santa Maria* 2012, 42(9):1524-1527.°**(Ex08)**
Fagan J, Antoniou M, Robinson C: GMO Myths and Truths. 2 edn: Earthopensource; 2014: 331 pages.°**(Ex01)**
Farinos GP, Poza Mdl, Hernandez-Crespo P, Ortego F, Castanera P: Diversity and seasonal phenology of spiders, ground beetles and rove beetles in conventional and transgenic maize in Central Spain. *IOBC/WPRS Bulletin* 2008, 33:75-78.°**(Ex02)**
Federici BA: Benefits of Bt-crops for biological control. *California Conference on Biological Control II: 11-12 July, 2000; The Historic Mission Inn Riverside, California, USA*: Edited by Hoddle MS. Center for Biological Control, College of Natural Resources, University of California 2000: 130-134.°**(Ex01)**
Federici BA: Scientific basis for assessing the impact of Bt crops on biological control agents. *California Conference on Biological Control III: 15-16 August, 2002; Berkeley, USA*: Edited by Hoddle MS. Center for Biological Control, College of Natural Resources, University of California 2002.°**(Ex01)**
Felke M, Langenbruch GA: Auswirkungen des Pollens von transgenem Bt-Mais auf ausgewählte Schmetterlingslarven. *BfN - Skripten.* vol. 157. Bonn: Federal Agency for Nature Conservation; 2005: 143.°**(Ex03)**
Fernandes OD: Effect of genetically modified corn (Mon810) on Spodoptera frugiperda (J. E. Smith, 1797) and on egg parasitoid Trichogramma spp.: *Phd*, Escola Superior de Agricultura Luiz de Queiroz, Estado de São Paulo – Brasil; 2003.°**(Ex08)**
Fernandes OD: Effect of genetically modified corn (mon810) on spodoptera frugiperda (j. e. smith, 1797) and on egg parasitoid trichogramma spp. ; Efeito do milho geneticamente modificado (Mon810) em Spodoptera frugiperda (J.E.Smith, 1797) e no parasitóide de ovos Trichogramma spp [http://www.teses.usp.br/teses/disponiveis/11/11146/tde-12052003-141132/]°**(ExDUP)**
Ferrante M, Elena Larisa T, Gabor LL, Giovanni B, Lenka M, Ludovit C, Mihael Cristin I, Serena M: Predation pressure in maize across Europe and in Argentina: an intercontinental comparison. *Insect science* 2019, 26(3):545-554.°**(ExDUP)**
Ferreira TE, Matiello Fadini MA, Martins Mendes S, Santos Marinho CG, VCruz I: Phytophagous mites on genetically modified maize with Bacillus thuringiensis genes. *Ciencia Rural* 2017, 47:1-7.°**(Ex08)**
Fontes EMG, Carvalho VF: Biosafety on genetically modified plants: an overview in Brazil and in the world. *1st International conference on the processing tomato and 1 International symposium on tropical tomato diseases: 18-22 November 1996; Recife, Pernambuco, Brazil.* American Society for Horticultural Science 1997: 100-103.°**(Ex01)**
Freier B, Richter C, Beuthner V, Schmidt G, Volkmar C: Structure of arthropod communities in Bt maize and conventional maize - results of redundancy analyses of long-term field data from the Oderbruch region in Germany. *Journal fur Kulturpflanzen* 2011, 63(12):401-410.°**(Ex02)**
Freier B, Schorling M, Traugott M, Juen A, Volkmar C: Results of a 4-year plant survey and pitfall trapping in Bt maize and conventional maize fields regarding the occurrence of selected arthropod taxa. *IOBC-WPRS Bulletin* 2004, 27(3):79-84.°**(Ex02)**
French BW, Chandler LD, Ellsbury MM, Fuller BW, West M: Ground beetle (Coleoptera : Carabidae) assemblages in a transgenic corn-soybean cropping system. *Environmental Entomology* 2004, 33(3):554-563.°**(Ex06)**
Frizzas MR: Effect of genetically modified corn MON810 on insect community. *PhD*, Escola Superior de Agricultura Luiz de Queiroz, Estado de São Paulo – Brasil; 2003.°**(Ex08)**
Frizzas MR: Effect of genetically modified corn MON810 on insect community: Piracicaba, SP (Brazil) 2003 192 p; 2003.°**(ExDUP)**
Frizzas MR, Neto SS, de Oliveira CM, Omoto C: Genetically modified corn on fall armyworm and earwig populations under field conditions. *Ciencia Rural* 2014, 44(2):203-209.°**(Ex02)**
Frizzas MR, de Oliveira CM, Omoto C: Diversity of insects under the effect of Bt maize and insecticides. *Arquivos do Instituto Biologico Sao Paulo* 2017, 84:e0062015.°**(Ex08)**
Gatehouse AMR, Ferry N, Raemaekers RJM: The case of the monarch butterfly: a verdict is returned. *Trends in Genetics* 2002, 18(5):249-251.°**(Ex01)**
Gathmann A, Wirooks L, Bartsch D, Schuphan I: Effects of growing Bt-maize on non target organisms: Lepidopteran larvae in weed strips. *Mitteilungen der Deutschen Gesellschaft fuer Allgemeine und Angewandte Entomologie* 2004, 14(1-6):427-430.°**(Ex02)**
Gould F, Amasino RM, Brossard D, Buell CR, Dixon RA, Falck-Zepeda JB, Gallo MA, Giller K, Glenna L, Griffin TS *et al*: Genetically Engineered Crops Through 2015: Natl Academies Press, Washington, Dc, USA; 2016.°**(Ex01)**
Grabowski M, Beres PK, Dabrowski ZT: Characteristic of selected carabid species (Coleoptera: Carabidae) and their suitability for ERA and monitoring of GMO release to the environment. *Progress in Plant Protection* 2010, 50(4):1602-1606.°**(Ex05)**
Griffiths BS, Caul S, Thompson J, Birch ANE, Scrimgeour C, Cortet J, Foggo A, Hackett CA, Krogh PH: Soil microbial and faunal community responses to Bt maize and insecticide in two soils. *Journal of Environmental Quality* 2006, 35(3):734-741.°**(Ex03)**
Griffiths BS, Caul S, Thompson J, Hackett CA, Cortet J, Pernin C, Krogh PH: Soil microbial and faunal responses to herbicide tolerant maize and herbicide in two soils. *Plant and Soil* 2008, 308(1-2):93-103.°**(Ex03)**
Griffiths NA, Tank JL, Royer TV, Rosi-Marshall EJ, Whiles MR, Chambers CP, Frauendorf TC, Evans-White MA: Rapid decomposition of maize detritus in agricultural headwater streams. *Ecological Applications* 2009, 19(1):133-142.°**(Ex03)**
Guo J, He K, Hellmich RL, Bai S, Zhang T, Liu Y, Ahmed T, Wang Z: Field trials to evaluate the effects of transgenic cry1Ie maize on the community characteristics of arthropod natural enemies. *Scientific Reports* 2016, 6(22102).°**(ExDUP)**
Habustova O, Dolezal P, Hussein HM, Spitzer L, Turanli F, Ruzicka V, Sehnal F: Assessment of the impact of Cry1Ab expression on insects dwelling on the maize plants. *Entomological Research* 2007, 37(Suppl. 1):A50-A51.°**(Ex01)**

Habustova O, Dolezal P, Spitzer L, Svobodova Z, Hussein H, Sehnal F: Impact of Cry1Ab toxin expression on the non- target insects dwelling on maize plants. *Journal of Applied Entomology* 2014, 138(3):164-172.°**(ExDUP)**
Habustova O, Sehnal F: Results of a four-year study of the Bt maize impact on arthropod communities. *Kosmos (Warsaw)* 2007, 56(3-4):275-284.°**(Ex02)**
Habustova O, Sehnal F, Hussein H: Insect communities on maize expressing a Bt-toxin. *Acta Fytotechnica et Zootechnica* 2005, 8(1):9-11.°**(Ex02)**
Habustova O, Turanli F, Spitzer L, Ruzicka V, Dolezal P, Sehnal F: Communities of beetles and spiders in the stands of normal and genetically modified maize. *Pestycydy* 2005, 3:125-131.°**(Ex02)**
Habustova OS, Svobodova Z, Spitzer L, Dolezal P, Hussein HM, Sehnal F: Communities of ground-dwelling arthropods in conventional and transgenic maize: background data for the post-market environmental monitoring. *Journal of Applied Entomology* 2015, 139(1-2):31-45.°**(ExDUP)**
Hallam T, Westbrook J, Kennard K, Purucker S, McCracken G: Impact of Bt (Bacillus thuringiensis) crops on bat activity in South Texas agroecosystems. EPA Science Inventory 2007.°**(Ex01)**
Hanley AV: Effect of transgenic Bt corn pollen and glyphosate-resistant canola pollen on the survival of the honey bee (Apis mellifera L.) worker larvae and the use of Bt corn pollen as a controlling method for the greater wax moth (Galleria mellonella L.). *M.S.* Ann Arbor: Michigan State University, USA; 2002.°**(Ex03)**
Harland MLH: Influence of Diabrotica-resistant Cry3Bb1 maize on Chrysoperla spp. (Neuroptera: Chrysopidae) and Orius tristicolor (Heteroptera: Anthocoridae) in South Dakota. *M.S.* Ann Arbor: South Dakota State University, USA; 2003.°**(NO)**
Harwood JD, Samson RA, Obrycki JJ: Temporal detection of Cry1Ab-endotoxins in coccinellid predators from fields of Bacillus thuringiensis corn. *Bulletin of Entomological Research* 2007, 97(6):643-648.°**(Ex07)**
Harwood JD, Wallin WG, Obrycki JJ: Uptake of Bt endotoxins by nontarget herbivores and higher order arthropod predators: molecular evidence from a transgenic corn agroecosystem. *Molecular Ecology* 2005, 14(9):2815-2823.°**(Ex07)**
Heissenberger A, al. e: Monitoring von mit gentechnisch verändertem Mais kontaminierten Maisfeldern. In: *Forschungsberichte der Sektion IV.* vol. 9. Wien: Bundesministerium für Gesundheit und Frauen; 2004: 143 pages.°**(Ex05)**
Hellmich RL, Prasifka JR, Anderson PL: Effects of Bt plants on non-target herbivores. *Aspects of Applied Biology* 2005, 74:75-80.°**(Ex01)**
Herman RA, Zhuang M, Storer NP, Cnudde F, Delaney B: Risk-Only Assessment of Genetically Engineered Crops Is Risky. *Trends in Plant Science* 2019, 24(1):58-68.°**(Ex01)**
Hernández-Juárez A, Aguirre LA, Cerna E, Flores M, Frías GA, Landeros J, Ochoa YM: Abundance of Non-Target Predators in Genetically Modified Corn. *Florida Entomologist* 2019, 102(1):96-100.°**(ExDUP)**
Higgins LS, Dively G: Field-scale assessment of the effects of Cry34Ab1/Cry35AB1 Bt rootworm corn on non-target predaceous coleopterans and their prey. Pioneer Hi-Bred International; 2006: 21 pages.°**(ExPERM)**
Higgins LS, Hong B: Monitoring of Key Non-Target Arthropods in Transgenic Maize Lines Containing Events 59122, 1507x59122, 1507xNK603, 59122xNK603, and 59122x1507xNK603 – 2005 Spain Field Study, Supplemental Analysis Report. Pioneer Hi-Bred International; 2007: 56 pages.°**(ExPERM)**
Higgins LS, Hong B: Monitoring of Key Non-Target Arthropods in Transgenic Maize Lines Containing Events 59122, 1507x59122, 1507xNK603, 59122xNK603, and 59122x1507xNK603 – 2006 European Field Studies (Hungary location). Pioneer Hi-Bred International; 2007: 80 pages.°**(Ex02)**
Higgins LS, Hong B: Monitoring of Key Non-Target Arthropods in Transgenic Maize Lines Containing Events 59122, 1507x59122, 1507xNK603, 59122xNK603, and 59122x1507xNK603 – 2006 European Field Studies (Spain location). Pioneer Hi-Bred International; 2007: 97 pages.°**(ExPERM)**
Higgins LS, Storer N, Pascual A, Layton RJ, Moellenbeck DJ: Three year field monitoring of Cry34/Cry35Ab1 and Cry1FxCry34/Cry35Ab1 maize hybrids for nontarget arthropod effects. Pioneer Hi-Bred International; 2009: 48 pages.°**(Ex02)**
Hilbeck A, McMillan JM, Meier M, Humbel A, Schläpfer-Miller J, Trtikova M: A controversy re-visited: Is the coccinellid Adalia bipunctata adversely affected by Bt toxins? *Environmental Sciences Europe* 2012, 24(10).°**(Ex03)**
Hilbeck A, Andow DA: Environmental risk assessment of genetically modified organisms. Volume 1: A case study of Bt maize in Kenya. Wallingford: CABI Publishing; 2004.°**(Ex01)**
Holst N, Lang A, Lovei G, Otto M: Increased mortality is predicted of Inachis io larvae caused by Bt-maize pollen in European farmland. *Ecological Modelling* 2013, 250:126-133.°**(Ex03)**
Honemann L, Zurbrugg C, Nentwig W: Effects of Bt-corn decomposition on the composition of the soil meso- and macrofauna. *Applied Soil Ecology* 2008, 40(2):203-209.°**(Ex03)**
Horner TA, Dively GP, Herbert DA: Development, survival and fitness performance of Helicoverpa zea (Lepidoptera : Noctuidae) in MON810 Bt field corn. *Journal of Economic Entomology* 2003, 96(3):914-924.°**(Ex04)**
Hurej M, Mietkiewski R, Twardowski JP: The effect of Cry1AB insecticidal protein on the incidence of entomopathogenic fungi infecting aphids on Bt maize. *Zemdirbyste-Agriculture* 2014, 101(3):279-284.°**(Ex07)**
Hussein MH, Svobodova Z, Habustova O, Puza V, Sehnal F: Impact of genetically modified maize expressing Cry 3Bb1 on some non-target arthropods. *Journal of Applied Sciences Research* 2012, 12(October):5124-5131.°**(Ex02)**
Hutchison WD: Insect Resistance Management and Integrated Pest Management for Bt Crops: Prospects for an Area-wide View, vol. 4. Wallingford: Cabi Publishing-CAB Int; 2015.°**(Ex01)**
Jasinski J, Eisley B, Young C, Willson H, Kovach J: Beneficial arthropod survey in transgenic and non-transgenic field crops in

Ohio. *Special Circular Ohio Agricultural Research and Development Center* 2001, 179:99-102.°**(Ex02)**
Jensen PD, Dively GP, Swan CM, Lamp WO: Exposure and nontarget effects of transgenic Bt corn debris in streams. *Environmental Entomology* 2010, 39(2):707-714.°**(Ex03)**
Jesse LCH, Obrycki JJ: Field deposition of Bt transgenic corn pollen: lethal effects on the monarch butterfly. *Oecologia* 2000, 125(2):241-248.°**(Ex03)**
Jesse LCH, Obrycki JJ: Assessment of the non-target effects of transgenic Bt corn pollen and anthers on the milkweed tiger moth, Euchatias egle Drury (Lepidoptera : Arctiidae). *Journal of the Kansas Entomological Society* 2002, 75(1):55-58.°**(Ex03)**
Kalthoff N, Kreuter T, Freier B, Volkmar C, Stark A, Hommel B, Moll E: Field studies on quality and quantity of arthropod communities in Bt-maize and not genetic modified maize. *Mitteilungen der Deutschen Gesellschaft fuer Allgemeine und Angewandte Entomologie* 2001, 13(1-6):269-272.°**(Ex02)**
Kamota A: Effects of bt maize on earthworm activity silage quality and residue decomposition in the central Eastern Cape. *Ph.D* University of Fort Hare, South Africa; 2011.°**(Ex02)**
Kendrick DL, Clark PL: Phenotypic evaluation and ecological observations of lepidopteran-protected corn MON 89034 in Midwestern U.S. field trials during 2005. Monsanto Company; 2006: 96 pages.°**(ExPERM)**
Kendrick DL, Clark PL: Phenotypic evaluation and ecological observations of lepidopteran-protected corn MON 89034 in Northern U.S. field trials during 2005. Monsanto Company; 2006: 44 pages.°**(ExPERM)**
Kendrick DL, Fernandez S, Leafgren RL, Horak MJ: Phenotypic evaluations and ecological observations of lepidopteran protected corn MON 89034 and MON 89597 in U.S. field trials during 2004 for an assessment of equivalence and weed potential. Monsanto Company; 2005: 98 pages.°**(ExPERM)**
Keulder R: Oviposition site preference of lacewings in maize ecosystems and the effect of Bt maize on Chrysoperla pudica (Neuroptera: Chrysopidae). *Ph.D.* North-West University, South Africa; 2010.°**(Ex05)**
Koch MS, Ward JM, Levine SL, Baum JA, Vicini JL, Hammond BG: The food and environmental safety of Bt crops. *Frontiers in Plant Science* 2015, 6:22.°**(Ex01)**
Koch RL, Hutchison WD, Venette RC: Survival of monarch butterfly, Danaus plexippus (Nymphalidae), larvae on milkweed near Bt cornfields. *Journal of the Lepidopterists' Society* 2003, 57(2):92-99.°**(Ex03)**
Krogh PH, Griffiths B: ECOGEN - Soil ecological and economic evaluation of genetically modified crops. *Pedobiologia* 2007, 51(3):171-269.°**(Ex01)**
Kručkienė K: Genetiškai modifikuotų augalų poveikio aplinkai vertinimas ; Genetically modified plant environmental inpact assessment. *M.Sc.* Vytautas Magnus University, Lithuania; 2011.°**(Ex01)**
Kruse-Plass M, Hofmann F, Kuhn U, Otto M, Schlechtriemen U, Schroeder B, Voegel R, Wosniok W: Reply to the EFSA (2016) on the relevance of recent publications (Hofmann et al. 2014, 2016) on environmental risk assessment and management of Bt-maize events (MON810, Bt11 and 1507) (vol 29, pg 12, 2017). *Environmental Sciences Europe* 2017, 29:20.°**(Ex01)**
Lami F, Masetti A, Staiano G, Lener M, Rastelli V, Neri U, Arpaia S, Burgio G: Lady Beetles (Coleoptera: Coccinellidae) in Ecological Compensation Areas surrounding maize fields: chance of exposure to Bt toxins through pollen feeding. *24th Congresso della Società Italiana di Ecologia: 15-17 September 2014; Ferrara, Italy*. Poster available from Research Gate 2014.°**(Ex05)**
Lang A, Abdel-Kader K, Arndt M, Bauchhenss J, Beck R, Benker U, Hermann A, Mautz D, Zellner M, Pommer G: Monitoring the environmental impact of Bt maize: a research project of the Bavarian State Ministry for Health, Environment and Consumer Protection, and the Bavarian State Research Center for Agriculture. *Mitteilungen aus der Biologischen Bundesanstalt fuer Land- und Forstwirtschaft Berlin-Dahlem* 2006, 403:136-139.°**(Ex01)**
Laszlo B: The genetically modified (GM) plants and the honeybee (Apis mellifera) - Literature review. *Magyar Allatorvosok Lapja* 2005, 127(5):307-313.°**(Ex01)**
Lee MS, Albajes R: Butterflies for post market environmental monitoring of GM maize in Spain. *IOBC/WPRS Bulletin* 2013, 97:63-72.°**(Ex06)**
Lee MS, Albajes R: Monitoring carabid indicators could reveal environmental impacts of genetically modified maize. *Agricultural and Forest Entomology* 2016, 18(3):238-249.°**(Ex05)**
Lehman RM, Osborne SL, Rosentrater KA: No differences in decomposition rates observed between Bacillus thuringiensis and non-Bacillus thuringiensis corn residue incubated in the field. *Agronomy Journal* 2008, 100(1):163-168.°**(Ex03)**
Lehman RM, Osborne SL, Rosentrater KA: No Evidence That Bacillus thuringiensis Genes and Their Products Influence the Susceptibility of Corn Residue to Decomposition. *Agronomy Journal* 2008, 100(6):1687-1693.°**(Ex03)**
Lepping MD: Ground-dwelling beetles as bioindicators in transgenic corn. *Ph.D.* Ann Arbor: University of Maryland, College Park, USA; 2009.°**(Ex07)**
Leslie TW: Epigeal insect diversity and dynamics in agroecosystems adopting transgenic crops. *Ph.D.* Ann Arbor: The Pennsylvania State University, USA; 2009.°**(Ex02)**
Lheureux K, Menrad K: A decade of European field trials with genetically modified plants. *Environmental Biosafety Research* 2004, 3(2):99-107.°**(Ex01)**
Li ZL, Cui J, Mi ZR, Tian DS, Wang JS, Ma ZL, Wang BX, Chen HYH, Niu SL: Responses of soil enzymatic activities to transgenic Bacillus thuringiensis (Bt) crops - A global meta-analysis. *Science of the Total Environment* 2019, 651:1830-1838.°**(Ex01)**
Linn MD, Moore PA: The Effects of Bt Corn on Rusty Crayfish (Orconectes Rusticus) Growth and Survival. *Archives of Environmental Contamination and Toxicology* 2014, 67(3):436-443.°**(Ex03)**

Lit IL, Jr., Caasi-Lit MT, Benigno EA: In search of efficient and effective sampling methods for field evaluation of non-target organisms in Bt corn variety trials: a comparative study. *Philippine Entomologist* 2014, 28(2):171-193.°**(Ex02)**

Lorch A: Maize 1507: toxic and inadequately tested- Why GM maize 1507 should be banned. Brussels, Belgium: Friends of the Earth Europe; 2013: 5 pages.°**(Ex01)**

Lorch A, Cotter J: EFSA fails again: insect resistant GM Bt maize 1507 (C/ES/01/01) should not be grown in Europe. *Technical Note.* vol. 13. Exeter, UK: Greenpeace Research Laboratories 2005: 11 pages.°**(Ex01)**

Losey JE, Obrycki JJ, Hufbauer RA: Impacts of genetically engineered crops on non-target herbivores: fit-corn and monarch butterflies as a case study. In: *Genetically engineered organisms: assessing environmental and human health effects.* Edited by Letourneau DK, Burrows BE. Boca Raton: CRC Press LLC; 2001: 143-160.°**(Ex01)**

Lövei G, Bohn T, Hilbeck A: Biodiversity, Ecosystem Services and Genetically Modified Organisms. *TWN Biotechnology & Biosafety Series.* Third World Networt (TWN); 2010.°**(Ex01)**

Lozzia GC, Rigamonti IE: Preliminary observations on the arthropod fauna of transgenic corn fields. *Atti, Giornate fitopatologiche, Scicli e Ragusa* 1998:223-228.°**(Ex08)**

Lozzia GC, Rigamonti IE, Agosti M: Evaluation methods of the effects of transgenic corn on non target species. *Notiziario Sulla Protezione delle Plante* 1998, 8:27-39.°**(Ex02)**

Ludy C: A risk assessment of genetically modified organisms: Potential effects of Bt maize on spiders. *Ph.D.* University of Bremen, Germany; 2005.°**(Ex02)**

Ludy C, Lang A: Bt maize pollen exposure and impact on the garden spider, Araneus diadematus. *Entomologia Experimentalis Et Applicata* 2006, 118(2):145-156.°**(Ex03)**

Ludy C, Lang A, Meissle M: Monitoring of Bt maize and effects on populations of beneficials using spiders as an example. *DGaaE Nachrichten* 2003, 17(1):12.°**(Ex01)**

Lumbierres B, Albajes R, Pons X: Transgenic Bt maize and Rhopalosiphum padi (Hom., Aphididae) performance. *Ecological Entomology* 2004, 29(3):309-317.°**(Ex02)**

Lumbierres B, Lopez C, Albajes R: Abundance of non-target pests in transgenic Bt-maize: A farm scale study. *European Journal of Entomology* 2005, 102(1):73-79.°**(ExDUP)**

Malerbo-Souza DT, da Silva TG, de Andrade MO, de Farias LR, Medeiros NMG: Factors affecting the foraging behavior of bees in different maize hybrids. *Revista Brasileira De Ciencias Agrarias-Agraria* 2018, 13(3):8.°**(Ex06)**

Manachini B, Agosti M, Rigamonti I: Environmental impact of Bt-corn on non target entomofauna: Synthesis of field and laboratory studies. *11th Symposium on Pesticide Chemistry, Human and Environmental Exposure to Xenobiotics: Sep 11-15, 1999; Cremona, Italy*: Edited by DelRe AAM, Brown C, Capri E, Errera G, Evans SP, Trevisan M. WOS CAB: La Goliardica Pavese 1999: 873-882.°**(Ex02)**

Manila-Fajardo AC, Gonzales AKBM: A Multilayer, Large Scale Comparison of Arthropod Communities in Commercially Managed Bt and Non-Bt Corn Fields (vol 24, pg 150, 2010). *Philippine Entomologist* 2011, 25(1):110.°**(NO)**

Martin C, de Billot M: Phenotypic evaluation and ecological observations of coleopteran-protected and glyphosate-tolerant maize MON 88017 in Germany and Spain field trials during 2006. Monsanto Company; 2008: 67 pages.°**(ExPERM)**

Martins F, Vieira MM, Lavadinho AMP, Mendonça TR: Efeito de milho Bt sobre a entomofauna não alvo. *Revista de Ciências Agrárias* 2008, 31(2):29-33.°**(Ex08)**

Martins F, Vieira MM, Lavadinho AMP, Mendonca TR: Side-efect of maize Bt on non-target arthropods. *Revista de Ciências Agrárias* 2008, 31(2):29-33.°**(ExDUP)**

Marvier M: Ecology of Transgenic Crops Genetically engineered plants might generate weed problems and affect nontarget organisms, but measuring the risk is difficult. *American Scientist* 2001, 89:160-168.°**(Ex01)**

Marvier M: Improving risk assessment for nontarget safety of transgenic crops. *Ecological Applications* 2002, 12(4):1119-1124.°**(Ex01)**

McManus BL: Abundance of non-target coccinellids in corn rootworm-resistant maize. *M.S.* Ann Arbor: South Dakota State University, USA; 2004.°**(NO)**

Meissle M: Compatibility of biological control with Bt maize expressing Cry3Bb1 in controlling corn rootworms. *Ph.D.* Bern University, Switzerland; 2009.°**(Ex03)**

Meissle M, Naranjo SE, Kohl C, Riedel J, Romeis J: Does the growing of Bt maize change abundance or ecological function of non-target animals compared to the growing of non-GM maize? A systematic review protocol. *Environmental Evidence* 2014, 3(7).°**(Ex01)**

Midega CAO, Khan ZR, Van den Berg J, Ogol C, Pickett JA, Wadhams LJ: Maize stemborer predator activity under 'push-pull' system and Bt-maize: A potential component in managing Bt resistance. *International Journal of Pest Management* 2006, 52(1):1-10.°**(Ex08)**

Milius S: Bt Corn Variety OK for Black Swallowtails. *Science News* 2000, 157(24):372-373.°**(Ex01)**

Milne AE, Bell JR, Hutchison WD, van den Bosch F, Mitchell PD, Crowder D, Parnell S, Whitmore AP: The Effect of Farmers' Decisions on Pest Control with Bt Crops: A Billion Dollar Game of Strategy. *PLoS Computational Biology* 2015, 11(12):e1004483.°**(Ex01)**

Morse ME: Nontarget effects of Bt corn pollen on the monarch butterfly (U915536). EPA Science Inventory; 2007.°**(Ex01)**

Mota TA, Fernandes MG, Alegre EA, Sousa MFd, Tiago EF, Lourencao ALF: Can Bt maize change the spatial distribution of predator Cycloneda sanguinea (L.) (Coleoptera: Coccinelidae)? *African Journal of Biotechnology* 2013, 12(42):6086-6090.°**(Ex08)**

Naegeli H, Birch AN, Casacuberta J, De Schrijver A, Gralak MA, Guerche P, Jones H, Manachini B, Messean A, Nielsen EE *et al*: Assessment of genetically modified maize Bt11 x MIR162 x 1507 x GA21 and three subcombinations independently of their origin, for food and feed uses under Regulation (EC) No 1829/2003 (application EFSA-GMO-DE-2010-86). *EFSA Journal* 2018, 16(7):35.°**(Ex01)**
Naegeli H, Birch AN, Casacuberta J, De Schrijver A, Gralak MA, Guerche P, Jones H, Manachini B, Messéan A, Nielsen EE *et al*: Annual post-market environmental monitoring (PMEM) report on the cultivation of genetically modified maize MON 810 in 2015 from Monsanto Europe S.A. 2017.°**(ExDUP)**
Naegeli H, Birch AN, Casacuberta J, De Schrijver A, Gralak MA, Guerche P, Jones H, Manachini B, Messéan A, Nielsen EE *et al*: Assessment of genetically modified maize GA21 for renewal of authorisation under Regulation (EC) No 1829/2003 (application EFSA-GMO-RX-005). 2017.°**(Ex01)**
Naegeli H, Birch AN, Casacuberta J, De Schrijver A, Gralak MA, Guerche P, Jones H, Manachini B, Messean A, Nielsen EE *et al*: Assessment of genetically modified maize 4114 for food and feed uses, under Regulation (EC) No 1829/2003 (application EFSA-GMO-NL-2014-123). *EFSA Journal* 2018, 16(5):25.°**(Ex01)**
Naegeli H, Birch AN, Casacuberta J, De Schrijver A, Gralak MA, Guerche P, Jones H, Manachini B, Messean A, Nielsen EE *et al*: Scientific opinion on an application for renewal of authorisation for continued marketing of maize 1507 and derived food and feed submitted under Articles 11 and 23 of Regulation (EC) No 1829/2003 by Pioneer Overseas Corporation and Dow AgroSciences LLC. *EFSA Journal* 2017, 15(1):11.°**(Ex01)**
Naegeli H, Birch AN, Casacuberta J, De Schrijver A, Gralak MA, Guerche P, Jones H, Manachini B, Messean A, Nielsen EE *et al*: Scientific opinion on an application for renewal of authorisation for continued marketing of maize 59122 and derived food and feed submitted under articles 11 and 23 of Regulation (EC) No 1829/2003 by Pioneer Overseas Corporation and Dow AgroSciences LLC. *EFSA Journal* 2017, 15(6):10.°**(Ex01)**
Naegeli H, Birch AN, Casacuberta J, De Schrijver A, Gralak MA, Guerche P, Jones H, Manachini B, Messean A, Nielsen EE *et al*: Scientific Opinion on application EFSA-GMO-BE-2013-118 for authorisation of genetically modified maize MON87427xMON89034x1507x MON88017x59122 and subcombinations independently of their origin, for food and feed uses, import and processing submitted under Regulation (EC) No1829/2003 by Monsanto Company. *EFSA Journal* 2017, 15(8):32.°**(Ex01)**
Naegeli H, Birch AN, Casacuberta J, De Schrijver A, Gralak MA, Guerche P, Jones H, Manachini B, Messean A, Nielsen EE *et al*: Assessment of genetically modified maize MON 87411 for food and feed uses, import and processing, under Regulation (EC) No 1829/2003 (application EFSA-GMO-NL-2015-124). *EFSA Journal* 2018, 16(6):29.°**(Ex01)**
Naegeli H, Birch AN, Casacuberta J, De Schrijver A, Gralak MA, Guerche P, Jones H, Manachini B, Messean A, Nielsen EE *et al*: Assessment of genetically modified maize NK603 x MON810 for renewal of authorisation under Regulation (EC) No1829/2003 (application EFSA-GMO-RX-007). *EFSA Journal* 2018, 16(2):10.°**(Ex01)**
Naegeli H, Birch AN, Casacuberta J, De Schrijver A, Gralak MA, Guerche P, Jones H, Manachini B, Messéan A, Nielsen EE *et al*: Assessment of genetically modified maize NK603 x MON810 for renewal of authorisation under Regulation (EC) No 1829/2003 (application EFSA-GMO-RX-007). 2018.°**(ExDUP)**
Naegeli H, Birch AN, Casacuberta J, De Schrijver A, Gralak MA, Guerche P, Jones H, Manachini B, Messean A, Nielsen EE *et al*: Assessment of genetically modified maize MON 87403 for food and feed uses, import and processing, under Regulation (EC) No 1829/2003 (application EFSA-GMO-BE-2015-125). *EFSA Journal* 2018, 16(3):28.°**(Ex01)**
Naegeli H, Birch AN, Casacuberta J, De Schrijver A, Gralak MA, Guerche P, Jones H, Manachini B, Messean A, Nielsen EE *et al*: Scientific Opinion on an application by Dow AgroSciences (EFSA-GMO-NL-2013-116) for placing on the market of genetically modified insect-resistant soybean DAS-81419-2 for food and feed uses, import and processing under Regulation (EC) No 1829/2003. *EFSA Journal* 2016, 14(12):23.°**(Ex01)**
Naegeli H, Birch AN, Casacuberta J, De Schrijver A, Gralak MA, Guerche P, Jones H, Manachini B, Messéan A, Nielsen EE *et al*: Assessment of genetically modified maize 1507 × 59122 × MON810 × NK603 and subcombinations, for food and feed uses, under Regulation (EC) No 1829/2003 (application EFSA-GMO-NL-2011-92). 2017.°**(Ex01)**
Naegeli H, Birch AN, Casacuberta J, De Schrijver A, Gralak MA, Guerche P, Jones H, Manachini B, Messean A, Nielsen EE *et al*: Assessment of genetically modified maize 1507 x 59122 x MON810 x NK603 and subcombinations, for food and feed uses, under Regulation (EC) No1829/2003 (application EFSA-GMO-NL-2011-92). *EFSA Journal* 2017, 15(11):29.°**(Ex01)**
Naegeli H, Birch AN, Casacuberta J, De Schrijver A, Gralak MA, Guerche P, Jones H, Manachini B, Messéan A, Nielsen EE *et al*: Scientific Opinion on application EFSA-GMO-BE-2013-117 for authorisation of genetically modified maize MON 87427 × MON 89034 × NK603 and subcombinations independently of their origin, for food and feed uses, import and processing submitted under Regulation (EC) No 1829/2003 by Monsanto Company. 2017.°**(Ex01)**
Naegeli H, Birch AN, Casacuberta J, De Schrijver A, Gralak MA, Guerche P, Jones H, Manachini B, Messean A, Nielsen EE *et al*: Scientific Opinion on application EFSA-GMO-BE-2013-117 for authorisation of genetically modified maize MON87427xMON89034xNK603 and subcombinations independently of their origin, for food and feed uses, import and processing submitted under Regulation (EC) No1829/2003 by Monsanto Company. *EFSA Journal* 2017, 15(8):26.°**(Ex01)**
Naegeli H, Birch AN, Casacuberta J, Gmo E, Gralak MA, Guerche P, Jones H, Manachini B, Messean A, Nielsen EE *et al*: Assessment of genetically modified maize GA21 for renewal of authorisation under Regulation (EC) No1829/2003 (application EFSA-GMO-RX-005). *EFSA Journal* 2017, 15(10):11.°**(Ex01)**
Naegeli H, Birch AN, Casacuberta J, Schrijver Ad, Gralak MA, Guerche P, Jones H, Manachini B, Messean A, Nielsen EE *et al*: Scientific opinion on application EFSA-GMO-BE-2013-118 for authorisation of genetically modified maize MON 87427 x

MON 89034x1507 x MON 88017 x 59122 and subcombinations independently of their origin, for food and feed uses, import and processing submitted under regulation (EC) no 1829/2003 by Monsanto company. *EFSA Journal* 2017, 15(8).°**(Ex01)**
Naegeli H, Birch AN, Casacuberta J, Schrijver Ad, Gralak MA, Guerche P, Jones H, Manachini B, Messean A, Nielsen EE *et al*: Scientific opinion on application EFSA-GMO-BE-2013-117 for authorisation of genetically modified maize MON 87427 x MON 89034 x NK603 and subcombinations independently of their origin, for food and feed uses, import and processing submitted under regulation (EC) no 1829/2003 by Monsanto company. *EFSA Journal* 2017, 15(8).°**(Ex01)**
Naegeli H, Bresson JL, Dalmay T, Dewhurst IC, Epstein MM, Firbank LG, Guerche P, Hejatko J, Moreno FJ, Mullins E *et al*: Assessment of genetically modified maize MZHG0JG for food and feed uses, import and processing under Regulation (EC) No 1829/2003 (application EFSA-GMO-DE-2016-133). *EFSA Journal* 2018, 16(11):26.°**(Ex01)**
Nais J, Busoli AC, Michelotto MD: Behavior of transgenic maize hybrids and their conventional isogenic counterparts in relation to infestation of Spodoptera frugiperda (J. E. Smith, 1727) (Lepidoptera: Noctuidae) at two locations and sowing dates. *Arquivos do Instituto Biologico Sao Paulo* 2013, 80(2):159-167.°**(Ex04)**
Nakai S, Hoshikawa K, Yamane S, Shimono A, Ohsawa R: Example of an isolated field study and environmental risk assessment for GM corn in Japan. *Breeding Research* 2015, 17(1):1-15.°**(Ex03)**
Nakai S, Hoshikawa K, Shimono A, Ohsawa R: Transportability of confined field trial data from cultivation to import countries for environmental risk assessment of genetically modified crops. *Transgenic Research* 2015, 24(6):929-944.°**(Ex01)**
Ndemah R, Schulthess F, Nolte C: The effect of grassy field margins and fertilizer on soil water, plant nutrient levels, stem borer attacks and yield of maize in the humid forest zone of Cameroon. *Annales de la Societe Entomologique de France* 2006, 42(3-4):461-470.°**(Ex05)**
Nicolia A, Manzo A, Veronesi F, Rosellini D: An overview of the last 10 years of genetically engineered crop safety research. *Critical Reviews in Biotechnology* 2014, 34(1):77-88.°**(Ex01)**
Nijmeijer A: Environmental risks of Bt-maize and transgenic drought tolerant maize. *Thesis* Utrecht University, The Netherlands; 2013.°**(Ex01)**
Ortego F, Farinos GP, Hernandez-Crespo P, Castanera P: Post- market monitoring programmes for Bt-maize in Spain. *Entomological Research* 2007, 37(Suppl. 1):A48-A49.°**(Ex01)**
Palinkas Z, Zalai M, Szenasi A, Dorner Z, Kiss J, North S, Woodward G, Balog A: Arthropods dataset from different genetically modified maize events and associated controls. *Scientific Data* 2018, 5:6.°**(Ex02)**
Palinkas Z, Zalai M, Szenasi A, Kadar F, Dorner Z, Balog A: Rove beetles (Coleoptera Staphylinidae)-Their abundance and competition with other predatory groups in Bt maize expressing Cry34Ab1, Cry35Ab1, Cry1F and CP4 EPSPS proteins. *Crop Protection* 2016, 80:87-93.°**(Ex02)**
Palizada SA, Tiroesele B, Kondidie DB, Ullah MI, Mustafa F, Hunt TE, Clark PL, Molina-Ochoa J, Skoda SR, Foster JE: Minute pirate bug (Orius insidiosus Say) populations on transgenic and non-transgenic maize using different sampling techniques. *Pakistan Entomologist* 2014, 36(1):1-6.°**(ExDUP)**
Pascual A: Monitoring of Key Non-Target Arthropods in Transgenic Maize Lines Containing Event DAS-59122-7, and the Combined Trait Products DAS-Ø15Ø7-1x DAS-59122-7, DAS-Ø15Ø7-1x MON-ØØ6Ø3-6, and DAS-59122-7x DAS-Ø15Ø7-1x MONØØ6Ø3-6: 2008 Hungary Field Study. Pioneer Hi-Bred International; 2010: 141 pages.°**(Ex02)**
Pascual A, Hong B: Monitoring of Key Non-Target Arthropods in Transgenic Maize Lines Containing Event DAS-59122-7, and the Combined Trait Products DAS-Ø15Ø7-1x DAS-59122-7, DAS-Ø15Ø7-1x MON-ØØ6Ø3-6, and DAS-59122-7x DAS-Ø15Ø7-1x MONØØ6Ø3-6: 2007 European Field Studies (Hungary location). Pioneer Hi-Bred International; 2008: 66 pages.°**(Ex02)**
Pascual A, Hong B: Monitoring of Key Non-Target Arthropods in Transgenic Maize Lines Containing Event DAS-59122-7, and the Combined Trait Products DAS-Ø15Ø7-1x DAS-59122-7, DAS-Ø15Ø7-1x MON-ØØ6Ø3-6, and DAS-59122-7x DAS-Ø15Ø7-1x MONØØ6Ø3-6: 2007 European Field Studies (Spain location). Pioneer Hi-Bred International; 2008: 91 pages.°**(ExPERM)**
Pearsons KA, Tooker JF: In-Field Habitat Management to Optimize Pest Control of Novel Soil Communities in Agroecosystems. *Insects* 2017, 8(3):14.°**(Ex01)**
Perry JN: The effect of BT-maize on butterflies - reckoning the risk. Outlooks on Pest Management 2011, October:1-7.°**(Ex03)**
Peterson JA: Delineating the influence of genetically modified crops and non-prey food resources on generalist predator food webs. *Ph.D.* Ann Arbor: University of Kentucky, USA; 2012.°**(Ex07)**
Peterson RKD, Meyer SJ, Wolf AT, Wolt JD, Davis PM: Genetically engineered plants, endangered species, and risk: A temporal and spatial exposure assessment for Karner blue butterfly larvae and Bt maize pollen. *Risk Analysis* 2006, 26(3):845-858.°**(Ex03)**
Phillips SL, Sammons B, Clark PL: Phenotypic evaluation and ecological observation of MON 89034 x NK603 in Argentina field trials during 2004/2005. Monsanto Company; 2006: 47 pages.°**(ExPERM)**
Pilcher CD, Rice ME, Obrycki JJ: Impact of transgenic Bacillus thuringiensis corn and crop phenology on five nontarget arthropods. *Environmental Entomology* 2005, 34(5):1302-1316.°**(Ex02)**
Pilcher CD, Rice ME, Obrycki JJ, Lewis LC: Field and laboratory evaluations of transgenic Bacillus thuringiensis corn on secondary lepidopteran pests (Lepidoptera: Noctuidae). *Journal of Economic Entomology* 1997, 90(2):669-678.°**(Ex03)**
Pimentel DS, Raven PH: Bt corn pollen impacts on nontarget Lepidoptera: Assessment of effects in nature. *Proceedings of the National Academy of Sciences* 2000, 97(15):8198-8199.°**(Ex01)**
Poletika NN: Non-target invertebrate ecological risk assessment for field corn expressing Cry34Ab1 and Cry35Ab1 insecticidal

crystal proteins in event DAS-59122-7. Dow AgroSciences; 2003: 101 pages.°**(Ex01)**
Pons X, Stary P: Spring aphid-parasitoid (Hom., Aphididae, Hym., Braconidae) associations and interactions in a Mediterranean arable crop ecosystem, including Bt maize. *Journal of Pest Science* 2003, 76:133-138.°**(Ex08)**
Prasifka JR, Hellmich RL, Dively GP, Higgins LS, Dixon PM, Duan JJ: Selection of nontarget arthropod taxa for field research on transgenic insecticidal crops: Using empirical data and statistical power. *Environmental Entomology* 2008, 37(1):1-10.°**(Ex01)**
Prasifka PL, Hellmich RL, Prasifka JR, Lewis LC: Effects of Cry1Ab-expressing corn anthers on the movement of monarch butterfly larvae. *Environmental Entomology* 2014, 36(1):228-233.°**(Ex03)**
Pretorius D, Engelbrecht E, van den Berg J: Diversity and comparitive phenology of lepidoptera on Bt and non-Bt maize at twelve sites in the North West and Limpopo provinces, South Africa. *16th Entomological Congress: July 05 -07, 2009; Stellenbosch, South Africa*: Edited by Terblanche J, Venter R.: Entomological Society of Southern Africa 2009: 61.°**(Ex01)**
Priesnitz KU, Benker U, Schaarschmidt F: Assessment of the potential impact of a Bt maize hybrid expressing Cry3Bb1 on ground beetles (Carabidae). *Journal of Plant Diseases and Protection* 2013, 120(3):131-140.°**(Ex02)**
Rajan, Khetarpal RK: Nematode community for 'non-target safety' studies on transgenic crops. *Indian Journal of Plant Protection* 2007, 35(2):194-201.°**(Ex01)**
Ramirez-Romero R, Desneux N, Chaufaux J, Kaiser L: Bt-maize effects on biological parameters of the non-target aphid< i> Sitobion avenae</i>(Homoptera: Aphididae) and Cry1Ab toxin detection. *Pesticide Biochemistry and Physiology* 2008, 91(2):110-115.°**(Ex03)**
Rauschen S: Biosafety research into Diabrotica-resistant Bt-maize: Indicator organisms and monitoring methods. 2013.°**(ExDUP)**
Rauschen S, Eckert J, Gathmann A, Schuphan I: Impact of growing Bt-maize on cicadas: diversity, abundance and methods. *IOBC-WPRS Bulletin* 2004, 27(3):137-142.°**(Ex02)**
Rauschen S, Schaarschmidt F, Gathmann A: Occurrence and field densities of Coccinellidae in the maize herb layer: implications for environmental risk assessment. *IOBC/WPRS Bulletin* 2010, 52:85-90.°**(Ex02)**
Rauschen S, Schaarschmidt F, Gathmann A: Occurrence and field densities of Coleoptera in the maize herb layer: implications for Environmental Risk Assessment of genetically modified Bt-maize. *Transgenic Research* 2010, 19(5):727-744.°**(Ex02)**
Rauschen S, Schultheis E, Hunfeld H, Schaarschmidt F, Schuphan I, Eber S: Diabrotica-resistant Bt-maize DKc5143 event MON88017 has no impact on the field densities of the leafhopper Zyginidia scutellaris. *Environmental Biosafety Research* 2010, 9(2):87-99.°**(Ex02)**
Rauschen S, Schultheis E, Pagel-Wieder S, Schuphan I, Eber S: Impact of Bt-corn MON88017 in comparison to three conventional lines on Trigonotylus caelestialium (Kirkaldy) (Heteroptera: Miridae) field densities. *Transgenic Research* 2009, 18(2):203-214.°**(Ex02)**
Rauschen S, Schuphan I, Eber S: Assessment of possible non-target impacts of the novel Bt-maize event MON88017 resistant against the Western Corn Rootworm Diabrotica virgifera virgifera (LeConte). *IOBC/WPRS Bulletin* 2008, 33:93-100.°**(Ex02)**
Ren Z, Shen W, Liu B, Xue K: Effects of transgenic maize on biodiversity of arthropod communities in the fields. *Scientia Agricultura Sinica* 2017, 50(12):2315-2325.°**(Ex02)**
Resende DC, Mendes SM, Marucci RC, Silva AD, Campanha MM, Waquil JM: Does Bt maize cultivation affect the non-target insect community in the agro ecosystem? *Revista Brasileira De Entomologia* 2016, 60(1):82-93.°**(Ex08)**
Reyes SG: Assessment of insect communities in Bt- and non Bt-corn from Carulay, Echague, Isabela [Philippines]: wet season. *33rd Anniversary & Annual Scientific Meeting of the Pest Management Council of the Philippines: 8-10 May 2002; Davao City, Philippines*. Pest Management Council of the Philippines Inc. 2002: 97.°**(Ex01)**
Reyes SG: Insect species diversity and population abundance of selected natural enemies in Bt corn and non-Bt corn agroecosystems. *Philippine Entomologist* 2003, 17(2):194.°**(Ex01)**
Reyes SG, Jovillano-Mostoles MDA: XDiversity, community structure and wet season population abundance of insectsin Bt-corn agroecosystem in two sites on Luzon Island, Philippines. *Asia Life Sciences* 2005, 14(1):55-73.°**(ExDUP)**
Riedel J, Romeis J, Meissle M: Update and expansion of the database of bio-ecological information on non-target arthropod species established to support the environmental risk assessment of genetically modified crops in the EU. *EFSA Supporting Publications* 2016, 13(1).°**(Ex01)**
Rojas J, Gomez JR, Barreda A: Control de Spodoptera frugiperda (J. E. Smith) con diferentes medios biologicos. *Centro Agricola* 2001, 28(2):19-22.°**(Ex03)**
Romeis J, Bartsch D, Bigler F, Candolfi MP, Gielkens MMC, Hartley SE, Hellmich RL, Huesing JE, Jepson PC, Layton R *et al*: Assessment of risk of insect-resistant transgenic crops to nontarget arthropods. *Nature Biotechnology* 2008, 26(2):203-208.°**(Ex01)**
Romeis J, Meissle M, Alvarez-Alfageme F, Bigler F, Bohan DA, Devos Y, Malone LA, Pons X, Rauschen S: Potential use of an arthropod database to support the non-target risk assessment and monitoring of transgenic plants. *Transgenic Research* 2014, 23(6):995-1013.°**(Ex01)**
Rosca I: Preliminary research regarding studying schema of non target organisms in genetically modified crops. *Lucrari Stiintifice Universitatea de Stiinte Agronomice Bucuresti Seria A, Agronomie* 2008, 51:763-768.°**(Ex01)**
Rosca I: Impact of genetically, modified corn on arthropod communities. *Bulletin OILB/SROP* 2006, 29(5):137-141.°**(Ex08)**
Rosca I, Cagan L: Research on the influence of genetically modified maize on the Coccinellidae fauna. *IOBC/WPRS Bulletin* 2012, 73:83-88.°**(Ex08)**
Rosca I, Cagan L: Research on the influence of genetically modified maize on the Neuroptera fauna. *IOBC/WPRS Bulletin* 2012,

73:89-94.°**(Ex08) Source: CAB**
Rosca I, Cagan L: Research on syrphid fauna from different maize hybrids. *Romanian Agricultural Research* 2013, 30:297-305.°**(924 N1) Source: WOS CAB SCO**
Rosca I, Popov C: Key species for evaluation of biodiversity, in main crop field, for measurement the influence of genetic modified organisms on agroecosystem. *Entomological Research* 2007, 37(Suppl. 1):A125-A126.°**(Ex01)**
Rose R, Dively GP, Pettis J: Effects of Bt corn pollen on honey bees: emphasis on protocol development. *Apidologie* 2007, 38(4):368-377.°**(Ex03)**
Rozman L, Gomboc S: A review of potential hazards of deliberately released Bt maize into the environment. *Zbornik Biotehniske fakultete Univerze* 2002:127-141.°**(Ex01)**
Rudeen ML: Tritrophic interactions among larval western corn rootworm, Bt corn and entomopathogens. *M.S.* Ann Arbor: Iowa State University, USA; 2010.°**(Ex03)**
Rüdelsheim PLJ, Smets G: Baseline information on agricultural practices in the EU Maize (Zea mays L.) Perseus BVBA; 2011: 66 pages.°**(Ex05)**
Rule DM, Nolting SP, Prasifka PL, Storer NP, Hopkins BW, Scherder EF, Siebert MW, Hendrix WH: Efficacy of Pyramided Bt Proteins Cry1F, Cry1A.105, and Cry2Ab2 Expressed in SmartStax Corn Hybrids Against Lepidopteran Insect Pests in the Northern United States. *Journal of Economic Entomology* 2014, 107(1):403-409.°**(Ex04)**
Sammons B, Leafgren R: Phenotypic evaluation and ecological observation of MON 89034 x MON 88017 in U.S. field trials during 2004 for an assessment of equivalence and weed potential. Monsanto Company; 2006: 53 pages.°**(ExPERM)**
Saxena D, Stotzky G: Bacillus thuringiensis (Bt) toxin released from root exudates and biomass of Bt corn has no apparent effect on earthworms, nematodes, protozoa, bacteria, and fungi in soil. *Soil Biology & Biochemistry* 2001, 33(9):1225-1230.°**(Ex03)**
Schaafsma AW, Holmes ML, Whistlecraft J, Dudley SA: Effectiveness of three Bt corn events against feeding damage by the true armyworm (Pseudaletia unipuncta Haworth). *Canadian Journal of Plant Science* 2007, 87(3):599-603.°**(Ex03)**
Schmidt JEU, Hilbeck A: Ecology of transgenic crop plants expressing insecticidal Bt ∂-endotoxins - Effects on trophic interactions and biodiversity of insect pollinators, non-target herbivores and natural enemies. *Bulletin of the Geobotanical Institute ETH* 2001, 67:79-87.°**(Ex01)**
Schmitz G, Bartsch D, Pretscher P: Selection of relevant non-target herbivores for monitoring the environmental effects of Bt maize pollen. *Environmental Biosafety Research* 2003, 2(2):117-132.°**(Ex03)**
Schori A: Agroscope Changins-Wadenswil research station reports in 2009 on genetically modified corn trials. *Revue Suisse D Agriculture* 2008, 40(3):116-116.°**(Ex01)**
Schultheis E, Slusarenko AJ, Rauschen S: The community of herbivorous arthropods as an indicator for biological control function in the Post Market Environmental Monitoring of the cultivation of stacked Bt maize. *4th International Workshop on Post Market Environmental Monitoring of Genetically Modified Plants: 3-4 May 2010; Julius Kühn-Institut (JKI), Quedlinburg, Germany*: Poster available from Nature Precedings 2010.°**(Ex02)**
Schuphan I, Eckert J, Gathmann A, Ross-Nickoll M, Toschki A: Verbundprojekt: Sicherheitsforschung und Monitoringmethoden zum Anbau von Bt-Mais, Teilprojekt 1.2.1: Effekte des Anbaus von Bt-Mais auf die epigäische und die Krautschichtfauna verschiedener trophischer Bezüge. 2005.°**(Ex02)**
Schuppener M: Risikobewertung von gentechnisch verändertem Mais im Hinblick auf ausgewählte Schmetterlinge der Agrarlandschaft ; Risk assessment of genetically engineered maize with regard to selected butterflies in the agricultural landscape. *Ph.D.* RWTH Aachen University, Germany; 2011.°**(Ex05)**
Schuppener M, Muhlhause J, Muller AK, Rauschen S: Environmental risk assessment for the small tortoiseshell Aglais urticae and a stacked Bt-maize with combined resistances against Lepidoptera and Chrysomelidae in central European agrarian landscapes. *Molecular Ecology* 2012, 21(18):4646-4662.°**(Ex05)**
Schuppener M, Slusarenko AJ, Rauschen S: Potential effects of pollen from stacked Bt maize on non-target Lepidoptera in agrarian systems. *4th International Workshop on Post Market Environmental Monitoring of Genetically Modified Plants: 3-4 May 2010; Julius Kühn-Institut (JKI), Quedlinburg, Germany*. Poster available from Nature Precedings 2010.°**(Ex02)**
Sears MK: Bt corn pollen and its effects on monarch butterfly populations. *California Conference on Biological Control III: 15-16 August, 2002; Berkeley, USA*: Edited by Hoddle MS. Center for Biological Control, College of Natural Resources, University of California 2002: 23-30.°**(Ex01)**
Sears MK: Impact of Bacillus thuringiensis corn pollen on monarch butterfly populations: A risk assessment. *Agricultural Biotechnology: Challenges and Prospects.* Edited by Bhalgat MK, Ridley WP, Felsot AS, Seiber JN, vol. 866. Washington: Amer Chemical Soc.; 2004: 125-137.°**(Ex01)**
Sehnal F, Habustova O, Hany HM, Zdenka S: Long-term monitoring of environmental impact of GM maize. *Current Opinion in Biotechnology* 2011, 22:S73-S74.°**(Ex01)**
Sharma HC, Romeis J, Dhillon MK: Deployment of transgenic crops for pest management and their effects on non-target natural enemies of crop pests. *Entomological Research* 2007, 37(Suppl. 1):A49.°**(Ex01)**
Shirai Y, Takahashi M: Effects of transgenic Bt corn pollen on a non-target lycaenid butterfly, Pseudozizeeria maha. *Applied Entomology and Zoology* 2005, 40(1):151-159.°**(Ex03)**
Shu Y, Zhang Y, Zeng H, Zhang Y, Wang J: Effects of Cry1Ab Bt maize straw return on bacterial community of earthworm Eisenia fetida. Elsevier, 173, 1-13. *Chemosphere* 2017, 173:1-13.°**(Ex03)**
Silva GA, Santos IB, Campos SO, Galdino TVS, Morais EGF, Martins JC, Ferreira LR, Guedes RNC, Picanco MC: Spatial distribution and losses by grain destroying insects in transgenic corn expressing the toxin Cry1 Ab. *Plos One* 2018,

13(8):14.°**(Ex08)**
Singh AK, Dubey SK: Current trends in Bt crops and their fate on associated microbial community dynamics: a review. *Protoplasma* 2016, 253(3):663-681.°**(Ex01)**
Singh AK, Dubey SK: Transgenic Plants and Soil Microbes. In: Current Developments in Biotechnology and Bioengineering: Crop Modification, Nutrition, and Food Production, Edited by Dubey SK, Pandey A, Sangwan RS. Amsterdam: Elsevier Science Bv; 2017.°**(Ex01)**
Sisterson MS, Carriere Y, Dennehy TJ, Tabashnik BE: Nontarget effects of transgenic insecticidal crops: Implications of source-sink population dynamics. *Environmental Entomology* 2007, 36(1):121-127.°**(Ex01)**
Smith JF, Luttrell RG, Greene JK: Seasonal abundance of stink bugs (Heteroptera : Pentatomidae) and other polyphagous species in a multi-crop environment in south Arkansas. *Journal of Entomological Science* 2008, 43(1):1-12.°**(Ex06)**
Solante RN: Arthropod profile and incidence of Asian Corn Borer, Ostrinia furnacalis (Guenee) (Lepidoptera:Pyralidae), in corn in Mambusao, Capiz [Philippines]. *Thesis* Philippines University, Los Banos, College, Laguna (Philippines); 2010.°**(NO)**
Song YY, Liu RY, Wang MF, Liu MQ, Liu XH, Ge F, Chen FJ: Effects of transgenic Bt rice lines with single Cry1Ab and fused Cry1Ab/Cry1Ac on the abundance dynamics and community diversity of soil mites. *Archives of Agronomy and Soil Science* 2019:14.°**(Ex05)**
Spendeler L, Carrasco J-F: The impact of GM corn in Spain - A report by Greenpeace and Friends of the Earth. Greenpeace and Friends of the Earth; 2003: 33 pages.°**(Ex01)**
Spitzer L, Ruzicka V, Hussein HM, Habustova O, Sehnal F: Expression of a Bacillus thuringiensis toxin in maize does not affect epigeic communities of carabid beetles and spiders. *Acta Fytotechnica et Zootechnica* 2004, 7(4):110-112.°**(Ex02)**
Spök A, Eckerstorfer M, Heissenberger A, Gaugitsch H: Risk Assessment of "stacked events" - Untersuchungen zur Risikoabschätzung von "Stacked Events". In: *Forschungsberichte der Sektion IV.* vol. 2. Wien: Bundesministerium für Gesundheit, Familie und Jugend; 2007: 68 pages.°**(Ex01)**
Spök A, Dolezel M, Eckerstorfer M, Freigassner M, Gaugitsch H, Heissenberger A, Karner S, Klade M, Proksch M, Schneider L, Treiber F, Uhl M: Assessment of Toxic and Ecotoxic Properties of Novel Proteins in GMO's, Teil 1. In: *Forschungsberichte der Sektion IV.* vol. 1. Wien: Bundesministerium für Gesundheit, Familie und Jugend; 2008: 193 pages.°**(Ex01)**
Spök A, Dolezel M, Eckerstorfer M, Freigassner M, Gaugitsch H, Heissenberger A, Karner S, Klade M, Proksch M, Schneider L, Treiber F, Uhl M: Assessment of Toxic and Ecotoxic Properties of Novel Proteins in GMO's, Teil 2 Supplement. In: *Forschungsberichte der Sektion IV.* vol. 1. Wien: Bundesministerium für Gesundheit, Familie und Jugend; 2008: 76 pages.°**(Ex01)**
Stastna P, Bezdek J: Beetle families (Coleoptera) from pitfall traps in different agricultural crops of Zabcice during 1999-2001. *Acta Universitatis Agriculturae et Silviculturae Mendelianae Brunensis* 2002:19-24.°**(Ex05)**
Stenoien C, Nail KR, Zalucki JM, Parry H, Oberhauser KS, Zalucki MP: Monarchs in decline: a collateral landscape-level effect of modern agriculture. *Insect Science* 2018, 25(4):528-541.°**(Ex01)**
Svobodova Z, Habustova O, Hussein HM, Puza V, Sehnal F: Impact of genetically modified maize expressing Cry3Bb1 on non-target arthropods: first year results of a field study. *IOBC/WPRS Bulletin* 2012, 73:107-120.°**(Ex02)**
Svobodova Z, Habustova OS, Hutchison WD, Hussein HM, Sehnal F: Risk Assessment of Genetically Engineered Maize Resistant to Diabrotica spp.: Influence on Above-Ground Arthropods in the Czech Republic. *Plos One* 2015, 10(6):16.°**(ExDUP)**
Świątek M, Kiełkiewicz M, Zagdańska B: Insect-resistant Bt-maize response to the short-term non-target mite-pest infestation and soil drought. *Acta Physiologiae Plantarum* 2014, 36(10):2705-2715.°**(Ex03)**
Szekeres D: Assessment of the impact of Bt (Mon 810, cry1ab) maize on selected non-target Coleoptera (Carabidae, Coccinellidae). Gödöllö, Hungary: *Ph.D.* Szent István University, Hungary; 2011.°**(Ex02)**
Szekeres D, Kadar F, Dorner Z: Ground beetles (Coleoptera: Carabidae) in transgenic herbicide tolerant maize hybrids: impact of the transgenic crop or the weed control practice? *IOBC/WPRS Bulletin* 2008, 33:105-110.°**(Ex06)**
Szekeres D, Kadar F, Kiss J: Ground beetle (Coleoptera, Carabidae) assemblages in Bt- (Cry1Ab, MON 810) and isogenic maize plots in Hungary. *Novenyvdelem* 2006, 42(7):357-363.°**(Ex02)**
Szekeres D, Kadar F, Szentkiralyi F, Kiss J: Structural characteristics and seasonal dynamics of ground beetle (Coleoptera, Carabidae) assemblages in experimental Bt and isogenic maize fields in Hungary. *Biodiversity implications on genetically modified plants: 2003; Monte Verità, Ascona, Switzerland, September 7-12 2003.* 26.°**(Ex01)**
Then C: Agro-Biotechnology: New plant pest caused by genetically engineered corn - The spread of the western bean cutworm causes massive damage in the US. Testbiotech; 2010: 22 pages.°**(Ex01)**
Then C: Risk assessment of toxins derived from Bacillus thuringiensis—synergism, efficacy, and selectivity. *Environ Sci Pollut Res* 2010, 17:791-797.°**(Ex01)**
Then C: 30 years of genetically engineered plants - 20 years of commercial cultivation in the United States: a critical assessment. Testbiotech; 2013: 48 pages.°**(Ex01)**
Then C, Bauer-Panskus A: How industry and EFSA have been systematically undermining the risk assessment of 'SmartStax'. TestBiotech; 2011: 18 pages.°**(Ex01)**
Then C, Bauer-Panskus A: Testbiotech comment on EFSA Panel on Genetically Modified Organisms (GMO); Scientific Opinion on an application (EFSA GMO-NL-2012-107) for the placing on the market of maize MON 810 pollen under Regulation (EC) No 1829/2003 from Monsanto. Testbiotech; 2013: 3 pages.°**(Ex01)**
Then C, Bauer-Panskus A: Flaws in EFSA´s risk assessment: Examples from eight genetically engineered crops. TestBiotech;

2014: 9 pages.°**(Ex01)**
Then C, Bauer-Panskus A: Genetically engineered Maize 1507: EFSA cannot invalidate evidence of substantial gaps in risk assessment. TestBiotech; 2014: 7 pages.°**(Ex01)**
Tian J-C, Yao J, Long L-P, Romeis J, Shelton AM: Bt crops benefit natural enemies to control non-target pests. *Scientific Reports* 2015, 5:16636.°**(Ex03)**
Toscano LC, Fernandes MA, Rota MS, Maruyama WI, Andrade JV: Maize hybrids against the attack of Spodoptera frugiperda in association with silicone fertilization and the effect on the predator Doru luteipes. *Revista De Agricultura Neotropical* 2016, 3(1):51-55.°**(Ex06)**
Toschki A: Suitability of different monitoring methods applied as basis in Risk Assessment for arable landscapes : using a biocoenological serial investigation and a plot design study as examples. *Ph.D.* RWTH Aachen University, Germany; 2008.°**(Ex02)**
Traxler A, Gollmann G, Pascher K, Riegler M, Stauffer C: Ökologische Risikoabschätzung von gentechnisch veränderten Pflanzen. In: *Forschungsberichte der Sektion IV.* vol. 10. Wien: Bundesministerium für Gesundheit und Frauen; 1998: 128 pages.°**(Ex01)**
Truter J, Van Hamburg H, Van Den Berg J: Comparative Diversity of Arthropods on Bt Maize and Non-Bt Maize in two Different Cropping Systems in South Africa. *Environmental Entomology* 2014, 43(1):197-208.°**(ExDUP)**
Truter J-M, van Hamburg H, van den Berg J: Arthropod diversity on genetically modified Bt and non-Bt maize. *16th Entomological Congress July 05 -07, 2009; Stellenbosch, South Africa*: Edited by Terblanche J, Venter R. Entomological Society of Southern Africa 2009: 74.°**(Ex01)**
Twardowski JP, Beres P, Hurej M, Klukowski Z: Ground beetles (Col., Carabidae) in Bt-maize - preliminary results from the first large scale field experiment in Poland. *IOBC/WPRS Bulletin* 2010, 52:97-102.°**(Ex02)**
Ullah I, Asif M, Ranjha MH, Iftikhar R, Ullah M, Khan NM, Ashfaq M: Biosafety risk assessment approaches for insect-resistant genetically modified crops. *Advancements in Life Sciences* 2017, 4(2):39-46.°**(Ex05)**
Van Den Berg J, Warren J, Du Plessis H: The potential effect of Bt maize on Chrysoperla pudica (Neuroptera: Chrysopidae). *Environmental Entomology* 2017, 46(2):413-417.°**(Ex03)**
Van der Voet H, Goedhart PW: The power of statistical tests using field trial count data of nontarget organisms in environmental risk assessment of genetically modified plants. *Agricultural and Forest Entomology* 2015, 17(2):164-172.°**(Ex01)**
Van Wyk A: Comparative phenology of Lepidoptera on genetically modified BT- and non-BT maize. *Ph.D.* North-West University, South Africa; 2006.°**(Ex02)**
Van Wyk A, Van den Berg J, Van Hamburg H: Selection of non-target Lepidoptera species for ecological risk assessment of Bt maize in South Africa. *African Entomology* 2007, 15(2):356-366.°**(Ex03)**
Van Wyk A, Van den Berg J, Van Hamburg H: Diversity and comparative phenology of Lepidoptera on Bt and non-Bt maize in South Africa. *International Journal of Pest Management* 2008, 54(1):77-87.°**(Ex08)**
Villanyi I, Bakonyi G, Biro B: Effects of genetic modification on the decomposition of corn residues measured by litter-bag method. *Cereal Research Communications* 2008, 36:475-478.°**(Ex03)**
Virla EG, Casuso M, Frias EA: A preliminary study on the effects of a transgenic corn event on the non-target pest Dalbulus maidis (Hemiptera: Cicadellidae). *Crop Protection* 2010, 29(6):635-638.°**(Ex08)**
Volkmar C, Freier B: Spider communities in Bt. maize and not genetically modified maize fields. *Zeitschrift Fur Pflanzenkrankheiten Und Pflanzenschutz-Journal of Plant Diseases and Protection* 2003, 110(6):572-582.°**(Ex08)**
Volkmar C, Freier B, Wieacker K, Wendt C: Variation of spider pitfall trappings in conventional and Bt maize fields and its uses for the evaluation of a good ecological state. *Mitteilungen der Deutschen Gesellschaft fuer Allgemeine und Angewandte Entomologie* 2009, 17:125-129.°**(Ex02)**
Volkmar C, Freir C, Juen A, Traugott M, Schorlling M: Results of a 4-year plant survey and pitfall trapping in Bt maize and conventional maize fields regarding the occurrence of selected arthropod taxa. *Schweizerische Entomologische Gesellschaft, Mitteilungen* 2004, 27(3):78-84.°**(NO)**
Volkmar C, Freir C, Juen A, Traugott M, Schorlling M: Spider communities in Bt maize and conventional maize fields. *Schweizerische Entomologische Gesellschaft, Mitteilungen* 2004, 27(3):165-170.°**(NO)**
Volkmar C, Hussein MLA, Wetzel T: Ecological field studies in transgenic maize at Friemar (Thuringia). *Zeitschrift Fur Pflanzenkrankheiten Und Pflanzenschutz-Journal of Plant Diseases and Protection* 2004:1017-1024.°**(Ex05)**
Volkmar C, Traugott M, Juen A, Schorling M, Freier B: Spider communities in Bt maize and conventional maize fields. *IOBC-WPRS Bulletin* 2004, 27(3):165-170.°**(Ex02)**
Volkmar C, Wetzel T, Hussein MLA, Jany D, Richter L: Ecological studies accompanying the cultivation of transgenic maize plants in Friemar (Free State of Thuringia) from 1994 to 1996. *Archives of Phytopathology and Plant Protection* 1999, 32(4):291-335.°**(Ex05)**
Wagner DL, Peacock J, Carter JL, Talley SE: Field assessment of Bacillus thuringiensis on nontarget Lepidoptera. *Environmental Entomology* 1996, 25(6):1444-1454.°**(Ex05)**
Wang B-F, Song X-Y, Chang L, Wu D-H: Effect of transgenic corn (C63-1) cultivation on soil Collembola. *Chinese Journal of Applied Entomology* 2014, 51(5):1215-1221.°**(Ex05)**
Wang CL, Lin FC, Lin CY: Insect-resistant transgenic plants and the environmental impact assessment - special concern for insects. *Plant Protection Bulletin (Taichung)* 2004, 46(3):181-209.°**(Ex01)**
Wang F, Peng S, Cui K, Nie L, Huang J: Field performance of'Bt'transgenic crops: A review. *Australian Journal of Crop Science*

2014, 8(1):18.°**(Ex01)**
White JA, Andow DA: Host-parasitoid interactions in a transgenic landscape: Spatial proximity effects of host density. *Environmental Entomology* 2005, 34(6):1493-1500.°**(Ex03)**
Wilde GE, Ahmad A: No adverse affect of Coleopteran-specific Cry3bb1 toxin from transgenic corn pollen on Coleomegilla maculata (DeGeer). *Journal of Insect Science (Tucson)* 2003, 3(33 Cited October 24, 2003):27-28.°**(Ex01)**
Williams IH: Cultivation of GM crops in the EU, farmland biodiversity and bees. *Bee World* 2002, 83(3):119-133.°**(Ex01)**
Wohlfender-Buhler D, Feusthuber E, Wager R, Mann S, Aubry SJ: Genetically modified crops in Switzerland: implications for agrosystem sustainability evidenced by multi-criteria model. *Agronomy for Sustainable Development* 2016, 36(2):16.°**(Ex01)**
Wolt JD, Hellmich RL, Prasifka JR, Sears MK: Global regulatory perspectives regarding transgenic crop risks to nontarget insects: the case of Cry1F maize and butterflies. *Bulletin OILB/SROP* 2006, 29(5):187-194.°**(Ex01)**
Wolt JD, Peterson RKD, Bystrak P, Meade T: A screening level approach for nontarget insect risk assessment: Transgenic Bt corn pollen and the monarch butterfly (Lepidoptera : Danaidae). *Environmental Entomology* 2003, 32(2):237-246.°**(Ex01)**
Xie W, Ali T, Cui Q, Huang J: Economic impacts of commercializing insect-resistant GM maize in China. (Special Issue: Genetically engineered (GE) technology development, impact and policy.). *China Agricultural Economic Review* 2017, 9(3):340-354.°**(Ex01)**
Xing Y, Qin Z, Feng M, Li A, Zhang L, Wang Y, Dong X, Zhang Y, Tan S, Shi W: The impact of Bt maize expressing the Cry1Ac protein on non-target arthropods. *Environmental science and pollution research international* 2019, 26(6):5814-5819.°**(ExDUP)**
Zangerl AR, McKenna D, Wraight CL, Carroll M, Ficarello P, Warner R, Berenbaum MR: Effects of exposure to event 176 Bacillus thuringiensis corn pollen on monarch and black swallowtail caterpillars under field conditions. *Proceedings of the National Academy of Sciences of the United States of America* 2001, 98(21):11908-11912.°**(Ex03)**
Zenner de Polania I, Alvarez Alcaraz G: Analysis of the influence on the main beneficial fauna by two transgenic cultivars, cotton and corn, at El Espinal (Tolima). *Revista UDCA Actualidad & Divulgacion Cientifica* 2008, 11(1):133-142.°**(Ex08)**
Zhao Y, Neher D, Dively GP: Non-target effects of Bacillus thuringiensis transgenic corn on soil microarthropod community. *92nd Annual Meeting Ecological Society of America/Society for Ecological Restoration: 2007, August 5-10; San Jose, California, USA*. Ecological Society of America 2007.°**(NO)**
Zwahlen C, Hilbeck A, Gugerli P, Nentwig W: Field studies on the decomposition of transgenic BT-maize litter in the ground. *Verhandlungen der Gesellschaft fur Okologie* 2001, 31:252.°**(Ex02)**
Zwahlen C, Hilbeck A, Howald R, Nentwig W: Effects of transgenic Bt corn litter on the earthworm Lumbricus terrestris. *Molecular Ecology* 2003, 12(4):1077-1086.°**(Ex05)**
Zwahlen C, Hilbeck A, Nentwig W: Field decomposition of transgenic Bt maize residue and the impact on non-target soil invertebrates. *Plant and Soil* 2007, 300(1-2):245-257.°**(Ex05)**
