# Supplementary material for: Does the growing of Bt maize change abundance or ecological function of non-target animals compared to the growing of non-GM maize? A systematic review
Source: Environ Evid. 2022 Jun 6;11:21. doi: 10.1186/s13750-022-00272-0 (PMC11378853; doi:10.1186/s13750-022-00272-0)
Supplement: Supplementary file 3 — Additional file 3: Narrative summary and tables—studies relevant for the systematic review but not fitting the database for quantitative analyses (5 Tables). [file 13750_2022_272_MOESM3_ESM.pdf]

Does the growing of Bt maize change abundance or ecological function of non-target animals compared to the growing of non-GM maize? A systematic review

Michael Meissle[1*], Steven E. Naranjo[2], and Jörg Romeis[1]
[1]Agroscope, Research Division Agroecology and Environment, Reckenholzstrasse 191, 8046 Zurich, Switzerland, michael.meissle@agroscope.admin.ch; joerg.romeis@agroscope.admin.ch
[2]USDA-ARS, Arid-Land Agricultural Research Center, 21881 North Cardon Lane, Maricopa 85138, Arizona, USA, steve.naranjo@usda.gov

[*] Corresponding author

Published in: Environmental Evidence (2022), https://doi.org/10.1186/s13750-022-00272-0

Additional file 3: Narrative summary and tables - studies relevant for the systematic review but not fitting the database for quantitative analyses (narrative summary and 5 Tables)

Detailed tables with all extracted information for studies relevant to the review question, but not included in the quantitative database can be found in the following (Tables S3.1-S3.5). This also includes a narrative summary of the data based on the findings reported by the authors.

Vertebrates
Only three articles were identified that compared vertebrates in Bt maize with non-Bt maize (Table S3.1). Kennard [950] hypothesized that Bt maize fields might harbour fewer moths and might thus attract fewer bats to feed on them. While bat and moth activity were correlated, the link between moth activity and Bt maize, and consequently bats and Bt maize, was only evident in one of two studied sites in Texas (USA). Kryger [951] found no difference in damage of Bt and non-Bt maize plants planted next to forest edges by white-tailed deer in Michigan (USA). Campos & Hernandez [958] recorded 26 different mammal species by camera traps installed in forest fragments next to Bt or non-Bt fields in Brazil, while no differences were observed between both maize types.

Non-target invertebrates in field margins or other adjacent habitats.
Three studies investigated Lepidoptera species on weeds within or around Bt maize fields (Table S3.2). Jesse and Obrycki [942] recorded similar numbers of *Danaus plexippus* larvae (Danaidae, Monarch butterfly) on milkweed in and around commercial Bt and non-Bt maize fields in Central

Iowa (USA). In Germany, Gathmann et al. [945] installed 1×20m strips of goosefoot and mustard within Bt and non-Bt maize plots, either untreated or treated with pyrethroid. While no detrimental effects of Bt maize on larvae of *Plutella xylostella* (Plutellidae) and *Pieris rapae* (Pieridae) were observed, insecticide treatments in non-Bt maize reduced the number of larvae. Finally, Lang [941] monitored adult butterflies in field margins around Bt and non-Bt maize fields in Germany, but Bt status was not correlated with butterfly abundance or species richness. Five articles investigated invertebrates other than Lepidoptera inhabiting field-margins or habitats adjacent to Bt and non-Bt maize fields. Two studies from Brazil reported differences in dung beetle abundance and species composition between forest fragments surrounded by Bt and non-Bt maize. Because the studies were conducted next to commercial fields, however, field management including pesticide applications and maize hybrids planted varied according to farmers' needs [946,958]. In Germany, spider abundance and species composition in nettle margins sown next to Bt and non-Bt maize fields were similar [244]. A broad analysis of sweep net samples in the Philippines did not result in significant effects of Bt maize on arthropod abundance and composition in adjacent riparian sites [601]. Finally, an assessment of benthic cores from streams adjacent to Bt or non-Bt maize fields revealed no effects in 2005 and lower numbers of two aquatic taxa in 2006, while 55 other taxa remained unaffected [947]. In summary, the studies that compared invertebrates in habitats adjacent to Bt maize fields with those adjacent to non-Bt maize reported comparable communities, but only few studies were available.

Different pesticide regimes in Bt and non-Bt fields

In seven articles pesticide applications in Bt maize were different to non-Bt maize (Table S3.3) [505, 642, 675, 948, 949, 959, 962]. For example, Bt maize received insecticide-seed treatments, while non-Bt maize remained untreated [642, 948], or different numbers of pyrethroid sprays and different application dates resulted from applications according to threshold levels, or Bt maize stacked with herbicide tolerance was sprayed with glyphosate, while non-GM maize received different herbicides or mechanical weeding [675, 959]. No clear conclusions on effects of Bt maize on arthropods can be drawn from studies with different pesticide treatment between Bt and non-Bt maize.

Biodiversity, species richness, and community composition

Altogether 53 articles included analyses of arthropod biodiversity, species richness, and community structure involving Bt and non-Bt maize (Table S3.4). Nineteen articles also included insecticide treated plots. Overall, no consistent effects on biodiversity, species richness and community structure were reported by the study authors when both Bt and non-Bt plots remained untreated. When non-Bt plots were treated with insecticides (in most cases pyrethroids), either no effects or adverse effects on arthropod communities were observed when compared with untreated Bt maize.

Data on non-target invertebrate populations in Bt maize not useable for the database

Thirtyfive articles included experiments on arthropod populations (abundance, activity density, or predation/parasitism) in Bt and non-Bt maize, but the data are not presented in a form useable for the database (Table S3.5). Sixteen articles also included insecticide treated non-Bt maize as a comparator. We contacted the authors for details on the data, but they either did not respond or were unable to provide the needed information. In most cases, the data could not be used for the database because means were presented without a measure of variation (SD or SE). In addition, some articles presented error bars that were not specified (SD or SE) or unreadable in figures, and sometimes data were not presented for specific taxa, or only part of the data were presented selectively. In general the authors reported that Bt maize had no consistent effects on non-target arthropods. Two articles reported lower activity density or parasitism by *Macrocentrus* species, which are specialized hymenopteran larval parasitoids of the target pest of Lepidoptera-active Bt maize, i.e., *O. nubilalis* [907, 918].

Tables S3.1-S3.5

Note: Text in red in the "observed effects" columns indicates negative effects, text in green positive effects on non-target animals in Bt maize compared with the corresponding non-Bt maize as reported by the study authors. Critical appraisal criteria underlined in red or yellow represent low or medium internal or external validity of the data with a given reason. For critical appraisal criteria see main paper and the reference therein. Criteria rated high validity (no issues identified) are not mentioned explicitly. Tables are sorted alphabetically by country, location (data from USA by state), and year.

Table S3.1: Studies investigating activity of vertebrates in Bt and non-Bt maize fields. Labelled as N1 in Additional file 2.

Ref.*	Country	Location	Years	Experi-mentID	Event (Toxin)	Plot size	Comparison	Recorded taxa	Methods (Samples)	Response variables	N	Observed effects	Critical appraisal
958	Brazil	Campos Novos	2013, 2014	900	unknown (Cry1F, Cry1Ab, Cry1A.105)	farmer fields	20 commercial Bt fields compared to 20 non-Bt fields, different insecticides from field to field	medium and large mammal species	baited camera traps to record presence in forest fragments next to Bt or non-Bt fields	activity recorded 10m from field edge	20 total (10 per year)	26 large and medium mammals recorded, most present in Bt and non-Bt fragments. No statistical comparison	RC: different Bt and non-Bt maize lines, cultivars not related; FC: clustering present, especially 2014; HM: some fields never cropped with GE plants, likely different history of management; PD: different pesticides (insecticides, herbicides, fungicides) from field to field; VA: No statistical comparisons done; remark:authors state that environmental variables and management of crop fields was homogenous between Bt and non-Bt crops
951	USA	Southwest Michigan	2008	901	TC1507 × DAS59122 (Cry1F & Cry34/ 35Ab1)	5×5 maize plants	untreated Bt vs. untreated non-Bt	*Odocoileus virginianus* (white-tailed deer)	damage assessment	Feeding activity on Bt and non-Bt maize plants	9	no preference for Bt or non-Bt maize observed	EX: expression not measured (commercialized event); remark: small plots (5×5 plants)
950	USA	Uvalde, Medina (Texas)	2006	902	MON810 (Cry1Ab)	farmer fields	untreated Bt vs. untreated non-Bt	*Tadarida brasiliensis* (Brazilian free-tailed bats); moth adults, eggs and larvae	accoustig monitoring using 2 different types of detector (bats); pheromone traps (adult moths), visual counts (eggs & larvae), video imaging (adult moths)	activity (bats); activity and abundance (moths)	2	correlations of bat activity with moth activity, but moth activity could be linked to Bt or non-Bt maize only at one site. Overall no effect of Bt maize on bat activity	RC: commercial fields, Bt and non-Bt most likely unrelated; RE: only 2 sites ca. 50 km distance;

*References: **950** KS Kennard (2008) The effects of prey abundance and Bt (*Bacillus thuringiensis*) crops on bat activity in South-Central Texas agroecosystems. MSc thesis, University of Tennessee, Knoxville, USA (link); **951** AM Kryger (2009) Behavioral response of white-tailed deer to *Bacillus thuringiensis* maize. MSc thesis, Western Michigan University, Kalamazoo, Michigan, USA (link); **958** Campos & Hernandez (2015) PLOS ONE 10: e0145000 (doi)

Table S3.2: Studies investigating non-target invertebrates in field margins or adjacent habitats next to Bt maize fields compared with those next to non-Bt maize fields. Labelled as N2 in Additional file 2.

Ref.*	Country	Location	Years	Experi-mentID	Event (Toxin)	Plot size (ha)	Comparison	Method (Samples)	Recorded taxa	Response variable	N	Observed effects	Critical appraisal
946	Brazil	Campos Novos	2011	904	MON810 (Cry1Ab); TC1507 (Cry1F)	farmer fields	10 commercial Bt fields compared to 10non-Bt fields	baited pitfall traps (1) in 0.1-35 ha forest fragments (Araucaria) surrounded by maize fields	Scarabaeidae (dung beetles)	activity density; species richness; Shannon-Wiener index; principal components analysis	10	differences in composition of functional groups (more dwellers, less tunnelers and rollers in Bt fragments)	RC: different Bt and non-Bt maize lines, cultivars not related; FC: location of fields unknown; HM/IB/PD: Bt maize farmers typically used a package of agricultural inputs sold by manufacturing company, conventional farmers used several agricultural inputs, some non-chemical (animal manure, pork and chicken)
958	Brazil	Campos Novos	2013, 2014	900 (both years presented together)	unknown (Cry1F, Cry1Ab, Cry1A.105)	farmer fields	20 commercial Bt fields compared to 20 non-Bt fields, different insecticides from field to field	baited pitfall traps (1, either 2013 or 2014) in forest fragments (Araucaria) surrounded by maize fields 10m from edge	Scarabaeidae (dung beetles)	activity density, biomass	20 total (10 per year)	lower dung beetle abundance and species richness in Bt maize	RC: different Bt and non-Bt maize lines; cultivars not related; FC: clustering present, especially 2014; HM: some fields never cropped with GE plants, likely different history of management; PD: different pesticides (insecticides, herbicides, fungicides) from field to field; remark: environmental variables and management of crop fields was homogeneous between Bt and non-Bt crops
941	Germany	Bavaria	2000-2002	51, 74, 98 (3 years presented together)	Bt176 (Cry1Ab)	1.5-12	untreated Bt vs. untreated non-Bt	adult Lepidoptera on various host plants	visual counts (1-2) in margins (different natural habitats)	abundance; species richness	10	factor Bt status was included in the statistical model, but was not significant (3 years analyzed together)	SS: unequal sampling per site and year; remark: 10 margins Bt and non-Bt over 5 different farms and 3 years; ref. 244 and ref. 650 state that experiment was conducted 2001-2003
244	Germany	Bavaria	2001-2003	51, 74, 98	Bt176 (Cry1Ab)	2	untreated Bt vs. untreated non-Bt (separated fields)	aspirator (4-5) in *Urtica dioica* strips planted on northern edge of maize fields	Araneae	abundance; number of species; proportion of guilds	3	no effects (Fig. 1b, 2, 3, Tab. 1, 2)	RE: only 3 replicated fields
945	Germany	Bonn	2001-2003	53, 76, 100	MON810 (Cry1Ab)	0.25	untreated Bt vs. untreated non-Bt; untreated Bt vs. Baytroid (pyrethroid) treated non-Bt (1 spray)	larvae of *Plutella xylostella* and *Pieris rapae on Chenopodium album / Sinapis alba* in weed strips	beat tray in 1×20m weed strips established in maize plots (before and during pollen shed)	abundance	8	without insecticides: no effect on *P. rapae* in any year; no effect on *P. xylostella* in 2001, 2003, and before anthesis in 2002; more in Bt maize during anthesis in 2002 (Tab. 3); with insecticide: no effect	SD: only two sampling dates

												on *P. rapae* in 2001, 2003, and during anthesis in 2002, more in Bt maize before anthesis in 2002; no effect on *P. xylostella* in 2001, before anthesis in 2002, during anthesis in 2003, more in Bt maize during anthesis in 2002 and before anthesis in 2003 (Tab. 3)	
601	Philippines	Camarines Sur	2006-2009	133, 141, 142, 149, 160	MON810 (Cry1Ab)	1	untreated Bt vs. untreated non-Bt	sweep net (6) in riparian sites in 5-25m distance to the maize field	all taxa	abundance; principal components curve analysis (PCA); exponential of Shannon index	3	no effects (Fig. 2, Tab. 2)	EX: expression not confirmed (commercialized event); IB: one application of Larvin (thiodicarb) against *Atherigona oryzae* (rice shoot fly) applied to all fields at planting;
942	USA	Central Iowa	2000	903	MON810 (Cry1Ab)	farmer fields	untreated Bt vs. untreated non-Bt	eggs and larvae of *Danaus plexippus* (Monarch) on milkweeds *(Asclepias syriaca*)	weekly visual counts (4) 7m into the field and margins	abundance	3	no effects (Tab. 1)	RE: only 3 separated fields for Bt and non-Bt, within 3.2km; HM/PD: no information on field management and pesticide applications;
947	USA	North-central Indiana	2005, 2006	905, 906	not specified (Cry1Ab, see PNAS 2008, 105:E11)	farmer fields	untreated Bt vs. untreated non-Bt	benthic cores (2) taken from streams adjacent to Bt or non-Bt maize fields (> 200m upstream same crop type)	all taxa	abundance, EPT richness; Shannon diversity	2-4 in 2005; 3 in 2006	2005: no differences, 2006: differences for *Simulium* and *Tropisternus*, no effect on 55 other taxa	RC: cultivars unrelated; FC: location of fields unknown; HM/IB/PD: no information (commercial fields); remark: not clear if observed effects were positive or negative

[*]**References: 244** Ludy & Lang (2006) Biol Control 38: 314-324 (doi); **601** Alcantara (2012) Environ Entomol 41: 1268-1276 (doi); **941** Lang (2004) Environ Biosafety Res 3: 55-66 (doi); **942** Jesse & Obrycki (2003) Agric Ecosyst Environ 97: 225-233 (doi); **945** Gathmann et al. (2006) Mol Ecol 15: 2677-2685 (doi); **946** Campos & Hernandez (2014) Ecol Indic 49: 216-227 (doi); **947** Chambers et al. (2010) Ecol Appl 20: 1949-1960 (doi); **958** Campos & Hernandez (2015) PLOS ONE 10: e0145000 (doi)

Table S3.3: Studies where different pesticides were applied to Bt and non-Bt maize (e.g., IPM studies with pesticide applications according to product labels or thresholds). Labelled as N3 in Additional file 2.

Ref.*	Country	Location	Years	Experi-mentID	Event (Toxin)	Plot size (ha)	Pesticide treatment	Method (Samples)	Recorded taxa	Response variable	N	Observed effects	Critical appraisal
962	Colombia	Cereté and San Pelayo	unknown	985, 986	unknown (P-3032WHR)	commercial	2 Bt and 2 conventional fields, commercial fields with unknown agricultural practice. No information on insecticide treatments and Bt proteins	soil extraction, pitfall traps, sticky traps, sweep nets	all taxa	abundance, similarity index Jaccard, Sorensen, Dice	2	no differences in total abundance and diversity profiles	RC/FC/HM/IB/PD/SD: no information; comparison: no information on insecticide treatments, most likely different treatments (commercial fields) Bt proteins: no information on Bt proteins
959	India	Madhya Pradesh	2009, 2010	907, 908	MON89034 × NK603 (Cry1A.105; Cry2Ab2)	0.0018	2 Bt maize lines: round-up at 3 concentrations, no insecticide; corresponding controls: either no treatment; or only endosulfan; or atrazine & endosulfan	visual counts (3)	aphids, leafhoppers, coccinellids, spiders, syrphids, pollinators	abundance	3	no statistical comparison done by authors	RC: non-Bt most likely unrelated to Bt lines; EX: not measured (commercialized event); PD: no untreated Bt treatment available - differences in herbicide treatment may influence arthropods; SD: 3 samples per season; remark: no statistical comparisons done by authors
675	Mexico	14 locations in 5 eco-regions across Mexico	2009-2013	909-922	MON89034 × MON88017 (Cry1A.105; Cry2Ab2; Cry3Bb1); MON89034 × NK603 (Cry1A.105; Cry2Ab2)	0.04-0.4 (pilot trials)	insecticide treatment according to threshold levels, non-Bt plots received more insecticides, in particular against target pests; also weed treatment was different between plots: glyphosate-tolerant maize received glyphosate treatments, other maize selective herbicides or mechanical weeding	pitfall traps (2-7), sticky traps (2-7), visual counts (1-3)	20 taxa	abundance	3-4	fewer *Chaetocnema* spp., *Euxesta* spp., Carabidae, parasitic wasps in MON89034 x MON88017, fewer parasitic wasps in MON89034xNK603, no effects in 69 other statistical comparisons (data analyzed together with experimental plots)	EX: expression not measured (commercialized event); SD: some experiments and methods only 1-3 samples per season; remark: results are based on all data including the 18 experimental trials (those data are in the database for meta-analyses)
505	USA	Rock Springs PA	2001	923	BT11 (Cry1Ab)	0.062	isoline: diazinon + lindane (seed treatment), lambda-cyhalothrin (spraying according to thresholds, week 31 and 34); Bt: diazinon + landane, lambda-cyhalothrin (week 32)	visual counts (11-14)	Coccinellidae, Aphididae	abundance	3	more *Harmonia axyridis* and less *Coleomegilla maculata* in Bt maize; no differences in 4 other ladybird species; less aphids in Bt maize in 2 out of 8 weeks	PD: spraying according to threshold levels

949	USA	Rock Springs PA	2001, 2002	923, 924	BT11 (Cry1Ab)	0.062	2001: isoline: diazinon + lindane (seed treatment), lambda-cyhalothrin (spraying according to thresholds, week 31 and 34); Bt: diazinon + landane, lambda-cyhalothrin (week 32)<br>2002: isoline: lambda-cyhalothrin (2 sprays); Bt: no insecticide	pitfall traps (8-9)	Coleoptera, Formicidae	species richness (rarefaction curves); redundancy analyses;	3	rarefaction curves: no differences for epigeal beetles and ants and for Carabidae between Bt and non-Bt sweet maize in both years. RDA was done for Bt sweet maize, virus resistant acorn squash and Colorado potato beetle resistant potato, no differences between Bt and non-Bt sweet maize reported	PD: spraying according to threshold levels; remark: RDA data combined for multiple crops and years, no clear conclusion about Bt sweet maize alone can be drawn.
642	USA	Rock Springs PA	2003, 2004	925, 926	MON863 (Cry3Bb1) field corn study	0.11	Bt maize: neonicotinoid seed treatment (TraNeo) and seed treatment plus at-planting soil application of pyrethroid (TraNeoPyr); non-Bt maize: untreated (Iso) and pyrethroid treated (IsoPyr)	pitfall traps (10-11)	Carabidae (4 species)	activity density	3	*Poecilus chalcites* lower in IsoPyr, TraNeo, and TraNeoPyr than in Iso in one year;<br>*Pterostichus melanarius* higher in Iso and TraNeoPyr than in TraNeo and IsoPyr in one year:<br>*Harpalus pensylvanicus* higher in Iso than in other treatments in both years at some sampling dates.<br>No significances reported for *Scarites quadriceps*	IB: Bt maize seed with seed treatment (realistic scenario); carabids are known to be affected by neonicotinoids; remark: data from the sweet corn trial in database
948	USA	Rock Springs PA	2003, 2004	925, 926	MON863 (Cry3Bb1) field corn study	0.11	Bt maize: neonicotinoid seed treatment (TraNeo) and seed treatment plus at-planting soil application of pyrethroid (TraNeoPyr); non-Bt maize: untreated (Iso) and pyrethroid treated (IsoPyr)	pitfall traps (10-11)	Carabidae, Chrysomelidae, Nitidulidae	activity density; species richness; rarefaction curves; principal response curve analysis; redundancy analysis	3	carabid activity density: no effect in 2003, higher in Iso than in all other treatments in 2004; no effect on Carabidae species richness; PRC showed negative response of Coleoptera communities in all treatment plots compared to untreated non-Bt maize; no effect on functional groups.	IB: Bt maize seed with seed treatment (realistic scenario); carabids are known to be affected by neonicotinoids; remark: diversity data from the sweet corn trial in **Table 5** of this appendix

[*]**References: 505** Hoheisel & Fleischer (2007) J Insect Sci 7:61 (doi); **642** Leslie et al. (2009) Environ Entomol 38: 935-943 (doi); **675** Madrid et al. (2018) J Appl Entomol 142: 525-538 (doi); **948** Leslie et al. (2010) Environ Entomol 39: 2045-2055 (doi); **949** Leslie et al. (2007) Environ Entomol 36: 234-244 (doi); **959** Sushilkumar et al. (2017) Indian J Weed Sci 49: 241-247 (doi) ; **962** Sanchez et al. (2018) Temas Agrarios 23: 121-130 (link);

Table S3.4: Studies measuring parameters other than abundance, activity density, or predation/parasitism rates, e.g., species richness, biodiversity, or community structure. Labelled as N4 in Additional file 2.

Ref.*	Country	Location	Years	Experi-ments	Event (Toxin)	Plot size (ha)	Comparison	Method (Samples per year)	Recorded taxa	Response variable	N	Observed effects	Critical appraisal
644	China	Gongzhuling	2012	188	MON89034 × NK603 (Cry1A.105 & Cry2Ab)	0.04	untreated Bt vs. untreated non-Bt	soil extraction (4)	Collembola	canonical correspondence analysis; species richness	3	no effects (Tab. 3, Fig. 3)	
665	China	Gongzhuling	2014	215	Bt799 (Cry1Ac)	0.015	untreated Bt vs. untreated non-Bt	litter bags buried 2013, extracted 2014 (3)	all taxa	species richness, Pielou index, Simpson index, Shannon-Wiener index	3	no effects (Tab. 2, 3)	
664	China	Gongzhuling	2014, 2015	215, 227	Bt799 (Cry1Ac)	0.015	untreated Bt vs. untreated non-Bt	soil extraction (6)	Collembola	species richness, Shannon-Wiener index, redundancy analysis (RDA), morphological parameters (ocelli number, length, pigmentation, furca development)	3	species richness, Shannon, morphological parameters lower in Bt before sowing 2015, no effects on other dates and total in both years, RDA no effect of maize type (Tab. 4, 5, 6, Fig. 1)	
674	China	Gongzhuling	2014, 2015	215, 227	Bt799 (Cry1Ac)	0.015	untreated Bt vs. untreated non-Bt	soil extraction (5), soil hand sorting (5)	meso-, micro-, macrofauna	species richness, Simpson index, Shannon-Wiener index, Pielou index	3	no effects (Tab. 3)	
966	China	Gongzhuling	2014, 2015	940, 941	IE09S034 (Cry1Ie)	0.015	untreated Bt vs. untreated non-Bt	soil extraction (5), soil hand sorting (5)	all taxa	Shannon-Wiener, Simpson, Pielou indices, species number, redundancy analysis (RDA)	3	no effects (Tab. 2, 4, Fig. 2, 3)	EX: expression not measured, non-commercialized event
670	China	Hebei Province	2012, 2013	190, 206	IE09S034 (Cry1Ie)	0.02	untreated Bt vs. untreated non-Bt	visual counts (10)	non-lepidopteran pests (all taxa)	Shannon-Weaver diversity index, Simpson diversity index, number of species, Pielou evenness index, redundancy analysis (RDA), Bray Curtis	3	no effects (Tab. 1, 2, Fig. 3, 4)	EX: expression not measured, non-commercial event
671	China	Hebei Province	2012, 2013	190, 206	IE09S034 (Cry1Ie)	0.02	untreated Bt vs. untreated non-Bt	visual counts (10), suction samples (4), pitfall traps (4)	natural enemies (all taxa)	Shannon-Weaver diversity index, Simpson diversity index, number of species, Pielou evenness index, redundancy analysis (RDA), Bray Curtis	3	no effects (Tab. 1, 3, Fig. 2, 4), except higher Pielou evenness for Bt maize in 2013 with visual counts (Tab. 1)	EX: expression not measured, non-commercial event
953	China	Hebei province	2012, 2013	190, 206	IE09S034 (Cry1Ie)	0.02	untreated Bt vs. untreated non-Bt	visual counts (12/11); pitfall traps (7); aspirator (4/5); pan traps (4/5)	all taxa	species richness; Simpson index; Shannon-Wiener index; Pielou index	3	no effects (Tab. 5)	EX: expression not measured, non-commercial event; remark: subsets of those data published in Ref. 670 and 671

626	China	Shangzhuang	2012, 2013	195, 209	Bt799 (Cry1Ac)	0.015	untreated Bt vs. untreated non-Bt	visual counts (10)	all taxa	Shannon diversity index (H), Pielou evenness index (J); Simpson diversity index (D); Principal response curve (PRC); Bray-Curtis index	3	no overall effects (Tab. 1); H, J, and D lower in Bt for the last sample date in 2013 (Fig. 1)	
662	China	Xinlitun, Haidian District, Beijing	2014-2016	222, 230, 232	Bt38 (Cry1Ac)	0.015	untreated Bt vs. untreated non-Bt	visual counts (5-10)	all taxa	Shannon diversity index, Pielou evenness index, Simpson diversity index, species richness, Bray-Curtis dissimilarity	3	no effects in 2015 and 2016, in 2014 Shannon 1 date higher in Bt, Pielou 1 date higher, 2 dates lower in Bt, Simpson 1 date higher, 1 date lower in Bt, (Fig. 1-3), no difference for species richness and Bray-Curtis (Fig. 4,5)	EX: expression not quantified (but effects on target described)
673	China	Yitong Manchu	2015, 2017	228, 233	DBN9936 (Cry1Ab)	0.015	untreated Bt vs. untreated non-Bt	visual counts (13-14), pitfall traps (6-7)	all taxa	species accumulation curves, species richness, Margalef, community similarity, Shannon-Wiener, Simpson, Sorenson, Pielou, dominant concentration indices	3	no effects (Fig. 1-7, Tab. 2)	EX: expression not measured, but lower numbers of target Lepidoptera confirmed in the field
604	Czech Republic	Ceske Budejovice	2003-2005	102, 115, 121	MON810 (Cry1Ab)	0.5	untreated Bt vs. untreated non-Bt	plant removal (6)	all plant-dwelling taxa	canonical correspondence analysis	5	no effects (Fig. 3, Tab. 3, 4) (all years analyzed together)	
605	Czech Republic	Ceske Budejovice	2003-2005	102, 115, 121	MON810 (Cry1Ab)	0.5	untreated Bt vs. untreated non-Bt	pitfall traps (4-6)	Carabidae, Staphylinidae, Araneae	species richness	5	no effects in any year (Tab. 1)	
606	Czech Republic	Ceske Budejovice	2009-2011	156, 165, 176	MON88017 (Cry3Bb1)	0.5	untreated Bt vs. untreated non-Bt or non-Bt treated with 1 soil application of chlorpyrifos	pitfall traps (4-6)	Araneae	canonical correspondence analysis; Berger-Parker index; Simpson dominance index; evenness index; Margalef index; Shannon index	5	no effects (text, Fig. 5)	
637	Czech Republic	Ceske Budejovice	2009-2011	156, 165, 176	MON88017 (Cry3Bb1)	0.5	untreated Bt vs. untreated non-Bt or non-Bt treated with 1 soil application of chlorpyrifos	plant removal (3-4), sticky traps (1)	all plant-dwelling taxa	redundancy analysis (RDA)	5	no effects (Fig. 2)	SD: only 1 sticky trap sample per year; 3 plant removal samples in 2009
654	Czech Republic	Ceske Budejovice	2009-2011	156, 165, 176	MON88017 (Cry3Bb1)	0.5	untreated Bt vs. untreated non-Bt or non-Bt treated with 1 soil application of chlorpyrifos	pitfall traps (4-6)	Carabidae	Spearman's rank correlation coefficient; Chao 1 index; principal components analysis	5	untreated: no effects (Fig. 1, 4, Tab. S6); insecticide treated non-Bt showed lower species number (Tab. S6), no effects on rank abundance (Fig. 1) and for PCA (Fig. 4)	
655	Czech Republic	Southern Bohemia	2009-2011	156, 165, 176	MON88017 (Cry3Bb1)	0.5	untreated Bt vs. untreated non-Bt or non-Bt treated with 1 soil application of chlorpyrifos	pitfall traps (4-6)	Staphylinidae	Simpson index; indices for bionomics, food specialization, temperature requirements, size group	5	no effects (Tab. 2, 3, Fig. 2)	

669	Denmark	Aarhus	2014	211	MON810 (Cry1Ab)	0.009	untreated Bt vs. untreated non-Bt	pitfall traps (3)	Carabidae	number of species, body size inequality	10	no effects (Tab. 2, Fig. 2-4)	remark: body sizes were taken from literature, not measured for individuals of this study
660	Denmark, Sweden, Spain	Flakkebjerg, Lund, Sesena	2013 (no indices for 2014 printed)	200, 207, 208	MON810 (Cry1Ab)	0.01	untreated Bt vs. untreated non-Bt	soil extraction (1)	Nematoda	Shannon-Weaver diversity index; maturity index (MI); Channel index (CI), enrichment index (EI), plant parasitic index (PPI); basal index, structure index	10	no effects in Spain & Sweden, Denmark: MI and CI higher in control, EI higher in Bt (Tab. 5)	EX: expression not measured (commercialized event); SD: only 1 sample; remark: no treatment comparison for 2014 presented
603	France	Sassenay near Chalon sur Saone, Burgundy	1998	24	Bt176 (Cry1Ab)	1.42	untreated Bt vs. untreated non-Bt or non-Bt sprayed once with lambda-cyhalothrin or Bt spray	beat cloth (7), pan traps (6), pitfall traps (8)	all taxa	principal response curves; number of taxa, Shannon diversity index	3	PRC: no effect of Bt maize or Delfin, but negative effects of pyrethroid on ground-dwelling taxa (Fig. 3, 4), no effect of Bt maize, weak effect of Delfin and stronger effect of pyrethroid on plant-dwelling taxa (Fig. 7, 8), no effects on flying arthropods (Fig. 12, 13);<br>diversity: no effects among treatments on soil dwellers, plant dwellers, or flying arthropods	
244	Germany	Bavaria	2001-2003	51, 74, 98	Bt176 (Cry1Ab)	2	untreated Bt vs. untreated non-Bt	aspirator (4-5)	Araneae	number of species; proportion of guilds	3	overall no effects, more spiders in Bt in 2003 (Fig. 1, 3, Tab.1, 2)	RE: only 3 replicated fields
639	Germany	Bonn	2001-2003	53, 76, 100	MON810 (Cry1Ab)	0.25	untreated Bt vs. untreated non-Bt or non-Bt sprayed once with pyrethroid	pitfall traps (13-14)	Araneae, Carabidae	principal component analysis (PCA)	8	community of spiders and carabids in Bt maize differed from non-Bt maize in 2001, not in 2002 and 2003 (Fig. 5) (3 species higher in Bt maize, 5 species lower, Tab. 3)	remark: probable effect 2001 because of massive corn borer infestation in untreated and somewhat also in treated non-Bt plots (brown and damaged plants) while Bt maize still green at end of season
656	Germany	Braunschweig	2008-2009	144, 154	MON89034 × MON88017 (Cry1A.105 & Cry2Ab2 & Cry3Bb1)	0.13	untreated Bt vs. untreated non-Bt or non-Bt with 1 soil application of tefluthrin	soil extraction (3)	Nematoda	maturity index, enrichment index, structure index, channel index	8	no effects (Tab. 3, Fig. 1)	SD: only 3 sampling dates per year
652	Germany	Halle Saale / Göttingen	2001-2003 / 2003	59, 84, 107/ 106	MON810 (Cry1Ab)	0.36 / 0.07	untreated Bt vs. untreated non-Bt	visual collection (2)	aphid parasitoids	Wainstein similarity index, connectivity, number of species in	6 / 8	similar values between Bt and control pairs (Tab. 4-8, Fig.	remark: no conclusions possible

										food web, host-parasitoid interactions, connectance		10,11), no statistical comparison	because data were not compared statistically
612	Germany	Oderbruch	2003-2004	96, 113	MON810 (Cry1Ab)	5 to 9	untreated Bt vs. untreated non-Bt	pitfall traps (4)	Carabidae, Araneae	Jaccard index; Sörensen-quotient; Renkonen-index; Shannon Weaver diversity index; evenness index	2	no statistical comparison (Tab. 20, 23)	RE: only 2 replicated split-fields; remark: no conclusions possible because data were not compared statistically
610	Germany	Schwarzenau near Würzburg	2005-2007	128, 136, 143	MON88017 (Cry3Bb1)	0.13	untreated Bt vs. untreated non-Bt	pitfall traps (11-25)	Carabidae	Shannon index; evenness index	8	no effects (Tab. 3-2)	
645	Germany	Schwarzenau near Würzburg	2007	143	MON88017 (Cry3Bb1)	0.128	untreated Bt vs. untreated non-Bt	soil extraction (3)	Nematoda	number of genera, maturity index, enrichment index, structure index, channel index, Bray-Curtis similarities	8	no effects (Tab. 3, Fig. 5, 6a, 7a, b) except genus composition, which was different in Bt in one of 3 sampling dates (2 genera more, 4 genera less, Fig. 7c)	SD: only 3 sampling dates per year
219	Hungary	Budapest	2001-2003	54, 77, 101	MON810 (Cry1Ab)	0.09	untreated Bt vs. untreated non-Bt	pitfall traps (14-23)	Carabidae	number of species; Shannon diversity index, Rényi diversity	6	no effects (Tab. 2, Fig. 2)	EX; expression not measured (commercialized event); remark: author N = 12
521	Hungary	Budapest	2001-2003	54, 77, 101	MON810 (Cry1Ab)	0.09	untreated Bt vs. untreated non-Bt	pitfall traps (14-23)	Staphylinidae	species richness; Fisher alpha diversity index; Metric ordination; principal coordinate analysis; Horn index	6	no effects (Fig. 2, 3)	EX; expression not measured (commercialized event)
666	Hungary	Budapest	2002, 2003	77, 101	MON810 (Cry1Ab)	0.09	untreated Bt vs. untreated non-Bt	sticky traps (12/7)	Alticini (Chrysomeli-dae)	species richness	6	no effects except for more species in Bt on 1 of 12 dates in 2002 and 1 of 7 dates in 2003 (Fig. 1)	EX; expression not measured (commercialized event)
629	Hungary	Budapest	2006-2008	130, 139, 145	DAS59122 (Cry34/35Ab1); DAS1507 × DAS59122 (Cry1F & Cry34/35Ab1)	0.06	untreated Bt vs. untreated non-Bt	visual counts (4), sticky traps (4), pitfalls traps (4)	all taxa	food web parameters (nr. trophic species, nr. of links between trophic groups, connectance, arithmetic means of bottom-up and top-down indexes	4	no significant effects (Fig. 1, Table 3)	EX; expression not measured (commercialized event)
960	Hungary	Budapest	2007-2008	130, 139, 145	DAS59122 (Cry34/35Ab1); DAS1507 × DAS59122 (Cry1F & Cry34/35Ab1)	0.06	untreated Bt vs. untreated non-Bt	visual counts, sticky traps, pitfalls traps (weekly June to September)	all taxa	food web parameters (nr. trophic species, nr. of links between trophic groups, connectance, arithmetic means of bottom-up and top-down indexes	4	differences for herbivores in trophic links/ link groups and path lengths (Tab. 2)	EX; expression not measured (commercialized event); remark: differences explained by different weed densities

619	Italy	Northern Italy	1999	31	Bt176 (Cry1Ab)	unknown	untreated Bt vs. untreated non-Bt	soil extraction (1)	Nematoda	Shannon-Weaver index; Hill numbers; Pielou index; Sörensen index	8	no statistical comparison (indices almost the same) (Tab. 5)	EX; expression not measured (commercialized event); HM: no information on history of field management; SD: only 1 sample date; remark: replicates up to 350km distance
601	Philippines	Camarines Sur	2006-2009	133, 141, 142, 149, 160	MON810 (Cry1Ab)	1	one application of Larvin (thiodicarb) at planting to Bt and non-Bt treatment	visual counts (6)	all taxa	principal response curve (PRC); exponential of Shannon index	3	no effects (Fig. 1, Tab. 1)	EX; expression not measured (commercialized event); RE: only 3 separated fields; HM: no information on history of field management; IB: one application of thiodicarb at planting; PD: no information on pesticide applications during study
632	Philippines	Camarines Sur	2006-2009	133, 141, 142, 149, 160	MON810 (Cry1Ab)	1	one application of Larvin (thiodicarb) at planting to Bt and non-Bt treatment	visual counts (6)	PRC: herbivores; RDA: natural enemies	principal response curve (PRC); Redundancy analyses (RDA)	3	no effects (Fig. 2, 4)	EX; expression not measured (commercialized event); RE: only 3 separated fields; HM: no information on history of field management; IB: one application of thiodicarb at planting; PD: no information on pesticide applications during study
917	Philippines	Isabela, Camarines Sur	2002	930, 931	MON810 (Cry1Ab)	0.0045	untreated Bt vs. untreated non-Bt or non-Bt sprayed twice with carbofuran. Additional in Camarines Sur 1 Karate spray in both treatments.	visual counts; sweep net (5)	*Chrysopa*, *Pseudomicro-mus*, *Micraspis* (predators)	Shannon index	3	no effects at both sites (Fig. 12, 13)	EX; expression not measured (commercialized event); remark: plot size 6 rows × 10m

968<br>904	Philippines	Isabela, Pangasinan, South Cotabato	2009	927, 928, 929	MON89034; MON89034 × NK603 (Cry1A.105 & Cry2Ab2)	0.01	untreated Bt vs. untreated non-Bt or non-Bt sprayed once with carbofuran or once with cypermethrin	sweep net; visual counts; aspirator; pitfall traps; sugar or tuna flakes baits (3 each)	canopy dwelling and ground dwelling arthropods	Shannon index	3	no effects (Fig. 5, 6)	EX: expression not measured (commercialized event); remark: small plots (10 rows × 10m)
920	Poland	Budziszow/ Gluchow	2008-2010	146, 155, 164/ 147, 158, 167	MON810 (Cry1Ab)	0.16	untreated Bt vs. untreated non-Bt or non-Bt sprayed once with lambda-cyhalothrine	pitfall traps (14-16)	Carabidae	Shannon index; no. of species, evenness, DCA	4	Budziszow: no effects in 2008 and 2009, in 2010 higher Shannon index in Bt (Tab. 3), Gluchow: lower in Bt in 2 of 3 years (Tab. 4); not confirmed by DCA (Fig. 1, 2);	EX; expression not measured (commercialized event);
935	Romania	Troian	2011, 2012	989, 990	7 transgenic hybrids (glyphosate-tolerant, Coleoptera-, Lepidoptera resistant)	0.002	untreated Bt, untreated non-Bt	pitfall traps (8)	Carabidae	Sörensen index	4	similarity of carabids 97% (all non-Bt and all Bt hybrids pooled together)	RC: used hybrids and events not described, data pooled for 7 transgenic and 7 conventional hybrids; EX: expression not measured; remark: plot size 4 rows × 7m
653	Slovakia	Borovce	2012, 2013	187, 203	MON810 (Cry1Ab)	0.01	untreated Bt vs. untreated non-Bt	soil extraction (1)	Nematoda	Shannon-Weaver diversity index; maturity index (MI); plant parasitic index (PPI); PPI to MI ratio; enrichment index; structure index	10	2012 no effects except lower structure index in Bt 2012; 2013 no effects (Tab. 3)	SD: only 1 sample
631	South Africa	Tshiombo/ Vaalharts	2008, 2009	152, 161/ 153, 162	MON810 (Cry1Ab)	25-30, 0.05	untreated Bt vs. untreated non-Bt	plant removal (1)	detritivores, chewing & sucking herbivores, chewing & sucking predators, parasitoids	Shannon index; Margalef index; species per 20 plants	3	no effects (Tab. 1)	HM: no information on history of field management; SD: only 1 sample
514	Spain	Madrid	2000-2002	39, 63, 87	Bt176 (Cry1Ab)	0.2	untreated Bt vs. untreated non-Bt or non-Bt with seed treatment (imidacloprid)	pitfall traps (6)	Araneae, Carabidae, Staphylinidae	species richness; Shannon-diversity index; Sorenson quantitative coefficient	3	untreated: no effects; treated non-Bt: no effect, except lower Staphylinid richness (Tab. 2) (all years analyzed in one model)	
657	Spain	Madrid province (Central Spain)	2009-2011	157, 166, 179	MON810 (Cry1Ab)	0.5	untreated Bt vs. untreated non-Bt	soil extraction (9-16)	Collembola	species richness, Simpson index, Shannon index	3	higher values in Bt (all 3 values significant, Tab. 4) (all years analyzed in one model)	VA: authors state that they worked with 54-96 samples per treatment, true N is 3 plots.
171	USA	Ames IA/ Monmouth IL	2001, 2002	56, 82/ 66, 90	MON863 (Cry3Bb1)	0.033	untreated Bt vs. untreated non-Bt, non-Bt with seed treated	pitfall traps (6-8); soil extraction (6-8)	Collembola	number of species; ACE estimator; Shannon diversity index; Simpson diversity index	4	no effects (Tab. 3, 4)	EX: expression not measured (commercialized event)

							(imidacloprid) or non-Bt with tefluthrin treatment at planting						
527	USA	Scott County IA, Fowler IN, York NE, Arkansaw WI	2004-2006	120, 129, 137	TC1507 (Cry1F)	0.8	untreated Bt vs. untreated non-Bt	visual counts, sticky traps, pitfall traps (3), litterbags (2)	16 taxa	principal response curves	4	no effects (Fig. 1, 2)	EX: expression not measured (commercialized event); SD: only 2 or 3 sampling dates
952	USA	Johnston IA/ Frankfort IN/ York NE	2005/ 2006-2007/ 2006-2007	934/ 937, 938/ 935-936	DAS59122 (Cry34/35Ab1); DAS1507 × DAS59122 (Cry1F & Cry34/35Ab1)	0.2 /0.4/ 0.4	untreated Bt vs. untreated non-Bt or non-Bt with Poncho seed treatment	visual counts; sticky cards; pitfall traps 3); litterbags (1)	all taxa	principal response curves	4	untreated: no effects (pp. 79-81 Fig. 1-3); differences between seed treated and untreated communities (pp. 79-81 Fig. 1-3)	EX: expression not measured (commercialized event); SD: only 3 samples (visual counts, sticky cards, pitfall traps) or 1 sample (litterbags) per year
224	USA	Marlboro MD/ Salisbury MD	2000, 2001	41, 65/ 47, 69	Bt11 (Cry1Ab) sweet corn	0.05	untreated Bt vs. untreated non-Bt or non-Bt sprayed 5 times with pyrethroid	visual counts (6); sticky cards (4); pitfall traps (6)	all taxa	Shannon-Weaver diversity index; principal response curve (PRC)	4	untreated: no effects (Fig. 1) insecticide treated non-Bt: visual counts: less taxa (1 year, both locations); PRC 17 taxa decreased; sticky cards: less taxa (both years at 1 location, 1 year at the other location); PRC 24 taxa decreased; pitfall traps: PRC: 4 taxa decreased	EX: expression not measured (commercialized event)
638	USA	Marlboro MD	2002	89	Bt11 (Cry1Ab) sweet corn	0.046	untreated Bt vs. untreated non-Bt or non-Bt sprayed 5 times with pyrethroid	emergence traps (4), litter bags (5)	all taxa	Shannon-Wiener index	4	untreated: no effects (Fig. 2.3, 2.9); insecticide treated non-Bt: emergence traps: higher in Bt maize on one sampling after all insecticide treatments (Fig. 2.3); litter bags: no effects (Fig. 2.9)	
170	USA	Queenstown MD	2000, 2001	46, 68	Pacha & Bt11 (VIP3A & Cry1Ab)	0.4	untreated Bt vs. untreated non-Bt and non-Bt sprayed twice with lambda-cyhalothrine	visual inspections (10/14), yellow sticky cards (9/11), pitfall traps (8/11)	all taxa (family, order level, carabids to genus level)	Shannon-Weaver index	3	untreated: overall no effects, in 4 week periods before and after anthesis, Bt communities less diverse (Fig. 1); treated non-Bt showed declined diversity after second pyrethroid application (Fig. 1)	
635	USA	Beltsville MD	2003, 2004	99, 114	MON863 (Cry3Bb1)	0.27	untreated Bt vs. untreated non-Bt or non-Bt with 1 soil treatment of pyrethroid	soil (3) & litter extraction (5)	Nematoda	successional maturity indices (PPI and MI); trophic diversity; Channel index, enrichment index (EI), structural index,	3	untreated: no effects (Fig. 1, Tab. 2) treated non-Bt: soil samples: MI higher in Bt maize over the season (mainly due to	EX: expression not measured (commercialized event); SD: only 3 soil samples per year

												difference at planting), no effects on overwintering; root samples: MI higher in Bt maize, EI lower in Bt maize	
634	USA	Freeville NY	2001-2003	57, 83, 104	MON863 (Cry3Bb1)	0.25	untreated Bt vs. untreated non-Bt or non-Bt with 1 application of pyrethroid	pitfall traps (12-14)	Carabidae	Simpsons's D	4-7	no effects except higher in Bt in 2001 compared to untreated non-Bt (Tab. 1)	EX: expression not measured (commercialized event); SS: unbalanced design: Bt: N=4, non-Bt N=7
948	USA	Rock Springs PA	2003, 2004	105, 116	Bt11 (Cry1Ab) - sweet corn study	0.11	untreated Bt vs. untreated non-Bt or non-Bt sprayed 4 times with pyrethroid	pitfall traps (10-11)	Carabidae, Chrysomelidae, Nitidulidae	species richness; rarefaction curves; principal response curve (PRC); redundancy analysis (RDA)	3	untreated: no effect on Carabidae species richness; negative response in PRC of Coleoptera communities in Bt maize in one year June to August (comparison Bt vs. Iso), no effect in comparison Bt+Ins vs. Iso+Ins; no effect on functional groups (Fig. 3-5); insecticide treated non-Bt: no effect on Carabidae species richness, Coleoptera communities, and functional groups (Fig. 3-5)	remark: Iso untreated had much higher values in PRC than all other treatments in 2003, including the Iso+Ins treatment although insecticide treatments were done later. Likely a by-chance difference.
916	USA	Burlington VT	2003, 2004	932, 933	MON810 (Cry1Ab)	0.25	untreated Bt vs. untreated non-Bt	pitfall traps (8-9); soil extraction (7-8)	Carabidae, Collembola	Simpson's diversity index; Shannon-Wiener diversity index; Simpson's evenness index; principal response curves (PRC)	5	PRC soil dwelling Collembola: significant, 3 taxa reduced in Bt maize on some sampling dates (Fig. 1c). No effects for other taxa (Fig. 1) and diversity indices (Tab. 4)	EX: expression not measured (commercialized event); remark: small plots (8-row transects)

[*]**References: 170** Dively (2005) Environ Entomol 34: 1267-1291 (doi); **171** Bitzer et al. (2005) Environ Entomol 34: 1346-1376 (doi); **219** Szekeres et al. (2006) Entomol Fennica 17: 269-275 (link); **224** Rose & Dively (2007) Environ Entomol 36: 1254-1268 (doi); **244** Ludy & Lang (2006) Biol Control 38: 314-324 (doi); **514** Farinós et al. (2008) Biol Control 44: 362-371 (doi); **521** Balog et al. (2010) Crop Prot 29: 567-571 (doi); **527** Higgins et al. (2009) Environ Entomol 38: 281-292 (doi); **601** Alcantara (2012) Environ Entomol 41: 1268-1276 (doi); **603** Candolfi et al. (2004) Biocontrol Sci Technol 14: 129-170 (doi); **604** Habuštová et al. (2014) J Appl Entomol 138: 164-172 (doi); **605** Skoková Habuštová et al. (2015) J Appl Entomol 139: 31-45 (doi); **606** Svobodová et al. (2013) J Appl Entomol 137: 56-67 (doi); **610** KU Priesnitz (2010) Potential impact of *Diabrotica* resistant Bt-maize expressing Cry3Bb1 on ground beetles (Coleoptera: Carabidae). PhD thesis, RWTH Aachen University, Aachen, Germany (link); **612** M Schorling (2005) Ecological and phytomedical investigations on Bt maize grown in the European corn borer (*Ostrinia nubilalis*) infested area in the Oderbruch region (Germany). PhD thesis, University of Potsdam, Potsdam, Germany (link); **619** Manachini & Lozzia (2002) Boll Zool Agr Bachic 34: 85-96 (link); **626** Guo et al. (2014) PLOS ONE 9: e114228 (doi); **629** Szénási et al. (2014) Sci Rep 4: 5315 (doi); **631** Truter et al. (2014) Environ Entomol 43: 197-208 (doi); **632** Alcantara et al. (2010) Philipp Entomol 24: 150-164 (link); **634** Stephens et al. (2012) Agric Ecosyst Environ 156: 72-81 (doi); **635** Neher et al. (2014) Soil Biol Biochem 76: 127-135 (doi); **637** Svobodová et al. (2015) PLOS ONE 10: e0130656 (doi); **638** RI Rose (2005) An ecological risk assessment of Bt transgenic sweet corn on non-target arthropod communities. PhD thesis, University of Maryland, College Park, USA; **639** Toschki et al. (2007) Environ Entomol 36: 967-981 (doi); **644** Wang et al. (2014) J Agric Sci Technol A 4: 235-242 (link); **645** Höss et al. (2011) Sci Total Environ 409: 2674-2684 (doi); **652** S Vidal (2004) Final Report, Verbundprojekt: Sicherheitsforschung und Monitoringmethoden zum Anbau von Bt-Mais, Teilprojekt 1.2.3, Georg-August-Universität Göttingen, Göttingen, Germany (link); **653** Čerevková & Cagáň (2015) Helminthologia 52: 41-49 (doi); **654** Svobodová et al. (2020) Insect Sci 27: 375-388 (doi); **655** Svobodová et al. (2016) Bull Ent Res 106: 432-445 (doi); **656** Höss et al. (2015) Soil Biol Biochem 91: 109-118 (doi); **657** Arias-Martín et al. (2016) Agric Ecosyst Environ 220: 125-134 (doi); **660** Čerevková et al. (2018) Soil Biol Biochem 199: 194-202 (doi); **662** Xing et al. (2019) Env Sci Pollut Res 26: 5814-5819 (doi); **664** Song et al. (2019) Env Entomol 48: 263-269 (doi); **665** Liu et al. (2016) J Plant Prot 43: 384-390 (doi); **666** Szénási & Markó (2015) Crop Prot 77: 38-44 (doi); **669** Di Grumo & Lövei (2016) Period Biol 118: 223-230 (doi); **670** Guo et al. (2016) Transgenic Res 25: 761-772 (doi); **671** Guo et al. (2016) Sci Rep 6: 22102 (doi); **673** Ma et al. (2019) Biodiv Sci 27: 419-432 (doi); **674** Wang et al. (2019) J Plant Prot 46: 167-174 (doi); **904** Lit et al. (2012) Philipp Entomol 26: 28-53; **916** Priestley & Brownbridge (2009) Transgenic Res 18: 425-443 (doi); **917** Reyes et al. (2005) Asia Life Sci 14: 55-73; **920** Twardowski et al. (2017) Romanian Agric Res 34: 351-361 (link); **935** Coman & Rosca (2013) Scientific Papers A, Agronomy 56: 477-479 (link); **948** Leslie et al. (2010) Environ Entomol 39: 2045-2055 (doi); **952** LS Higgins (2011) Three-year field monitoring of Cry1F, event DAS-01507-1, maize hybrids for nontarget arthropod effects. PhD thesis, University of Nebraska, Lincoln, USA (link); **953** Guo et al. (2014) Acta Phytophylacica Sinica 41: 482-489; **960** Pálinkás et al. (2016) Ecol Evol 7: 2286-2293 (doi); **968** Lit et al. (2011) Philipp Entomol 25: 131-155

Table S3.5: Studies investigating abundance, activity density, or predation/parasitim rates, but data are not in a form useable for the quantitative data extraction (database), e.g., no measure of variation is given or means for particular experiments and defined comparisons are not available. Authors were contacted, but did not reply or were not able to provide the information needed. Labelled as N5 in Additional file 2.

**Ref.***	**Country**	**Location**	**Years**	**Experi-mentID**	**Event (Toxin)**	**Plot size (ha)**	**Comparison**	**Method (Samples per season)**	**Recorded taxa**	**Response variable**	**N**	**Observed effects**	**Critical appraisal**
911	Brazil	Rio Verde	2009	939	MON810 (Cry1Ab)	0.01	untreated Bt vs. untreated non-Bt	visual counts (6)	*Doru luteipes* (Dermaptera)	abundance	24	no statistical comparison (similar population curves in both maize hybrids, Fig. 2)	VA: no variation given; remark: small plots (20 rows × 5m);
932	Brazil	Votuporanga	2011	983	TC1507 (Cry1F); MON810 (Cry1Ab); MON89034 (Cry1A.105 & Cry2Ab2); BT11 (Cry1Ab)	0.002	5 different Bt maize lines and non-Bt lines; 3 insecticide experimental treatments: thiametoxam (seed treatment); imidacloprid (seed treatment); imidacloprid + beta-cyfluthrin (1 spray at seedling stage)	visual counts	*Dichelops melacanthus* (Het: Pentatomidae)	damage rate	4	Bt maize had lower % attacked plants, less symptoms of injury and higher plants	VA: no variation given; EX: expression not measured (commercialized events); remark: plot size 4 rows × 4m
966	China	Gongzhuling, Jilin Province	2014, 2015	940, 941	IE09S034 (Cry1Ie)	0.015	untreated Bt vs. untreated non-Bt	soil extraction (5), soil hand sorting (5)	all taxa	abundance	3	individual taxa not compared statistically	VA: no variation given; EX: expression not measured (non-commercialized event)
913	Denmark/ France/ France	Foulum/ Varois / Narbons	2002, 2003 / 2002, 2003 / 2003	82, 103/ 95, 112/ 110	MON810 (Cry1Ab)	0.13/ 0.02/ 0.02	untreated Bt vs. untreated non-Bt	soil extraction (2/ 1,2/ 2)	nematodes	abundance	4	no effects (Tab. 1)	VA: no variation given; SD only 1-2 sampling dates
914	Denmark/ France/ France	Foulum/ Varois/ Narbons	2004, 2005	942, 943/ 119, 944/ 945, 946	MON810 (Cry1Ab)	0.13/ 0.02/ 0.02	Foulum & Varois: untreated Bt vs. untreated non-Bt Narbons: Bt maize and non-Bt maize treated with the same pesticides suitable for Bt production	soil extraction (2, 3)	nematodes	abundance	4	Foulum: less nematodes in Bt maize in one of 5 samples. Varois & Narbons: no effects (Tab. 1)	VA: no variation given; SD: only 2-3 sampling dates; remark: pesticide treatments in Narbons were not specified
903	France	Montesquieu-Lauragais	2001	947	MON810 (Cry1Ab)	0.1	untreated Bt vs. untreated non-Bt and non-Bt with 1 spray of deltamethrin	visual counts (16); pitfall traps (13)	visual counts: non-target pests, predators:	abundance; activity density	3	untreated: no effects (Fig. 1-6); after insecticide spraying more herbivores and beneficials in Bt maize, by end of season, less spider	VA: no variation given; EX: expression not measured (commercialized event); remark: no seasonal means presented, but data before spray treatment,

											mites on Bt maize. No effects on Carabidae	after treatment and at end of season;
225	Germany	Bavaria	2000-2003	947, 50, 73, 97	MON810; Bt176 (Cry1Ab)	0.15	untreated Bt vs. untreated non-Bt	soil and root extraction (2)	nematodes (*Pratylenchus*)	abundance	2-5	no effects (Chap. 3.2.10,)	RE: locations cannot be used as replicates because presented data are based on different numbers of samples for each location
648	Germany	Braunschweig	2008, 2010	144, 154, 163	MON89034 × MON88017 (Cry1A.105 & Cry2Ab & Cry3Bb1)	0.13	untreated Bt vs. untreated non-Bt and non-Bt with 1 soil treatment of pyrethroid	soil extraction (1)	Lumbricidae	abundance	8	no effects (Fig. 57, 60)	VA: means per plot and variation is given, but unclear if error bars represent SD or SE; SD: only 1 sample
901	Germany	Würzburg	2005-2007	128, 136, 143	MON88017 (Cry3Bb1)	0.13	untreated Bt vs. untreated non-Bt	emergence traps (ca. 17); underground pit traps (ca. 17)	Diptera, Carabidae	density, hatching rate	8	emergence traps: Chloropidae (frit flies) and *Aphidoletes aphidimyza* (aphid feeding gall midge) higher in Bt maize, no effects on Carabidae; pit traps: total number of carabids lower in Bt maize	DP: data not fully presented, only results briefly explained in the text. Two figures, one on hatched ground beetles, one on *Aphidoletes aphidimyza* available. Selective presentation on 2 figures showing effects, rest of data summarized in text. Also species number; species diversity and eveness measured, but no data presented except one sentence
52	Italy	Pavia, Treviso	1997, 1998	14, 21	Bt176 (Cry1Ab)	10	untreated Bt vs. untreated non-Bt	Malaise trap (?)	Lepidoptera, Coleoptera, Staphylinidae, Hymenoptera, Cicadellidae, other Homoptera, Hymenopteran parasitoids, Syrphidae	activity density	2	no effects (Tab. 4)	RE/FC: 2 locations 250km distance, each 1 field Bt/non-Bt, 4 subplots as replicates by author. VA: no variation given
961	Mexico	El Camalote, Oso Viejo	2013	948, 949	MIR162 (Cry1Ab, Vip3Aa20, mCry3A)	0.004	untreated Bt vs. untreated non-Bt and non-Bt with 2 applications of emamectin benzoate	visual inspections (every 2 weeks season-long)	*Chaetocnema pulicaria* (Chrysomelidae)	abundance	4	no effects (Tab. 1)	VA: no variation given; remark: small plots (10 rows × 5m),
963	Mexico	El Camalote, Oso Viejo	2013	948, 949	MIR162 (Cry1Ab, Vip3Aa20, mCry3A)	0.004	untreated Bt vs. untreated non-Bt and non-Bt with 2 applications of emamectin benzoate	visual inspections (every 2 weeks season-long)	*Orius insidiosus*, *Chrysoperla carnea*, *Coleomegilla maculata*	abundance	4	no effects (Tab. 1)	VA: no variation given; remark: small plots (10 rows × 5m),

968 904	Philippines	Isabela, Pangasinan, South Cotabato	2009	927, 928, 929	MON89034; MON89034 × NK603 (Cry1A.105 & Cry2Ab2)	0.01	untreated Bt vs. untreated non-Bt and non-Bt with 1 spray of carbofuran and cypermethrin	sweep net; visual counts; aspirator; pitfall traps; sugar or tuna flakes baits (3 each)	canopy dwelling and ground dwelling arthropods	abundance; activity density	3	no effects (ref. 904: Fig. 1, 3, ref. 968: Fig. 12)	EX: expression not measured (commercialized event); remark: small plots (10 rows × 10m); not in database because data were not presented on taxa level, but on very general level (canopy dwelling or ground dwelling arthropods; predators & parasitoids)
955	Philippines	Isabela, South Cotabato	2010	950, 951	MON89034; MON89034 × NK603 (Cry1A.105 & Cry2Ab2);	0.01	untreated Bt vs. untreated non-Bt	sweep net; visual counts; aspirator; pitfall traps; sugar or tuna flakes baits (3 each)	canopy andground-dwelling arthropods	abundance; activity density	3	no overall effects (Fig. 3), no consistent effects on different taxa and guilds (Fig. 5-14)	EX: expression not measured (commercialized event); remark: small plots (10 rows × 10m); not in database because data were not presented on taxa level, but on very general level (canopy dwelling or ground dwelling arthropods; predators & parasitoids)
917	Philippines	Isabela, Camarines Sur	2002	930, 931	MON810 (Cry1Ab)	0.0045	untreated Bt vs. untreated non-Bt or non-Bt sprayed twice with carbofuran. Additional in Camarines Sur 1 Karate spray in both treatments	visual counts; sweep net (5)	neutral, phytophagous, predatory, and parasitoid species	abundance	3	no differences (Fig. 18)	VA: no variation given; EX: expression not measured (commercialized event); remark: small plots: 6 rows × 10m
677	Romania	Nadlac	2009	159	Bt11 × MIR604 × GA21 (Cry1Ab & mCry3A)	0.063	untreated Bt vs. untreated non-Bt and non-Bt treated with Pyrinex 48 EC (chlorpyrifos)	pitfall traps (5), sticky traps (5), (visual counts, 4)	all taxa	activity density	3.5	untreated: no effects; insecticide treated: no effects for pitfall and sticky traps. Visual counts revealed lower *Elymana sulphurella* (Cicadellidae) and *Phyllotreta nemorum* (Chrysomelidae) in treated control plots versus untreated control plots	VA: one insecticide treated plot was discarded because it was sown with another maize cultivar. Unclear if statistics were done without this plot. No plot-to-plot data available, no variation available; remark: data on visual counts of untreated plots in database
933	Romania	not specified	2009, 2010	987, 988	21 transgenic hybrids (glyphosate-tolerant, Coleoptera-, Lepidoptera resistant)	0.002	untreated Bt, untreated non-Bt	visual counts (8)	all taxa	abundance	4	no differences between conventional and transformed plants (all non-Bt and all Bt hybrids pooled together; no statistical analysis mentioned)	RC: used hybrids and events not described, data pooled for 21 transgenic and 6 conventional hybrids; EX: expression not measured; VA: no variation given; remark: plot size 4 rows × 7m
934	Romania	Troian	2011, 2012	989, 990	7 transgenic hybrids (glyphosate-tolerant,	0.002	untreated Bt, untreated non-Bt	sticky traps (15)	*Chrysoperla carnea*	activity density	4	no differences between conventional and transformed plants (all non-Bt and all Bt hybrids pooled	RC: used hybrids and events not described, data pooled for 7 transgenic and 7 conventional hybrids;

					Coleoptera-, Lepidoptera resistant)							together; no statistical analysis mentioned)	EX: expression not measured; VA: no variation given; remark: plot size 4 rows × 7m
935	Romania	Troian	2011, 2012	989, 990	7 transgenic hybrids (glyphosate-tolerant, Coleoptera-, Lepidoptera resistant)	0.002	untreated Bt, untreated non-Bt	pitfall traps (8)	Carabidae	activity density	4	no differences between conventional and transformed plants (all non-Bt and all Bt hybrids pooled together; no statistical analysis mentioned)	RC: used hybrids and events not described, data pooled for 7 transgenic and 7 conventional hybrids; EX: expression not measured; VA: no variation given; remark: plot size 4 rows × 7m
905	South Africa	Fort Hare	2009, 2010	952, 953	2 lines of MON810 (Cry1Ab)	0.01	untreated Bt vs. untreated non-Bt	soil extraction (3)	earthworms	abundance	3	no effects (Tab. 1)	VA: no variation given; EX: expression not measured (commercialized event); SD: only 3 sampling dates; remark: small plots (12 × 7 m)
902	Spain	Lleida	2000-2009	38, 61, 86, 122, 954-963	15 trials with several events, most stacked with Lepidoptera and Coleoptera activity	0.1-0.5	untreated Bt vs. untreated non-Bt	visual counts; pitfall traps; sticky traps (4-8 each)	*Orius*, *Nabis*, Carabidae, Chrysopidae, Cocinellidae, Araneae, Dermaptera, Staphylinidae, Collembola, Myriapoda, Cicadellidae, Fulgoroidea, Aphididae, Ichneumonidae, Mymaridae, Chalcidoidea, Chloropidae, Muscoidea	abundance, activity density	3-4	meta-analysis over all events, trials and years showed no effects on any taxon with any method	remark: meta-analyses over different years and events, data not presented for individual trials
952	USA	Johnston, IA, York NE	2001, 2002	979-982	(Cry34/35Ab1) 2 events	0.026	untreated Bt vs. untreated non-Bt and non-Bt with soil insecticide (Force 3G) or foliar insecticide (Capture)	visual counts (7); sticky cards (5); pitfall traps (10); soil core samples(10)	all taxa	abundance; activity density	2	no statistical analysis	VA: no variation given; EX: expression not measured (event unknown); RE: only 2 replicates

952	USA	Johnston IA / York NE / Frankfort IN	2005/ 2006-2007/ 2006-2007	934/ 935-936/ 937-938	DAS59122 (Cry34/35Ab1); DAS1507 × DAS59122 (Cry1F & Cry34/35Ab1)	0.2/ 0.4/ 0.4	untreated Bt vs. untreated non-Bt and non-Bt with Poncho seed treatment	visual counts; sticky cards; pitfall traps (3); litterbags (1)	all taxa	abundance; activity density	4	2005: fewer Carabidae in one Bt hybrid (pitfall traps); 2006: more ladybird larvae in one Bt hybrid; 2007: globular collembola less in one Bt hybrid, leafhoppers and thrips less in both Bt hybrids (Tab. 4-7); Comparison Bt vs. INS was not done by the authors.	VA: no variation given; EX: expression not measured (commercialized event): SD: only 3 samples (visual counts, sticky cards, pitfall traps) or 1 sample (litterbags) per year
900	USA	Kilbourne IL	1997	984	Bt176; MON810; BT11	0.01	untreated Bt vs. untreated non-Bt	visual counts (1)	Nitidulidae, *Orius*, Coccinellidae, aphids	abundance	6	no effects on any taxa for MON810 and BT11, Bt176 more aphids on Bt, no effects on Nitidulidae, Orius, Coccinellidae (Tab. 4)	VA: no variation given; SD: only 1 sample; remark: small plots (4 rows)
918	USA	Auburn IL	1997, 1998	974, 975	unknown (Cry1Ab)	16-23	untreated Bt vs. untreated non-Bt	visual collection	*Macrocentrus cingulum (=grandii)*	abundance	2	first generation *O. nubilalis*: no effect (Tab. 1) second generation: 1997 no effect, 1998 no parasitism in Bt maize, 8.6% in non-Bt maize (Tab. 2)	VA: no variation given; RC: commercial-scale fields, likely non-Bt and Bt not related; FC: no information on position of fields; HM/IB/PD: no information on field management; SS/LN: collected larvae in Bt less then 10% of those collected in non-Bt maize; parasitism <15%, thus sample size from Bt maize very low., SD: number of weeks sampled per generaton is unclear

919	USA	Havana IL	2001-2003	976, 977, 978	BT11 (Cry1Ab)	0.06	untreated Bt vs. untreated non-Bt	plant removal (1)	sap beetles; *Orius*	abundance	3-4	less sap beetles in Bt in 2 of 3 years; more *Orius* in Bt in 1 of 3 years (Tab. 3)	VA: no variation given; SS: only 1 sample per year; remark: plot size: 8-row strips, 80m long
90	USA	Scandia 1 KS	2001	70	MON863 (Cry3Bb1)	0.003	untreated Bt vs. untreated non-Bt	soil and root extraction (2)	nematodes (7 genera)	abundance		no effects (Tab. 4, 5)	VA: no variation given; EX: expression not measured (commercialized event); SD: only 2 sampling dates; remark: small plots (4 rows)
638	USA	Marlboro MD	2002	89	BT11 (Cry1Ab)	0.046	untreated Bt vs. untreated non-Bt and non-Bt sprayed 5 times with pyrethroid	emergence traps (4), litter bags (5)	Collembola, Diptera, fungivorous beetles, Psocoptera, parasitic Hymenoptera, predators, herbivores, mites	abundance	4	untreated: emergence traps (pp. 79-82): Collembola lower in Bt maize (Fig. 2.4), no effect on Diptera (Fig. 2.5), fungivorous Coleoptera (Fig. 2.6), Psocoptera (Fig. 2.7), parasitic Hymenoptera (Fig. 2.8);<br>litter bags: no effects on Collembola (Fig. 2.10), Oribatid mites (Fig. 2.11), predatory mites (Fig. 2.12), fungivorous beetles (Fig. 2.13);<br>insecticide treated: emergence traps (pp.79-82): Collembola and Psocoptera (Fig. 2.7) lower in Bt maize (Fig. 2.4), Diptera (Fig. 2.5) and fungivorous Coleoptera (Fig. 2.6) higher in Bt maize, no effect on parasitic Hymenoptera (Fig. 2.8)<br>litter bags: fungivorous beetles higher in Bt maize (Fig. 2.13), no effects on Collembola (Fig. 2.10), Oribatid mites (Fig. 2.11), predatory mites (Fig. 2.12)	VA: means and error bars in the figures overlapping for different treatments, not possible to estimate seasonal mean in a reliable way
908	USA	Mead, Clay Center, Concord (Nebraska)	2007, 2008	967-972	(Cry1Ab); (Cry1Ab & Cry3Bb1)	0.01	untreated Bt vs. untreated non-Bt and non-Bt sprayed once with permethrin	visual counts (1-2); sticky cards (1); plant removal (1)	*Orius insidiosus*	abundance, activity density	4	untreated: no consistent effects (some comparisons higher in Bt, others lower, most no effect) (Tab. 1-3);	VA: no variation given; SD: only 1 or 2 samples per year; remark: small plots (8 rows × 10m),

							against first generation *O. nubilalis*; in another experimental treatment bifenthrin (1 spray) against second generation.					insecticide treated: more or neutral in Bt maize with visual observations (Tab. 1) and sticky traps (Tab. 2), lower or neutral with plant removal (Tab. 3)	different comparisons of life stages, methods, several Bt events and several insecticide treatments.
912	USA	Freeville NY	2002, 2003	83, 104	MON863 (Cry3Bb1)	0.4	untreated Bt vs. untreated non-Bt and non-Bt with 1 soil application of tefluthrin. Imidacloprid seed treatment for all seeds	seed predation arenas (4)	seed predators	seed predation	3-4	less seed predation in Bt maize for 1 of 3 weed species (Fig. 1); no effects for insecticide treated plots	VA: no variation given; remark: data summarized for 3 different subplots (exclosure of large animals) and 2 years
915	USA	Freeville NY	2002, 2003	83, 104	MON863 (Cry3Bb1)	0.23	untreated Bt vs. untreated non-Bt and non-Bt with 1 soil application of tefluthrin. Imidacloprid seed treatment for all seeds	2002: sticky traps (11); 2003 visual counts (?)	2002: *Harmonia axyridis*; 2003: Cocinellidae; Aphididae	2002: activity density; 2003: abundance	3-4	2002: fewer *H. axyridis* in Bt maize (Tab. 3) 2003: no effect on Coccinellids (Tab. 4), less aphids on Bt maize (Tab. 5, 8)	VA: no variation given; DP: not sure if 2002 data were corrected for unbalanced design (not mentioned in Table legend); 2003 data: differences were rather small, but all significant.
907	USA	Pennsylvania	2001	966	unknown (Cry1Ab)	0.01	untreated Bt vs. untreated non-Bt	sticky traps (16)	*Macrocentrus cingulum*	activity density	4	50% less in Bt maize (Fig. 4)	VA: no variation given; remark: small plots (12 × 7m); no information on transformation event
910	USA	Charleston SC	2000	973	BT11 (Cry1Ab)	0.004	untreated Bt vs. untreated non-Bt	aspirator (1)	herbivores and beneficial arthropods (14 taxa)	abundance	6	no effects (Tab. 3, 4)	VA: no variation given; SD: 1 sample; LN: non-targets collected in low and variable numbers; remark: small plots (7.3 × 6.1m), 3 planting dates;
906	USA	Brookings SD	2001, 2002	964, 965	MON863 (Cry3Bb1)	1.6	untreated Bt vs. untreated non-Bt and non-Bt with 1 soil application of tefluthrin. Imidacloprid seed treatment in Bt and non-Bt maize, not in the tefluthrin treated maize	sticky traps (9); visual counts (9)	Coccinellidae	activity density, abundance	4	generally more *C. maculata* in Bt maize (Fig. 1-5)	VA: no variation given; EX: expression not measured (commercialized event)
916	USA	Burlington VT	2003, 2004	932, 933	MON810 (Cry1Ab)	0.25	untreated Bt vs. untreated non-Bt	pitfall traps (8-9); soil extraction (7-8)	Carabidae, Collembola	activity density, abundance	5	no effect of Bt maize on carabids (Fig. 1a) and surface-dwelling Collembola (Fig. 1b). Fewer subterranean Collembola in Bt maize (3 species) (Fig. 1c, 2)	VA: no variation given; remark: small plots: 8-row transects

*References: 52 Lozzia (1999) Boll Zool Agr Bachic 31:37-58; 90 Al-Deeb et al. (2003) Environ Entomol 32: 859-865 (doi); 225 Lang et al. (2005) Monitoring der Umweltwirkungen des Bt-Gens. Bayerische Landesanstalt für Landwirtschaft, Freising-Weihenstephan, Germany. LfL Schriftenreihe 7 (link); 638 RI Rose (2005) An ecological risk assessment of Bt transgenic sweet corn on non-target arthropod communities. PhD thesis, University of Maryland, College Park, USA; 648 E Schultheis (2011) Faunistic approach for an environmental risk assessment of a Bt-maize with multiple resistance genes and evaluation of *Trigonotylus caelestialium* (KIRKALDY) and *Lumbricus terrestris* LINNAEUS as model organisms. PhD thesis, RWTH Aachen University, Aachen, Germany (link); 677 KF Lauer & H Graepel (2010) Potential effects of Bt11 × MIR604 × GA21 maize on non-target arthropods – Romania. Syngenta MIR604 maize, Part I: Technical Dossier, Appendix 39. Final Report, EEX-100-10.; 900 Dowd (2000) J Econ Entomol 93: 1669-1679 (doi); 901 W Büchs (2009) Auswirkungen von Bt-Mais auf Trauermückenlarven als Zersetzer. bioSicherheit, Gentechnik – Pflanzen – Umwelt (Webpage) (link); 902 Comas et al. (2014) Transgenic Res 23: 135-143 (doi); 903 Naïbo & Marque (2003) Perspectives Agricoles 293: 14-17; 904 Lit et al. (2012) Philipp Entomol 26: 28-53; 905 Kamota et al. (2012) African J Biotechnol 11: 15168-15170 (link); 906 McManus et al. (2005) J Econ Entomol 98: 1992-1998 (doi); 907 SL Sked & DD Calvin (2002) California Conference on Biological Control III, 15-16 August 2002, Berkeley, USA; 908 Palizada et al. (2014) Pak Entomol 36: 1-6 (link); 910 Hassell & Shepard (2002) J Entomol Sci 37: 285-292 (doi); 911 De Araujo et al. (2011) Revista Brasileira de Milho e Sorgo 10: 205-214 (link); 912 DiTommaso et al. (2014) Weed Sci 62: 619-624 (doi); 913 Griffiths et al. (2005) Plant Soil 275: 135-146 (doi); 914 Griffiths et al. (2007) Pedobiologia 51: 195-206 (doi); 915 EJ Stephens (2006) Direct and indirect non-target effects of Cry3Bb Bt corn. PhD thesis, Cornell University, NY, USA; 916 Priestley & Brownbridge (2009) Transgenic Res 18: 425-443 (doi); 917 Reyes et al. (2005) Asia Life Sci 14: 55-73; 918 Venditti & Steffey (2002) 1st International Symposium on Biological Control of Arthropods, 14-18 January 2002, Honolulu, Hawaii, USA, 278-283 (link); 919 Dowd (2005) J Econ Entomol 98: 856-861 (doi); 932 J Crosariol Netto (2013) Infestation and damages of *Dichelops* melacathus (Dallas, 1851) (Heteroptera: Pentatomidae) on transgenic and conventional maize hybrids submitted to chemical control. PhD thesis, Universidade Estadual Paulista "Júlio de Mesquita Filho", São Paulo, Brasil (link); 933 Coman (2012) Scientific Papers A, Agronomy 55: 133-136 (link); 934 Coman & Rosca (2013) Scientific Papers A, Agronomy 56: 480-483 (link); 935 Coman & Rosca (2013) Scientific Papers A, Agronomy 56: 477-479 (link); 952 LS Higgins et al. (2011) Field monitoring of Bt maize for non-target organism effects. PhD thesis, University of Nebraska, Lincoln, USA (link); 955 Lit et al. (2016) Philipp Entomol 30: 29-51; 961 Hernandez-Juarez et al. (2018) Southwestern Entomol 43: 841-846 (doi); 963 Hernandez-Juarez et al. (2019) Florida Entomol 102: 96-100 (doi); 966 Fan et al. (2019) Sci Rep 9: 10333 (doi); 968 Lit et al. (2011) Philipp Entomol 25: 131-155
