# Supplementary material for: Does the growing of Bt maize change abundance or ecological function of non-target animals compared to the growing of non-GM maize? A systematic review
Source: Environ Evid. 2022 Jun 6;11:21. doi: 10.1186/s13750-022-00272-0 (PMC11378853; doi:10.1186/s13750-022-00272-0)
Supplement: Supplementary file 4 — Additional file 4: Rules for extraction of data for the database. [file 13750_2022_272_MOESM4_ESM.pdf]

Does the growing of Bt maize change abundance or ecological function of non-target animals compared to the growing of non-GM maize? A systematic review

Michael Meissle[1*], Steven E. Naranjo[2], and Jörg Romeis[1]
[1]Agroscope, Research Division Agroecology and Environment, Reckenholzstrasse 191, 8046 Zurich, Switzerland, michael.meissle@agroscope.admin.ch; joerg.romeis@agroscope.admin.ch
[2]USDA-ARS, Arid-Land Agricultural Research Center, 21881 North Cardon Lane, Maricopa 85138, Arizona, USA, steve.naranjo@usda.gov

[*] Corresponding author

Published in: Environmental Evidence (2022), https://doi.org/10.1186/s13750-022-00272-0

Additional file 4: Rules for extraction of data for the database

The following rules were established to determine which data to enter into the database:

Taxonomic level: Usually, the authors of an experiment make a decision on which taxonomic levels they analyze, report and publish. For the database, data were entered at the finest taxonomic resolution possible, i.e., entering data on the same level as the authors did in their papers. If data were available for different taxonomic levels (e.g., several individual carabid species and also the total sum of carabids), data were entered for both levels. To be able to perform meta-analyses on comparable taxonomic levels, data on lower taxonomic levels were aggregated and the aggregated values also were entered in the database. Taxonomic levels for aggregation were chosen depending on the availability of data and the similarity of the lifestyle of the species within a taxonomic group. The groups used were: Nematoda (phylum); Myriapoda (subphylum); Acarina, Oligochaeta, and Collembola (subclass); Araneae, Opiliones, Dermaptera, Mecoptera, Neuroptera, Orthoptera, Psocoptera, and Thysanoptera (order); Anthicidae, Cantharidae, Carabidae, Chrysomelidae, Cicindelidae, Coccinellidae, Elateridae, Lathrididae, Nitidulidae, Scarabaeidae, and Staphylinidae (Coleoptera family); Chironomidae, Chloropidae, Dolichopodidae, Otitidae, Tachinidae, and Syrphidae (Diptera family); Aphididae, Anthocoridae, Cicadellidae, Delphacidae, Geocoridae, Miridae, Nabidae, and Pentatomidae (Hemiptera family); and Braconidae, Formicidae, Ichneumonidae, Mymaridae, and Vespidae (Hymenoptera family).

Data presented in articles (tables, figures) were entered, even if the total number of recorded individuals of a taxon was low. Such records with less than 20 individuals over all plots (Bt and non-Bt) and sampling dates per season were flagged “red” in the critical appraisal and thus excluded in most meta-analyses. This threshold was derived from experience and practicability. The aim was to find a threshold that did not exclude too many datasets, but ensured that the number of replicates with a zero seasonal mean in either Bt or non-Bt maize was minimized. With most experiments, the defined threshold of 20 worked well in this

regard. For extensive faunistic studies, where the full dataset from authors or supplementary material became available, there was often a multitude of taxa with low abundance. Entering all taxa with low abundance would have been very time consuming and the value of those data for meta-analyses would have been low (“red label”). Therefore, taxa with less than 20 individuals recorded over all plots and sampling dates were not entered from full datasets.

Aggregations to higher taxonomic units (e.g., family or order level) were made based on biology (e.g., species with similar lifestyle). For example, if a study provided data for individual species, we entered individual species (if more than 20 individuals were recorded over all plots and sampling dates) as well as data aggregated to family level. Similarly, if separate records for life stages, e.g., juveniles and adults were available, individual life stages were entered (if more than 20 individuals of the respective stage were recorded) as well as data aggregated to “all stages”.

Seasonal means: Records in the database were generally based on the mean value of one growing season (usually one year except in tropical areas where two seasons per year are possible). In the rare event that no yearly data were available, combined data for two or more years were entered. When raw data were available, the mean over subsamples and sampling dates was first calculated for each true replicate (plot or field) separately, resulting in seasonal plot-by-plot values (without SD). Then, the mean of those seasonal values per plot and the corresponding SD were calculated for each treatment and entered in the database (SD for N plots). In several cases, means and SDs or SEs were available for individual sampling dates, but not for the whole season. In contrast to Marvier et al. [Ref. 10 in the main manuscript], we did not rely on the peak day method whenever possible. Selecting data from one sampling day only would result in the loss of information available for the other days. Instead, we calculated the mean values and a mean SD over all sampling dates, e.g., $SD_{season} = (SD_{Date1} + SD_{Date2} + SD_{Date3}) / 3$. This approximation generally resulted in an overestimation of the true SDs compared with the preferred replicate-by-replicate method described above. The resulting estimated SD thus can be considered more conservative. In some studies, seasonal means were given per plant or trap instead of per plot or field and the SEs accompanying those means were usually also calculated based on N plants or traps and not on true experimental units (plots or fields). In these cases, the SDs entered into the database were back-calculated to SDs based on the number of true replicates. This created bias because the SE based on individual plants or traps involves pseudoreplication and is generally lower than the SE calculated from true replicates. For transparency, we noted in the database if SDs were estimated or if it was uncertain how the authors calculated the reported values.

Multiple use of data: Data for the database were extracted as inclusively as possible. When multiple Bt lines were compared to one non-Bt line, each Bt / non-Bt comparison was entered in the database such that the values of the non-Bt line were used several times. The same applied if untreated Bt maize was compared to a range of different insecticide treatments in non-Bt maize. Furthermore, data for different sampling methods, different life stages of the same taxon, and different levels of taxonomy were entered whenever available and appropriate, as described above. For these latter categories, separate specific analyses were conducted to provide a more complete assessment of available data.
