# Supplementary material for: Does the growing of Bt maize change abundance or ecological function of non-target animals compared to the growing of non-GM maize? A systematic review
Source: Environ Evid. 2022 Jun 6;11:21. doi: 10.1186/s13750-022-00272-0 (PMC11378853; doi:10.1186/s13750-022-00272-0)
Supplement: Supplementary file 7 — Additional file 7: Detailed results of statistical meta-analyses (14 Tables, 2 Figures). [file 13750_2022_272_MOESM7_ESM.pdf]

Does the growing of Bt maize change abundance or ecological function of non-target animals compared to the growing of non-GM maize? A systematic review

Michael Meissle[1*], Steven E. Naranjo[2], and Jörg Romeis[1]
[1]Agroscope, Research Division Agroecology and Environment, Reckenholzstrasse 191, 8046 Zurich, Switzerland, michael.meissle@agroscope.admin.ch; joerg.romeis@agroscope.admin.ch
[2]USDA-ARS, Arid-Land Agricultural Research Center, 21881 North Cardon Lane, Maricopa 85138, Arizona, USA, steve.naranjo@usda.gov

[*] Corresponding author

Published in: Environmental Evidence (2022), https://doi.org/10.1186/s13750-022-00272-0

Additional file 7: Detailed results of statistical meta-analyses (14 Tables, 2 Figures)

Table S7.1: Main meta-analyses with all Bt proteins and records with any “red” critical appraisal label excluded. Given is the analyzed taxon, the estimated effect size (estimate) with standard error (SE) and 95% confidence interval (lower boundary; upper boundary), the measure of heterogenetiy (Q value and significance, p<0.05 is significant), the number of records (rec), experiments (exp) and articles (art) included in the respective analysis, and the number of records per article (articleID in parenthesis). Significant heterogeneity and significant effect sizes (confidence intervals do not include zero) are marked in bold.

Taxon	Estimate ± SE [ci.lb; ci.ub]	Q (p)	Rec	Exp	Art	Records per article
**all Taxa**	-0.029 ± 0.02 [-0.06; 0.003]	1822.8 (0.99)	1976	214	107	353(675), 155(629), 124(173), 96(659), 80(169), 59(88), 47(516, 603), 46(647), 42(527), 40(225), 38(224), 37(600), 33(626, 652), 28(182), 26(170, 637), 25(166), 24(167), 22(507), 21(90, 230, 648), 19(631, 632, 678), 18(630, 673), 16(175, 601, 674), 15(604), 14(676), 13(38), 12(620, 650, 658, 662), 11(655), 10(612, 614, 677), 9(75, 144, 605, 672), 8(211, 213, 504, 621, 646, 668), 7(10, 228), 6(4, 31, 214, 246, 519, 521, 624, 628, 639, 640, 657, 660, 667), 5(613), 4(171, 514, 615, 663), 3(219, 244, 606, 610, 633, 634, 638, 649, 654), 2(86, 524, 602, 608, 617, 622, 635, 642, 653, 656, 666), 1(83, 208, 215, 508, 609, 618, 619, 636, 643, 644, 645, 651, 665, 669)
Nematoda	-0.233 ± 0.13 [-0.49; 0.02]	18.3 (0.31)	17	17	8	6(660), 2(608, 635, 653, 656), 1(90, 619, 645)
Oligochaeta	0.039 ± 0.18 [-0.32; 0.4]	6.2 (0.96)	15	10	7	6(628), 2(144, 225, 674), 1(508, 624, 636)
Acarina	0.055 ± 0.12 [-0.18; 0.29]	24.5 (0.97)	40	34	14	11(90), 8(675), 4(504), 3(527,657), 2(175, 674), 1(144, 603, 609, 622, 624, 633, 678)
Araneae	0.048 ± 0.05 [-0.05; 0.15]	138.5 (1)	192	115	41	33(675), 21(173), 20(629), 8(169, 659), 6(88, 182), 5(224, 225, 614), 4(170, 507, 516, 601), 3(166, 244, 519, 527, 600, 605, 606, 637, 639, 647, 662, 672), 2(167, 175, 603, 626, 646, 673, 676, 678), 1(75, 215, 228, 230, 620, 674, 677)
Opiliones	0.449 ± 0.43 [-0.39; 1.29]	3.1 (0.55)	5	5	3	3(519), 1(516, 603)
Myriapoda	-0.031 ± 0.19 [-0.41; 0.35]	7.5 (0.91)	15	15	8	3(166, 514, 527), 2(516), 1(603, 624, 674, 678)
Collembola	-0.050 ± 0.08 [-0.20; 0.10]	48.2 (1)	88	59	22	29(675), 10(629), 9(90), 4(171, 225, 504), 3(527, 657), 2(88, 170, 175, 213, 516, 673, 674), 1(603, 622, 624, 644, 647, 651, 665, 678)
**Coleoptera**	-0.030 ± 0.03 [-0.09; 0.03]	512.4 (0.39)	505	160	69	
Anthicidae (Col.)	-0.012 ± 0.21 [-0.43; 0.40]	13 (0.3)	12	9	4	6(675), 4(169), 1(659, 676)
Cantharidae (Col.)	0.501 ± 0.32 [-0.12; 1.13]	2.9 (0.72)	6	6	3	3(224), 2(516), 1(603)
Carabidae (Col.)	0.001 ± 0.07 [-0.13; 0.13]	76 (1)	113	92	35	22(675), 10(629), 6(88, 640), 4(169, 224, 615), 3(166, 219, 507, 527, 605, 610, 634, 639, 649, 654, 672), 2(170, 175, 516, 612, 614, 642, 673), 1(228, 603, 613, 620, 643, 646, 659, 669, 676, 678)
Chrysomelidae (Col.)	-0.176 ± 0.11 [-0.39; 0.04]	62.2 (0.11)	51	33	11	24(675), 10(629), 4(169), 3(167), 2(516, 666, 673), 1(620, 626, 647, 677)
Cicindelidae (Col.)	0.198 ± 0.29 [-0.36; 0.76]	5.3 (0.51)	7	6	3	4(169), 2(507), 1(88)
Coccinellidae (Col.)	0.063 ± 0.06 [-0.06; 0.19]	106.9 (0.83)	123	102	36	21(173), 10(629), 8(88, 659), 5(182, 652), 4(169, 601), 3(75, 167, 224, 527, 604, 637, 647, 648, 662), 2(10, 31, 38, 170, 211, 246, 507, 516, 600, 621, 626, 673), 1(4, 230, 603, 620, 646, 676, 677)
Elateridae (Col.)	-0.105 ± 0.18 [-0.46; 0.25]	27.2 (0.17)	22	16	7	7(629), 6(88), 3(169), 2(175, 516), 1(603, 676)

Lathrididae (Col.)	0.797 ± 0.77 [-0.72; 2.31]	**39.1 (<0.0001)**	8	8	4	3(213, 647), 1(144, 603)
Nitidulidae (Col.)	-0.146 ± 0.15 [-0.43; 0.14]	31.6 (0.14)	25	25	7	11(659), 4(169), 3(166), 2(86, 170, 516), 1(230)
Scarabaeidae (Col.)	-0.268 ± 0.29 [-0.84; 0.31]	14.6 (0.1)	10	9	5	4(169), 2(516, 675), 1(88, 166)
Staphylinidae (Col.)	**-0.184 ± 0.08 [-0.34; -0.02]**	75.7 (0.61)	81	62	24	20(675), 8(629), 6(88), 4(169, 224, 663), 3(166, 521, 527, 605, 655, 672), 2(170, 175, 507, 516, 524), 1(182, 228, 230, 603, 646, 676, 678)
Dermaptera	-0.159 ± 0.14 [-0.43; 0.11]	24.3 (0.71)	30	20	5	14(675), 10(659), 4(507), 1(646, 676)
**Diptera**	**-0.186 ± 0.05 [-0.29; -0.08]**	197.2 (0.07)	170	78	26	
Chironomidae (Dip.)	-0.014 ± 0.22 [-0.45; 0.42]	4.2 (0.52)	6	6	3	3(647), 2(655), 1(600)
Dolichopodidae (Dip.)	0.088 ± 0.23 [-0.36; 0.54]	6.6 (0.68)	10	8	5	4(675), 3(527), 1(603, 655, 659)
Otitidae (Dip.)	-0.236 ± 0.16 [-0.55; 0.08]	**43 (0.05)**	30	21	3	20(675), 6(659), 4(169)
Syrphidae (Dip.)	-0.173 ± 0.09 [-0.35; 0.00]	79.7 (0.06)	63	42	15	21(173), 10(629), 7(652), 6(675), 3(167, 637, 650), 2(600, 648), 1(38, 230, 601, 603, 626, 659)
Tachinidae (Dip.)	**-0.492 ± 0.17 [-0.83; -0.15]**	21.5 (0.09)	15	11	5	10(675), 2(617), 1(38, 230, 618)
**Hemiptera**	0.023 ± 0.03 [-0.04; 0.09]	378 (1)	460	154	50	
Anthocoridae (Hem.)	0.116 ± 0.06 [-0.003; 0.24]	144.4 (0.38)	141	109	35	22(675), 20(173), 14(88), 10(629, 659), 6(182), 4(169, 224), 3(31, 75, 167, 527, 604, 637, 647, 648), 2(10, 170, 211, 213, 246, 516, 621, 626), 1(4, 38, 83, 228, 230, 600, 603, 646, 673, 676, 677)
Aphididae (Hem.)	-0.025 ± 0.07 [-0.16; 0.11]	78.1 (0.91)	97	77	30	10(629, 675), 7(652), 6(225, 630, 667), 4(659), 3(167, 214, 521, 527, 600, 604, 637, 647, 662), 2(38, 516, 612, 626, 632, 648, 673), 1(75, 228, 230, 603, 613, 620, 677)
Cicadellidae (Hem.)	0.032 ± 0.08 [-0.13; 0.19]	53.4 (0.97)	75	61	20	24(675), 11(659), 6(225), 4(169), 3(214, 527, 647, 648, 658), 2(516, 600, 611, 626, 632), 1(144, 230, 603, 620, 673, 676, 677)
Delphacidae (Hem.)	-0.118 ± 0.25 [-0.61; 0.37]	4.8 (0.85)	10	10	3	4(632), 3(658, 659)
Geocoridae (Hem.)	0.126 ± 0.23 [-0.32; 0.57]	10.8 (0.37)	11	9	3	6(675), 4(169), 1(659)
Miridae (Hem.)	-0.112 ± 0.12 [-0.34; 0.12]	21 (0.95)	34	20	7	14(675), 10(629), 3(647, 648), 2(626), 1(230, 676)
Nabidae (Hem.)	-0.045 ± 0.13 [-0.30; 0.21]	10.4 (1)	28	21	10	10(629), 6(182), 3(167), 2(211, 621), 1(169, 600, 603, 646, 677)
Pentatomidae (Hem.)	0.051 ± 0.24 [-0.42; 0.52]	2.7 (0.97)	10	8	5	4(675), 3(662), 1(169, 230, 516)
**Hymenoptera**	**-0.153 ± 0.06 [-0.27; -0.04]**	**204.2 (0.01)**	158	95	38	
Braconidae (Hym.)	**-1.548 ± 0.24 [-2.02; -1.08]**	**61.6 (0.0001)**	27	18	4	21(173), 3(167), 2(516), 1(230)
Formicidae (Hym.)	-0.105 ± 0.16 [-0.42; 0.21]	13.9 (0.83)	21	20	10	5(88), 4(169), 3(166), 2(175, 516), 1(601, 603, 675, 677, 678)
Vespidae (Hym.)	0.474 ± 0.31 [-0.13; 1.08]	8.3 (0.14)	6	5	3	3(675), 2(600), 1(603)
Neuroptera	0.054 ± 0.06 [-0.07; 0.18]	123 (0.48)	124	94	33	26(675), 20(173), 10(629), 7(652, 659), 4(182), 3(224, 527, 604, 647, 650), 2(10, 38, 144, 170, 211, 516, 600, 601, 621, 626, 637, 648, 673), 1(4, 75, 167, 208, 228, 230, 603, 677, 678)
Orthoptera	-0.023 ± 0.13 [-0.29; 0.24]	17.2 (0.97)	32	25	10	10(675), 4(169, 632), 3(88, 166), 2(175, 516), 1(626, 676, 678)
Thysanoptera	0.058 ± 0.08 [-0.11; 0.22]	51.9 (0.91)	68	52	21	20(675), 10(629), 4(169), 3(527, 600, 604, 637, 647, 648), 2(38, 612, 614, 626), 1(228, 230, 603, 613, 620, 676, 677, 678)

Table S7.2: Main meta-analyses for Lepidoptera-active, Coleoptera-active, or stacked Lepidoptera & Coleoptera-active Bt proteins separately and records with any "red" critical appraisal label excluded. Given is the analyzed taxon, the estimated effect size (estimate) with standard error (SE) and 95% confidence interval (lower boundary; upper boundary), the measure of heterogenetiy (Q value and significance, p<0.05 is significant), the number of records (rec), experiments (exp) and articles (art) included in the respective analysis, and the number of records per article (articleID in parenthesis). Significant heterogeneity and significant effect sizes (confidence intervals do not include zero) are marked in bold.

Taxon	Estimate ± SE [ci.lb; ci.ub]	Q (p)	Rec	Exp	Art	Records per article
**Lepidoptera-active Bt proteins**						
all Taxa	-0.024 ± 0.02 [-0.06; 0.02]	1166.1 (0.86)	1220	160	79	157(675), 124(173), 80(169), 47(516, 603), 42(527), 40(225), 38(224), 37(600), 33(626, 652), 32(629), 28(182), 26(170), 22(507), 21(230), 19(631, 632), 18(630, 673), 16(601, 674), 15(604), 13(38), 12(620, 650, 658, 662), 10(612, 614), 9(75, 605, 672), 8(211, 213, 504, 621, 646), 7(10, 228, 668), 6(4, 31, 214, 519, 521, 624, 639, 640, 657, 660, 667), 5(613), 4(514, 615, 628, 663), 3(219, 244, 633, 638), 2(86, 617, 622, 642, 653, 666), 1(83, 208, 215, 508, 618, 619, 636, 643, 644, 651, 665, 669)
Nematoda	-0.299 ± 0.15 [-0.6; 0.001]	5.6 (0.69)	9	9	3	6(660), 2(653), 1(619)
Oligochaeta	0.126 ± 0.21 [-0.29; 0.54]	4.2 (0.94)	11	8	6	4(628), 2(225, 674), 1(508, 624, 636)
Acarina	-0.022 ± 0.17 [-0.35; 0.30]	10.5 (0.94)	20	20	9	4(504, 675), 3(527, 657), 2(674), 1(603, 622, 624, 633)
Araneae	0.057 ± 0.07 [-0.07; 0.18]	75.9 (1)	124	86	30	21(173), 16(675), 8(169), 6(182), 5(224, 225, 614), 4(170, 507, 516, 601, 629), 3(244, 519, 527, 600, 605, 639, 662, 672), 2(603, 626, 646, 673), 1(75, 215, 228, 230, 620, 674)
Opiliones	0.449 ± 0.43 [-0.39; 1.29]	3.1 (0.55)	5	5	3	3(519), 1(516, 603)
Myriapoda	-0.049 ± 0.24 [-0.53; 0.43]	4.8 (0.9)	11	11	6	3(514, 527), 2(516), 1(603, 624, 674)
Collembola	-0.015 ± 0.11 [-0.22; 0.19]	25.5 (0.99)	46	42	17	14(675), 4(225, 504), 3(527, 657), 2(170, 213, 516, 629, 673, 674), 1(603, 622, 624, 644, 651, 665)
**Coleoptera**	0.005 ± 0.04 [-0.08; 0.09]	307.8 (0.35)	300	113	49	
Cantharidae (Col.)	0.501 ± 0.32 [-0.12; 1.13]	2.9 (0.72)	6	6	3	3(224), 2(516), 1(603)
Carabidae (Col.)	0.032 ± 0.09 [-0.14; 0.20]	44.4 (0.99)	68	66	25	11(675), 6(640), 4(169, 224, 615), 3(219, 507, 527, 605, 639, 672), 2(170, 516, 612, 614, 629, 642, 673), 1(228, 603, 613, 620, 643, 646, 669)
Chrysomelidae (Col.)	0.020 ± 0.16 [-0.28; 0.32]	24.2 (0.39)	24	23	8	10(675), 4(169), 2(516, 629, 666, 673), 1(620, 626)
Coccinellidae (Col.)	0.062 ± 0.08 [-0.09; 0.22]	73.7 (0.73)	83	71	27	21(173), 5(182, 652), 4(169, 601), 3(75, 224, 527, 604, 662), 2(10, 31, 38, 170, 211, 507, 516, 600, 621, 626, 629, 673), 1(4, 230, 603, 620, 646)
Elateridae (Col.)	-0.047 ± 0.27 [-0.58; 0.48]	6.5 (0.48)	8	8	4	3(169), 2(516, 629), 1(603)
Nitidulidae (Col.)	**-0.503 ± 0.23 [-0.96; -0.05]**	13.2 (0.21)	11	11	5	4(169), 2(86, 170, 516), 1(230)
Scarabaeidae (Col.)	-0.127 ± 0.33 [-0.77; 0.51]	9.4 (0.15)	7	7	3	4(169), 2(516), 1(675)
Staphylinidae (Col.)	-0.180 ± 0.11 [-0.39; 0.03]	46.6 (0.49)	48	45	17	11(675), 4(169, 224, 663), 3(521, 527, 605, 672), 2(170, 507, 516, 629), 1(182, 228, 230, 603, 646)

Dermaptera	-0.001 ± 0.22 [-0.43; 0.43]	3.7 (0.98)	12	9	3	7(675), 4(507), 1(646)
**Diptera**	**-0.261 ± 0.07 [-0.4; -0.12]**	**124.5 (0.04)**	100	54	19	
Dolichopodidae (Dip.)	-0.115 ± 0.30 [-0.71; 0.47]	3.6 (0.61)	6	6	3	3(527), 2(675), 1(603)
Syrphidae (Dip.)	-0.219 ± 0.11 [-0.44; 0.01]	**59.9 (0.03)**	42	30	11	21(173), 7(652), 3(650), 2(600, 629, 675), 1(38, 230, 601, 603, 626)
Tachinidae (Dip.)	**-0.648 ± 0.21 [-1.05; -0.24]**	11.1 (0.2)	9	9	5	4(675), 2(617), 1(38, 230, 618)
**Hemiptera**	0.044 ± 0.04 [-0.04; 0.13]	238.4 (0.94)	274	109	39	
Anthocoridae (Hem.)	**0.176 ± 0.08 [0.01; 0.34]**	96.2 (0.09)	80	69	26	20(173), 9(675), 6(182), 4(169, 224), 3(31, 75, 527, 604), 2(10, 170, 211, 213, 516, 621, 626, 629), 1(4, 38, 83, 228, 230, 600, 603, 646, 673)
Aphididae (Hem.)	-0.054 ± 0.09 [-0.22; 0.11]	53.8 (0.84)	66	56	24	7(652), 6(225, 630, 667), 3(214, 521, 527, 600, 604, 662, 675), 2(38, 516, 612, 626, 629, 632, 673), 1(75, 228, 230, 603, 613, 620)
Cicadellidae (Hem.)	0.049 ± 0.11 [-0.17; 0.27]	24 (0.98)	41	37	14	10(675), 6(225), 4(169), 3(214, 527, 658), 2(516, 600, 626, 632), 1(230, 603, 620, 673)
Miridae (Hem.)	-0.096 ± 0.23 [-0.54; 0.35]	9.9 (0.45)	11	10	4	6(675), 2(626, 629), 1(230)
Nabidae (Hem.)	-0.149 ± 0.17 [-0.48; 0.18]	5.7 (0.98)	16	16	8	6(182), 2(211, 621, 629), 1(169, 600, 603, 646)
Pentatomidae (Hem.)	-0.005 ± 0.26 [-0.52; 0.51]	0.8 (1)	8	8	5	3(662), 2(675), 1(169, 230, 516)
**Hymenoptera**	**-0.214 ± 0.07 [-0.34; -0.08]**	**176.5 (0.001)**	124	70	27	
Braconidae (Hym.)	**-1.735 ± 0.26 [-2.24; -1.23]**	**54.6 (0.0002)**	24	15	3	21(173), 2(516), 1(230)
Formicidae (Hym.)	0.074 ± 0.26 [-0.44; 0.59]	7.4 (0.5)	9	9	5	4(169), 2(516), 1(601, 603, 675)
Neuroptera	0.053 ± 0.08 [-0.10; 0.21]	83.9 (0.39)	82	70	25	20(173), 11(675), 7(652), 4(182), 3(224, 527, 604, 650), 2(10, 38, 170, 211, 516, 600, 601, 621, 626, 629, 673), 1(4, 75, 208, 228, 230, 603)
Orthoptera	0.097 ± 0.20 [-0.29; 0.48]	10.3 (0.8)	16	15	5	5(675), 4(169, 632), 2(516), 1(626)
Thysanoptera	0.108 ± 0.12 [-0.12; 0.34]	20.6 (0.97)	36	35	15	8(675), 4(169), 3(527, 600, 604), 2(38, 612, 614, 626, 629), 1(228, 230, 603, 613, 620)
**Coleoptera-active Bt proteins**						
**all Taxa**	-0.010 ± 0.03 [-0.08; 0.06]	343.4 (1)	423	47	24	
Nematoda	0.022 ± 0.27 [-0.51; 0.55]	3.1 (0.68)	6	6	4	2(608, 635), 1(90, 645)
Acarina	0.304 ± 0.21 [-0.10; 0.71]	9.4 (0.8)	15	13	4	11(90), 2(175), 1(144, 609)
Araneae	0.046 ± 0.12 [-0.20; 0.29]	41.3 (0.29)	38	28	10	8(659), 6(88, 629), 3(166, 606, 637, 647), 2(167, 175, 675)
Collembola	-0.079 ± 0.16 [-0.39; 0.23]	10.4 (0.97)	22	20	7	9(90), 4(171), 3(629), 2(88, 175), 1(647, 675)
**Coleoptera**	-0.085 ± 0.06 [-0.21; 0.04]	115.8 (0.64)	123	39	16	
Carabidae (Col.)	-0.024 ± 0.13 [-0.29; 0.24]	19.4 (0.73)	25	24	9	6(88), 3(166, 610, 629, 634, 654), 2(175, 659, 675)
Chrysomelidae (Col.)	-0.519 ± 0.31 [-1.14; 0.10]	10.7 (0.15)	8	8	4	3(167, 629), 1(647, 675)
Coccinellidae (Col.)	-0.068 ± 0.13 [-0.32; 0.18]	20.4 (0.88)	30	28	7	8(88, 659), 3(167, 629, 637, 647), 2(246)
Elateridae (Col.)	0.006 ± 0.32 [-0.62; 0.63]	15.7 (0.07)	10	9	3	6(88), 2(175, 629)
Staphylinidae (Col.)	-0.209 ± 0.17 [-0.55; 0.13]	16.1 (0.52)	18	17	7	6(88), 3(166, 655), 2(175, 629), 1(524, 675)
**Diptera**	0.040 ± 0.10 [-0.16; 0.24]	32.6 (0.63)	37	20	7	
Syrphidae (Dip.)	-0.039 ± 0.22 [-0.47; 0.39]	6.4 (0.7)	10	10	4	3(167, 629, 637), 1(659)

**Hemiptera**	0.042 ± 0.07 [-0.10; 0.18]	68.9 (0.97)	94	38	9	
Anthocoridae (Hem.)	0.012 ± 0.12 [-0.22; 0.24]	33.1 (0.69)	39	34	8	14(88), 10(659), 3(167, 629, 637, 647), 2(246), 1(675)
Aphididae (Hem.)	0.065 ± 0.16 [-0.25; 0.38]	10.4 (0.79)	16	16	5	4(659), 3(167, 629, 637, 647)
Cicadellidae (Hem.)	0.155 ± 0.17 [-0.17; 0.48]	11.2 (0.74)	16	16	4	11(659), 3(647), 1(144, 675)
Miridae (Hem.)	-0.011 ± 0.23 [-0.46; 0.43]	1.6 (0.95)	7	7	3	3(629, 647) 1(675)
**Hymenoptera**	-0.077 ± 0.13 [-0.33; 0.18]	11.6 (0.99)	27	21	8	
Formicidae (Hym.)	-0.349 ± 0.23 [-0.80; 0.10]	3.5 (0.94)	10	9	3	5(88), 3(166), 2(175)
Neuroptera	0.002 ± 0.16 [-0.30; 0.31]	16.8 (0.47)	18	18	6	7(659), 3(629, 647), 2(144, 637), 1(167)
Orthoptera	-0.093 ± 0.25 [-0.58; 0.40]	4.4 (0.82)	9	9	4	3(80, 166), 2(175), 1(675)
Thysanoptera	0.075 ± 0.20 [-0.31; 0.46]	6.4 (0.7)	10	10	4	3(629, 637, 647), 1(675)
**Stacked Lepidoptera- and Coleoptera-active Bt proteins**						
**all Taxa**	-0.069 ± 0.04 [-0.14; 0.01]	311.9 (0.78)	333	29	11	
Araneae	0.034 ± 0.13 [-0.22; 0.29]	21.2 (0.85)	30	19	5	15(675), 10(629), 2(676, 678), 1(677)
Collembola	-0.103 ± 0.16 [-0.42; 0.21]	12.2 (0.88)	20	18	3	14(675), 5(629), 1(678)
**Coleoptera**	-0.066 ± 0.08 [-0.22; 0.09]	87.1 (0.3)	82	27	9	
Carabidae (Col.)	-0.060 ± 0.15 [-0.35; 0.23]	11.9 (0.89)	20	18	5	10(675), 5(629), 3(649), 1(676, 678)
Chrysomelidae (Col.)	-0.283 ± 0.20 [-0.67; 0.10]	24.2 (0.15)	19	17	3	13(675), 5(629), 1(677)
Coccinellidae (Col.)	0.390 ± 0.20 [-0.01; 0.79]	9.2 (0.42)	10	8	4	5(629), 3(648), 1(676, 677)
Staphylinidae (Col.)	-0.165 ± 0.19 [-0.53; 0.20]	13 (0.52)	15	13	5	8(675), 4(629), 1(524, 676, 678)
**Diptera**	**-0.298 ± 0.13 [-0.55; -0.05]**	33.3 (0.4)	33	17	4	
Syrphidae (Dip.)	-0.170 ± 0.21 [-0.59; 0.25]	12.9 (0.23)	11	9	3	5(629), 4(675), 2(648)
**Hemiptera**	-0.055 ± 0.07 [-0.20; 0.09]	69.3 (0.96)	92	25	6	
Anthocoridae (Hem.)	0.093 ± 0.15 [-0.20; 0.38]	13.7 (0.88)	22	20	5	12(675), 5(629), 3(648), 1(676, 677)
Aphididae (Hem.)	-0.009 ± 0.18 [-0.37; 0.35]	13.4 (0.5)	15	13	4	7(675), 5(629), 2(648), 1(677)
Cicadellidae (Hem.)	-0.135 ± 0.18 [-0.48; 0.21]	16.8 (0.47)	18	18	4	13(675), 3(648), 1(676, 677)
Miridae (Hem.)	-0.177 ± 0.17 [-0.51; 0.15]	9.2 (0.87)	16	14	4	7(675), 5(629), 3(648), 1(676)
**Hymenoptera**	0.456 ± 0.29 [-0.10; 1.02]	9.6 (0.14)	7	6	4	
Neuroptera	0.101 ± 0.14 [-0.18; 0.38]	22.2 (0.51)	24	22	5	15(675), 5(629), 2(648), 1(677, 678)
Orthoptera	-0.178 ± 0.28 [-0.72; 0.36]	1.8 (0.87)	6	6	3	4(675), 1(676, 678)
Thysanoptera	-0.032 ± 0.15 [-0.32; 0.26]	24.4 (0.27)	22	20	6	11(675), 5(629), 3(648), 1(676, 677, 678)

Table S7.3: Moderator analyses of Bt proteins for main meta-analyses. Records with any "red" critical appraisal label were excluded. Given is the analyzed taxon and Bt protein, the estimated effect size (estimate) with standard error (SE) and 95% confidence interval (lower boundary; upper boundary), and the number of records (rec), experiments (exp) and articles (art) included in the respective analysis. Significant effect sizes (confidence intervals do not include zero) are marked in bold.

Taxon	Estimate ± SE [ci.lb; ci.ub]	Rec	Exp	Art
**all Taxa** - Cry1A.105&Cry2Ab&Cry3Bb	**-0.101 ± 0.05 [-0.20; -0.004]**	210	22	5
Cry1Ab	-0.030 ± 0.02 [-0.08; 0.02]	887	130	69
Cry1Ab&mCry3A	0.0001 ± 0.09 [-0.18; 0.18]	43	3	3
Cry1Ac	**0.211 ± 0.10 [0.01; 0.41]**	62	7	4
Cry3Bb	-0.052 ± 0.04 [-0.13; 0.03]	280	30	21
Nematoda – Cry1Ab	-0.291 ± 0.17 [-0.62; 0.03]	9	9	3
Cry3Bb	0.017 ± 0.28 [-0.54; 0.57]	6	6	4
Oligochaeta – Cry1Ab	0.110 ± 0.23 [-0.33; 0.55]	9	6	5
Acarina – Cry1Ab	0.096 ± 0.22 [-0.33; 0.52]	11	11	6
Cry3Bb	0.304 ± 0.21 [-0.10; 0.71]	15	13	4
Araneae – Cry1Ab	0.050 ± 0.08 [-0.10; 0.20]	89	61	23
Cry1Ab & mCry3A	-0.023 ± 0.27 [-0.55; 0.51]	5	3	3
Cry1Ac	0.117 ± 0.32 [-0.51; 0.74]	6	6	3
Cry3Bb	-0.097 ± 0.14 [-0.37; 0.18]	24	17	8
Opiliones – Cry1Ab	0.449 ± 0.43 [-0.39; 1.29]	5	5	3
Myriapoda – Cry1Ab	-0.0001 ± 0.33 [-0.64; 0.64]	7	7	4
Collembola – Cry1Ab	0.087 ± 0.15 [-0.20; 0.37]	21	19	10
Cry3Bb	-0.045 ± 0.16 [-0.36; 0.27]	19	17	7
**Coleoptera** - Cry1A.105&Cry2Ab&Cry3Bb	-0.034 ± 0.10 [-0.24; .017]	46	20	3
Cry1Ab	0.011 ± 0.05 [-0.09; 0.11]	214	85	42
Cry1Ab&mCry3A	0.038 ± 0.19 [-0.33; 0.40]	11	3	3
Cry1Ac	0.162 ± 0.22 [-0.26; 0.59]	14	7	3
Cry3Bb	-0.135 ± 0.08 [-0.28; 0.01]	82	23	13
Cantharidae (Col.) – Cry1Ab	0.501 ± 0.32 [-0.12; 1.13]	6	6	3
Carabidae (Col.) – Cry1Ab	0.008 ± 0.10 [-0.19; 0.20]	48	48	21
Cry3Bb	-0.062 ± 0.15 [-0.35; 0.22]	21	20	7
Chrysomelidae (Col.) – Cry1Ab	0.155 ± 0.24 [-0.32; 0.63]	11	11	5
Cry3Bb	-0.416 ± 0.34 [-1.09; 0.26]	5	5	3
Coccinellidae (Col.) – Cry1Ab	0.035 ± 0.09 [-0.13; 0.20]	70	59	22
Cry3Bb	-0.173 ± 0.16 [-0.49; 0.14]	19	17	5
Elateridae (Col.) – Cry1Ab	-0.368 ± 0.36 [-1.08; 0.34]	6	6	3

Nitidulidae (Col.) – Cry1Ab	-0.460 ± 0.24 [-0.93; 0.01]	9	9	4
Staphylinidae (Col.) – Cry1Ab	-0.125 ± 0.13 [-0.39; 0.14]	29	29	13
Cry3Bb	-0.254 ± 0.19 [-0.62; 0.11]	15	14	5
**Diptera** – Cry1Ab	**-0.328 ± 0.08 [-0.49; -0.17]**	71	38	14
Cry3Bb	0.026 ± 0.11 [-0.20; 0.25]	26	10	5
Syrphidae (Dip.) – Cry1Ab	-0.215 ± 0.12 [-0.44; 0.01]	37	25	8
Tachinidae (Dip.) – Cry1Ab	**-0.850 ± 0.30 [-1.44; -0.26]**	5	5	4
**Hemiptera** – Cry1Ab	0.055 ± 0.05 [-0.04; 0.15]	200	83	32
Cry1Ab&mCry3A	0.087 ± 0.21 [-0.33; 0.51]	9	3	3
Cry3Bb	0.007 ± 0.09 [-0.17; 0.19]	50	21	7
Anthocoridae (Hem.) – Cry1Ab	**0.188 ± 0.09 [0.01; 0.37]**	61	51	20
Cry3Bb	0.021 ± 0.14 [-0.26; 0.30]	26	21	6
Aphididae (Hem.) – Cry1Ab	-0.064 ± 0.09 [-0.25; 0.12]	53	43	19
Cry3Bb	0.054 ± 0.20 [-0.34; 0.45]	9	9	3
Cicadellidae (Hem.) – Cry1Ab	0.135 ± 0.14 [-0.14; 0.41]	26	23	11
Cry3Bb	0.172 ± 0.26 [-0.33; 0.68]	5	5	3
Nabidae (Hem.) – Cry1Ab	-0.194 ± 0.18 [-0.55; 0.16]	14	14	7
**Hymenoptera** – Cry1Ab	**-0.234 ± 0.07 [-0.37; -0.10]**	111	58	22
Cry3Bb	-0.178 ± 0.15 [-0.47; 0.11]	20	14	7
Braconidae (Hym.) – Cry1Ab	**-1.705 ± 0.25 [-2.19; -1.22]**	24	15	3
Formicidae (Hym.) – Cry1Ab	0.115 ± 0.28 [-0.43; 0.66]	8	8	4
Cry3Bb	-0.349 ± 0.23 [-0.80; 0.10]	10	9	3
Neuroptera – Cry1Ab	0.037 ± 0.09 [-0.14; 0.21]	62	50	20
Cry3Bb	-0.111 ± 0.21 [-0.53; 0.30]	8	8	3
Orthoptera – Cry1Ab	0.088 ± 0.25 [-0.41; 0.58]	10	10	3
Cry3Bb	-0.093 ± 0.25 [-0.58; 0.40]	9	9	4
Thysanoptera – Cry1Ab	0.167 ± 0.15 [-0.12; 0.45]	21	21	11
Cry3Bb	0.164 ± 0.22 [-0.27; 0.60]	7	7	3

Table S7.4: Main meta-analyses with all Bt proteins and either no records excluded based on critical appraisal, or only records with all "green" critical appraisal labels included. Given is the analyzed taxon, the estimated effect size (estimate) with standard error (SE) and 95% confidence interval (lower boundary; upper boundary), the measure of heterogenetiy (Q value and significance, p<0.05 is significant), the number of records (rec), experiments (exp) and articles (art) included in the respective analysis, and the number of records per article (articleID in parenthesis). Significant heterogeneity and significant effect sizes (confidence intervals do not include zero) are marked in bold.

Taxon	Estimate ± SE [ci.lb; ci.ub]	Q (p)	Rec	Exp	Art	Records per article
**No records excluded**						
**all Taxa**	**-0.030 ± 0.02 [-0.06; 0.0003]**	2058.7 (0.99)	2220	226	116	358(675), 158(629), 126(173), 96(659), 87(169), 59(88), 50(600), 48(516), 47(603), 46(647), 42(527, 611), 41(224, 225), 39(637), 36(626), 35(652), 32(507), 28(182), 27(166), 26(170), 25(167, 208), 24(674), 22(52, 671), 21(90, 230, 648), 20(631, 632), 19(678), 18(630, 673), 17(670), 16(175, 601), 15(604), 14(38, 676), 13(655), 12(620, 650, 658, 662), 11(75, 614), 10(144, 211, 612, 677), 9(10, 28, 213, 605, 672), 8(228, 504, 621, 646, 668), 7(624), 6(4, 31, 214, 246, 514, 519, 521, 627, 628, 639, 640, 657, 660, 667), 5(613, 625), 4(171, 602, 615, 663), 3(219, 244, 606, 633, 634, 638, 649, 654), 2(86, 524, 608, 616, 617, 622, 635, 642, 653, 656, 666), 1(83, 215, 508, 609, 618, 619, 636, 641, 643, 644, 645, 651, 665, 669)
Nematoda	-0.239 ± 0.13 [-0.49; 0.01]	18.4 (0.37)	18	18	9	6(660), 2(608, 635, 653, 656), 1(90, 619, 625, 645)
Oligochaeta	0.039 ± 0.18 [-0.32; 0.40]	6.2 (0.96)	15	10	7	6(628), 2(144, 225, 674), 1(508, 624, 636)
Acarina	0.045 ± 0.10 [-0.16; 0.25]	31.7 (0.98)	51	43	20	11(90), 8(675), 4(504), 3(527, 657), 2(144, 175, 611, 637, 670, 671, 674), 1(28, 208, 603, 609, 622, 624, 633, 678)
Araneae	0.020 ± 0.05 [-0.08; 0.12]	171.8 (0.98)	212	127	46	33(675), 21(173), 20(629), 8(169, 507, 659), 6(88, 182), 5(224, 225, 614), 4(170, 516, 601, 611, 671), 3(166, 167, 208, 244, 519, 527, 600, 605, 606, 637, 639, 647, 662, 672), 2(52, 175, 603, 626, 646, 673, 674, 676, 678), 1(28, 75, 215, 228, 230, 620, 677)
Opiliones	0.526 ± 0.33 [-0.13; 1.18]	5.2 (0.52)	7	7	4	3(519), 2(611), 1(516, 603)
Myriapoda	-0.040 ± 0.17 [-0.38; 0.30]	9.1 (0.96)	19	19	10	3(166, 514, 527), 2(516, 611, 674), 1(603, 624, 625, 678)
Collembola	-0.067 ± 0.08 [-0.21; 0.08]	50.9 (1)	92	65	26	29(675), 10(629), 9(90), 4(171, 225, 504), 3(527, 657), 2(88, 170, 175, 213, 516, 611, 673, 674), 1(208, 603, 622, 624, 625, 644, 647, 651, 665, 678)
**Coleoptera**	-0.021 ± 0.03 [-0.08; 0.04]	542.6 (0.71)	563	172	78	
Anthicidae (Col.)	-0.012 ± 0.21 [-0.43; 0.40]	13 (0.3)	12	9	4	6(675), 4(169), 1(659, 676)
Cantharidae (Col.)	0.501 ± 0.32 [-0.12; 1.13]	2.9 (0.72)	6	6	3	3(224), 2(516), 1(603)
Carabidae (Col.)	-0.004 ± 0.06 [-0.13; 0.12]	78.3 (1)	121	99	40	22(675), 10(629), 6(88, 640), 4(169, 224, 507, 615), 3(166, 219, 527, 605, 610, 634, 639, 649, 654, 672), 2(170, 175, 516, 611, 612, 614, 616, 642, 673), 1(28, 228, 603, 613, 620, 625, 643, 646, 659, 669, 671, 676, 678)
Chrysomelidae (Col.)	-0.150 ± 0.10 [-0.35; 0.05]	67.8 (0.16)	58	40	15	24(675), 10(629), 4(169), 3(167), 2(52, 516, 626, 666, 670, 673), 1(600, 620, 625, 647, 677)

Cicindelidae (Col.)	0.198 ± 0.29 [-0.36; 0.76]	5.3 (0.51)	7	6	3	4(169), 2(507), 1(88)
Coccinellidae (Col.)	0.088 ± 0.06 [-0.03; 0.21]	119.7 (0.88)	140	115	42	21(173), 10(629), 8(88, 659), 7(652), 5(182), 4(169, 224, 507, 601, 627), 3(75, 167, 527, 604, 637, 647, 648, 662), 2(10, 31, 38, 52, 170, 211, 246, 516, 600, 611, 621, 626, 671, 673), 1(4, 28, 228, 230, 603, 620, 646, 676, 677)
Elateridae (Col.)	-0.033 ± 0.15 [-0.32; 0.26]	29.1 (0.31)	27	19	8	10(629), 6(88), 4(169), 2(175, 516), 1(603, 637, 676)
Lathrididae (Col.)	0.797 ± 0.77 [-0.72; 2.31]	**39.1 (<0.0001)**	8	8	4	3(213, 647), 1(144, 603)
Nitidulidae (Col.)	-0.146 ± 0.15 [-0.43; 0.14]	31.6 (0.14)	25	25	7	11(659), 4(169), 3(166), 2(86, 170, 516), 1(230)
Scarabaeidae (Col.)	-0.277 ± 0.23 [-0.72; 0.17]	14.9 (0.19)	12	11	5	4(169), 3(166), 2(516, 675), 1(88)
Staphylinidae (Col.)	**-0.171 ± 0.08 [-0.32; -0.02]**	79.2 (0.71)	88	67	27	21(675), 8(629), 6(88), 4(169, 224, 507, 663), 3(166, 521, 527, 605, 655, 672), 2(170, 175, 516, 524, 611), 1(28, 182, 228, 230, 603, 646, 671, 676, 678)
Dermaptera	-0.120 ± 0.13 [-0.37; 0.13]	27.6 (0.73)	34	24	8	14(675), 10(659), 4(507), 2(674), 1(208, 213, 646, 676)
**Diptera**	**-0.179 ± 0.05 [-0.28; -0.08]**	223.1 (0.06)	193	91	33	
Chironomidae (Dip.)	-0.065 ± 0.21 [-0.47; 0.34]	4.8 (0.58)	7	7	3	3(647, 655), 1(600)
Chloropidae (Dip.)	-0.346 ± 0.43 [-1.19; 0.50]	**12.9 (0.02)**	6	6	3	
Dolichopodidae (Dip.)	0.088 ± 0.23 [-0.36; 0.54]	6.6 (0.68)	10	8	5	4(675), 3(527), 1(603, 655, 659)
Otitidae (Dip.)	-0.236 ± 0.16 [-0.55; 0.08]	**43 (0.046)**	30	21	3	20(675), 6(659), 4(169)
Syrphidae (Dip.)	**-0.188 ± 0.08 [-0.35; -0.03]**	85.6 (0.11)	72	51	19	21(173), 10(629), 7(652), 6(675), 3(167, 637, 650), 2(38, 211, 600, 611, 626, 648, 671), 1(28, 230, 601, 603, 659)
Tachinidae (Dip.)	**-0.492 ± 0.17 [-0.83; -0.15]**	21.5 (0.09)	15	11	5	10(675), 2(617), 1(38, 230, 618)
**Hemiptera**	0.014 ± 0.03 [-0.05; 0.08]	427.9 (1)	510	165	57	
Anthocoridae (Hem.)	0.109 ± 0.06 [-0.01; 0.23]	159.6 (0.21)	147	114	37	24(675), 21(173), 14(88), 10(629, 659), 6(182), 4(169, 224), 3(31, 75, 167, 527, 604, 637, 648), 2(10, 170, 211, 213, 246, 516, 621, 626, 671), 1(4, 28, 38, 83, 228, 230, 600, 603, 646, 673, 676, 677)
Aphididae (Hem.)	-0.030 ± 0.07 [-0.16; 0.10]	83.6 (0.91)	103	83	33	10(629, 675), 7(652), 6(225, 630, 667), 4(659), 3(167, 214, 521, 527, 600, 604, 637, 647, 662), 2(38, 52, 516, 611, 612, 626, 632, 648, 670, 673), 1(75, 228, 230, 603, 613, 620, 677)
Cicadellidae (Hem.)	0.011 ± 0.08 [-0.14; 0.16]	60.1 (0.98)	85	71	24	24(675), 11(659), 6(225), 4(169), 3(208, 214, 527, 600, 647, 648, 658), 2(52, 516, 611, 626, 632, 670), 1(144, 230, 603, 620, 673, 676, 677)
Delphacidae (Hem.)	-0.118 ± 0.25 [-0.61; 0.37]	4.8 (0.85)	10	10	3	4(632), 3(658, 659)
Geocoridae (Hem.)	0.126 ± 0.23 [-0.32; 0.57]	10.8 (0.37)	11	9	3	6(675), 4(169), 1(659)
Miridae (Hem.)	-0.048 ± 0.11 [-0.26; 0.17]	26 (0.91)	38	24	9	14(675), 10(629), 3(647, 648), 2(600, 626, 670), 1(230, 676)
Nabidae (Hem.)	-0.117 ± 0.12 [-0.35; 0.11]	18.3 (0.99)	36	29	12	10(629), 6(182), 4(169), 3(167), 2(10, 75, 211, 600, 621), 1(603, 646, 677)
Pentatomidae (Hem.)	0.142 ± 0.20 [-0.26; 0.54]	4.4 (0.99)	14	12	6	4(675), 3(169, 662), 2(670), 1(230, 516)
**Hymenoptera**	**-0.145 ± 0.05 [-0.24; -0.04]**	**250.5 (0.004)**	195	109	45	
Braconidae (Hym.)	**-1.286 ± 0.23 [-1.74; -0.83]**	**85.5 (<0.0001)**	33	24	7	21(173), 3(167, 637), 2(516, 611), 1(230, 671)
Formicidae (Hym.)	-0.022 ± 0.14 [-0.30; 0.26]	17.8 (0.77)	24	22	11	5(88), 4(169), 3(166), 2(175, 516, 600, 675), 1(601, 603, 677, 678)

Ichneumonidae (Hym.)	-0.060 ± 0.31 [-0.67; 0.55]	4.6 (0.47)	6	5	4	2(611, 627), 1(601, 603)
Mymaridae (Hym.)	-0.214 ± 0.38 [-0.95; 0.52]	6.3 (0.28)	6	6	3	3(658), 2(611), 1(603)
Vespidae (Hym.)	0.447 ± 0.25 [-0.04; 0.94]	8.4 (0.21)	7	5	3	4(675), 2(600), 1(603)
Mecoptera	-0.325 ± 0.25 [-0.81; 0.16]	3.3 (0.66)	6	6	3	3(637), 2(600), 1(603)
Neuroptera	0.071 ± 0.06 [-0.05; 0.19]	132.7 (0.42)	131	101	36	26(675), 21(173), 10(629), 7(652, 659), 4(182), 3(224, 527, 604, 637, 647, 650), 2(10, 38, 144, 170, 211, 516, 600, 601, 611, 621, 626, 648, 671, 673), 1(4, 28, 75, 167, 208, 230, 603, 677, 678)
Orthoptera	-0.067 ± 0.13 [-0.32; 0.19]	21.6 (0.94)	34	28	11	10(675), 5(632), 4(169), 3(88, 166), 2(175, 516, 626), 1(674, 676, 678)
Psocoptera	0.529 ± 0.31 [-0.07; 1.13]	6.8 (0.24)	6	6	4	2(600, 674), 1(230, 678)
Thysanoptera	0.070 ± 0.08 [-0.08; 0.22]	55 (0.97)	77	61	25	20(675), 10(629), 4(169), 3(208, 527, 600, 604, 637, 647, 648), 2(38, 52, 611, 612, 614, 626, 670), 1(228, 230, 603, 613, 620, 676, 677, 678)
**Only records with all “green” critical appraisal labels included**						
**all Taxa**	-0.025 ± 0.04 [-0.10; 0.05]	**505.2 (<0.0001)**	371	53	26	120(173), 46(603), 31(626), 29(652), 18(230), 14(604), 12(662), 11(600), 10(38, 637, 677), 9(605), 8(648), 7(228), 6(639), 5(614, 668), 3(606, 610, 654, 655), 2(31, 642, 647), 1(215, 669)
Araneae	0.088 ± 0.10 [-0.10; 0.28]	28.1 (0.98)	47	33	14	20(173), 3(605, 606, 614, 639, 662), 2(600, 603, 626, 637), 1(215, 228, 230, 677)
**Coleoptera**	0.081 ± 0.08 [-0.07; 0.23]	83.2 (0.32)	79	43	22	
Carabidae (Col.)	-0.048 ± 0.14 [-0.31; 0.22]	10.4 (0.92)	19	19	9	3(605, 610, 639, 654), 2(614, 642), 1(228, 603, 669)
Coccinellidae (Col.)	**0.256 ± 0.12 [0.02; 0.49]**	43.8 (0.21)	38	29	13	20(173), 3(662), 2(38, 604, 626, 648), 1(31, 230, 600, 603, 637, 652, 677)
Staphylinidae (Col.)	-0.126 ± 0.25 [-0.62; 0.36]	13.3 (0.1)	9	9	5	3(605, 655), 1(228, 230, 603)
**Diptera**	**-0.358 ± 0.12 [-0.59; -0.13]**	**78 (0.01)**	51	22	7	
Syrphidae (Dip.)	**-0.294 ± 0.13 [-0.56; -0.03]**	**51.7 (0.02)**	33	21	7	20(173), 7(652), 2(648), 1(230, 603, 626, 637)
**Hemiptera**	0.136 ± 0.08 [-0.02; 0.29]	97.8 (0.07)	80	38	15	
Anthocoridae (Hem.)	0.174 ± 0.16 [-0.14; 0.49]	**52.8 (0.01)**	33	24	10	20(173), 3(604), 2(626, 637), 1(31, 38, 228, 230, 603, 677)
Aphididae (Hem.)	-0.036 ± 0.13 [-0.30; 0.23]	21.3 (0.5)	23	20	11	7(652), 3(604, 662), 2(600, 626), 1(38, 228, 230, 603, 637, 677)
Cicadellidae (Hem.)	0.210 ± 0.20 [-0.18; 0.60]	10.8 (0.37)	11	11	7	2(600, 626, 647, 648), 1(230, 603, 677)
**Hymenoptera**	**-0.747 ± 0.16 [-1.07; -0.42]**	**104.5 (<0.0001)**	47	22	7	
Neuroptera	0.018 ± 0.11 [-0.19; 0.23]	43 (0.27)	39	27	10	20(173), 7(652), 3(604), 2(38, 648), 1(228, 603, 626, 637, 677)
Thysanoptera	0.156 ± 0.17 [-0.18; 0.49]	15.6 (0.34)	15	15	9	3(604), 2(38, 600, 626, 637), 1(228, 230, 603, 677)

Table S7.5: Higher level analyses with functional groups and either all Bt proteins or Lepidoptera-targeted, Coleoptera-targeted, or stacked Bt proteins separately. Records with any "red" critical appraisal label excluded. Given is the analyzed taxon, the estimated effect size (estimate) with standard error (SE) and 95% confidence interval (lower boundary; upper boundary), the measure of heterogenetiy (Q value and significance, p<0.05 is significant), and the number of records (rec), experiments (exp) and articles (art) included in the respective analysis. Significant heterogeneity and significant effect sizes (confidence intervals do not include zero) are marked in bold.

	Estimate ± SE [ci.lb; ci.ub]	Q (p)	Rec	Exp	Art
**All Bt proteins**					
Decomposers	-0.022 ± 0.06 [-0.14; 0.10]	123.8 (0.8)	139	73	31
Herbivores	-0.034 ± 0.03 [-0.10; 0.03]	459 (0.86)	494	131	48
Omnivores	0.035 ± 0.20 [-0.36; 0.43]	4.7 (0.79)	9	9	5
Parasitoids	**-0.230 ± 0.06 [-0.35; -0.11]**	**194 (0.0005)**	135	86	35
Predators	0.012 ± 0.02 [-0.03; 0.05]	921.7 (1)	1064	184	78
**Lepidoptera-targeted Bt proteins**					
Decomposers	0.030 ± 0.08 [-0.13; 0.19]	92.4 (0.27)	86	51	23
Herbivores	-0.006 ± 0.04 [-0.09; 0.08]	225 (0.97)	267	88	32
Parasitoids	**-0.342 ± 0.08 [-0.50; -0.19]**	**172.1 (<0.0001)**	106	66	27
Predators	0.027 ± 0.03 [-0.03; 0.08]	590.5 (0.99)	674	135	58
**Coleoptera-targeted Bt proteins**					
Decomposers	-0.075 ± 0.13 [-0.32; 0.17]	18.2 (0.96)	31	25	10
Herbivores	0.039 ± 0.07 [-0.09; 0.17]	104 (0.54)	107	35	13
Parasitoids	0.076 ± 0.15 [-0.22; 0.37]	6.3 (0.99)	19	16	6
Predators	-0.042 ± 0.05 [-0.13; 0.05]	186.7 (0.95)	222	41	17
**Lepidoptera- and Coleoptera-targeted stacks**					
Decomposers	-0.122 ± 0.15 [-0.41; 0.17]	12.2 (0.93)	22	18	3
Herbivores	**-0.167 ± 0.07 [-0.30; -0.04]**	124.1 (0.36)	120	28	7
Parasitoids	-0.064 ± 0.21 [-0.48; 0.36]	9.2 (0.42)	10	8	3
Predators	0.026 ± 0.05 [-0.08; 0.13]	142.8 (0.91)	168	28	9

Table S7.6: Moderator analyses of Bt proteins for functional groups. Records with any "red" critical appraisal label were excluded. Given is the analyzed group and Bt protein, the estimated effect size (estimate) with standard error (SE) and 95% confidence interval (lower boundary; upper boundary), the measure of heterogenetiy (Q value and significance, p<0.05 is significant), the number of records (rec), experiments (exp) and articles (art) included in the respective analysis, and the number of records per article (articleID in parenthesis). Significant heterogeneity and significant analyses (confidence intervals do not include zero) are marked in bold.

	**Estimate ± SE [ci.lb; ci.ub]**	**Rec**	**Exp**	**Art**
**Decomposers**				
Cry1Ab	0.042 ± 0.10 [-0.16; 0.25]	47	26	15
Cry1Ac	0.230 ± 0.27 [-0.30; 0.76]	10	4	3
Cry3Bb	-0.073 ± 0.13 [-0.33; 0.19]	28	22	9
**Herbivores**				
Cry1Ab	0.008 ± 0.05 [-0.10; 0.11]	172	61	25
Cry3Bb	0.002 ± 0.09 [-0.17; 0.18]	49	18	11
**Parasitoids**				
Cry1Ab	**-0.343 ± 0.08 [-0.49; -0.19]**	93	55	23
Cry3Bb	-0.004 ± 0.18 [-0.35; 0.35]	12	9	5
**Predators**				
Cry1A.105&Cry2Ab&Cry3Bb	-0.030 ± 0.07 [-0.17; 0.11]	99	22	4
Cry1Ab	0.024 ± 0.03 [-0.04; 0.09]	506	107	50
Cry1Ab&mCry3A	0.041 ± 0.13 [-0.20; 0.29]	24	3	3
Cry1Ac	0.267 ± 0.19 [-0.10; 0.63]	19	6	3
Cry3Bb	-0.097 ± 0.06 [-0.21; 0.01]	147	24	14

Table S7.7: Higher level analyses for private sector contribution and all Bt proteins, or Lepidoptera-targeted, Coleoptera-targeted, or stacked Bt proteins separately. Records with any "red" critical appraisal label were excluded. Given is the analyzed group, the estimated effect size (estimate) with standard error (SE) and 95% confidence interval (lower boundary; upper boundary), the measure of heterogenetiy (Q value and significance, p<0.05 is significant), and the number of records (rec), experiments (exp) and articles (art) included in the respective analysis. Significant heterogeneity and significant effect sizes (confidence intervals do not include zero) are marked in bold.

	**Estimate ± SE [ci.lb; ci.ub]**	**Q (p)**	**Rec**	**Exp**	**Art**
**All Bt proteins**					
Private	**-0.085 ± 0.03 [-0.13; -0.04]**	862.3 (0.6)	874	76	22
Public	0.008 ± 0.02 [-0.03; 0.05]	952.5 (1)	1102	141	85
**Lepidoptera-targeted Bt proteins**					
Private	**-0.104 ± 0.04 [-0.17; -0.04]**	507.2 (0.13)	474	47	12
Public	0.018 ± 0.03 [-0.03; 0.07]	650.9 (0.99)	746	113	67
**Coleoptera-targeted Bt proteins**					
Private	0.016 ± 0.06 [-0.09; 0.12]	141.8 (0.95)	172	21	7
Public	-0.025 ± 0.04 [-0.11; 0.06]	201.2 (0.99)	251	27	17
**Lepidoptera- and Coleoptera-targeted stacks**					
Private	**-0.122 ± 0.05 [-0.22; -0.03]**	209.1 (0.8)	228	23	5
Public	0.024 ± 0.06 [-0.10; 0.15]	99.5 (0.61)	105	8	6

Table S7.8: Moderator analyses of different Bt proteins for private sector contribution. Records with any “red” critical appraisal label were excluded. Given is the analyzed group and Bt protein, the estimated effect size (estimate) with standard error (SE) and 95% confidence interval (lower boundary; upper boundary), and the number of records (rec), experiments (exp) and articles (art) included in the respective analysis. Significant effect sizes (confidence intervals do not include zero) are marked in bold.

	**Estimate ± SE [ci.lb; ci.ub]**	**Rec**	**Exp**	**Art**
**Private**				
Cry1Ab	**-0.129 ± 0.05 [-0.23; -0.03]**	237	28	9
Cry1Ab&mCry3A	0.0001 ± 0.09 [-0.18; 0.18]	43	3	3
Cry3Bb	-0.116 ± 0.08 [-0.28; 0.05]	76	7	6
**Public**				
Cry1A.105&Cry2Ab&Cry3Bb	0.120 ± 0.10 [-0.08; 0.32]	25	4	3
Cry1Ab	-0.002 ± 0.03 [-0.05; 0.05]	650	102	60
Cry1Ac	**0.211 ± 0.10 [0.01; 0.41]**	62	7	4
Cry3Bb	-0.033 ± 0.05 [-0.12; 0.06]	204	24	15

Table S7.9: Subgroup analyses on lower taxonomic units and species for all Bt proteins and records with any "red" critical appraisal label excluded. Given is the analyzed taxon, the estimated effect size (estimate) with standard error (SE) and 95% confidence interval (lower boundary; upper boundary), the measure of heterogenetiy (Q value and significance, p<0.05 is significant), the number of records (rec), experiments (exp) and articles (art) included in the respective analysis, and the number of records per article (articleID in parenthesis). Significant heterogeneity and significant effect sizes (confidence intervals do not include zero) are marked in bold.

Taxon	Estimate ± SE [ci.lb; ci.ub]	Q (p)	Rec	Exp	Art	Records per article
Aca: Oribatidae	0.148 ± 0.16 [-0.16; 0.46]	23.7 (0.42)	24	22	6	11(90), 4(504), 3(527, 657), 2(175), 1(678)
Mesostigmata	0.052 ± 0.21 [-0.36; 0.46]	15.5 (0.27)	14	12	3	11(90), 2(175), 1(678)
Ara: Linyphiidae: *Bathyphantes gracilis*	-0.034 ± 0.37 [-0.77; 0.7]	8.6 (0.13)	6	6	3	2(519, 605, 639)
Erigone atra	-0.136 ± 0.36 [-0.83; 0.56]	6.6 (0.16)	5	5	3	3(639), 1(228, 519)
Oedothorax apicatus	-0.225 ± 0.18 [-0.57; 0.12]	3.9 (0.98)	13	13	5	3(519, 505, 606, 639), 1(228)
Porrhomma microphthalmum	-0.125 ± 0.23 [-0.57; 0.32]	1.2 (0.95)	6	6	3	3(639), 2(605), 1(228)
Ara: Lycosidae: *Pardosa agrestis*	0.276 ± 0.23 [-0.17; 0.72]	6.4 (0.7)	10	10	4	3(519, 605, 606), 1(228)
Myriapoda: Chilopoda	0.078 ± 0.22 [-0.36; 0.52]	4 (0.95)	11	11	6	3(166, 514), 2(516), 1(603, 674, 678)
Col: Carabidae: *Anchomenus dorsalis*	0.038 ± 0.19 [-0.34; 0.42]	2.6 (0.96)	9	9	5	3(610), 2(612, 640), 1(639, 669)
Bembidion lampros	0.135 ± 0.17 [-0.21; 0.48]	3.9 (0.92)	10	10	5	3(639, 640), 2(610), 1(228, 669)
Bembidion obtusum	0.388 ± 0.24 [-0.08; 0.85]	2.6 (0.62)	5	5	3	3(639), 1(603, 669)
Bembidion quadrimaculatum	0.054 ± 0.15 [-0.23; 0.34]	19.3 (0.37)	19	19	8	5(640), 3(605, 615, 639), 2(610), 1(228, 642, 669)
Calathus fuscipes	0.097 ± 0.14 [-0.18; 0.38]	13.3 (0.65)	17	17	8	3(605, 610, 639, 640), 2(654), 1(228, 612, 669)
Harpalus affinis	-0.080 ± 0.17 [-0.41; 0.25]	5.6 (0.93)	13	13	7	3(639, 640), 2(610, 612), 1(228, 603, 669)
Harpalus distinguendus	-0.020 ± 0.23 [-0.48; 0.44]	0.2 (1)	6	6	3	3(219), 2(610), 1(603)
Harpalus rufipes	-0.013 ± 0.13 [-0.27; 0.24]	22.3 (0.5)	24	24	11	6(640), 3(219, 605, 639), 2(610, 612), 1(228, 603, 620, 643, 669)
Poecilus cupreus	-0.045 ± 0.16 [-0.35; 0.26]	10.6 (0.83)	17	17	9	3(605, 639, 654), 2(612, 640), 1(228, 603, 610, 620)
Pterostichus melanarius	-0.057 ± 0.12 [-0.29; 0.17]	16.9 (0.96)	30	30	12	6(640), 4(615), 3(605, 610, 639, 654), 2(612, 642), 1(228, 603, 643, 669)
Trechus quadristriatus	-0.101 ± 0.19 [-0.47; 0.27]	23 (0.06)	15	15	7	3(219, 639, 640), 2(610, 654), 1(228, 669)
Col: Chrysomelidae: Alticini	-0.169 ± 0.13 [-0.42; 0.08]	53.9 (0.09)	42	26	6	24(675), 8(629), 4(169), 3(167), 2(666), 1(677)
Diabrotica spp.	-0.280 ± 0.21 [-0.69; 0.13]	11.2 (0.51)	13	13	4	7(675), 2(169, 516, 629)
Col: Coccinellidae: *Coccinella septempunctata*	0.031 ± 0.28 [-0.51; 0.57]	3 (0.7)	6	6	5	2(38), 1(169, 626, 648, 677)
Coleomegilla maculata	0.188 ± 0.12 [-0.05; 0.43]	43.5 (0.21)	38	29	9	21(173), 4(169), 3(75, 88, 167), 1(10, 31, 246, 516)
Harmonia axyridis	**0.569 ± 0.27 [0.05; 1.09]**	3 (0.81)	7	7	5	2(167, 626), 1(31, 75, 648)
Hippodamia convergens	-0.018 ± 0.33 [-0.66; 0.62]	0.9 (0.97)	6	5	3	4(88), 1(169, 246)
Propylea quatuordecimpunctata	0.100 ± 0.18 [-0.25; 0.45]	11.4 (0.58)	14	7	4	10(629), 2(648), 1(230, 603)
Scymnus spp.	-0.283 ± 0.24 [-0.75; 0.19]	6.4 (0.6)	9	9	4	4(169), 2(246, 621), 1(230)

Col: Staphylinidae: *Aleochara bipustulata*	-0.130 ± 0.25 [-0.63; 0.37]	4.3 (0.5)	6	6	3	3(605), 2(521), 1(228)
Anotylus rugosus	0.187 ± 0.29 [-0.38; 0.75]	7.8 (0.26)	7	7	4	3(663), 2(655), 1(228, 605)
Hem: Anthocoridae: *Orius insidiosus*	0.018 ± 0.10 [-0.18; 0.21]	49.8 (0.77)	59	44	13	20(173), 14(88), 4(169), 3(31, 75, 167, 527), 2(10, 246, 516), 1(4, 38, 83)
Hem: Aphididae: *Metopolophium dirhodum*	0.084 ± 0.13 [-0.18; 0.35]	28 (0.14)	22	19	8	7(652), 3(214, 604), 2(38, 600, 637, 648), 1(228)
Rhopalosiphum maidis	-0.155 ± 0.33 [-0.8; 0.49]	2.5 (0.64)	5	5	3	3(167), 1(603, 677)
Rhopalosiphum padi	-0.178 ± 0.13 [-0.44; 0.08]	37.5 (0.11)	29	26	10	7(652), 6(667), 3(214, 521, 604), 2(600, 637), 1(38, 213, 228)
Sitobion avenae	0.111 ± 0.18 [-0.24; 0.46]	16.9 (0.26)	15	12	5	7(652), 3(214), 2(38, 600), 1(604)
Hem: Cicadellidae: *Zyginidia scutellaris*	-0.006 ± 0.15 [-0.3; 0.29]	8.1 (0.88)	15	15	7	3(214, 647, 648, 658), 1(229, 600, 603)
Hym: Braconidae: *Macrocentrus cingulum*	**-1.613 ± 0.23 [-2.06; -1.17]**	**53.8 (0.0007)**	26	17	3	21(173), 3(167, 516)
Neu: Chrysopidae	0.055 ± 0.07 [-0.08; 0.19]	116.3 (0.4)	114	84	29	26(675), 20(173), 10(629), 7(652, 659), 4(182), 3(224, 527, 604), 2(10, 38, 167, 170, 211, 600, 601, 621, 626, 637, 673), 1(4, 75, 228, 230, 516, 603, 648, 677, 678)
Neu: Chrysopidae: *Chrysoperla carnea*	0.025 ± 0.12 [-0.2; 0.26]	43.4 (0.16)	36	27	12	20(173), 3(604), 2(38, 167, 621), 1(75, 228, 230, 603, 648, 677, 678)
Neu: Hemerobiidae	0.003 ± 0.14 [-0.26; 0.27]	16.1 (0.91)	26	18	6	19(173), 2(144, 648), 1(211, 516, 603)
Thysanoptera: herbivores	-0.031 ± 0.10 [-0.22; 0.16]	31.6 (0.88)	43	25	7	20(675), 6(647, 648), 4(629), 3(213, 637), 1(677)
predators	0.226 ± 0.20 [-0.16; 0.61]	3.3 (0.91)	9	6	4	4(629), 3(647), 1(648, 676)

Table S7.10: Moderator analyses target order (Lepidoptera-active, Coleoptera-active, or stacked) of subgroup analyses on lower taxonomic units and species. Records with any “red” critical appraisal label were excluded. Given is the analyzed taxon, the estimated effect size (estimate) with standard error (SE) and 95% confidence interval (lower boundary; upper boundary), and the number of records (rec), experiments (exp) and articles (art) included in the respective analysis. Significant effect sizes (confidence intervals do not include zero) are marked in bold.

Taxon	Lepidoptera-active Estimate ± SE [ci.lb; ci.ub]	Rec	Exp	Art	Coleoptera-active Estimate ± SE [ci.lb; ci.ub]	Rec	Exp	Art	Lep.&Col. stacks Estimate ± SE [ci.lb; ci.ub]	Rec	Exp	Art
Aca: Oribatidae	-0.005 ± 0.25 [-0.49; 0.48]	10	10	3								
Ara: Linyphiidae: *Bathyphantes gracilis*	-0.034 ± 0.37 [-0.77; 0.70]	6	6	3								
Erigone atra	-0.136 ± 0.36 [-0.83; 0.56]	5	5	3								
Oedothorax apicatus	-0.224 ± 0.20 [-0.62; 0.17]	10	10	4								
Porrhomma microphthalmum	-0.125 ± 0.23 [-0.57; 0.32]	6	6	3								
Ara: Lycosidae: *Pardosa agrestis*	0.180 ± 0.28 [-0.37; 0.73]	7	7	3								
Myriapoda: Chilopoda	0.083 ± 0.31 [-0.53; 0.69]	7	7	4								
Col: Carabidae: *Anchomenus dorsalis*	0.223 ± 0.26 [-0.29; 0.73]	6	6	4								
Bembidion lampros	0.124 ± 0.20 [-0.27; 0.51]	8	8	4								
Bembidion obtusum	0.388 ± 0.24 [-0.08; 0.85]	5	5	3								
Bembidion quadrimaculatum	0.046 ± 0.17 [-0.28; 0.37]	17	17	7								
Calathus fuscipes	-0.012 ± 0.18 [-0.36; 0.33]	12	12	6								
Harpalus affinis	-0.099 ± 0.19 [-0.47; 0.27]	11	11	6								
Harpalus rufipes	-0.007 ± 0.15 [-0.29; 0.28]	22	22	10								
Poecilus cupreus	-0.139 ± 0.18 [-0.50; 0.22]	13	13	7								
Pterostichus melanarius	-0.105 ± 0.14 [-0.37; 0.17]	24	24	10								
Trechus quadristriatus	-0.286 ± 0.21 [-0.70; 0.12]	11	11	5								
Col: Chrysomelidae: Alticini	0.149 ± 0.19 [-0.22; 0.52]	16	15	3	**-0.647 ± 0.30 [-1.24; -0.06]**	7	7	3	-0.283 ± 0.18 [-0.64; 0.08]	19	17	3
Diabrotica spp.	-0.280 ± 0.21 [-0.69; 0.13]	13	13	4								
Coleomegilla maculata	0.232 ± 0.14 [-0.04; 0.50]	31	22	6	0.003 ± 0.29 [-0.56; 0.57]	7	7	3				
Scymnus spp.	-0.269 ± 0.27 [-0.80; 0.26]	7	7	3								
Col: Staphylinidae: *Aleochara bipustulata*	-0.130 ± 0.25 [-0.63; 0.37]	6	6	3								
Anotylus rugosus	-0.035 ± 0.33 [-0.68; 0.61]	5	5	3								
Hem: Anthocoridae: *Orius insidiosus*	0.051 ± 0.12 [-0.18; 0.28]	40	30	10	-0.061 ± 0.18 [-0.42; 0.30]	19	14	3				
Hem: Aphididae: *Metopolophium dirhodum*	0.018 ± 0.16 [-0.29; 0.32]	18	15	6								
Rhopalosiphum padi	-0.149 ± 0.14 [-0.42; 0.12]	27	24	9								
Sitobion avenae	0.111 ± 0.18 [-0.24; 0.46]	15	12	5								
Hem: Cicadellidae: *Zyginidia scutellaris*	-0.147 ± 0.23 [-0.59; 0.30]	9	9	5								
Neu: Chrysopidae	0.060 ± 0.08 [-0.10; 0.22]	77	65	23	0.050 ± 0.19 [-0.33; 0.43]	14	14	4	0.043 ± 0.15 [-0.25; 0.34]	23	21	5
Neu: Chrysopidae: *Chrysoperla carnea*	-0.036 ± 0.13 [-0.29; 0.22]	31	22	8								
Neu: Hemerobiidae	-0.013 ± 0.15 [-0.31; 0.29]	22	14	4								
Thysanoptera: herbivores	-0.223 ± 0.19 [-0.60; 0.15]	12	11	3	0.052 ± 0.17 [-0.28; 0.39]	11	8	4	0.019 ± 0.15 [-0.27; 0.31]	20	16	4

Table S7.11: Subgroup analyses on sampling methods and life stages (juveniles or eggs) for all Bt proteins and records with any "red" critical appraisal label excluded. Given is the analyzed taxon, the estimated effect size (estimate) with standard error (SE) and 95% confidence interval (lower boundary; upper boundary), the measure of heterogenetiy (Q value and significance, p<0.05 is significant), the number of records (rec), experiments (exp) and articles (art) included in the respective analysis, and the number of records per article (articleID in parenthesis). Significant heterogeneity and significant effect sizes (confidence intervals do not include zero) are marked in bold.

Taxon	Estimate ± SE [ci.lb; ci.ub]	Q (p)	Rec	Exp	Art	Records per article
Sampling methods						
Acarina - soil extraction	0.180 ± 0.16 [-0.13; 0.49]	12.5 (0.95)	23	21	6	11(90), 4(504), 3(657), 2(175, 674), 1(622)
pitfall trap	-0.237 ± 0.22 [-0.66; 0.19]	6.3 (0.85)	12	8	3	8(675), 3(527), 1(603)
litter extraction	0.189 ± 0.29 [-0.37; 0.75]	2.2 (0.83)	6	6	4	3(527), 1(624, 665, 678)
Araneae - pitfall trap	0.027 ± 0.08 [-0.13; 0.18]	40.8 (1)	85	64	22	22(675), 10(629), 6(88), 5(614), 4(169, 224), 3(166, 519, 527, 605, 606, 639, 672), 2(170, 175, 507, 516), 1(228, 603, 646, 676, 678)
visual counts	0.105 ± 0.09 [-0.07; 0.28]	48.3 (0.95)	67	51	21	10(629), 8(675), 7(659), 6(182, 225), 4(169, 601), 3(662), 2(170, 507, 516, 600, 626, 673), 1(75, 224, 230, 646, 676, 677, 678)
sticky trap	0.202 ± 0.13 [-0.05; 0.45]	29.3 (0.65)	34	24	7	21(173), 3(659, 675), 2(167, 170, 516), 1(230)
beat cloth	-0.044 ± 0.19 [-0.42; 0.34]	6 (0.54)	8	8	4	3(600, 647), 1(215, 603)
litter extraction	0.298 ± 0.27 [-0.24; 0.83]	3.5 (0.62)	6	6	3	3(527), 2(224), 1(678)
plant removal	-0.079 ± 0.22 [-0.51; 0.35]	5.1 (0.53)	7	7	4	3(637), 2(647), 1(215)
vac-aspirator	0.118 ± 0.38 [-0.62; 0.86]	3.8 (0.44)	5	5	3	3(244), 1(215, 620)
Opiliones - pitfall trap	0.449 ± 0.43 [-0.39; 1.29]	3.1 (0.55)	5	5	3	3(519), 1(516, 603)
Myriapoda - pitfall trap	-0.223 ± 0.24 [-0.69; 0.24]	8.3 (0.5)	10	10	5	3(166, 514), 2(516), 1(603)
litter extraction	-0.205 ± 0.31 [-0.81; 0.39]	2.1 (0.71)	5	5	3	3(527), 1(624, 678)
Oligochaeta - soil extraction	0.091 ± 0.19 [-0.28; 0.47]	4.1 (0.98)	13	8	6	6(628), 2(225, 674), 1(144, 508, 636)
Collembola - pitfall trap	-0.086 ± 0.10 [-0.28; 0.11]	31.7 (0.99)	55	32	10	28(675), 10(629), 4(171), 3(88, 527), 2(170, 516), 1(603, 651, 678)
soil extraction	-0.014 ± 0.13 [-0.27; 0.24]	14.3 (1)	32	28	9	11(90), 4(171, 225, 504), 3(657), 2(175, 674), 1(622, 644)
litter extraction	-0.126 ± 0.28 [-0.68; 0.43]	1 (0.97)	6	6	4	3(527), 1(624, 665, 678)
Nematoda - soil extraction	-0.254 ± 0.14 [-0.52; 0.01]	22 (0.14)	17	17	8	6(660), 2(608, 635, 653, 656), 1(90, 619, 645)
Coleoptera - Anthicidae - pitfall trap	0.437 ± 0.30 [-0.16; 1.03]	6.4 (0.27)	6	5	3	3(169), 2(675), 1(676)
Carabidae - pitfall trap	0.008 ± 0.07 [-0.12; 0.14]	74.5 (1)	110	89	33	22(675), 10(629), 6(88, 640), 4(169, 224, 615), 3(166, 219, 507, 527, 605, 610, 634, 639, 649, 654, 672), 2(170, 175, 516, 612, 614, 642), 1(228, 603, 613, 620, 643, 646, 669, 676, 678)
litter extraction	0.223 ± 0.27 [-0.31; 0.76]	4.7 (0.45)	6	6	3	3(527), 2(224), 1(678)
Chrysomelidae - sticky trap	-0.225 ± 0.13 [-0.48; 0.03]	52.6 (0.09)	41	23	5	24(675), 10(629), 3(167), 2(516, 666)

visual counts	-0.297 ± 0.17 [-0.63; 0.03]	**42.7 (0.03)**	28	17	7	10(629), 8(675), 4(169), 2(516, 673), 1(626, 677)
pitfall trap	-0.318 ± 0.19 [-0.68; 0.05]	4.5 (0.99)	15	7	3	8(675), 6(629), 1(144)
Cicindelidae - pitfall trap	0.198 ± 0.29 [-0.36; 0.76]	5.3 (0.51)	7	6	3	4(169), 2(507), 1(88)
Coccinellidae - visual counts	0.029 ± 0.08 [-0.13; 0.19]	69.5 (0.79)	81	69	28	10(629), 8(88), 5(182, 652, 659), 4(169, 601), 3(75, 527, 662), 2(10, 31, 38, 170, 211, 224, 246, 507, 516, 600, 621, 626, 673), 1(4, 230, 646, 676, 677)
sticky trap	**0.266 ± 0.10 [0.07; 0.46]**	53.8 (0.41)	53	37	10	21(173), 10(629), 8(659), 3(167, 224), 2(516, 637, 648), 1(230, 676)
Elateridae - pitfall trap	-0.143 ± 0.24 [-0.61; 0.33]	20.2 (0.12)	15	14	6	6(88), 3(169), 2(175, 516), 1(603, 676)
Nitidulidae - visual counts	0.116 ± 0.16 [-0.21; 0.44]	21.4 (0.26)	19	19	5	11(659), 3(169), 2(86, 170), 1(230)
Scarabaeidae - pitfall trap	-0.268 ± 0.29 [-0.84; 0.31]	14.6 (0.1)	10	9	5	4(169), 2(516, 675), 1(88, 166)
Staphylinidae - pitfall trap	-0.094 ± 0.08 [-0.25; 0.07]	61 (0.93)	80	60	23	19(675), 9(629), 6(88), 4(169, 224), 3(166, 521, 527, 605, 655, 672), 2(170, 175, 507, 516, 524), 1(182, 228, 603, 646, 676, 678)
sticky trap	**-0.428 ± 0.18 [-0.78; -0.07]**	11.2 (0.79)	17	8	6	10(629), 2(516, 524), 1(527, 675, 676)
litter extraction	0.100 ± 0.24 [-0.37; 0.57]	3 (0.89)	8	8	3	4(224), 3(527), 1(678)
Dermaptera - pitfall trap	-0.353 ± 0.19 [-0.72; 0.01]	9.8 (0.83)	16	8	3	14(675), 1(646, 676)
Diptera - Syrphidae - sticky trap	**-0.384 ± 0.12 [-0.61; -0.16]**	**67.3 (0.02)**	47	29	8	21(173), 10(629), 6(675), 3(167, 637), 2(648), 1(230, 659)
visual counts	-0.008 ± 0.17 [-0.33; 0.32]	7.5 (0.82)	13	10	6	7(652), 2(600), 1(38, 230, 601, 626)
Hemiptera - Anthocoridae - visual counts	**0.192 ± 0.08 [0.03; 0.35]**	70 (0.88)	86	66	24	14(88, 675), 10(629), 9(659), 6(182), 4(169), 3(75, 224), 2(10, 170, 211, 246, 516, 621, 626), 1(4, 38, 83, 230, 600, 646, 673, 676, 677)
sticky trap	-0.115 ± 0.09 [-0.28; 0.05]	74.3 (0.43)	74	48	10	22(675), 20(173), 10(629), 8(659), 4(224), 3(167, 527, 516), 1(230, 676)
plant removal	0.316 ± 0.18 [-0.04; 0.67]	26.5 (0.07)	18	17	7	3(31, 213, 604, 637, 647), 2(169), 1(228)
beat cloth	-0.009 ± 0.20 [-0.40; 0.38]	3.2 (0.78)	7	7	3	3(647, 648), 1(603)
Aphididae - visual counts	-0.045 ± 0.09 [0.22; 0.13]	58.6 (0.53)	61	43	19	10(629), 7(652), 6(225, 630, 667), 3(214, 662), 2(38, 516, 600, 612, 626, 632, 675), 1(75, 613, 659, 677)
sticky trap	-0.024 ± 0.13 [-0.29; 0.24]	20.5 (0.88)	30	21	7	10(629), 8(675), 3(167, 527, 659), 2(516), 1(230)
plant removal	-0.068 ± 0.16 [-0.38; 0.25]	14 (0.37)	14	14	6	3(213, 604, 637, 647), 1(228, 612)
beat cloth	**-0.449 ± -0.19 [-0.82; -0.08]**	2.6 (0.92)	8	8	4	3(600, 647), 1(603, 648)
Cicadellidae - sticky trap	0.022 ± 0.10 [-0.18; 0.22]	29.9 (0.97)	47	39	11	18(675), 11(659), 3(527, 647, 648, 658), 2(516), 1(144, 229, 230, 676)
visual counts	0.021 ± 0.13 [-0.23; 0.27]	25.1 (0.72)	31	23	10	10(675), 6(225), 4(169), 2(516, 600, 626, 632), 1(230, 673, 677)
sweep net	-0.106 ± 0.20 [-0.49; 0.28]	8.6 (0.28)	8	8	4	3(647, 648), 1(229, 600)
plant removal	0.225 ± 0.24 [-0.24; 0.69]	1.2 (0.94)	6	6	3	3(214), 2(647), 1(213)
Miridae - sticky trap	-0.021 ± 0.14 [-0.30; 0.26]	16.8 (0.82)	24	12	4	10(629, 675), 3(648), 1(676)

Nabidae - visual counts	-0.092 ± 0.14 [-0.37; 0.18]	8.3 (1)	24	17	8	10(629), 6(182), 2(211, 621), 1(169, 600, 646, 677)
Pentatomidae - visual counts	0.051 ± 0.24 [-0.42; 0.52]	2.7 (0.97)	10	8	5	4(675), 3(662), 1(169, 230, 516)
Hymenoptera - Braconidae - sticky trap	**-1.548 ± 0.24 [-2.02; -1.08]**	**61.6 (0.0001)**	27	18	4	21(173), 3(167), 2(516), 1(230)
Formicidae - pitfall trap	-0.128 ± 0.17 [-0.46; 0.21]	10.2 (0.9)	18	17	8	5(88), 4(169), 2(166, 175, 516), 1(603, 675, 678)
Neuroptera - sticky trap	-0.081 ± 0.09 [-0.25; 0.09]	57.7 (0.83)	70	43	10	26(675), 20(173), 10(629), 6(659), 2(144, 648), 1(167, 230, 516, 637)
visual counts	**0.223 ± 0.09 [0.04; 0.41]**	44 (0.86)	56	46	21	10(629), 7(652), 4(182, 659), 3(224, 527), 2(10, 38, 170, 211, 516, 600, 601, 621, 626, 673), 1(4, 75, 230, 677, 678)
plant removal	0.431 ± 0.47 [-0.50; 1.36]	**13.4 (0.02)**	6	6	3	3(604), 2(637), 1(228)
Orthoptera - pitfall trap	-0.094 ± 0.14 [-0.38; 0.19]	14.6 (0.95)	26	20	8	10(675), 4(169), 3(88, 166), 2(175, 516), 1(676, 678)
Thysanoptera - sticky trap	-0.078 ± 0.13 [-0.34; 0.18]	22.9 (0.85)	32	18	6	16(675), 10(629), 3(527), 1(230, 676, 678)
plant removal	-0.083 ± 0.14 [-0.36; 0.20]	13.8 (0.68)	18	18	7	4(169), 3(213, 604, 637, 647), 1(228, 612)
visual counts	0.199 ± 0.16 [-0.12; 0.52]	14.7 (0.62)	18	15	10	4(629), 2(38, 600, 612, 614, 626), 1(613, 676, 677, 678)
beat cloth	0.047 ± 0.18 [-0.31; 0.40]	10.5 (0.31)	10	10	4	3(600, 647, 648), 1(603)
**Life stages (eggs and juveniles)**						
Coleoptera - Carabidae - larvae	0.102 ± 0.35 [-0.58; 0.79]	8.8 (0.18)	7	7	3	3(527), 2(516, 640)
Coccinellidae - eggs	-0.042 ± 0.25 [-0.54; 0.45]	8.8 (0.36)	9	8	4	3(527, 652), 2(516), 1(10)
larvae & pupae	-0.052 ± 0.15 [-0.34; 0.24]	12.8 (0.96)	26	19	11	8(629), 3(31, 75, 527), 2(10, 516), 1(4, 230, 600, 648, 652)
Diptera - Syrphidae - larvae & pupae	0.298 ± 0.26 [-0.21; 0.80]	8.6 (0.2)	7	6	3	5(652), 1(230, 600)
Tachinidae - larvae & pupae	**-0.956 ± 0.39 [-1.72; -0.19]**	8.8 (0.07)	5	5	4	2(617), 1(38, 230, 618)
Hemiptera - Anthocoridae - nymphs	0.189 ± 0.14 [-0.09; 0.47]	25.7 (0.64)	30	20	7	11(88), 10(629), 3(75), 2(31, 246), 1(4, 83)
Neuroptera - eggs	**0.502 ± 0.15 [0.21; 0.79]**	19.9 (0.65)	24	16	8	10(629), 3(527, 604), 2(10, 516, 652), 1(75, 228)

Table S7.12: Moderator analyses target order (Lepidoptera-active, Coleoptera-active, or stacked) of subgroup analyses on sampling methods and life stages (juveniles or eggs). Records with any "red" critical appraisal label were excluded. Given is the analyzed taxon and sampling method or life stage, the estimated effect size (estimate) with standard error (SE) and 95% confidence interval (lower boundary; upper boundary), and the number of records (rec), experiments (exp) and articles (art) included in the respective analysis. Significant effect sizes (confidence intervals do not include zero) are marked in bold.

Taxon	Lepidoptera-active Estimate ± SE [ci.lb; ci.ub]	Rec	Exp	Art	Coleoptera-active Estimate ± SE [ci.lb; ci.ub]	Rec	Exp	Art	Lep.&Col. stacks Estimate ± SE [ci.lb; ci.ub]	Rec	Exp	Art
Sampling methods												
Acarina - soil extraction	0.095 ± 0.23 [-0.35; 0.54]	10	10	4								
pitfall trap	-0.164 ± 0.26 [-0.68; 0.35]	8	8	3								
litter extraction	0.231 ± 0.35 [-0.45; 0.92]	5	5	3								
Araneae - pitfall trap	-0.075 ± 0.10 [-0.27; 0.12]	50	48	16	0.279 ± 0.17 [-0.05; 0.61]	18	17	6	0.061 ± 0.17 [-0.28; 0.40]	17	15	4
visual counts	0.076 ± 0.11 [-0.14; 0.29]	45	40	17	0.267 ± 0.22 [-0.17; 0.70]	11	11	3	0.066 ± 0.21 [-0.34; 0.48]	11	9	5
sticky trap	0.255 ± 0.14 [-0.02; 0.53]	27	18	5								
beat cloth	-0.040 ± 0.27 [-0.57; 0.49]	5	5	3								
vac-aspirator	0.118 ± 0.38 [-0.62; 0.86]	5	5	3								
Opiliones - pitfall trap	0.449 ± 0.43 [-0.39; 1.29]	5	5	3								
Myriapoda - pitfall trap	0.149 ± 0.34 [-0.52; 0.81]	6	6	3								
Oligochaeta - soil extraction	0.122 ± 0.22 [-0.30; 0.54]	10	7	5								
Collembola - pitfall trap	-0.090 ± 0.15 [-0.38; 0.20]	25	24	7	-0.052 ± 0.22 [-0.49; 0.38]	11	10	4	-0.100 ± 0.16 [-0.42; 0.22]	19	17	3
soil extraction	0.104 ± 0.18 [-0.26; 0.46]	15	13	6	-0.137 ± 0.19 [-0.50; 0.23]	17	15	3				
litter extraction	-0.292 ± 0.35 [-0.97; 0.39]	5	5	3								
Nematoda - soil extraction	-0.298 ± 0.18 [-0.64; 0.05]	9	9	3	-0.081 ± 0.30 [-0.67; 0.51]	6	6	4				
Coleoptera -Carabidae - pitfall trap	0.046 ± 0.09 [-0.12; 0.22]	66	64	24	-0.027 ± 0.14 [-0.30; 0.24]	24	23	8	-0.060 ± 0.15 [-0.35; 0.23]	20	18	5
Chrysomelidae - sticky trap	0.077 ± 0.19 [-0.30; 0.46]	16	15	4	**-0.648 ± 0.30 [-1.24; -0.05]**	7	7	3				
visual counts	-0.134 ± 0.24 [-0.61; 0.34]	15	15	6					-0.455 ± 0.29 [-1.03; 0.12]	10	8	3
Coccinellidae - visual counts	-0.071 ± 0.10 [-0.26; 0.12]	56	53	23	0.241 ± 0.19 [-0.12; 0.60]	18	16	4	0.393 ± 0.28 [-0.15; 0.94]	7	5	3
sticky trap	**0.280 ± 0.14 [0.01; 0.55]**	29	20	5	0.210 ± 0.18 [-0.14; 0.56]	16	16	4	0.321 ± 0.23 [-0.13; 0.77]	8	6	3
Elateridae - pitfall trap	-0.532 ± 0.39 [-1.30; 0.24]	6	6	3								
Nitidulidae - visual counts	-0.100 ± 0.25 [-0.58; 0.38]	8	8	4								
Scarabaeidae - pitfall trap	-0.114 ± 0.30 [-0.71; 0.48]	7	7	3								
Staphylinidae - pitfall trap	-0.149 ± 0.11 [-0.36; 0.06]	47	44	16	0.043 ± 0.17 [-0.28; 0.37]	19	17	7	-0.098 ± 0.19 [-0.47; 0.27]	14	12	5
sticky trap	-0.460 ± 0.34 [-1.13; 0.21]	5	5	3					-0.382 ± 0.26 [-0.90; 0.13]	8	5	4
Diptera - Syrphidae - sticky trap	**-0.476 ± 0.16 [-0.80; -0.16]**	26	17	4	-0.377 ± 0.25 [-0.86; 0.10]	10	10	4	-0.207 ± 0.24 [-0.67; 0.26]	11	9	3
visual counts	-0.008 ± 0.17 [-0.33; 0.32]	13	10	6								
Hemiptera - Anthocoridae - visual counts	**0.272 ± 0.11 [0.05; 0.49]**	49	44	19	0.014 ± 0.14 [-0.27; 0.30]	29	24	5	0.285 ± 0.20 [-0.11; 0.68]	13	11	4
sticky trap	-0.102 ± 0.12 [-0.33; 0.13]	41	31	7	-0.170 ± 0.19 [-0.54; 0.20]	15	15	4	-0.096 ± 0.18 [-0.44; 0.25]	18	16	3
plant removal	0.345 ± 0.23 [-0.12; 0.80]	12	11	5								
Aphididae - visual counts	-0.123 ± -994 [-0.32; 0.07]	50	40	17					0.248 ± 0.28 [-0.30; 0.79]	7	5	3
sticky trap	0.093 ± 0.23 [-0.36; 0.55]	10	10	5	0.043 ± 0.24 [-0.43; 0.51]	9	9	3				
plant removal	-0.149 ± 0.22 [-0.58; 0.29]	8	8	4								
Cicadellidae - sticky trap	-0.081 ± 0.18 [-0.43; 0.27]	17	16	6	0.155 ± 0.17 [-0.17; 0.48]	16	16	4	-0.028 ± 0.18 [-0.39; 0.33]	14	14	3
visual counts	0.090 ± 0.14 [-0.19; 0.37]	25	22	9								
Miridae - sticky trap									-0.004 ± 0.18 [-0.35; 0.35]	14	12	4
Nabidae - visual counts	-0.185 ± 0.17 [-0.53; 0.16]	15	15	7								
Pentatomidae - visual counts	-0.005 ± 0.26 [-0.52; 0.51]	8	8	5								

Formicidae - pitfall trap	-0.056 ± 0.27 [-0.59; 0.48]	8	8	4	-0.331 ± 0.24 [-0.81; 0.15]	9	7	3				
Neuroptera - sticky trap	-0.112 ± 0.12 [-0.35; 0.13]	35	26	5	0.066 ± 0.20 [-0.32; 0.46]	13	13	5	-0.121 ± 0.15 [-0.42; 0.18]	22	20	3
visual counts	0.169 ± 0.11 [-0.04; 0.38]	42	39	18					**0.525 ± 0.26 [0.02; 1.03]**	7	5	3
Orthoptera - pitfall trap	-0.036 ± 0.23 [-0.49; 0.41]	11	10	3	-0.093 ± 0.25 [-0.58; 0.40]	9	9	4	-0.178 ± 0.28 [-0.72; 0.36]	6	6	3
Thysanoptera - sticky trap	-0.056 ± 0.21 [-0.47; 0.36]	12	11	4					-0.104 ± 0.19 [-0.47; 0.26]	16	14	4
plant removal	-0.167 ± 0.18 [-0.53; 0.19]	12	12	5								
visual counts	0.216 ± 0.20 [-0.18; 0.61]	12	12	7					0.091 ± 0.29 [-0.48; 0.66]	5	4	4
**Life stages (eggs and juveniles)**												
Coleoptera - Carabidae - larvae	0.102 ± 0.35 [-0.58; 0.79]	7	7	3								
Coccinellidae - eggs	-0.042 ± 0.25 [-0.54; 0.45]	9	8	4								
larvae & pupae	-0.057 ± 0.16 [-0.38; 0.26]	19	18	10								
Diptera - Syrphidae - larvae & pupae	0.298 ± 0.26 [-0.21; 0.80]	7	6	3								
Tachinidae - larvae & pupae	**-0.956 ± 0.39 [-1.72; -0.19]**	5	5	4								
Hemiptera - Anthocoridae - nymphs	0.153 ± 0.25 [-0.34; 0.64]	9	9	5	-0.083 ± 0.20 [-0.47; 0.31]	16	13	3				
Neuroptera - eggs	**0.403 ± 0.18 [0.05; 0.75]**	16	15	8								

Table S7.13: Meta-analyses of insecticide-treated non-Bt maize for all Bt proteins and records with any "red" critical appraisal label excluded. Given is the analyzed taxon, the estimated effect size (estimate) with standard error (SE) and 95% confidence interval (lower boundary; upper boundary), the measure of heterogenetiy (Q value and significance, p<0.05 is significant), the number of records (rec), experiments (exp) and articles (art) included in the respective analysis, and the number of records per article (articleID in parenthesis). Significant heterogeneity and significant effect sizes (confidence intervals do not include zero) are marked in bold.

Taxon	Estimate ± SE [ci.lb; ci.ub]	Q (p)	Rec	Exp	Art	Records per article
**Pyrethroids**						
**All taxa**	**0.192 ± 0.04 [0.11; 0.27]**	**796.7 (<0.0001)**	466	49	33	
**Decomposers**	0.030 ± 0.15 [-0.26; 0.32]	**73.2 (0.0001)**	35	13	9	
**Herbivores**	0.134 ± 0.12 [-0.09; 0.36]	**208.1 (<0.0001)**	88	26	14	
**Parasitoids**	0.227 ± 0.16 [-0.09; 0.54]	**74.6 (0.0002)**	38	21	12	
**Predators**	**0.240 ± 0.05 [0.15; 0.33]**	**374 (<0.0001)**	262	47	26	
Acarina	-0.015 ± 0.24 [-0.49; 0.46]	1.7 (0.97)	8	6	6	2(90, 144), 1(603, 609, 622, 633)
Araneae	**0.588 ± 0.11 [0.37; 0.80]**	27.6 (0.93)	41	20	12	6(166), 5(224, 225), 4(167, 170, 516), 3(600, 639), 2(88, 603, 676), 1(215)
Myriapoda	-0.180 ± 0.25 [-0.68; 0.32]	5.1 (0.75)	9	6	3	6(166), 2(516), 1(603)
Collembola	-0.086 ± 0.18 [-0.45; 0.27]	0.3 (0.32)	14	13	8	2(88, 170, 171, 213, 225, 516), 1(603, 622)
**Coleoptera**	**0.179 ± 0.07 [0.03; 0.33]**	**262.7 (<0.0001)**	141	42	22	
Cantharidae (Col.)	**1.294 ± 0.35 [0.61; 1.98]**	1.7 (0.89)	6	6	3	3(224), 2(516), 1(603)
Carabidae (Col.)	0.186 ± 0.10 [-0.01; 0.39]	46 (0.31)	43	39	14	6(166, 640), 4(224, 615, 661), 3(634, 639, 649), 2(88, 170, 516, 642), 1(603, 676)
Coccinellidae (Col.)	**0.450 ± 0.19 [0.08; 0.82]**	**47.9 (0.0007)**	22	19	9	6(167), 3(224, 648), 2(31, 170, 516, 600), 1(603, 676)
Elateridae (Col.)	-0.253 ± 0.56 [-1.35; 0.85]	**12.2 (0.03)**	6	5	4	2(88, 516), 1(603, 676)
Lathrididae (Col.)	1.691 ± 1.30 [-0.86; 4.24]	**27.3 (<0.0001)**	6	5	3	3(213), 2(144), 1(603)
Nitidulidae (Col.)	**-0.66 ± 0.25 [-1.14; -0.18]**	6 (0.74)	10	7	3	6(166), 2(170, 516)
Staphylinidae (Col.)	-0.269 ± 0.15 [-0.57; 0.03]	22.7 (0.48)	24	19	9	6(166), 4(224, 663), 2(88, 170, 516, 524), 1(603, 676)
**Diptera**	0.025 ± 0.19 [-0.34; 0.39]	**71.2 (<0.0001)**	33	14	6	
Syrphidae (Dip.)	0.018 ± 0.20 [-0.37; 0.40]	14.6 (0.15)	11	8	4	6(167), 2(600, 648), 1(603)
**Hemiptera**	**0.424 ± 0.10 [0.23; 0.62]**	**189.3 (<0.0001)**	97	31	14	
Anthocoridae (Hem.)	**0.644 ± 0.17 [0.37; 1.02]**	**39.1 (0.04)**	26	21	11	6(167), 4(224), 3(31), 2(88, 170, 213, 516, 648), 1(600, 603, 676)
Aphididae (Hem.)	**-0.332 ± 0.17 [-0.66; -0.01]**	35.2 (0.07)	25	19	7	6(167, 225, 667), 3(600), 2(516), 1(603, 648)
Cicadellidae (Hem.)	**0.855 ± 0.26 [0.34; 1.37]**	**34.3 (0.003)**	16	13	7	5(225), 3(648), 2(144, 516, 600), 1(603, 676)
Nabidae (Hem.)	0.237 ± 0.25 [-0.25; 0.72]	5.8 (0.56)	8	5	3	6(167), 1(600, 603)
**Hymenoptera**	-0.014 ± 0.11 [-0.23; 0.21]	**92.4 (0.002)**	58	22	14	
Formicidae (Hym.)	**-0.484 ± 0.21 [-0.89; -0.07]**	5.3 (0.92)	12	8	5	6(166), 2(88, 516), 1(600, 603)
Neuroptera	-0.069 ± 0.18 [-0.41; 0.28]	20.9 (0.23)	18	15	8	4(144), 3(224), 2(167, 170, 516, 600, 648), 1(603)

Orthoptera	-0.154 ± 0.25 [-0.64; 0.33]	4.3 (0.83)	9	6	3	6(166), 2(516), 1(676)
Thysanoptera	0.195 ± 0.34 [-0.48; 0.87]	**13.8 (0.03)**	7	7	5	2(600, 648), 1(213, 603, 676)
**Chloro-nicotinyl**						
**All taxa**	0.116 ± 0.06 [-0.01; 0.24]	101.7 (0.89)	121	12	9	
**Decomposers**	-0.001 ± 0.18 [-0.36; 0.36]	11.7 (0.47)	13	8	5	
**Herbivores**	-0.143 ± 0.17 [-0.48; 0.19]	15.2 (0.3)	14	4	4	
**Parasitoids**	-0.041 ± 0.24 [-0.51; 0.43]	2.6 (0.86)	7	4	3	
**Predators**	**0.218 ± 0.08 [0.06; 0.37]**	65.4 (0.86)	80	9	7	
Araneae	**0.514 ± 0.18 [0.15; 0.87]**	4 (1)	15	9	4	5(182), 3(166, 672), 2(167, 678)
Myriapoda	-0.049 ± 0.27 [-0.57; 0.47]	3 (0.8)	7	7	3	3(166, 514), 1(678)
Collembola	-0.201 ± 0.27 [-0.73; 0.33]	3.7 (0.6)	6	6	3	4(171), 1(504, 678)
**Coleoptera**	-0.022 ± 0.12 [-0.26; 0.21]	33.2 (0.51)	35	9	7	
Carabidae (Col.)	0.187 ± 0.27 [-0.34; 0.71]	2.8 (0.84)	7	7	3	3(166, 672), 1(678)
Staphylinidae (Col.)	-0.076 ± 0.28 [-0.62; 0.46]	7.8 (0.35)	8	8	4	3(166, 672), 1(182, 678)
**Hemiptera**	**0.379 ± 0.17 [0.05; 0.71]**	20.5 (0.43)	21	9	4	
**Hymenoptera**	0.045 ± 0.23 [-0.40; 0.49]	9.3 (0.41)	10	4	4	
Neuroptera	0.034 ± 0.26 [-0.48; 0.55]	0.6 (1)	7	7	4	3(182), 2(144), 1(167, 678)
**Organophosphorous**						
**all Taxa**	0.160 ± 0.09 [-0.02; 0.34]	34.7 (0.95)	51	4	6	
**Predators**	**0.355 ± 0.17 [0.02; 0.69]**	20.8 (0.7)	26	5	3	
**Coleoptera**	0.260 ± 0.18 [-0.09; 0.61]	7 (0.86)	13	3	3	
**Microbial**						
**All taxa**	-0.008 ± 0.12 [-0.25; 0.24]	63.8 (0.19)	56	5	3	
**Predators**	**0.281 ± 0.12 [0.04; 0.52]**	20.4 (0.81)	28	4	5	
**Coleoptera**	0.210 ± 0.24 [-0.26; 0.68]	9 (0.7)	13	5	3	

Table S7.14: Results of robustness analyses for taxa or groups that showed significant effect sizes in the different meta-analyses. Given is the taxon or group, the direction of the effect ("+" positive or "-" negative), the record selection based on critical appraisal, and the target order of the Bt proteins in the respective analyses. Fail safe numbers indicate the number of studies with effect size 0 that need to be added to the analysis to turn the outcome to non-significant. In parenthesis is the threshold of 5n + 10 according to Rosenberg (Evolution 2005, 59: 464-468, doi), which indicates that a reported effect is robust. "Leave one out" refers to the number of analyses that turn to non-significant when repeatedly fitting the model, leaving one record/ experiment/ article out at a time. For example, 2 of 7 indicates that 2 analyses out of 7 turn to non-significant. For the "leave one out" analyses with articles, the articleIDs of the articles that turn the analysis to non-significant also is given. Robust fail safe numbers as well as "leave one out" values of 0 are in bold.

Taxon/group	Direction of effect	Critical appraisal	Target order Bt proteins	Fail safe number (5n+10)	Leave one out			ArticleID
					Record	Experiment	Article	
**Untreated Bt and non-Bt maize**								
All taxa[1]	-	All records	All	38 (11110)	na	na	na	
Col: Coccinellidae	+	All green	All	10 (200)	6 of 38	6 of 29	4 of 13	173, 626, 648, 677
sticky traps	+	No red	All	48 (275)	**0** of 53	**0** of 37	1 of 10	173
H. axyridis	+	No red	All	2 (45)	4 of 7	4 of 7	3 of 5	167, 626, 648
Nitidulidae	-	No red	Lepidoptera	3 (65)	2 of 11	2 of 11	3 of 5	170, 230, 516
Staphylinidae	-	No red	All	27 (415)	**0** of 81	**0** of 62	2 of 24	88, 629
	-	All records	All	22 (450)	1 of 88	1 of 67	4 of 27	88, 230, 629, 672
sticky traps	-	No red	All	8 (95)	1 of 17	1 of 8	1 of 6	629
Diptera	-	No red	All	380 (860)	**0** of 170	**0** of 78	**0** of 26	
	-	No red	Lepidoptera	255 (510)	**0** of 100	**0** of 54	**0** of 19	
	-	No red	C&L-stacks	15 (175)	**0** of 33	2 of 17	1 of 4	675
	-	All records	All	459 (975)	**0** of 193	**0** of 91	**0** of 33	
	-	All green	All	83 (265)	**0** of 51	1 of 22	2 of 7	173, 603
Syrphidae	-	All records	All	26 (370)	**0** of 72	4 of 51	2 of 19	173, 629
	-	All green	All	9 (175)	5 of 33	5 of 21	1 of 7	173
sticky traps	-	No red	All	95 (245)	**0** of 47	**0** of 29	1 of 8	173
Tachinidae	-	No red	All	17 (85)	**0** of 15	**0** of 11	1 of 5	617
	-	No red	Lepidoptera	15 (55)	**0** of 9	**0** of 9	**0** of 5	
	-	All records	All	17 (85)	**0** of 15	**0** of 11	1 of 5	617
larvae & pupae	-	No red	All	8 (35)	1 of 5	1 of 5	1 of 4	38
Hem: Anthocoridae	+	No red	Lepidoptera	15 (410)	11 of 80	10 of 69	10 of 26	31, 169, 182, 211, 224, 228, 600, 626, 629, 673
visual counts	+	No red	All	40 (440)	**0** of 86	1 of 66	1 of 24	629
Aphididae - beat cloth	-	No red	All	4 (50)	1 of 8	1 of 8	1 of 4	647
Hymenoptera	-	No red	All	133 (800)	**0** of 158	**0** of 95	1 of 38	173
	-	No red	Lepidoptera	211 (630)	**0** of 124	**0** of 70	1 of 27	173
	-	All records	All	214 (985)	**0** of 195	**0** of 109	1 of 45	173
	-	All green	All	**298** (245)	**0** of 47	**0** of 22	1 of 7	173
Braconidae	-	No red	All	**439** (145)	**0** of 27	**0** of 18	1 of 7	173
	-	No red	Lepidoptera	**424** (130)	**0** of 24	**0** of 15	1 of 3	173
	-	All records	All	**426** (175)	**0** of 33	**0** of 24	1 of 7	173
sticky traps	-	No red	All	**439** (145)	**0** of 27	**0** of 18	1 of 7	173
M. cingulum	-	No red	All	**474** (140)	**0** of 26	**0** of 17	**0** of 3	
Neuroptera, visual counts	+	No red	All	27 (56)	**0** of 56	**0** of 46	1 of 21	629

eggs	+	No red	All	49 (130)	**0** of 24	**0** of 16	1 of 8	629
Herbivores	-	No red	C&L-stacks	86 (610)	**0** of 120	2 of 28	2 of 7	629, 675
Parasitoids	-	No red	All	354 (685)	**0** of 135	**0** of 86	1 of 35	173
	-	No red	Lepidoptera	444 (540)	**0** of 106	**0** of 66	1 of 27	173
Private sector contribution	-	No red	All	1668 (4380)	**0** of 874	**0** of 76	1 of 22	675
	-	No red	Lepidoptera	609 (2380)	**0** of 474	**0** of 47	2 of 12	173, 675
	-	No red	C&L-stacks	157 (1150)	**0** of 228	1 of 23	1 of 5	675
**Pyrethroid insecticide applied to non-Bt maize, untreated Bt maize**								
All taxa	+	No red	All	**3081** (2340)	**0** of 466	**0** of 49	**0** of 33	
Araneae	+	No red	All	**265** (215)	**0** of 41	**0** of 20	**0** of 12	
Coleoptera	+	No red	All	101 (715)	**0** of 141	2 of 42	1 of 22	516
Cantharidae	+	No red	All	16 (40)	**0** of 6	**0** of 6	**0** of 3	
Coccinellidae	+	No red	All	16 (120)	**0** of 22	**0** of 19	1 of 9	31
Nitidulidae	-	No red	All	9 (60)	**0** of 10	**0** of 7	**0** of 3	
Hemiptera	+	No red	All	**531** (495)	**0** of 97	**0** of 31	**0** of 14	
Hem: Anthocoridae	+	No red	All	107 (140)	**0** of 26	**0** of 21	**0** of 11	
Aphididae	-	No red	All	17 (135)	13 of 25	9 of 19	4 of 7	225, 516, 600, 648
Cicadellidae	+	No red	All	74 (90)	**0** of 16	**0** of 13	**0** of 7	
Hym: Formicidae	-	No red	All	5 (70)	1 of 12	3 of 8	2 of 5	88, 166
Predators	+	No red	All	**1680** (1320)	**0** of 262	**0** of 47	**0** of 26	
**Chloro-nicotinyl insecticide applied to non-Bt maize, untreated Bt maize**								
Araneae	+	No red	All	16 (85)	**0** of 15	**0** of 9	**0** of 5	
Hemiptera	+	No red	All	9 (115)	2 of 21	4 of 9	2 of 4	167, 182
Predators	+	No red	All	76 (410)	**0** of 80	1 of 9	1 of 7	678
**Microbial insecticide applied to non-Bt maize, untreated Bt maize**								
Predators	+	No red	All	3 (140)	10 of 26	4 of 5	2 of 3	31, 661
**Organophosphorous insecticide applied non-Bt maize, untreated Bt maize**								
Predators	+	No red	All	10 (150)	**0** of 28	1 of 4	2 of 5	654, 655

[1] "leave one out" analyses not performed for all taxa because of immense calculation power needed for all possible iterations

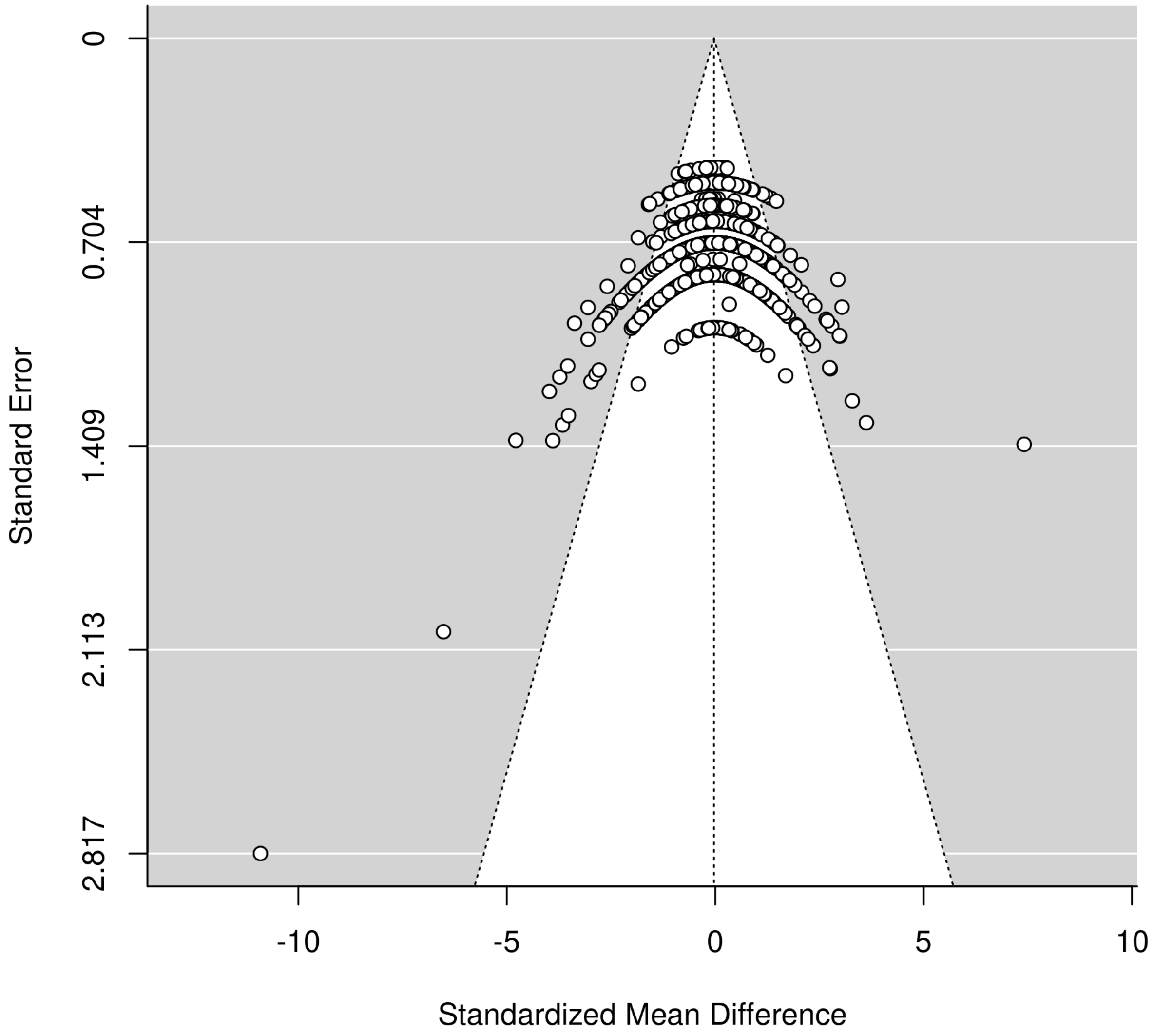

Figure S7.1: Funnel plot with standardized mean difference (x-axis) and standard error (y-axis). Symmetrical distribution of data indicates lack of publication bias. Records with any red flag in the critical appraisal were excluded.

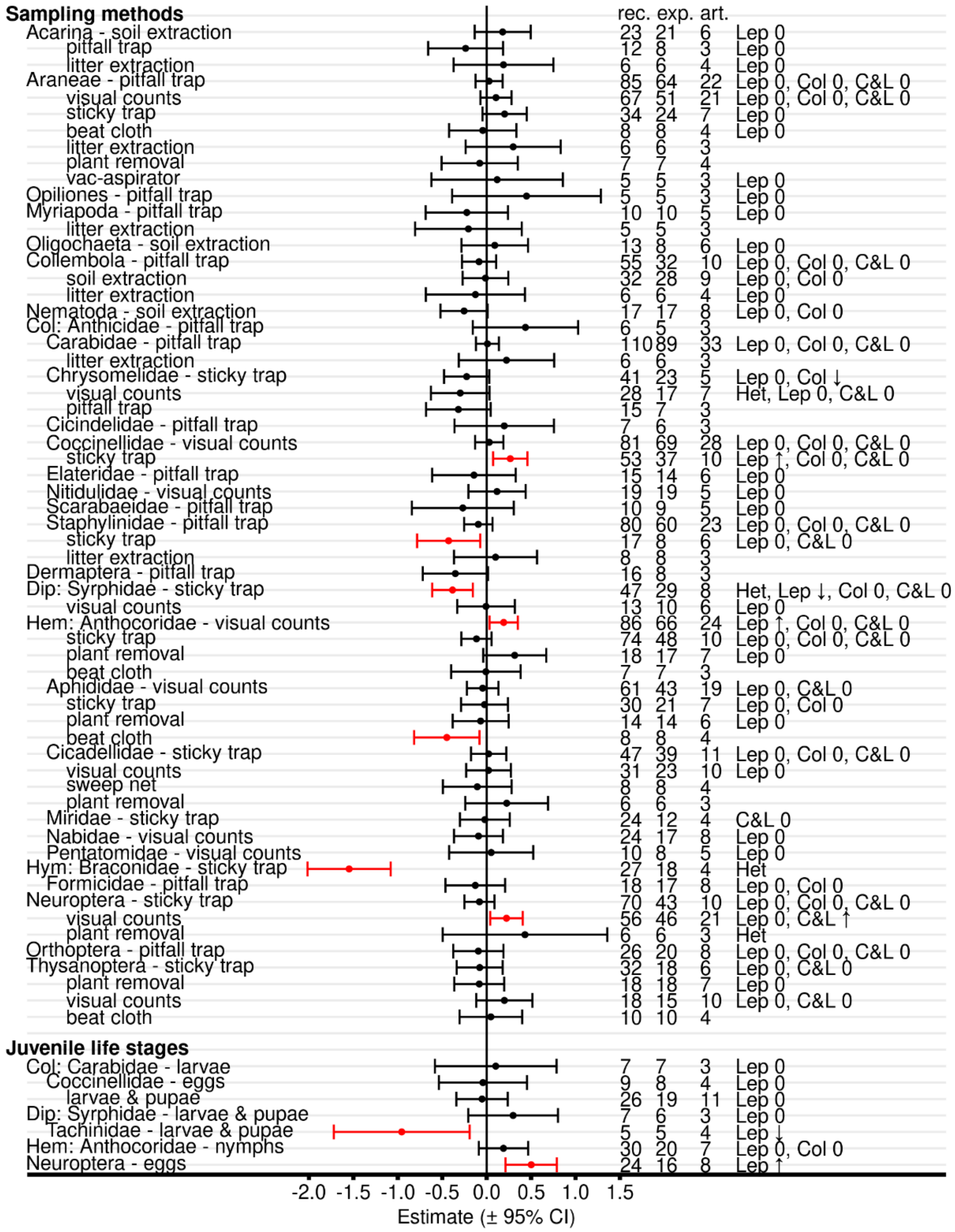

Figure S7.2: Subgroup-analyses on sampling methods and juvenile life stages for untreated Bt and non-Bt maize. Records with any red flag in the critical appraisal were excluded. All Bt proteins were included. For each taxon, the effect size estimate and the 95% confidence interval is given. Significant intervals (red) do not include 0. On the right side is the number of records (rec.), experiments (exp.), and articles (art.) included in each analysis. For details see Table S7.11. Results of moderator analyses with target order of Bt proteins (Table S7.12) are indicated: ↑ higher values in Bt compared with non-Bt treatment (positive effect size), ↓ lower values in Bt compared with non-Bt treatment (negative effect size), 0: no effect (Lep = Lepidoptera-active, Col = Coleoptera-active, C&L = stacked Lepidoptera- & Coleoptera-active).
